# Combined Systemic Intake of K-ATP Opener (Nicorandil) and Mesenchymal Stem Cells Preconditioned With Nicorandil Alleviates Pancreatic Insufficiency in a Model of Bilateral Renal Ischemia/Reperfusion Injury

**DOI:** 10.3389/fphys.2022.934597

**Published:** 2022-06-23

**Authors:** Asmaa Mohammed ShamsEldeen, Sarah A. Abd El-Aal, Basma Emad Aboulhoda, Hend AbdAllah, Sara Mahmoud Gamal, Fatma E. Hassan, Marwa Nagi Mehesen, Laila Ahmed Rashed, Abeer Mostafa, Nermeen Bakr Sadek

**Affiliations:** ^1^ Department of Physiology, Kasr Alainy, Faculty of Medicine, Cairo University, Giza, Egypt; ^2^ Department of Pharmacy, Kut University College‐ Al Kut, Wasit, Iraq; ^3^ Department of Anatomy and Embryology, Kasr Alainy, Faculty of Medicine, Cairo University, Giza, Egypt; ^4^ Department of Medical Pharmacology, Kasr Alainy, Faculty of Medicine, Cairo University, Giza, Egypt; ^5^ Department of Medical Biochemistry and Molecular Biology, Kasr Alainy, Faculty of Medicine, Cairo University, Giza, Egypt

**Keywords:** MSc, nicorandil, bilateral renal I/R, PI3K/AKT/mTOR, pancreatic damage

## Abstract

We used nicorandil, a K-ATP channel opener, to study the role of these channels in the amelioration of renal ischemia/reperfusion (I/R)-induced pancreatic injury, and the possible involvement of PI3K/Akt/mTOR signaling pathway. Forty-two male Wistar rats were included in this study, six were sacrificed for extraction of bone marrow mesenchymal stem cells (BM-MSCs) and conducting the *in-vitro* work, the others were included *in vivo* study and equally divided into six groups. Group 1 (sham control), but groups 2–6 were subjected to bilateral renal I/R: Group 2 (I/R); Group 3 (I/R-NC), treated with nicorandil; Group 4 (I/R-MSCs), treated with BM-MSCs; Group 5 (I/R-MSCC), treated with nicorandil-preconditioned BM-MSCs; Group 6 (I/R-NC-MSCC), treated with both systemic nicorandil and preconditioned BM-MSCC. Renal injury and subsequent pancreatic damage were detected in the I/R group by a significant increase in serum urea, creatinine, fasting glucose, and pancreatic enzymes. The pancreatic tissues showed a reduction in cellularity and a significant decrease in the expression of the cell survival pathway, PI3K/Akt/mTOR, in the I/R group compared to the control. Preconditioning MSCs with nicorandil significantly enhanced the proliferation assay and decreased their apoptotic markers. Indeed, combined systemic nicorandil and nicorandil-preconditioning maintained survival of MSC in the pancreatic tissue and amelioration of apoptotic markers and pancreatic TNF-α production. Histologically, all treated groups revealed better pancreatic architecture, and increased area % of anti-insulin antibody and CD31, which were all best observed in the NC-MSCC group. Thus, using K-ATP channel opener was efficient to enhance PI3K/Akt/mTOR expression levels (*in vivo* and *in vitro*).

## 1 Introduction

Renal ischemia/reperfusion injury (I/R) is one of the unavoidable co-incidences during kidney transplantation or vascular surgery. Tissue ischemia causes multiple series events that progress to cell injury, dysfunction, necrosis, and death (Kocaturk et al., 2020). Moreover, reperfusion exacerbates this damage *via* enhancing the production of reactive oxygen species (ROS) and inflammatory response (Yapca et al., 2013). Renal ischemia/reperfusion induces multi-organ damage including pancreatic injury (Hussein et al., 2014). Abogresha and colleagues (2016) depicted the contribution of ROS as an important cause of pancreatic cell death following renal I/R injury. In this context, M El Agaty and Ahmed (2020) reported a significant decrease in area % of insulin immune reactivity denoting a declined β-cell number in rats exposed to renal I/R injury for 45 min versus sham-operated group.

K-ATP channels are found in beta cells and in other extra-pancreatic tissues. In pancreatic tissues, K-ATP channels regulate insulin secretion. By inhibiting these channels, the beta cells depolarize and Ca^2+^ influx occurs, on the contrary opening K-ATP channels leads to hyperpolarization of the beta cells and prevents insulin release (Ashcroft and Gribble. 1999). Worthy, the activation of K-ATP channels in cardiac muscles protects the heart during ischemia by limiting Ca^2+^ entry. Even more, activation of these channels in blood vessels causes membrane hyperpolarization, thus it could inhibit voltage-sensitive Ca^2+^-channels eventually causing vasodilation (Nichols, 2016). In brain tissues, the K-ATP opener exerts neuroprotective anti-neuroinflammatory effects against trauma and ischemia (Zhou et al., 2008). Previous data showed inhibition of rotenone-induced production of tumor necrosis factor-alpha (TNF-α) and ROS in brain tissues of rats treated with K-ATP opener (Zhou et al., 2008). One of the K-ATP openers is a 2-nicotiamidoethyl-nitrate ester (Nicorandil), it is the only mitochondrial K-ATP opener and also acts as nitric oxide (NO) donor (Zhang et al., 2015). Indeed; Horinaka et al, (2001) reported that nicorandil enhanced the cardiac eNOS levels in rats, secondary to its K-ATP opening effect. In a dose-dependent manner, nicorandil succeeded in amelioration of acute tubular damage observed in rats exposed to unilateral renal I/R for 30 min (Shimizu et al., 2011), however, its therapeutic efficacy in the remote effects of prolonged bilateral I/R injury of the kidneys wasn’t investigated.

Mesenchymal stem cells (MSCs) have been widely studied in regenerative medicine. With its impressive paracrine ability, anti-inflammatory, and differentiation capacity, MSCs represent one of the most ideal lines for tissue regeneration in several diseases such as ischemic conditions (Hu and Zou. 2017). However, the major challenge for the therapeutic potential of MSCs is the poor survival rate following transplantation. In Zhang and colleagues’ study (2015), nicorandil enhanced PI3K/Akt pathway in stem cells exposed to hypoxia, therefore protecting them from apoptosis and decreased ROS production in culture media. The PI3K/Akt is a pro-survival pathway that controls cell apoptosis. Akt control pancreatic cell plasticity, consequently Akt/mTOR inhibition induces both autophagy and apoptotic cell death (Yu et al., 2018). Thus, it is likely to target the mitochondrial K-ATP role as a novel regulator against ischemia-reperfusion hazards. By administration of the mitochondrial K-ATP opener (nicorandil), that was used *in vitro* for MSCs preconditioning or *in vivo* in male rats exposed to bilateral renal I/R injury. We speculated that nicorandil may enhance the survival of MSCs *in vitro* and together with its *in vivo* intake could optimize pancreatic protection from the remote effect of bilateral renal ischemia for 45 min and reperfusion for 7 days.

## 2 Materials and Methods

### 2.1 Animals

A total of forty-two male Wistar rats (aged 10–12 months and of body weight 180–200 g) were included in this study. Six of them were used for isolation and expansion of MSCs and the others were included in the *in vivo* study. All experimental work and animal procedures were done in accordance with the international guidelines of Helsinki. Part of this assurance includes the establishment of an appropriated constituted Institutional Animal Care and Use Committee that had the responsibility to review and monitor research involving experimental animals. Animals were purchased from and inbred in the Experimental Animal Unit, Faculty of Medicine, Cairo University. Male rats were maintained in specific pathogen-free conditions, fed normal animal chow, and were subjected to a 12:12-h daylight/darkness. Rats were housed in standard cages at room temperature of 25 ± 2°C and left to acclimatize for a few days before starting the experimental procedures.

### 2.2 Ethical Approval

The experimental design and all animal procedures were carried out in accordance with the U.K. Animals (Scientific Procedures) Act, 1986 and associated guidelines, EU Directive 2010/63/EU for animal experiments. This study was conducted in and approved by the Animal House, Physiology-Faculty of Medicine, Cairo University according to the guidelines for the use of experimental animals of Cairo University.

### 2.3 Animal Grouping and the Study Protocol

#### 2.3.1 Animal (in vivo) Study

Thirty-six rats were involved in the *in vivo* study, divided into six groups (6 rats in each). Animals were randomly allocated into; Group 1 (control), as sham-operated rats, the rats in this group were anesthetized and the abdominal wall was opened then the kidneys were exposed. Animals were observed for the same duration equivalent to that of renal ischemia in the other I/R groups but no further surgical manipulations were done. A well-moistened gauze was applied over the abdominal viscera, thus keeping organs moist; Group 2 (I/R), as a group of bilateral renal I/R, rats in the group were subjected to bilateral renal ischemia for 45 min and reperfusion for 7 days; Group 3 (I/R-NC), a group of bilateral renal I/R treated with systemic nicorandil 30 min before and 24 h after the operation (3 mg·kg-1 I.P) (Shimizu et al., 2011); Group 4 (I/R-MSCs), bilateral renal I/R group treated with BM-MSCs; Group 5 (I/R-MSCC), bilateral renal I/R group treated with BM-MSCs preconditioned with nicorandil; Group 6 (I/R-NC-MSCC), presenting bilateral renal I/R group that was treated with a combined systemic nicorandil and conditioned BM-MSCC. Both conditioned and non-conditioned BM-MSCs were given once at a dose of 1.5 × 10^6^ cells for each rat (Erpicum et al., 2017) and injected to rat tail veins immediately after reperfusion. The total duration of the experiment was 7 days and early at the eighth-day blood samples were collected from the animals and all were sacrificed using a high dose of phenobarbital (800 mg/kg IP) (Zatroch et al., 2017).

#### 2.3.2 Surgical Induction of Bilateral Renal I/R in Rats

Rats were anesthetized with ketamine (50 mg/kg) and xylazine (5 mg/kg) that were injected intraperitoneal (Struck et al., 2011). The animals were fixed in the supine position on a thermoregulated heating board to maintain their body temperature at 37°C. After hair shaving, the skin of the abdominal wall was sterilized. A midline abdominal incision was done to open all layers of the anterior abdominal wall and fascia, thus allowing exposure of kidneys. Then, renal pedicles on both sides were exposed and clamped using a vascular clamp to induce renal ischemia. After 45 min, the vascular clamps were removed and the abdominal cavity was closed with double sutures (for peritoneum and facia) (Hussein et al., 2014).

Blood samples were collected at the end of the study; serum was separated after centrifugation and stored at -80°C for biochemical analysis. Pancreatic tissues were collected (extracted) and prepared for histological examinations, immunohistochemical analysis of CD31, and biochemical measurement of apoptotic markers.

### 2.4 Chemicals

- Nicorandil: obtained from Sigma-Aldrich was dissolved in saline (0.9% NaCl and pH 7.4)
- Ketamine HCl obtained from the pharmacy: a product of Sigma-Tec (Egypt) in the form of vials, each one containing 50 mg/ml.
- Xylazine HCl obtained from the veterinary pharmacy: a product of Kepro veterinary solutions (Holland) in the form of vials, each one containing 20 mg/ml.
- Phenobarbital obtained from the veterinary pharmacy was given for euthanasia once at the end of the study at a dose of 800 mg/kg

### 2.5 The *in vitro* Study

#### 2.5.1 Isolation, Preparation, Identification, Culturing, and Conditioning of BM-MSCs

Six male Wistar rats were sacrificed and femur bones were dissected and used for extraction of bone marrow samples. The bone marrow samples were obtained by flushing the bone marrow cavity with phosphate-buffered saline [PBS, Sigma Chemical Co. (United States)]. A preparation of 15 ml from flushed bone marrow cells was carefully layered for 35 min on 15 ml Ficoll-Paque (Gibco-Invitrogen, Grand Island, NY). Then the preparation was centrifuged at 400×g rpm. The mononuclear layer of cells was carefully aspirated and washed twice in PBS containing 2 mM Ethylene diamine tetra-acetic acid (EDTA) and centrifuged for 10 min at 200×g rpm at 5°C.

**FIGURE 1:**
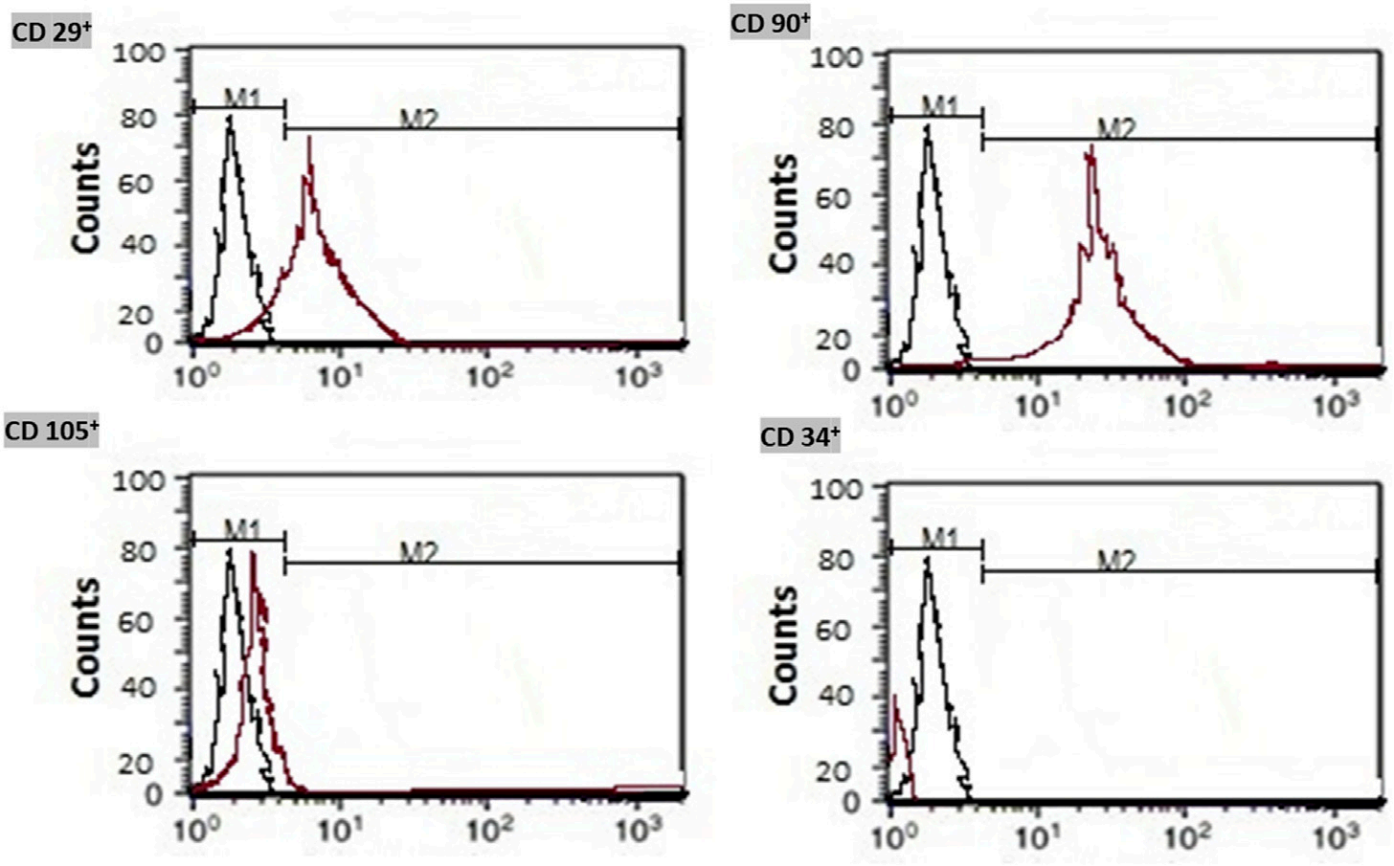
Immunophenotyping of the isolated bone marrow mesenchymal stem cells. Isolated MSCs were identified by flow cytometry for detection of CD 29^+^, CD 90^+^, and CD 105^+^ cell surface proteins and negativity of CD 34^+^.

On 25 ml flasks, the isolated bone marrow MSCs were expanded. Cells were cultured in Roswell Park Memorial Institute (RPMI)-1640 medium containing 10% fetal bovine serum (FBS product of Sigma, United States), 0.5% penicillin, and streptomycin. The prepared flasks were incubated at 37°C and 5% CO2 until reaching 80%–90% allowing their confluence within 7 days. Expansion of MSCs was done till reaching the third passage. MSCs were identified by morphology and Fluorescent Analysis Cell Sorting technique (FACS) to be CD 29^+^, CD 90^+^, and CD 105^+^ positive and CD 34^+^ negative before preconditioning. (Figure 1).

#### 2.5.2 Preconditioning of MSCs With Nicorandil

Obtained mesenchymal stem cells were divided into non-conditioned (MSCs) and nicorandil-conditioned (MSCs-Conditioned) stem cells.

The conditioned MSCs were incubated with nicorandil (100 µM) for a time period of 90 min. This time was selected according to Zhang and colleagues’ study (2015).

#### 2.5.3 Cell Proliferation Assay of MSCs

The proliferation of both non-conditioned and nicorandil-conditioned stem cells was determined using the 3-(4,5-dimethylthiazol-2-yl) −2.5-diphenyltetrazolium bromide (MTT) cell proliferation kit (Trevigen Inc., Gaithersburg, MD). All procedures were done according to the manufacturer’s instructions. After culturing the cells and overnight incubation to permit cell attachment, an MTT reagent was added to allow the reduction of the soluble intracellular MTT to an insoluble purple formazan dye. Viable cells with active metabolism convert MTT into a purple-colored formazan product. The formazan is then solubilized prior to recording absorbance. Absorbance was measured at 550–600 nm using a plate reading spectrophotometer to evaluate cell proliferation.

#### 2.5.4 Labeling of BM-MSCs

All MSCs were washed with DMEM then were labeled with PKH 26 Red Fluorescent Cell Linker Kit for General Cell Membrane Labeling (Sigma-Aldrich) and injected into the rat tail vein immediately after reperfusion. A fresh solution of PKH 26 ethanolic dye was prepared that contained a final concentration of 2 × 10^–6^ PKH 26. A mix of 2 μl of working dye and 1 × 10^7^ cell/ml were centrifuged then dilution was done according to manufacturer instructions. For detection of stem cells in the pancreas, after sacrifice pancreatic paraffin blocks were prepared and MSCs were detected under Leica immunofluorescence microscopy.

Transplanted stem cells (either non-conditioned or pre-conditioned) were directly injected into rat tail veins of assigned groups at a dose of 1.5 × 10^6^ cells for each rat (Erpicum et al., 2017).

### 2.6 Biochemical Assessments

Measurements of serum urea and creatinine were carried out by colorimetry using reagent kits (Urea Colorimetric Assay Kit Cat. No. E-BC-K329-S Houston, Texas) & Creatinine Assay Kit (Colorimetric) (ab204537) Waltham, United States) to assess renal function.

#### 2.6.1 Estimation of Fasting Blood Glucose

Fasting serum glucose was measured by colorimetry using (Glucose Colorimetric Assay Kit (ab282922), Waltham, United States) kits.

#### 2.6.2 The Pancreatic Function was Evaluated by Measuring Serum Concentrations of Amylase, LDH, and MPO

Serum amylase was measured using Colorimetric assay kits obtained from Abcam (ab102523) following the manufacturer’s instructions. Lactate dehydrogenase Enzyme (LDH) Activity Assay: Tissue damage following ischemia-reperfusion was assessed using serum LDH activity. According to kit instruction [LDH Assay Kit/Lactate Dehydrogenase Assay Kit (Colorimetric) (ab102526)]. Determination of myeloperoxidase (MPO) activity: Evidence of inflammation and pancreatic neutrophil infiltration were detected by quantifying tissue MPO activity. According to kit instruction; MPO Activity Assay Kit (Colorimetric) (ab105136).

#### 2.6.3 Estimation of Pancreatic TNF-αAnd c-GMP Levels

Determination of TNF- α concentrations in pancreatic tissues by the sandwich Enzyme-Linked Immuno-Sorbent Assay (ELISA) kits according to the manufacturer’s instruction; TNF-alpha ELISA Kit, RAB1089-1 KT (Sigma-Aldrich Chemie GmbH Eschenstrasse 5 D-82024 TAUFKIRCHEN).

After tissue homogenization, the formed component was mixed with PBS, the formed homogenate was centrifugated and the supernatant was collected. Then using quantitative Sandwich Rat Cyclic Guanosine Monophosphate (c-GMP) ELISA Kit (MyBioSource, Catalog #MBS007871, the level of c-GMP was estimated and expressed per ml.

#### 2.6.4 Western Blotting Analysis of PI3K, Akt, and mTOR in Pancreatic Tissues and BM-MSCs

Phosphorylated PI3K/Akt/mTOR were detected by western blot analysis (Mahmood and Yang. 2012) using anti-phospho-PI3K (Tyr458, 1:1000; Cell Signaling), anti-phospho-AKT (Ser473, 1:2000; Cell Signaling) (#4060), anti-phospho-mTOR (Ser2448, 1:1000; Cell Signaling) antibodies (#2971) and were normalized to β-actin antibodies (Cell signaling technology), Proteins from pancreatic tissues and MSCs pellets were extracted by RIPA lysis buffer which was provided by Bio Basic Inc. (Markham, Ontario L3R 8T4, Canada). 20 μg protein were separated by SDS/PAGE on 4%–20% polyacrylamide gradient gels, then added in each lane. After incubation in 5% non-fat dry milk, Tris/HCl, 0.1% Tween 20 for 1 h; collagen-I monoclonal antibody was added to one of the membranes containing specimen samples and incubated at 4°C overnight. The blots were incubated with the peroxidase-conjugated secondary antibody (Novus Biologicals) solution for 2 h at room temperature. After being washed twice with 1× TBST, densitometric analyses of the immunoblots were performed to quantitate the amount of PI3K, Akt, and mTOR against the control sample by total protein normalization using image analysis software on the ChemiDocMP imaging system (version 3) produced by Bio-Rad (Hercules, CA).

#### 2.6.5 Quantitative Real-Time Polymerase Chain Reaction of Bax and Bcl2 Gene Expression in Pancreatic Tissues and BM-MSCs

The pancreatic tissues and MSCs of all the studied groups were lysed and total RNA was isolated with Gene JET Kit (Thermo Fisher Scientific Inc., Germany, #K0732). The one-step qRT-PCR reaction was done; for reverse transcription; about 5 µl from the total RNA from each sample was used with subsequent amplification with Bioline, amedian life science company, U.K. (SensiFASTTM SYBR R Hi-ROX) One-step Kit (catalog number PI-50217 V) in a 48-well plate using the Step-one instrument (Applied Biosystems, United States of America). The thermal profile was as follows: 45°C for 15min in one cycle (for cDNA synthesis), 10 min at 95°C for reverse transcriptase enzyme inactivation, followed by 40 cycles of PCR amplification. 10 s at 95°C, 30 s at 60°C, and 30 s at 72°C. were adjusted for each cycle. The expression levels of studied genes were normalized relative to the mean critical threshold (CT) values of ß actin as the housekeeping gene by the Ct method. The qPCR assay was done with primers specific for Bax (forward: 5′- GTT TCA TCC AGG ATC GAG CAG-3′; and reverse: 5′- CAT CTT CTT CCA GAT GGT GA-3′, for Bcl2 (forward: 5′- CCT GTG GAT GAC TGA GTA CC-3′ and reverse: 5′- GAG ACA GCC AGG AGA AAT CA-3′ and the housekeeping gene ß-actin (forward: 5′- GCA CCA CAC CTT CTA CAA TG-3′ and reverse: 5′- TGC TTG CTG ATC CAC ATC TG-3′).

### 2.7 Histopathological Examination and Immunohistochemical Evaluation

Pancreatic tissues were carefully-dissected and kept in 10% formol saline solution. After routine histological preparation and paraffin embedding, 3–5 μm sections were stained with hematoxylin and eosin (H&E) and were subjected to immunohistochemical evaluation. Histological assessment of the pancreas was performed in six non-over-lapping fields as previously described (Yang et al., 2020). Grading of the pancreas was evaluated based on a 0 to 4 score of edema, inflammatory cellular infiltration, and acinar necrosis where 0 indicates no lesion, 1 indicates focal edema, ductal inflammation, or peri-ductal necrosis, 2 is given to diffuse edema, <20% of the lobules with inflammation or focal parenchymal necrosis, 3 is scored for acinar disruption, 20%–50% of the lobules with inflammation or diffuse parenchymal necrosis, score 4 is given to separated acini, inflammation in >50% of the lobules or diffuse parenchymal necrosis (>50%).

Immunohistochemical staining was performed according to the manufacturer’s instructions using the following antibodies; rabbit polyclonal anti-cleaved caspase-3 antibody (Cat. # ab2302, 1:100), rabbit polyclonal anti-CD31antibody (ab124432, 1:100), rabbit polyclonal anti-insulin antibody (ab63820, 1:100) (IHC-P, species reactivity including rats, Abcam R, Cambridge, United States). The 3 μm sections were deparaffinized, rehydrated, and subjected to microwave mediated antigen retrieval, quenching of endogenous peroxidase activity, and staining with the avidin-biotin method as previously described (Wanas et al., 2022). The sections were counterstained with Meyer’s hematoxylin for nuclear visualization. Negative controls were prepared using the same procedure after the omission of the primary antibodies.

#### 2.7.1 **Histo-**Morphometric Study

The area percentage of insulin immune-reaction in the pancreatic islets, the count of CD31 positive micro-capillaries, the area percentage of caspase 3 immunohistochemical expression, and the count of PKH26-labeled MSCs, were all performed in six non-overlapping fields under magnification 400 inside a standard measuring frame of 85,550 mm^2^ using Leica Qwin-500 LTD-software image analysis computer system (Cambridge, England) and ImageJ analysis computer system (J Image Pro Plus6.0, Media Cybernetics, Silver Spring, MD, United States).

### 2.8 Statistical Analysis

Data were expressed as mean ± SD and analyzed using SPSS computer software version 22. For *in vivo* study; comparisons between groups were done using ANOVA and Bonferroni post hoc tests for multiple comparisons. Paired sample *t*-test was done to compare between MSCs groups in the *in vitro* study. *p*-value ≤ 0.05 was considered significant.

## 3 Results

### 3.1 Establishment of Acute Renal Injury Following Bilateral I/R

A significant increase in serum urea and creatinine levels was observed in I/R group compared to the sham control group (*p*-value <0.001). The level of urea and creatinine showed a significant decrease in all treated groups compared to the I/R group (*p*-value <0.001).

The results showed no significant difference in urea and creatinine levels between I/R-NC, I/R-MSCs, and I/R-MSCC groups (*p*-value >0.05), while the combined systemic intake of NC and MSC conditioned with NC resulted in a significant decrease in urea and creatinine compared to I/R-NC, I/R-MSCs and I/R-MSCC groups (*p*-value <0.001). (Table 1).

**TABLE 1:** Serum urea and creatinine levels in all studied groups (*n* = 6 in each group). *p*-value ≤0.05 is considered significant.

	Control	I/R	I/R –NC	I/R-MSCs	I/R-MSCC	I/R-NC-MSCC
Urea mg/dL	38.25 ± 1.43	108.57 ± 11.01^a^	72.55 ± 6.08^a^ ^,^ ^b^	62.6 ± 5.45^a^ ^,^ ^b^	64.48 ± 10.73^a^ ^,^ ^b^	41.6 ± 5.44^b^ ^,^ ^c^ ^,^ ^d^ ^,^ ^e^
Creatinine mg/dL	0.14 ± 0.05	1.96 ± 0.08^a^	0.6 ± 0.03^a^ ^,^ ^b^	0.72 ± 0.09^a^ ^,^ ^b^	0.68 ± 0.06^a^ ^,^ ^b^	0.18 ± 0.09^b^ ^,^ ^c^ ^,^ ^d^ ^,^ ^e^

a ^Denotes significant difference versus the control group.^

b ^Denotes significant difference versus I/R group.^

c ^Denotes significant difference versus I/R -NC group.^

d ^Denotes significant difference versus I/R-MSCs group.^

e ^Denotes significant difference versus I/R-MSCC group.^

### 3.2 Determination of Pancreatic Functions Following Bilateral I/R

Bilateral I/R injury resulted in pancreatic damage that was detected by a significant increase in fasting glucose level in rats subjected to I/R injury compared to control (*p*-value <0.001) (Table 2). Whereas, a significant decrease in glucose level was documented in all treated groups compared to the I/R group (*p*-value <0.001). A significant decrease in glucose level was observed in I/R-MSCC, and I/R-NC-MSCC compared to both I/R -NC, and I/R-MSCs (*p*-value 0.019, <0.001) (0.032, <0.001 respectively). Moreover, the combined systemic NC and MSC-conditioned stem cells induced a significant decrease in glucose level in I/R-NC-MSCC compared to I/R-MSCC, (*p*-value <0.001) (Table 2).

**TABLE 2:** Estimated levels of blood glucose, LDH, amylase, MPO, and TNF-α in all studied groups (*n* = 6 in each group). *p*-value ≤0.05 is considered significant.

	Control	I/R	I/R-NC	I/R-MSCs	I/R-MSCC	I/R-NC-MSCC
Glucose mmol/L	4.26 ± 0.38	11.4 ± 0.82^a^	9.33 ± 0.34^a^ ^,^ ^b^	9.26 ± 0.48^a^ ^,^ ^b^	8.33 ± 0.44^a^ ^,^ ^b^ ^,^ ^c^ ^,^ ^d^	5.5 ± 0.36^a^ ^,^ ^b^ ^,^ ^c^ ^,^ ^d^ ^,^ ^e^
LDH U/L	115.05 ± 10.71	247.5 ± 23.92^a^	156.77 ± 8.86^a^ ^,^ ^b^	179.98 ± 20.18^a^ ^,^ ^b^	165 ± 20.48^a^ ^,^ ^b^	137.13 ± 6.56^b^ ^,^ ^d^
Amylase U/L	305.35 ± 25.67	574.13 ± 36.23^a^	400.97 ± 30.24^a^ ^,^ ^b^	379.95 ± 36.39^a^ ^,^ ^b^	352.98 ± 38.49^b^	317.68 ± 20.23^b^ ^,^ ^c^ ^,^ ^d^
MPO U/L	117.55 ± 9.6	320.1 ± 10.24^a^	204.32 ± 9.78^a^ ^,^ ^b^	192.93 ± 17.15^a^ ^,^ ^b^	187.88 ± 15.5^a^ ^,^ ^b^	146.72 ± 16.69^a^ ^,^ ^b^ ^,^ ^c^ ^,^ ^d^ ^,^ ^e^
TNF-α Pg/mg Protein	15.48 ± 0.82	107.88 ± 3.92^a^	69.85 ± 5.57^a^ ^,^ ^b^	58.42 ± 3.69^a^ ^,^ ^b^ ^,^ ^c^	44.48 ± 3.07^a^ ^,^ ^b^ ^,^ ^c^ ^,^ ^d^	28.33 ± 0.96^a^ ^,^ ^b^ ^,^ ^c^ ^,^ ^d^ ^,^ ^e^
c-GMP pmol/ml	15 ± 0.92	4.06 ± 0.40^a^	10.92 ± 1.26^a^ ^,^ ^b^	5.24 ± 0.60^a^ ^,^ ^c^	5.63 ± 0.78^a^ ^,^ ^b^ ^,^ ^c^	12.97 ± 0.33^a^ ^,^ ^b^ ^,^ ^c^ ^,^ ^d^ ^,^ ^e^

a ^Denotes significant difference versus the control group.^

b ^Denotes significant difference versus I/R group.^

c ^Denotes significant difference versus I/R -NC group.^

d ^Denotes significant difference versus I/R-MSCs group.^

e ^Denotes significant difference versus I/R-MSCC group.^

The remote effect of I/R injury was also detected by the significant increase in LDH, amylase, and MPO (markers for pancreatic injury) in the I/R group compared to the control sham-operated group (*p*-value <0.001). In addition, all treated groups showed a significant decrease in LDH, amylase, and MPO levels compared to the I/R group (*p*-value <0.001), however, the level of LDH was still significantly higher in all treated groups except I/R-NC-MSCC compared to the control sham-operated group (*p*-value <0.001 for all and 0.219 for I/R-NC-MSCC), and no significant difference observed in amylase levels between I/R-NC-MSCC, I/R-MSCC, and control group (*p*-value >0.05). The combined treatment with systemic NC and MSCC produced a significant decrease in MPO compared to I/R-NC, I/R-MSCs, I/R-MSCC (*p*-value <0.001) (Table 2). Ischemic/reperfusion injury was associated with significant decrease in c-GMP level compared to control group. Whereas, nicorandil treatment yielded significant increase in tissue cGMP levels compared to I/R and I/R-MSC and I/R-MSCs.

### 3.3 Amelioration of Pancreatic TNF-Α Production in all I/R Treated Groups

Bilateral renal I/R injury enhanced the production of pancreatic TNF-α compared to the control group (*p*-value <0.001). However, in all treated groups, our results showed a significant decrease in TNF-α that was observed compared to the I/R group (*p*-value <0.001). The combined intake of nicorandil and MSCs that were conditioned with NC successfully decreased the tissue level of TNF-α, indeed the level of this marker was significantly decreased in I/R-NC-MSCC (*p*-value <0.001) compared to I/R-NC, I/R-MSCs, and I/R-MSCC groups (Table 2).

### 3.4 Systemic NC and MSC Pre-Conditioning Maximize the Activation of the PI3K/Akt/Mtor Signaling Pathway

**FIGURE 2:**
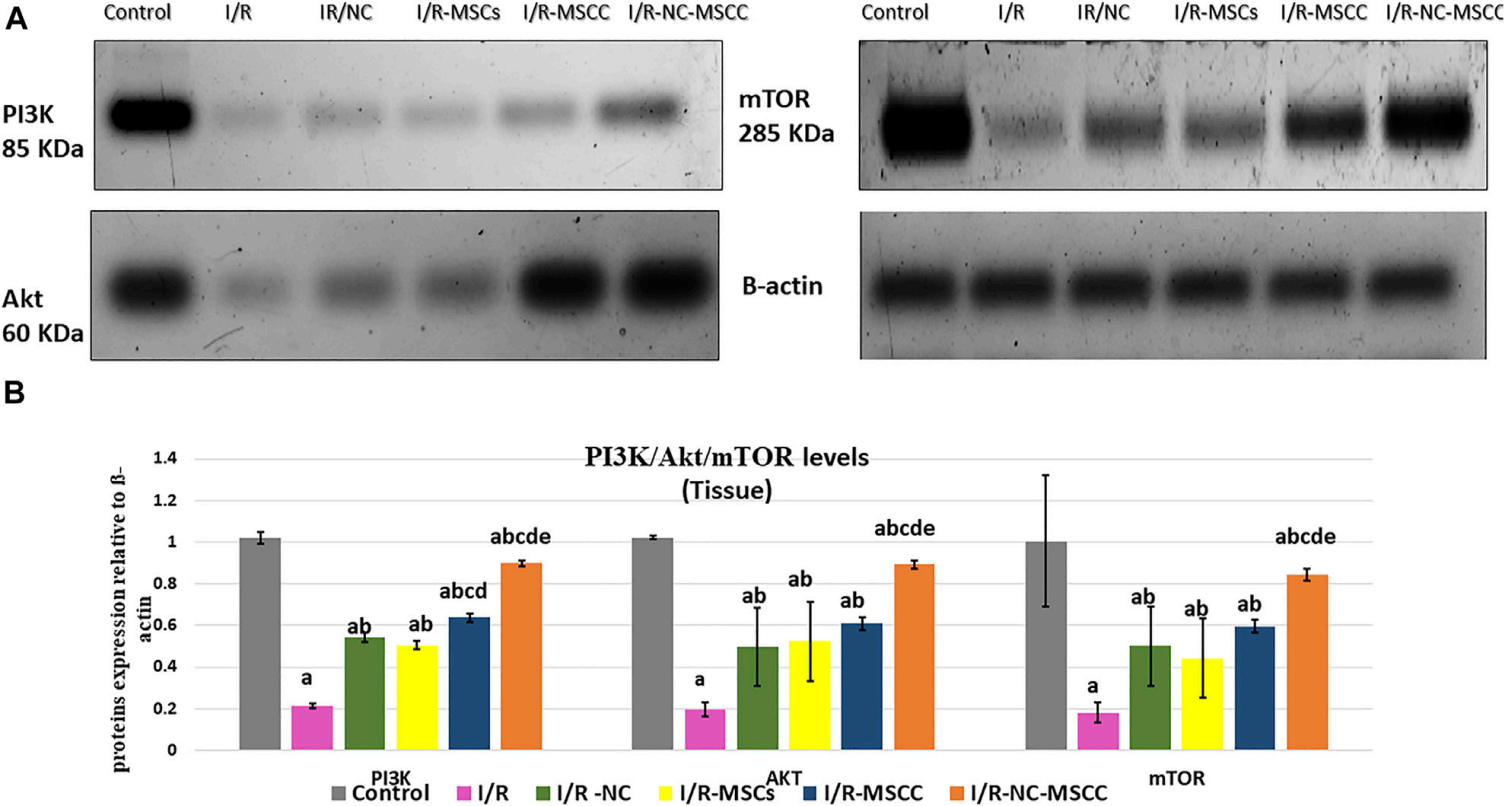
Western blot analysis of PI3K/Akt/mTOR in pancreatic tissues of the *in vivo* study. **(A)** Protein bands and **(B)** Quantitative measurement of mean and SD of all data. a: significant versus control group, b: significant versus I/R group, c: significant versus I/R -NC group, d: significant versus I/R-MSCs group, and e: denotes significant difference versus I/R-MSCC group at *p*-value ≤ 0.05 using ANOVA, Bonferroni post hoc test, *n* = 6).

Activation of the PI3K/Akt pathway supports and promotes cell survival and proliferation. Currently, the results showed a significant decrease in PI3K/Akt/mTOR expression in the I/R group compared to the control sham-operated group (*p*-value <0.001), while a significant increase in their expression levels was detected in all treated groups compared to the I/R group (*p*-value <0.05). The intake of NC systemically together with MSCC in the I/R-NC-MSCC group caused a significant increase in immunoblotting of PI3K/Akt levels compared to I/R–NC (*p*-value <0.001), I/R-MSCs group (*p*-value <0.001), and I/R-MSCC (*p*-value <0.001). mTOR is downstream signaling of PI3K/Akt was estimated also. Our results showed a significant increase in immunoblotting of mTOR in I/R-NC-MSCC group compared to I/R–NC (*p*-value = 0.004), I/R-MSCs group (*p*-value = 0.004), and I/R-MSCC (*p*-value = 0.047) (Figure 2).

### 3.5 Combined Systemic NC and MSC (Either Pre-Conditioned or Not) Exerted Anti-Apoptosis and Significantly Augmented Pancreatic Protection

Bcl2 gives rise to cellular protection *via* exerting anti-apoptosis, it could bind to Bax preventing the pore formation and subsequent molecular changes that end in cell death. For instance, the expression level of Bax was significantly increased (*p*-value <0.001) in the I/R group compared to the control sham-operated one. The results showed a significant decrease in Bax in all treated groups compared to the I/R group (*p*-value <0.001), however, the maximum inhibition of Bax, the death-promoting factor, was observed in combined systemic administration of NC and pre-conditioned MSCs. In this context, the level of Bax was significantly decreased in the I/R-NC-MSCC group compared to I/R-NC, I/R-MSCs, and I/R-MSCC groups (*p*-value <0.001).

Compared to Bax, the expressions levels of Bcl2 were significantly decreased in the I/R group compared to the control group (*p*-value <0.001). Over-expression of Bcl-2 enhances cell survival by suppressing apoptosis, thus our results showed a significant increase in Bcl2 in all treated groups compared to the I/R group (*p*-value <0.001). There was also a significant rise in I/R-MSCC, I/R-NC-MSCC groups compared to the I/R-MSCs group (*p*-value <0.001). Accordingly, pre-conditioning of MSCs augmented their anti-apoptotic effects (Table 3).

**TABLE 3:** Changes in Bax and Bcl2 in all studied groups (*n* = 6 in each group). *p*-value ≤0.05 is considered significant.

	Control	I/R	I/R -NC	I/R-MSCs	I/R-MSCC	I/R-NC-MSCC
Bax	1.03 ± 0.02	7.5 ± 0.27^a^	4.25 ± 0.26^a^ ^,^ ^b^	3.48 ± 0.29^a^ ^,^ ^b^ ^,^ ^c^	2.65 ± 0.1^a^ ^,^ ^b^ ^,^ ^c^ ^,^ ^d^	1.72 ± 0.09^a^ ^,^ ^b^ ^,^ ^c^ ^,^ ^d^ ^,^ ^e^
Bcl2	1.005 ± 0.038	0.26 ± 0.024^a^	0.41 ± 0.02^a^ ^,^ ^b^	0.55 ± 0.03^a^ ^,^ ^b^ ^,^ ^c^	0.69 ± 0.05^a^ ^,^ ^b^ ^,^ ^c^ ^,^ ^d^	0.84 ± 0.09^a^ ^,^ ^b^ ^,^ ^c^ ^,^ ^d^ ^,^ ^e^

a Denotes significant difference versus the control group.

b Denotes significant difference versus I/R group.

c Denotes significant difference versus I/R-NC group.

d Denotes significant difference versus I/R-MSCs group.

e Denotes significant difference versus I/R-MSCC group.

**FIGURE 3:**
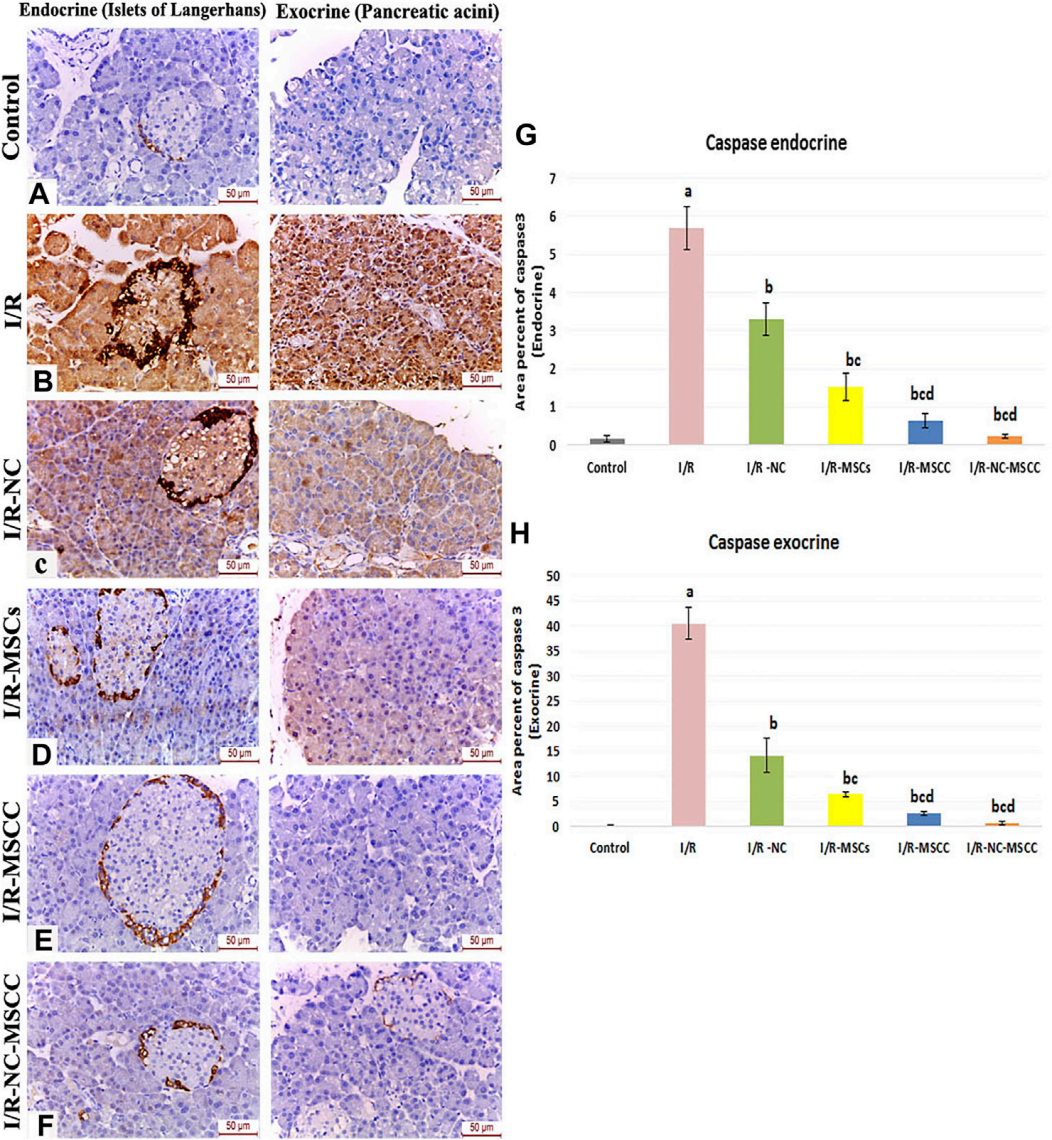
Immunohistochemical staining of caspase 3 in the endocrine and exocrine portion of the pancreatic tissue of **(A)** Control group, **(B)** Ischemia/reperfusion (I/R), **(C)** Ischemia/reperfusion-Nicorandil group (I/R-NC), **(D)** Ischemia/reperfusion-mesenchymal stem cell group (I/R-MSCs), **(E)** Ischemia/reperfusion-conditioned mesenchymal stem cell group (I/R-MSCC), **(F)** Ischemia/reperfusion-conditioned mesenchymal stem cell + nicorandil group (I/R-NC-MSCC) displaying strong positive caspase 3 expression in both the pancreatic islets and acini of the I/R group, moderate immunoreactivity in the I/R-NC group, mild expression in the I/R-MSCs and I/R-MSCC groups and minimal immunoreactivity in the I/R-NC-MSCC group. **(G,H)** Area percentage of caspase 3 expression in the endocrine **(G)** and exocrine **(H)** portion of the pancreas in the different study groups a: significant versus control group, b: significant versus I/R group, c: significant versus I/R -NC group, d: significant versus I/R-MSCs group and e: denotes significant difference versus I/R-MSCC group at *p*-value ≤ 0.05 using ANOVA, Bonferroni post hoc test, *n* = 6).

Comparable results have been obtained from the immunohistochemistry of caspase 3 (the effector of apoptosis) where the area percent of caspase 3 immunohistochemical expression showed a significant increase in the I/R group (*p*-value <0.001), and a significant decrease in all the treated groups with best results obtained in the combined NC-MSCC group. A noticeable result was the statistically-significant difference between the MSCs group and the conditioned MSC on one side and the combined NC-MSCC group on the other side, which further confirms the role of nicorandil in augmenting the anti-apoptotic effect of MSCs (Figure 3).

### 3.6 The Results of Mscs, Incubation With Nicorandil

#### 3.6.1 Proliferation Assay

Using MTT%, the proliferation of both non-conditioned and mesenchymal stem cells-conditioned with NC was determined. Our results showed increased MTT% of MSC-conditioned that was statistically significant to MSCs (P-value <0.001) (Table 4).

**TABLE 4:** Enhanced cell proliferation assay (MTT%) and decreased apoptotic markers of MSC incubated with nicorandil. *p*-value ≤0.05 is considered significant.

	MSCs	MSC-Conditioned
Cell Proliferation (MTT%)	97.2 ± 2.78	140.17 ± 10.7^a^
BAX	1.025 ± 0.018	0.73 ± 0.138^a^
BCL2	1.01 ± 0.012	5.18 ± 1.46^a^

a Denotes significant difference versus MSC non-conditioned as a control group.

#### 3.6.2 Incubation With Nicorandil Decreased Apoptotic Markers and Enhanced Pro-Survival Signaling Pathway of MSCs

**FIGURE 4:**
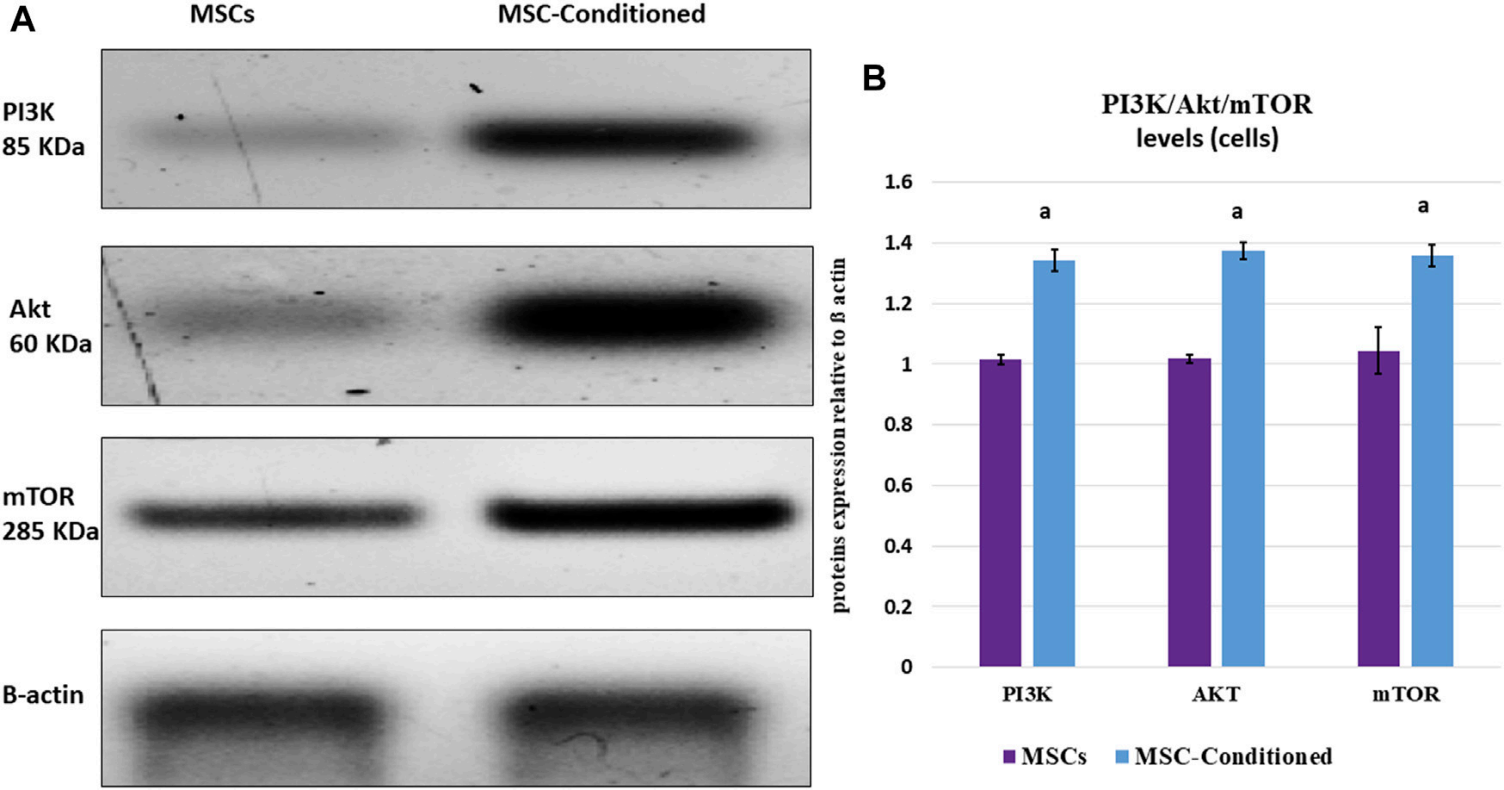
Western blot analysis of PI3K/Akt/mTOR *in vitro* study. Incubation with nicorandil enhanced the pro-survival PI3K/Akt/mTOR signaling pathway of MSCs. **(A)** Protein bands and **(B)** Quantitative measurement of mean and SD of all data. a: significant versus non-conditioned MSCs.

In culture media, the expression levels of Bax were significantly decreased in the MSC-Conditioned group compared to MSCs (*p*-value <0.001), whereas Bcl2 gene expression, as well as the immunoblotting of PI3K/Akt/mTOR, were significantly increased in the MSC-Conditioned group compared to MSCs (*p*-values 0.004, 0.001, <0.001 and <0.001 respectively) (Table 4; Figure 4).

### 3.7 Nicorandil Preconditioning Influences Mesenchymal Stem Cell Count and Improved Survival in Pancreatic Tissues

**FIGURE 5:**
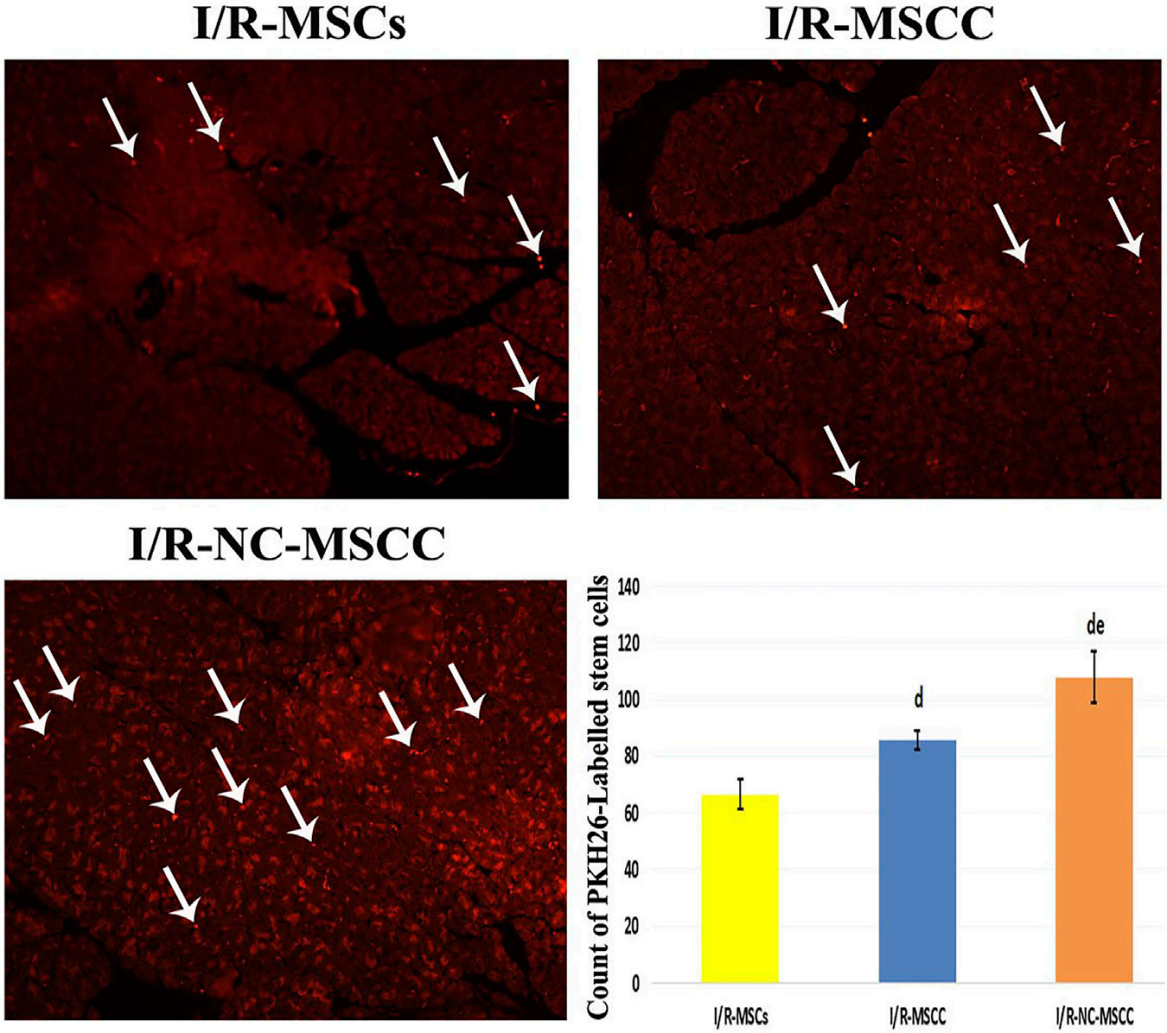
PKH26-labeled immunofluorescent images showing enhanced survival of the MSCs into the pancreatic tissue of the combined systemic nicorandil and nicorandil-conditioned MSC group (I/R-NC-MSCC) relative to the other groups, (I/R-MSCs: Ischemia/reperfusion mesenchymal stem cell group, I/R-MSCC: Ischemia/reperfusion-conditioned mesenchymal stem cell group) (d: significant versus I/R-MSCs group and e: denotes significant difference versus I/R-MSCC group at *p*-value ≤ 0.05 using ANOVA, Bonferroni post hoc test, *n* = 6).

Evaluation of delivered MSCs in pancreatic tissues under the Leica immunofluorescence microscope revealed a statistically-significant increase in the count of PKH26-labeled MSC, in the nicorandil-preconditioned MSC group relative to the non-conditioned MSCs. The combination between systemic nicorandil and nicorandil-preconditioning caused better survival of MSC in the pancreatic tissue with results superior to both the NC-MSCs and the NC-MSCC groups (Figure 5).

### 3.8 Amelioration of Renal I/R-Induced Pancreatic Histopathological Changes by Combined Nicorandil and Nicorandil-Conditioned MSCs

**FIGURE 6:**
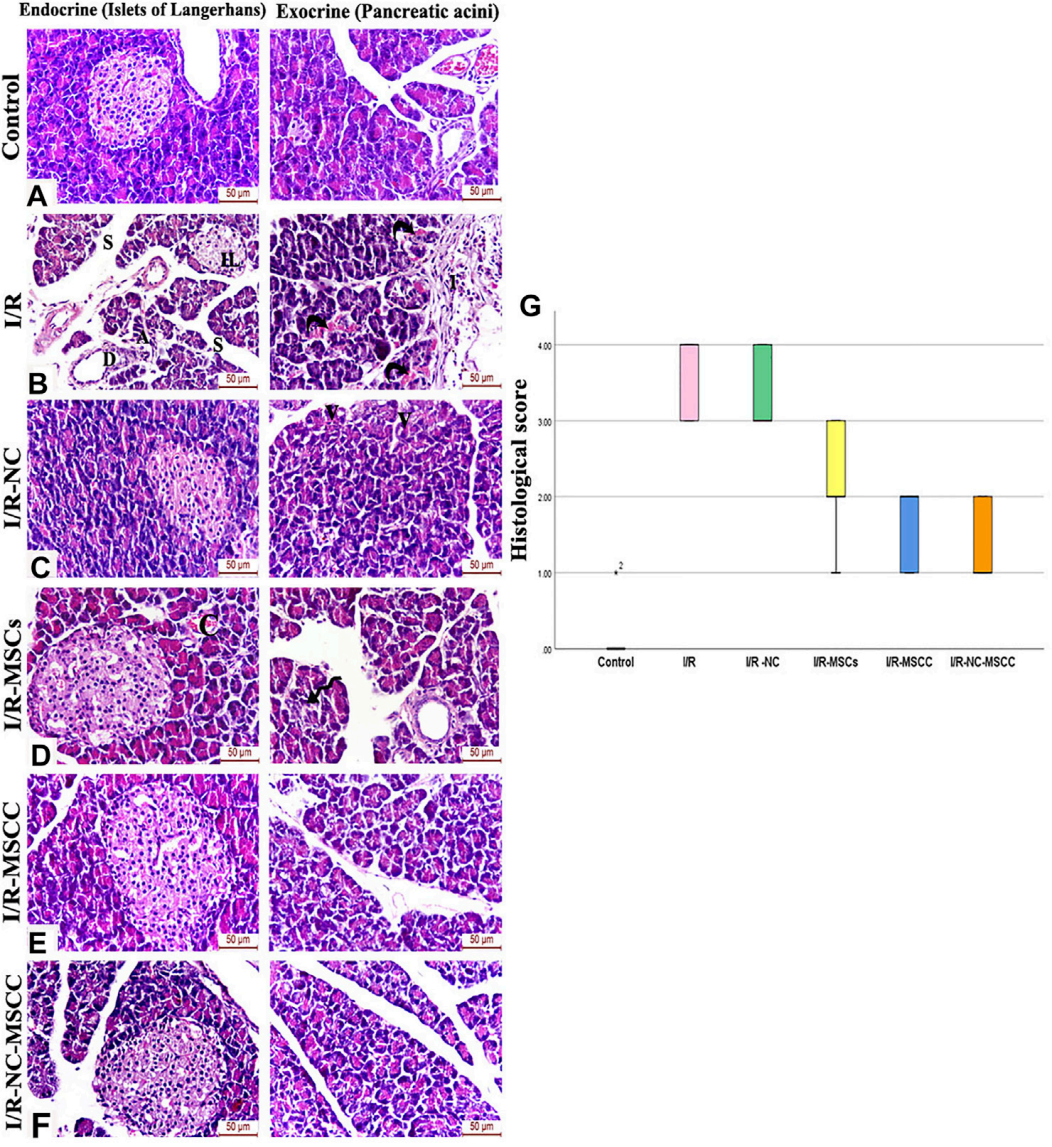
H&E-stained sections of the pancreatic tissue of **(A)** Control group, **(B)** Ischemia/reperfusion (I/R) showing small atrophied islet of Langerhans with a marked reduction in cellularity and pyknotic nuclei (IL). The pancreatic lobules appear shrunken and separated by wide interlobular septae (S). Degenerated acini **(A)**, ductal hyperplasia (D), and vascular angiopathy are also observed. A mononuclear inflammatory cellular infiltration (I) and blood extravasation (curved arrows) are also noticed between pancreatic lobules, **(C)** Ischemia/reperfusion-Nicorandil group (I/R-NC) showing mild irregularity and vacuolations (V) of the pancreatic acini, **(D)** Ischemia/reperfusion-mesenchymal stem cell group (I/R-MSCs) showing improved islet and acinar structure and normal interlobular duct with only mild vascular congestion (C) and focal acinar degeneration (spiral arrow), **(E)** Ischemia/reperfusion-conditioned mesenchymal stem cell group (I/R-MSCC) displaying preserved islet and acinar architecture, **(F)** Ischemia/reperfusion-conditioned mesenchymal stem cell + nicorandil group (I/R-NC-MSCC) illustrating intact cells of islets of Langerhans with dark rounded nuclei and pale acidophilic cytoplasm with blood capillaries in-between. The serous acini show well-defined boundaries, appear lined by pyramidal cells with basal rounded nuclei and apical acidophilic cytoplasm comparable to the control group, **(G)** pathological injury scoring of the different study groups.

Histological scoring of H&E-stained sections was carried out for evaluating the pathological alterations in the different study groups. Renal I/R caused significant pathological changes in both the endocrine and exocrine portion of the pancreas, where the islets of Langerhans appeared atrophied with pyknotic nuclei and exhibited a marked reduction in cellularity. The pancreatic lobules appear shrunken and were seen separated by wide interlobular septae. The pancreatic acini appeared degenerated and were invaded by mononuclear inflammatory cellular infiltration. Treatment with nicorandil and/or MSC caused improvement in pancreatic architecture. This improvement was unsurpassed observed in the combined NC-MSCC group where the islets of Langerhans displayed dark rounded nuclei and pale acidophilic cytoplasm with healthy capillaries in-between and the serous acini showed well-defined boundaries and were seen lined by intact pyramidal cells with basal rounded nuclei and apical acidophilic cytoplasm comparable to the control group (Figure 6).

### 3.9 Improved B-cell function Assessed by Insulin Immunohistochemistry in Response to Nicorandil and Nicorandil-Conditioned MSCs

**FIGURE 7:**
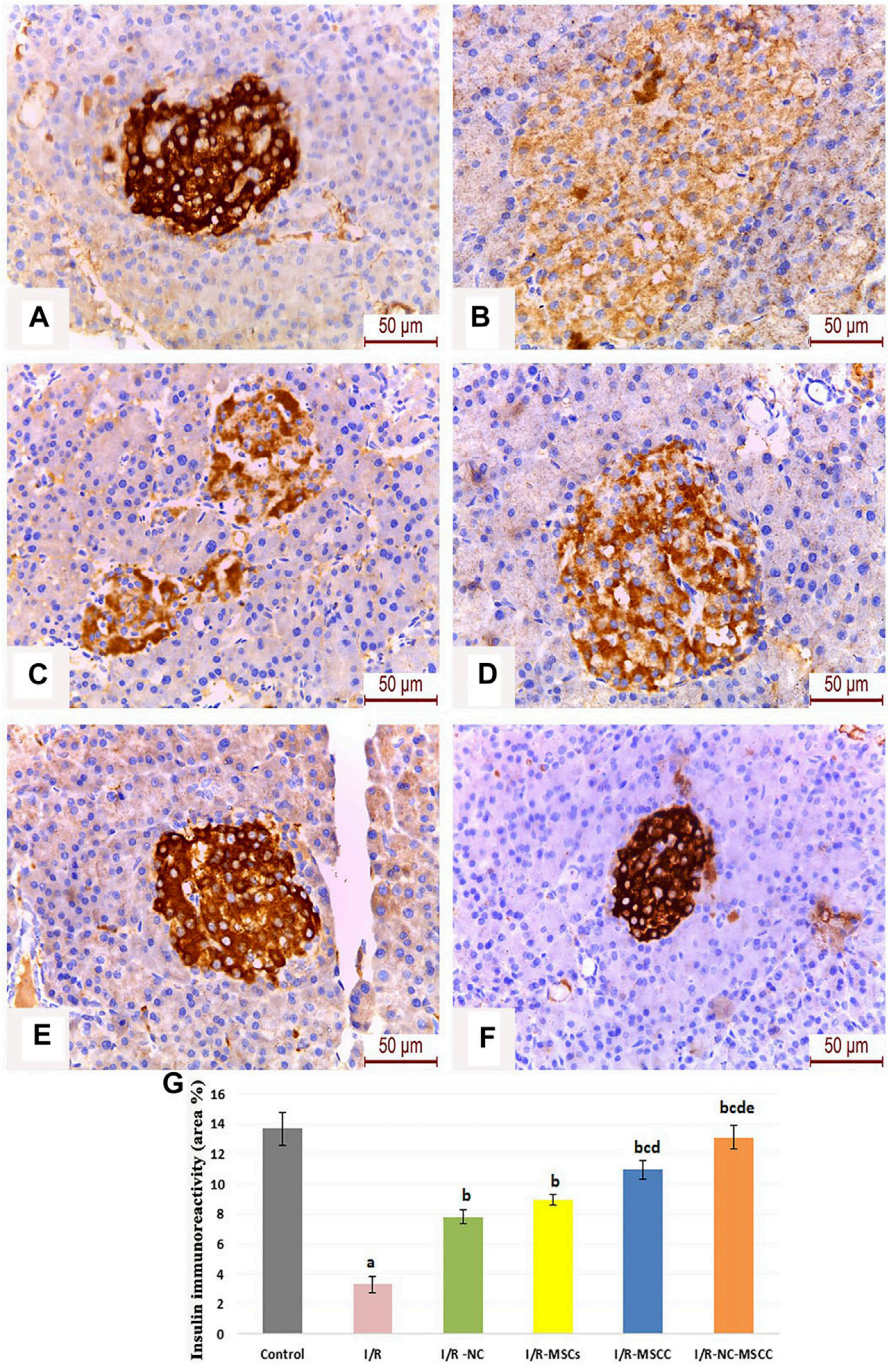
Immunohistochemical staining of insulin in the pancreatic islets of **(A)** Control group, **(B)** Ischemia/reperfusion (I/R), **(C)** Ischemia/reperfusion-Nicorandil group (I/R-NC), **(D)** Ischemia/reperfusion-mesenchymal stem cell group (I/R-MSCs), **(E)** Ischemia/reperfusion-conditioned mesenchymal stem cell group (I/R-MSCC), and **(F)** Ischemia/reperfusion-conditioned mesenchymal stem cell + nicorandil group (I/R-NC-MSCC). Notice: restoration of the pancreatic β-cells insulin immune-stained granules in the treatment groups with Strong positive immunoreactivity in the I/R-NC-MSCC group, **(G)** Area percentage of insulin immune-expression in the different study groups (a: significant versus control group, b: significant versus I/R group, c: significant versus I/R-NC group, d: significant versus I/R-MSCs group, and e: denotes significant difference versus I/R-MSCC group at *p*-value ≤ 0.05 using ANOVA, Bonferroni post hoc test, *n* = 6).

The area percentage of insulin immunohistochemistry showed a significant decrease in the I/R group compared with the control group. The treatment groups showed a significant increase in insulin expression compared to the I/R group (*p*-value <0.001) with the maximum induction observed in the combined systemic NC and nicorandil pre-conditioned MSCs (Figure 7).

### 3.10 MSC Pre-Conditioning and Systemic Nicorandil Causes Activation of Pancreatic Micro-Angiogenesis

Owing to the importance of CD31 as well-defined markers of angiogenesis highly expressed on the surface of endothelial cells, the current study has evaluated the changes in immunohistochemical expression of CD31 in ischemia/reperfusion with and without supplementation of nicorandil and mesenchymal stem cells.

**FIGURE 8:**
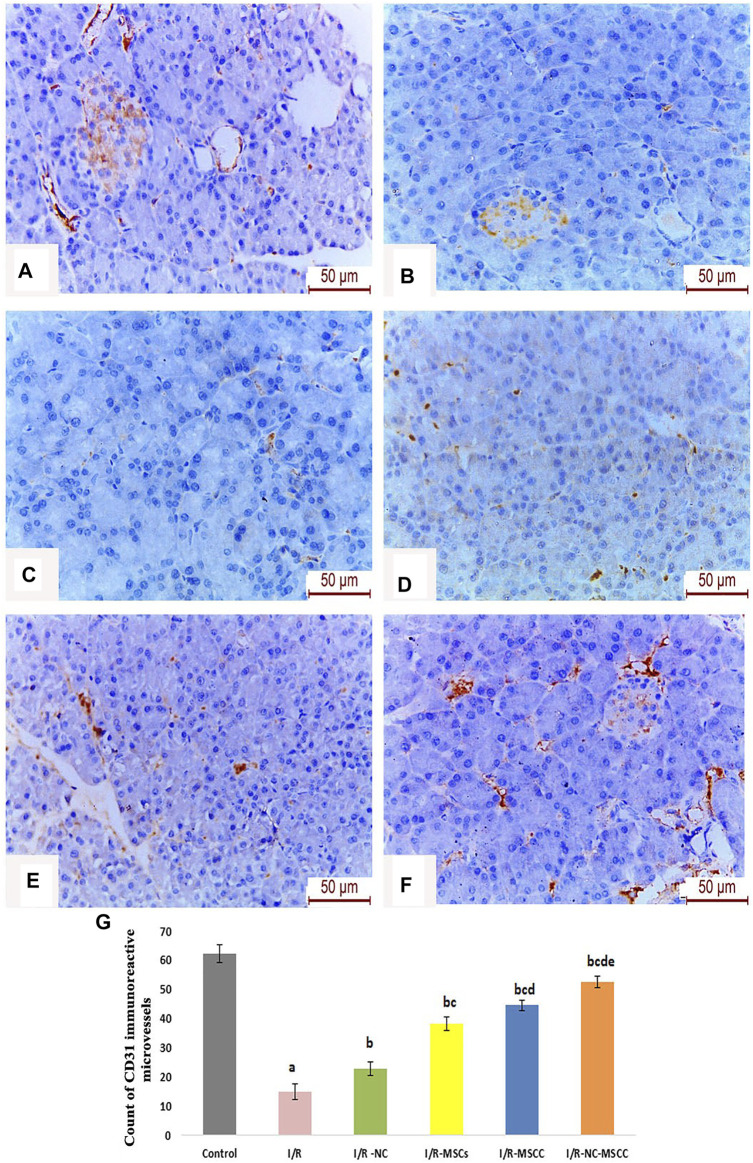
Immunohistochemical staining of CD31 reactive micro-vessels in the pancreas of **(A)** Control group, **(B)** Ischemia/reperfusion (I/R), **(C)** Ischemia/reperfusion-Nicorandil group (I/R-NC), **(D)** Ischemia/reperfusion-mesenchymal stem cell group (I/R-MSCs), **(E)** Ischemia/reperfusion-conditioned mesenchymal stem cell group (I/R-MSCC), **(F)** Ischemia/reperfusion-conditioned mesenchymal stem cell + nicorandil group (I/R-NC-MSCC) demonstrating marked reduction in CD31 immunoreactivity in the I/R group and numerous CD31 positive microcapillaries in both the pancreatic acini and islets of Langerhans in the combined I/R-NC-MSCC group, and **(G)** Count of CD31 immuno-reactive micro-capillaries in the different study groups (a: significant versus control group, b: significant versus I/R group, c: significant versus I/R -NC group, d: significant versus I/R-MSCs group and e: denotes significant difference versus I/R-MSCC group, at *p*-value ≤ 0.05 using ANOVA, Bonferroni post hoc test, *n* = 6).

Our results have revealed a statistically significant decrease in the CD31 immune-histochemical expression in the I/R group. Supplementation with nicorandil and nicorandil-preconditioned mesenchymal stem cells resulted in a significant increase in CD31 immunoreactivity which essentially denotes improvement in angiogenesis and microcirculation (Figure 8).

## 4 Discussion

In the last few decades, many efforts are expended to alleviate the damaging effect of ischemia-reperfusion injury (Frangogiannis. 2015). From this concept, we investigated in this study the effect of opening the K-ATP channel in modulating the deleterious hazards associated with the renal I/R injury in rat pancreas and the underlying molecular mechanism related to the PI3K/Akt/mTOR signaling pathway. To evaluate the role of the channel, we used nicorandil, a well-known K-ATP channel opener. In addition, we hypothesized that engaging several protocols by applying nicorandil, as a pharmacological therapeutic approach as well as a preconditioning therapy for mesenchymal stem cells may be so promising. Our findings revealed activated K-ATP channel may provide new insights in rescuing pancreatic damage following renal I/R injury through its anti-inflammatory, anti-apoptotic and pro-survival effect on stem cells.

It is well known that prolonged periods of renal ischemia over 35 min can result in irreversible acute tubular injury and kidney dysfunction (Holderied et al., 2020). In accordance, our results showed significantly impaired kidney function, manifested by elevated serum urea and creatinine in I/R rats.

The remote effect of renal I/R injury has been considerably investigated in various organs like the liver, heart, brain, and lungs. Here, we reported significant elevation in markers of pancreatic tissue injury (LDH, amylase, and MPO) after 7 days of I/R associated with high serum glucose levels. Such changes indicated that the pancreatic structure and function were significantly damaged after I/R, a finding which is consistent with previous studies (Hussein et al., 2014; Abogresha et al., 2016).

Besides the biochemical findings, these studies are also consistent with our histopathological results, which demonstrated marked deterioration of both the endocrine and exocrine portion of the pancreas in the form of atrophy of islets of Langerhans, shrinkage of pancreatic lobules, acinar necrosis, ductal hyperplasia, vascular angiopathy, blood extravasation, and mononuclear inflammatory cellular infiltration.

It was generally believed that I/R and associated distant organ failure is a complex process with multiple underlying mechanisms (Yuan et al., 2020). The release of inflammatory factors, such as TNF-α has been found to have a crucial role in mediating and exacerbating ischemia/reperfusion injury. In the current study, TNF-α was investigated as a proinflammatory cytokine that can mediate apoptosis via increasing caspase levels. It was significantly elevated in pancreatic tissue in ischemic rats compared to sham-operated rats. Consistent with excess TNF-α, elevated Bax/Bcl2 ratio denoting apoptotic cascades stimulation was significantly observed in injured pancreatic tissues. This agrees with previous works, which demonstrated that I/R was associated with excessive production of ROS leading to acute pancreatitis and apoptosis (Abogresha et al., 2016; Tong et al., 2019). The released TNF-α evoked *in situ* and systemic changes affecting distant organs leading eventually to the recruitment of neutrophils. Neutrophil infiltration to ischemic tissues leads to increased MPO enzymatic activity, that produces massive damage to vascular and parenchymal cells (Kvietys and Granger. 2012; Frangogiannis, 2015). This supports our results which indicates an association between TNF-α level in pancreatic tissues and elevated MPO levels, which goes in line with the correlation studies.

K-ATP channel is a special type of K channels activated by intracellular ATP depletion that occurs during pathological states of hypoxia or ischemia (Szeto et al., 2018). The previous meta-analysis demonstrated its protective effect on the ischemic heart (Zhao et al., 2019), and others proved that activation of K-ATP channels prevents neurodegeneration following cerebral ischemia in rats (Mayanagi et al, 2007; Wang et al., 2011).

Nicorandil has been recognized to have clinical importance as a K-ATP channel activator (Ahmed, 2019). There is growing evidence about its potential therapeutic role against ischemia-reperfusion injury in the heart, in skeletal muscle as well as its role in ischemic stroke (Cahoon et al., 2013; Yang et al., 2015; Sánchez-Duarte et al., 2017; Zhao et al., 2021). Meanwhile, treatment with nicorandil significantly suppresses renal damage after I/R with remarkable improvement in kidney functions.

To date, there have been no reports in the literature exploring the role of nicorandil in pancreatic tissue damage following bilateral renal I/R injury. The cardioprotective effect of nicorandil against ischemic injury has been previously attributed to its anti-inflammatory effects with neutrophil modulation in addition to anti-apoptotic function (Zhao et al., 2014; Li et al., 2015). More specifically, nicorandil could reduce hypoxia-induced apoptosis by activating mitochondrial K-ATP channels, lowering mitochondrial dysfunction and the associated oxidative damage (Ahmed and El-Maraghy. 2013; Afzal et al., 2016).

In the current study, we investigated the protective effect of nicorandil on the pancreas after renal ischemia-reperfusion injury, which to the best of our knowledge is the first time to be investigated. Our results aid further support as nicorandil-treated rats showed a significant reduction in TNF-α expression compared to untreated rats. Additionally, downregulation of Bax and upregulation of Bcl2 expression, were also recorded, indicating the antiapoptotic role of nicorandil. These observations are concordant with the work of Wang and colleagues (Wang et al., 2012) who found that nicorandil injection regulated apoptosis-related proteins before/during ischemia or at the onset of reperfusion. According to previous studies conducted on myocytes, nicorandil can influence cellular proliferation and apoptosis in tissues exposed to hypoxic conditions, these effects are attributed to its ability to activate mitochondrial K_ATP_ channels and cGMP-dependent mechanisms (Nagata et al., 2003; Carreira et al., 2008).

Thus, we measured the level of c-GMP in all studied groups, and we found significant increase in c-GMP in NC-treated groups.

In another study conducted in isolated rat lungs exposed to interrupted (ischemia) for 60 min, and then resumed for 60 min nicorandil pretreatment ameliorated increases in pulmonary microvascular permeability secondary I/R. Moreover, the protective effects of nicorandil against I/R lung injury was blocked by glibenclamide as a K_ATP_ channel blocker and 1H-[1,2,4] oxadiazolo [4,3-a]quinoxaline-1-one (ODQ) as inhibitors of guanylate cyclase (Abe et al., 2020). These protective effects may indicate the involvement of least two mechanisms, K_ATP_ channel opener and the cGMP pathway.

In other treated groups we used MSCs unconditioned and conditioned with nicorandil, in a trial to improve the survival rate of donor MSCs with a multipotential differentiation capacity, that have been widely used in regenerative medicine. *In vivo*, poor blood supply with limited nutrients in the ischemic area is suggested to be the leading cause for the massive MSCs death (Zhu et al., 2006).

Zhang and co-workers (2015) reported that nicorandil may exert protective effects on MSCs under ischemic conditions by activating mitochondrial K-ATP channels. It has been previously reported that nicorandil has a beneficial effect in dilating micro-vessels and improving myocardial perfusion through its effect as a K-ATP channels opener (Horinaka, 2011). In this study, Using MMT assay, MSCs proliferation was significantly increased after incubation with nicorandil.

Consistent with promoted MSCs proliferation, our findings showed significant improvement in renal function tests after MSCs treatment. Notably, combined treatment with systemic nicorandil and conditioned MSCs may induce better results.

The degree of pancreatic tissue injury detected by estimation of LDH, amylase, and MPO levels in all treated rats was significantly lower compared to untreated rats. Preconditioning of MSCs with nicorandil separately or combined with systemic nicorandil decreased both LDH, amylase levels near control levels. This agrees with Zhang and colleagues (2015) who reported pro-survival effects for nicorandil on MSCs under conditions resembling myocardial ischemia. Injection of bone marrow stem cells in a mouse model of remote liver injury, secondary to renal I/R, has been reported to decrease the inflammatory response and apoptotic marker levels, suggesting a remote regenerative and protective effect on injured organs by releasing factors in the bloodstream (El-Tahawy and Ali. 2017).

The beneficial effect was also observed as a preconditioning therapy, as preconditioned MSCs with nicorandil augmented the MSCs antiapoptotic effect, an observation which is consistent with Zhang and his colleague’s study (2015).

Interestingly, maximum protection occurs with combined treatment. These results suggested that nicorandil can suppress the inflammatory and apoptotic response caused by renal I/R injury.

A special focus has been placed in this study on the signaling pathways of nicorandil during pancreatic protection in I/R. Previous reports showed that mTOR-atypical serine/threonine-protein kinase, which belongs to the phosphoinositide 3 kinase (PI3K)-related kinase family-, plays a central role in regulating many essential cellular processes as cell growth, cell survival, and autophagy. Previous reports indicate that mTOR is involved in ischemic injury, and it was rapidly activated and exerts protective effects during the reperfusion phase (Matsui et al., 2007; Wei et al., 2016; Fan et al., 2017). Accumulating evidence indicates that stimulating PI3K/Akt/mTORC1 pathway may suppress apoptosis in brain tissues, causing neuroprotection against IR injury (Yan et al., 2016; Zhang et al., 2016).

The PI3K/Akt signaling pathway is one of the most essential signaling pathways regulating cell response to various stresses, especially anti-apoptosis, and pro-survival responses (Sun et al., 2017; Handa et al., 2019).

In different ischemia-reperfusion injury mouse models applied on hepatic and cardiac tissues, increased levels of p-PI3K, p-Akt have been recorded, indicating that I/R may activate the PI3K/Akt signaling pathway to initiate tissue repair following injury (Zhang et al., 2014; Thokala et al., 2017). Targeting PI3K/Akt signaling pathway by different pharmacological therapies as a potential protective pathway against I/R injury has attracted great attention in many studies (Yapca et al., 2013; Guan et al., 2020; Chen et al., 2021). In the current study, Western blot analysis showed significant upregulation in PI3K/Akt/mTOR expression in pancreatic tissue after nicorandil injection as a single treatment or combined with preconditioned MSCs. In accordance with our results, Wang’s study (2012) verified that nicorandil can alleviate apoptosis in diabetic cardiomyopathy through PI3K/Akt pathway activation. Moreover, in our *in vitro* studies, incubation with nicorandil decreased apoptotic markers and enhanced the pro-survival PI3K/Akt/mTOR signaling pathway of MSCs. Therefore, our results give further support to a previous work suggesting a fundamental role for the PI3K/Akt signaling pathway in the nicorandil-mediated protection of MSCs exposed to ischemic conditions (Zhang et al., 2015).

Limitations of the current study are to be considered and future experiments are recommended. Our key findings are that the pancreatic protective effects of nicorandil in bilateral I/R injury were associated with enhancement of both PI3K/AKT/mTOR and c-GMP in I/R groups treated with NC, which suggests their probable involvement in these effects. In addition, NC could enhance MSCs therapeutic potentials *via* attenuating stem cell apoptosis that was indicated by Bax/Bcl2 levels. Thus, our future experiments should employ knockout models and/or blocker of the PI3K/AKT/mTOR genes with or without 1H-[1,2,4] oxadiazolo [4,3-a] quinoxaline-1-one (ODQ) as an inhibitor of guanylate cyclase, that would more conclusively determine the role of these crucial pathways in bilateral renal I/R-induced pancreatic damage. Additionally, it will provide a more efficient link about the direct involvement of PI3K/AKT/mTOR and/or c-GMP in NC induced pancreatic protection.

**FIGURE 9:**
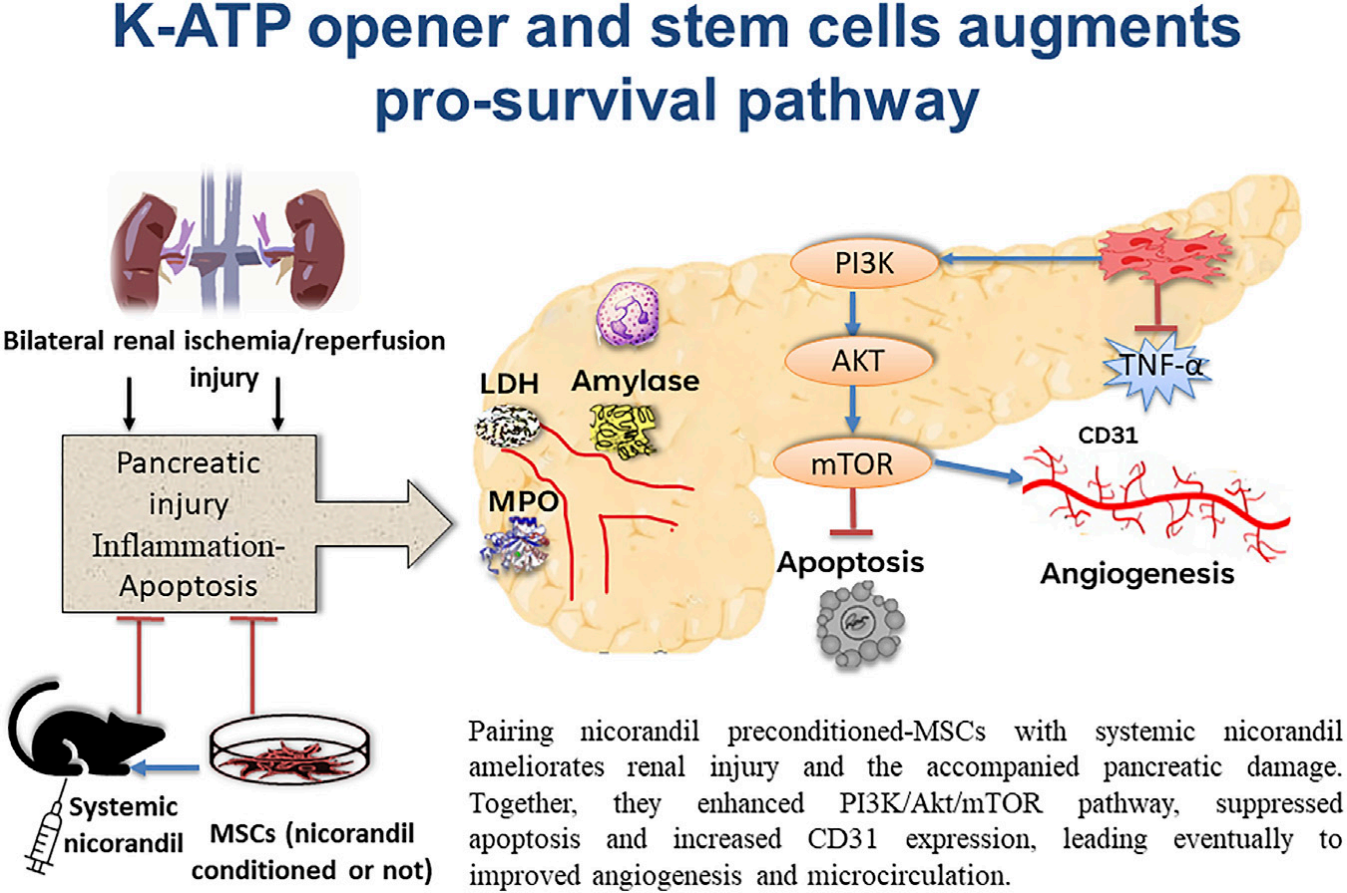
Schematic presentation demonstrating pancreatic protection from the remote effect of renal ischemia/reperfusion injury by engaging several protocols. Applying systemic nicorandil, as a pharmacological therapeutic approach, as well as a preconditioning therapy for mesenchymal stem cells, maximizes this protection.

In conclusion, bilateral renal I/R in rats resulted in pancreatic injury. This study showed that preconditioning MSCs with nicorandil and using combined nicorandil with MSCC ameliorates renal parameters (serum urea and creatinine) and the accompanied pancreatic damage. As it improved blood glucose level, LDH, serum amylase, and MPO. In addition, MSCs enhance PI3K/Akt/mTORC1 pathway, suppress apoptosis, and increase CD31 expression which essentially denotes improvement in angiogenesis and microcirculation (Figure 9). Our *in vitro* study showed that nicorandil enhanced MSCs proliferation, augmented pro-survival signaling pathway, and decreased apoptotic markers which strength our results *in vivo* study. This work supports the view that MSCs pre-conditioning with nicorandil, may introduce a novel treatment in IR injury-induced pancreatic damage which occurs after renal transplantation.

## Data Availability

The original contributions presented in the study are included in the article/Supplementary Materials, further inquiries can be directed to the corresponding author.
