# Supplementary material for: Combined Systemic Intake of K-ATP Opener (Nicorandil) and Mesenchymal Stem Cells Preconditioned With Nicorandil Alleviates Pancreatic Insufficiency in a Model of Bilateral Renal Ischemia/Reperfusion Injury
Source: Front Physiol. 2022 Jun 23;13:934597. doi: 10.3389/fphys.2022.934597 (PMC9260271; doi:10.3389/fphys.2022.934597)
Supplement: Supplementary file 1 [file DataSheet2.PDF]

```
EXAMINE VARIABLES=UREA CREATININE GLUCOSE LDH AMYLASE MPO TNF Bax BCL2 PI3K AK
T mTOR
    caspaseexocrine caspaseendocrine antiinsulinantibodypancreasescore BY Gro
ups
  /PLOT BOXPLOT NPPLOT
  /COMPARE GROUPS
  /STATISTICS DESCRIPTIVES
  /CINTERVAL 95
  /MISSING LISTWISE
  /NOTOTAL.
```

Explore

Notes

Output Created		12-MAR-2022 16:04:00
Comments		
Input	Data	C:\Users\Dr_Abeer\Desktop\statistics\dr asmaa shams paper\normality.sav
	Active Dataset	DataSet1
	Filter	<none>
	Weight	<none>
	Split File	<none>
	N of Rows in Working Data File	36
Missing Value Handling	Definition of Missing	User-defined missing values for dependent variables are treated as missing.
	Cases Used	Statistics are based on cases with no missing values for any dependent variable or factor used.

Notes

Syntax		EXAMINE VARIABLES=UREA CREATININE GLUCOSE LDH AMYLASE MPO TNF Bax BCL2 PI3K AKT mTOR<br>caspaseexocrine caspaseendocrine antiinsulinantibody pancreasescore BY Groups<br>/PLOT BOXPLOT NPPLOT<br>/COMPARE GROUPS<br>/STATISTICS DESCRIPTIVES<br>/CINTERVAL 95<br>/MISSING LISTWISE<br>/NOTOTAL.
Resources	Processor Time	00:00:23.23
	Elapsed Time	00:00:23.19

Groups

Case Processing Summary

	Groups	Cases Valid N	Valid Percent	Missing N	Missing Percent	Total N	Total Percent
UREA	Control	6	100.0%	0	0.0%	6	100.0%
	I/R	6	100.0%	0	0.0%	6	100.0%
	I/R -NC	6	100.0%	0	0.0%	6	100.0%
	I/R-MSCs	6	100.0%	0	0.0%	6	100.0%
	I/R-MSCC	6	100.0%	0	0.0%	6	100.0%
	I/R-NC-MSCC	6	100.0%	0	0.0%	6	100.0%
CREATININE	Control	6	100.0%	0	0.0%	6	100.0%
	I/R	6	100.0%	0	0.0%	6	100.0%
	I/R -NC	6	100.0%	0	0.0%	6	100.0%
	I/R-MSCs	6	100.0%	0	0.0%	6	100.0%
	I/R-MSCC	6	100.0%	0	0.0%	6	100.0%
	I/R-NC-MSCC	6	100.0%	0	0.0%	6	100.0%
GLUCOSE	Control	6	100.0%	0	0.0%	6	100.0%
	I/R	6	100.0%	0	0.0%	6	100.0%
	I/R -NC	6	100.0%	0	0.0%	6	100.0%
	I/R-MSCs	6	100.0%	0	0.0%	6	100.0%
	I/R-MSCC	6	100.0%	0	0.0%	6	100.0%
	I/R-NC-MSCC	6	100.0%	0	0.0%	6	100.0%
LDH	Control	6	100.0%	0	0.0%	6	100.0%
	I/R	6	100.0%	0	0.0%	6	100.0%
	I/R -NC	6	100.0%	0	0.0%	6	100.0%
	I/R-MSCs	6	100.0%	0	0.0%	6	100.0%
	I/R-MSCC	6	100.0%	0	0.0%	6	100.0%
	I/R-NC-MSCC	6	100.0%	0	0.0%	6	100.0%
AMYLASE	Control	6	100.0%	0	0.0%	6	100.0%
	I/R	6	100.0%	0	0.0%	6	100.0%
	I/R -NC	6	100.0%	0	0.0%	6	100.0%
	I/R-MSCs	6	100.0%	0	0.0%	6	100.0%
	I/R-MSCC	6	100.0%	0	0.0%	6	100.0%
	I/R-NC-MSCC	6	100.0%	0	0.0%	6	100.0%
MPO	Control	6	100.0%	0	0.0%	6	100.0%
	I/R	6	100.0%	0	0.0%	6	100.0%
	I/R -NC	6	100.0%	0	0.0%	6	100.0%

Case Processing Summary

	Groups	Cases					
		Valid		Missing		Total	
		N	Percent	N	Percent	N	Percent
	I/R-MSCs	6	100.0%	0	0.0%	6	100.0%
	I/R-MSCC	6	100.0%	0	0.0%	6	100.0%
	I/R-NC-MSCC	6	100.0%	0	0.0%	6	100.0%
TNF	Control	6	100.0%	0	0.0%	6	100.0%
	I/R	6	100.0%	0	0.0%	6	100.0%
	I/R -NC	6	100.0%	0	0.0%	6	100.0%
	I/R-MSCs	6	100.0%	0	0.0%	6	100.0%
	I/R-MSCC	6	100.0%	0	0.0%	6	100.0%
	I/R-NC-MSCC	6	100.0%	0	0.0%	6	100.0%
Bax	Control	6	100.0%	0	0.0%	6	100.0%
	I/R	6	100.0%	0	0.0%	6	100.0%
	I/R -NC	6	100.0%	0	0.0%	6	100.0%
	I/R-MSCs	6	100.0%	0	0.0%	6	100.0%
	I/R-MSCC	6	100.0%	0	0.0%	6	100.0%
	I/R-NC-MSCC	6	100.0%	0	0.0%	6	100.0%
BCL2	Control	6	100.0%	0	0.0%	6	100.0%
	I/R	6	100.0%	0	0.0%	6	100.0%
	I/R -NC	6	100.0%	0	0.0%	6	100.0%
	I/R-MSCs	6	100.0%	0	0.0%	6	100.0%
	I/R-MSCC	6	100.0%	0	0.0%	6	100.0%
	I/R-NC-MSCC	6	100.0%	0	0.0%	6	100.0%
PI3K	Control	6	100.0%	0	0.0%	6	100.0%
	I/R	6	100.0%	0	0.0%	6	100.0%
	I/R -NC	6	100.0%	0	0.0%	6	100.0%
	I/R-MSCs	6	100.0%	0	0.0%	6	100.0%
	I/R-MSCC	6	100.0%	0	0.0%	6	100.0%
	I/R-NC-MSCC	6	100.0%	0	0.0%	6	100.0%
AKT	Control	6	100.0%	0	0.0%	6	100.0%
	I/R	6	100.0%	0	0.0%	6	100.0%
	I/R -NC	6	100.0%	0	0.0%	6	100.0%
	I/R-MSCs	6	100.0%	0	0.0%	6	100.0%
	I/R-MSCC	6	100.0%	0	0.0%	6	100.0%
	I/R-NC-MSCC	6	100.0%	0	0.0%	6	100.0%

Case Processing Summary

	Groups	Cases					
		Valid		Missing		Total	
		N	Percent	N	Percent	N	Percent
mTOR	Control	6	100.0%	0	0.0%	6	100.0%
	I/R	6	100.0%	0	0.0%	6	100.0%
	I/R -NC	6	100.0%	0	0.0%	6	100.0%
	I/R-MSCs	6	100.0%	0	0.0%	6	100.0%
	I/R-MSCC	6	100.0%	0	0.0%	6	100.0%
	I/R-NC-MSCC	6	100.0%	0	0.0%	6	100.0%
caspaseexocrine	Control	6	100.0%	0	0.0%	6	100.0%
	I/R	6	100.0%	0	0.0%	6	100.0%
	I/R -NC	6	100.0%	0	0.0%	6	100.0%
	I/R-MSCs	6	100.0%	0	0.0%	6	100.0%
	I/R-MSCC	6	100.0%	0	0.0%	6	100.0%
	I/R-NC-MSCC	6	100.0%	0	0.0%	6	100.0%
caspaseendocrine	Control	6	100.0%	0	0.0%	6	100.0%
	I/R	6	100.0%	0	0.0%	6	100.0%
	I/R -NC	6	100.0%	0	0.0%	6	100.0%
	I/R-MSCs	6	100.0%	0	0.0%	6	100.0%
	I/R-MSCC	6	100.0%	0	0.0%	6	100.0%
	I/R-NC-MSCC	6	100.0%	0	0.0%	6	100.0%
antiinsulinantibody	Control	6	100.0%	0	0.0%	6	100.0%
	I/R	6	100.0%	0	0.0%	6	100.0%
	I/R -NC	6	100.0%	0	0.0%	6	100.0%
	I/R-MSCs	6	100.0%	0	0.0%	6	100.0%
	I/R-MSCC	6	100.0%	0	0.0%	6	100.0%
	I/R-NC-MSCC	6	100.0%	0	0.0%	6	100.0%
pancreasescore	Control	6	100.0%	0	0.0%	6	100.0%
	I/R	6	100.0%	0	0.0%	6	100.0%
	I/R -NC	6	100.0%	0	0.0%	6	100.0%
	I/R-MSCs	6	100.0%	0	0.0%	6	100.0%
	I/R-MSCC	6	100.0%	0	0.0%	6	100.0%
	I/R-NC-MSCC	6	100.0%	0	0.0%	6	100.0%

Descriptives

	Groups			Statistic	Std. Error
UREA	Control	Mean		38.2500	.58352
		95% Confidence Interval for Mean	Lower Bound	36.7500	
			Upper Bound	39.7500	
		5% Trimmed Mean		38.2500	
		Median		38.2500	
		Variance		2.043	
		Std. Deviation		1.42934	
		Minimum		36.30	
		Maximum		40.20	
		Range		3.90	
		Interquartile Range		2.55	
		Skewness		.000	.845
		Kurtosis		-.979	1.741
	I/R	Mean		108.5667	4.49412
		95% Confidence Interval for Mean	Lower Bound	97.0142	
			Upper Bound	120.1192	
		5% Trimmed Mean		108.4352	
		Median		107.2000	
		Variance		121.183	
		Std. Deviation		11.00830	
		Minimum		95.40	
		Maximum		124.10	
		Range		28.70	
		Interquartile Range		19.70	
		Skewness		.301	.845
		Kurtosis		-1.497	1.741
	I/R -NC	Mean		72.5500	2.48230
		95% Confidence Interval for Mean	Lower Bound	66.1690	
			Upper Bound	78.9310	
		5% Trimmed Mean		72.7778	
		Median		73.6500	
		Variance		36.971	
		Std. Deviation		6.08038	
		Minimum		62.50	
		Maximum		78.50	

Descriptives

Groups				Statistic	Std. Error
		Range		16.00	
		Interquartile Range		11.05	
		Skewness		-.899	.845
		Kurtosis		.241	1.741
	I/R-MSCs	Mean		62.6000	2.22366
		95% Confidence Interval for Mean	Lower Bound	56.8839	
			Upper Bound	68.3161	
		5% Trimmed Mean		62.7556	
		Median		63.9500	
		Variance		29.668	
		Std. Deviation		5.44683	
		Minimum		53.60	
		Maximum		68.80	
		Range		15.20	
		Interquartile Range		9.12	
		Skewness		-.894	.845
		Kurtosis		.402	1.741
	I/R-MSCC	Mean		64.4833	4.38120
		95% Confidence Interval for Mean	Lower Bound	53.2211	
			Upper Bound	75.7456	
		5% Trimmed Mean		64.4481	
		Median		64.8000	
		Variance		115.170	
		Std. Deviation		10.73171	
		Minimum		52.30	
		Maximum		77.30	
		Range		25.00	
		Interquartile Range		19.23	
		Skewness		.029	.845
		Kurtosis		-2.716	1.741
	I/R-NC-MSCC	Mean		41.6000	2.21916
		95% Confidence Interval for Mean	Lower Bound	35.8955	
			Upper Bound	47.3045	
		5% Trimmed Mean		41.7444	
		Median		42.0000	

Descriptives

	Groups			Statistic	Std. Error
		Variance		29.548	
		Std. Deviation		5.43581	
		Minimum		33.10	
		Maximum		47.50	
		Range		14.40	
		Interquartile Range		10.20	
		Skewness		-.565	.845
		Kurtosis		-.340	1.741
CREATININE	Control	Mean		.1400	.01983
		95% Confidence Interval for Mean	Lower Bound	.0890	
			Upper Bound	.1910	
		5% Trimmed Mean		.1400	
		Median		.1400	
		Variance		.002	
		Std. Deviation		.04858	
		Minimum		.07	
		Maximum		.21	
		Range		.14	
		Interquartile Range		.08	
		Skewness		.000	.845
		Kurtosis		-.009	1.741
	I/R	Mean		1.9683	.03371
		95% Confidence Interval for Mean	Lower Bound	1.8817	
			Upper Bound	2.0550	
		5% Trimmed Mean		1.9665	
		Median		1.9400	
		Variance		.007	
		Std. Deviation		.08256	
		Minimum		1.87	
		Maximum		2.10	
		Range		.23	
		Interquartile Range		.13	
		Skewness		.772	.845
		Kurtosis		-.029	1.741
	I/R -NC	Mean		.6083	.01400

Descriptives

Groups				Statistic	Std. Error
		95% Confidence Interval for Mean	Lower Bound	.5723	
			Upper Bound	.6443	
		5% Trimmed Mean		.6081	
		Median		.6050	
		Variance		.001	
		Std. Deviation		.03430	
		Minimum		.56	
		Maximum		.66	
		Range		.10	
		Interquartile Range		.05	
		Skewness		.211	.845
		Kurtosis		.283	1.741
	I/R-MSCs	Mean		.7150	.03766
		95% Confidence Interval for Mean	Lower Bound	.6182	
			Upper Bound	.8118	
		5% Trimmed Mean		.7133	
		Median		.6800	
		Variance		.009	
		Std. Deviation		.09225	
		Minimum		.63	
		Maximum		.83	
		Range		.20	
		Interquartile Range		.19	
		Skewness		.702	.845
		Kurtosis		-1.970	1.741
	I/R-MSCC	Mean		.6800	.02463
		95% Confidence Interval for Mean	Lower Bound	.6167	
			Upper Bound	.7433	
		5% Trimmed Mean		.6794	
		Median		.6800	
		Variance		.004	
		Std. Deviation		.06033	
		Minimum		.59	
		Maximum		.78	
		Range		.19	

Descriptives

	Groups			Statistic	Std. Error
		Interquartile Range		.06	
		Skewness		.369	.845
		Kurtosis		2.500	1.741
	I/R-NC-MSCC	Mean		.1767	.03844
		95% Confidence Interval for Mean	Lower Bound	.0778	
			Upper Bound	.2755	
		5% Trimmed Mean		.1796	
		Median		.2000	
		Variance		.009	
		Std. Deviation		.09416	
		Minimum		.02	
		Maximum		.28	
		Range		.26	
		Interquartile Range		.16	
		Skewness		-.930	.845
		Kurtosis		.396	1.741
GLUCOSE	Control	Mean		4.2650	.15420
		95% Confidence Interval for Mean	Lower Bound	3.8686	
			Upper Bound	4.6614	
		5% Trimmed Mean		4.2650	
		Median		4.2650	
		Variance		.143	
		Std. Deviation		.37772	
		Minimum		3.83	
		Maximum		4.70	
		Range		.87	
		Interquartile Range		.72	
		Skewness		.000	.845
		Kurtosis		-2.711	1.741
	I/R	Mean		11.4000	.33566
		95% Confidence Interval for Mean	Lower Bound	10.5372	
			Upper Bound	12.2628	
		5% Trimmed Mean		11.3889	
		Median		11.4500	
		Variance		.676	

Descriptives

Groups				Statistic	Std. Error
		Std. Deviation		.82219	
		Minimum		10.40	
		Maximum		12.60	
		Range		2.20	
		Interquartile Range		1.52	
		Skewness		.188	.845
		Kurtosis		-.816	1.741
	I/R -NC	Mean		9.3300	.13844
		95% Confidence Interval for Mean	Lower Bound	8.9741	
			Upper Bound	9.6859	
		5% Trimmed Mean		9.3344	
		Median		9.3500	
		Variance		.115	
		Std. Deviation		.33912	
		Minimum		8.80	
		Maximum		9.78	
		Range		.98	
		Interquartile Range		.47	
		Skewness		-.402	.845
		Kurtosis		.216	1.741
	I/R-MSCs	Mean		9.2667	.19777
		95% Confidence Interval for Mean	Lower Bound	8.7583	
			Upper Bound	9.7750	
		5% Trimmed Mean		9.2519	
		Median		9.2500	
		Variance		.235	
		Std. Deviation		.48442	
		Minimum		8.70	
		Maximum		10.10	
		Range		1.40	
		Interquartile Range		.72	
		Skewness		.922	.845
		Kurtosis		1.425	1.741
	I/R-MSCC	Mean		8.3317	.18093

Descriptives

Groups				Statistic	Std. Error
		95% Confidence Interval for Mean	Lower Bound	7.8666	
			Upper Bound	8.7968	
		5% Trimmed Mean		8.3296	
		Median		8.3500	
		Variance		.196	
		Std. Deviation		.44319	
		Minimum		7.80	
		Maximum		8.90	
		Range		1.10	
		Interquartile Range		.88	
		Skewness		-.001	.845
		Kurtosis		-1.863	1.741
	I/R-NC-MSCC	Mean		5.5000	.15055
		95% Confidence Interval for Mean	Lower Bound	5.1130	
			Upper Bound	5.8870	
		5% Trimmed Mean		5.5167	
		Median		5.6500	
		Variance		.136	
		Std. Deviation		.36878	
		Minimum		4.90	
		Maximum		5.80	
		Range		.90	
		Interquartile Range		.67	
		Skewness		-1.077	.845
		Kurtosis		-.361	1.741
LDH	Control	Mean		115.0500	4.37270
		95% Confidence Interval for Mean	Lower Bound	103.8096	
			Upper Bound	126.2904	
		5% Trimmed Mean		115.0500	
		Median		115.0500	
		Variance		114.723	
		Std. Deviation		10.71088	
		Minimum		99.60	
		Maximum		130.50	
		Range		30.90	

Descriptives

Groups			Statistic	Std. Error
	Interquartile Range		17.40	
	Skewness		.000	.845
	Kurtosis		.000	1.741
I/R	Mean		247.5000	9.76620
	95% Confidence Interval for Mean	Lower Bound	222.3952	
		Upper Bound	272.6048	
	5% Trimmed Mean		247.2222	
	Median		246.2500	
	Variance		572.272	
	Std. Deviation		23.92221	
	Minimum		213.40	
	Maximum		286.60	
	Range		73.20	
	Interquartile Range		31.58	
	Skewness		.427	.845
	Kurtosis		1.665	1.741
I/R -NC	Mean		156.7667	3.61651
	95% Confidence Interval for Mean	Lower Bound	147.4701	
		Upper Bound	166.0632	
	5% Trimmed Mean		156.4241	
	Median		154.1500	
	Variance		78.475	
	Std. Deviation		8.85859	
	Minimum		147.30	
	Maximum		172.40	
	Range		25.10	
	Interquartile Range		13.32	
	Skewness		1.240	.845
	Kurtosis		1.581	1.741
I/R-MSCs	Mean		179.9833	8.23745
	95% Confidence Interval for Mean	Lower Bound	158.8083	
		Upper Bound	201.1584	
	5% Trimmed Mean		180.7426	
	Median		188.7500	
	Variance		407.134	

Descriptives

	Groups			Statistic	Std. Error
		Std. Deviation		20.17755	
		Minimum		147.20	
		Maximum		199.10	
		Range		51.90	
		Interquartile Range		34.50	
		Skewness		-1.049	.845
		Kurtosis		-.316	1.741
	I/R-MSCC	Mean		165.0000	8.36134
		95% Confidence Interval for Mean	Lower Bound	143.5065	
			Upper Bound	186.4935	
		5% Trimmed Mean		164.9056	
		Median		165.8500	
		Variance		419.472	
		Std. Deviation		20.48102	
		Minimum		144.20	
		Maximum		187.50	
		Range		43.30	
		Interquartile Range		39.33	
		Skewness		-.007	.845
		Kurtosis		-3.025	1.741
	I/R-NC-MSCC	Mean		137.1333	2.68013
		95% Confidence Interval for Mean	Lower Bound	130.2438	
			Upper Bound	144.0228	
		5% Trimmed Mean		137.0481	
		Median		137.9000	
		Variance		43.099	
		Std. Deviation		6.56496	
		Minimum		129.60	
		Maximum		146.20	
		Range		16.60	
		Interquartile Range		12.85	
		Skewness		.010	.845
		Kurtosis		-1.268	1.741
AMYLASE	Control	Mean		305.3500	10.48016

Descriptives

Groups				Statistic	Std. Error
		95% Confidence Interval for Mean	Lower Bound	278.4099	
			Upper Bound	332.2901	
		5% Trimmed Mean		305.3500	
		Median		305.3500	
		Variance		659.003	
		Std. Deviation		25.67105	
		Minimum		268.30	
		Maximum		342.40	
		Range		74.10	
		Interquartile Range		41.10	
		Skewness		.000	.845
		Kurtosis		-.003	1.741
	I/R	Mean		574.1333	14.78988
		95% Confidence Interval for Mean	Lower Bound	536.1147	
			Upper Bound	612.1519	
		5% Trimmed Mean		574.5315	
		Median		570.5500	
		Variance		1312.443	
		Std. Deviation		36.22765	
		Minimum		523.80	
		Maximum		617.30	
		Range		93.50	
		Interquartile Range		66.57	
		Skewness		-.094	.845
		Kurtosis		-1.421	1.741
	I/R -NC	Mean		400.9667	12.34522
		95% Confidence Interval for Mean	Lower Bound	369.2323	
			Upper Bound	432.7011	
		5% Trimmed Mean		401.1685	
		Median		409.2500	
		Variance		914.427	
		Std. Deviation		30.23949	
		Minimum		356.20	
		Maximum		442.10	
		Range		85.90	

Descriptives

Groups			Statistic	Std. Error
	Interquartile Range		48.63	
	Skewness		-.350	.845
	Kurtosis		-.163	1.741
I/R-MSCs	Mean		379.9500	14.85635
	95% Confidence Interval for Mean	Lower Bound	341.7605	
		Upper Bound	418.1395	
	5% Trimmed Mean		380.2000	
	Median		381.0500	
	Variance		1324.267	
	Std. Deviation		36.39048	
	Minimum		325.10	
	Maximum		430.30	
	Range		105.20	
	Interquartile Range		51.05	
	Skewness		-.210	.845
	Kurtosis		.046	1.741
I/R-MSCC	Mean		352.9833	15.71550
	95% Confidence Interval for Mean	Lower Bound	312.5854	
		Upper Bound	393.3813	
	5% Trimmed Mean		354.1093	
	Median		361.1500	
	Variance		1481.862	
	Std. Deviation		38.49496	
	Minimum		291.70	
	Maximum		394.00	
	Range		102.30	
	Interquartile Range		70.95	
	Skewness		-.785	.845
	Kurtosis		-.301	1.741
I/R-NC-MSCC	Mean		317.6833	8.25705
	95% Confidence Interval for Mean	Lower Bound	296.4579	
		Upper Bound	338.9088	
	5% Trimmed Mean		317.4704	
	Median		318.0000	
	Variance		409.074	

Descriptives

	Groups			Statistic	Std. Error
		Std. Deviation		20.22557	
		Minimum		288.30	
		Maximum		350.90	
		Range		62.60	
		Interquartile Range		24.42	
		Skewness		.392	.845
		Kurtosis		1.979	1.741
MPO	Control	Mean		117.5500	3.92146
		95% Confidence Interval for Mean	Lower Bound	107.4696	
			Upper Bound	127.6304	
		5% Trimmed Mean		117.5500	
		Median		117.5500	
		Variance		92.267	
		Std. Deviation		9.60557	
		Minimum		103.70	
		Maximum		131.40	
		Range		27.70	
		Interquartile Range		15.70	
		Skewness		.000	.845
		Kurtosis		-.003	1.741
	I/R	Mean		320.1000	4.17955
		95% Confidence Interval for Mean	Lower Bound	309.3561	
			Upper Bound	330.8439	
		5% Trimmed Mean		319.9667	
		Median		318.9000	
		Variance		104.812	
		Std. Deviation		10.23777	
		Minimum		308.40	
		Maximum		334.20	
		Range		25.80	
		Interquartile Range		20.40	
		Skewness		.322	.845
		Kurtosis		-1.572	1.741
	I/R -NC	Mean		204.3167	3.99211

Descriptives

Groups				Statistic	Std. Error
		95% Confidence Interval for Mean	Lower Bound	194.0546	
			Upper Bound	214.5787	
		5% Trimmed Mean		204.3574	
		Median		203.9500	
		Variance		95.622	
		Std. Deviation		9.77863	
		Minimum		191.10	
		Maximum		216.80	
		Range		25.70	
		Interquartile Range		19.25	
		Skewness		-.013	.845
		Kurtosis		-1.200	1.741
	I/R-MSCs	Mean		192.9333	7.00279
		95% Confidence Interval for Mean	Lower Bound	174.9321	
			Upper Bound	210.9346	
		5% Trimmed Mean		192.9537	
		Median		189.7500	
		Variance		294.235	
		Std. Deviation		17.15327	
		Minimum		170.40	
		Maximum		215.10	
		Range		44.70	
		Interquartile Range		30.97	
		Skewness		.131	.845
		Kurtosis		-1.461	1.741
	I/R-MSCC	Mean		187.8833	6.32963
		95% Confidence Interval for Mean	Lower Bound	171.6125	
			Upper Bound	204.1542	
		5% Trimmed Mean		188.1704	
		Median		192.9000	
		Variance		240.386	
		Std. Deviation		15.50438	
		Minimum		163.40	
		Maximum		207.20	
		Range		43.80	

Descriptives

	Groups			Statistic	Std. Error
		Interquartile Range		24.53	
		Skewness		-.675	.845
		Kurtosis		.045	1.741
	I/R-NC-MSCC	Mean		146.7167	6.81226
		95% Confidence Interval for Mean	Lower Bound	129.2052	
			Upper Bound	164.2281	
		5% Trimmed Mean		146.6685	
		Median		149.7000	
		Variance		278.442	
		Std. Deviation		16.68657	
		Minimum		125.40	
		Maximum		168.90	
		Range		43.50	
		Interquartile Range		30.08	
		Skewness		-.095	.845
		Kurtosis		-1.440	1.741
TNF	Control	Mean		15.4833	.33408
		95% Confidence Interval for Mean	Lower Bound	14.6245	
			Upper Bound	16.3421	
		5% Trimmed Mean		15.5204	
		Median		15.6500	
		Variance		.670	
		Std. Deviation		.81833	
		Minimum		14.10	
		Maximum		16.20	
		Range		2.10	
		Interquartile Range		1.20	
		Skewness		-1.016	.845
		Kurtosis		.349	1.741
	I/R	Mean		107.8833	1.60404
		95% Confidence Interval for Mean	Lower Bound	103.7600	
			Upper Bound	112.0066	
		5% Trimmed Mean		108.0148	
		Median		108.9500	
		Variance		15.438	

Descriptives

Groups				Statistic	Std. Error
		Std. Deviation		3.92908	
		Minimum		101.30	
		Maximum		112.10	
		Range		10.80	
		Interquartile Range		6.60	
		Skewness		-1.001	.845
		Kurtosis		.480	1.741
	I/R -NC	Mean		69.8500	2.27490
		95% Confidence Interval for Mean	Lower Bound	64.0022	
			Upper Bound	75.6978	
		5% Trimmed Mean		69.7056	
		Median		66.8500	
		Variance		31.051	
		Std. Deviation		5.57234	
		Minimum		65.20	
		Maximum		77.10	
		Range		11.90	
		Interquartile Range		11.00	
		Skewness		.915	.845
		Kurtosis		-1.863	1.741
	I/R-MSCs	Mean		58.4167	1.50763
		95% Confidence Interval for Mean	Lower Bound	54.5412	
			Upper Bound	62.2921	
		5% Trimmed Mean		58.2796	
		Median		56.6500	
		Variance		13.638	
		Std. Deviation		3.69292	
		Minimum		55.50	
		Maximum		63.80	
		Range		8.30	
		Interquartile Range		7.10	
		Skewness		.886	.845
		Kurtosis		-1.545	1.741
	I/R-MSCC	Mean		44.4833	1.25709

Descriptives

	Groups			Statistic	Std. Error
		95% Confidence Interval for Mean	Lower Bound	41.2519	
			Upper Bound	47.7148	
		5% Trimmed Mean		44.5815	
		Median		45.0000	
		Variance		9.482	
		Std. Deviation		3.07923	
		Minimum		39.60	
		Maximum		47.60	
		Range		8.00	
		Interquartile Range		5.53	
		Skewness		-.725	.845
		Kurtosis		-.501	1.741
	I/R-NC-MSCC	Mean		28.3333	.39129
		95% Confidence Interval for Mean	Lower Bound	27.3275	
			Upper Bound	29.3392	
		5% Trimmed Mean		28.3370	
		Median		28.5000	
		Variance		.919	
		Std. Deviation		.95847	
		Minimum		27.10	
		Maximum		29.50	
		Range		2.40	
		Interquartile Range		1.95	
		Skewness		-.317	.845
		Kurtosis		-1.561	1.741
Bax	Control	Mean		1.0300	.00856
		95% Confidence Interval for Mean	Lower Bound	1.0080	
			Upper Bound	1.0520	
		5% Trimmed Mean		1.0300	
		Median		1.0300	
		Variance		.000	
		Std. Deviation		.02098	
		Minimum		1.00	
		Maximum		1.06	
		Range		.06	

Descriptives

Groups				Statistic	Std. Error
		Interquartile Range		.03	
		Skewness		.000	.845
		Kurtosis		-.248	1.741
	I/R	Mean		7.5367	.11141
		95% Confidence Interval for Mean	Lower Bound	7.2503	
			Upper Bound	7.8230	
		5% Trimmed Mean		7.5230	
		Median		7.5000	
		Variance		.074	
		Std. Deviation		.27289	
		Minimum		7.30	
		Maximum		8.02	
		Range		.72	
		Interquartile Range		.41	
		Skewness		1.245	.845
		Kurtosis		1.479	1.741
	I/R -NC	Mean		4.2517	.10719
		95% Confidence Interval for Mean	Lower Bound	3.9761	
			Upper Bound	4.5272	
		5% Trimmed Mean		4.2396	
		Median		4.1550	
		Variance		.069	
		Std. Deviation		.26256	
		Minimum		4.01	
		Maximum		4.71	
		Range		.70	
		Interquartile Range		.42	
		Skewness		1.273	.845
		Kurtosis		1.030	1.741
	I/R-MSCs	Mean		3.4817	.12029
		95% Confidence Interval for Mean	Lower Bound	3.1725	
			Upper Bound	3.7909	
		5% Trimmed Mean		3.4796	
		Median		3.5500	
		Variance		.087	

Descriptives

Groups				Statistic	Std. Error
		Std. Deviation		.29465	
		Minimum		3.10	
		Maximum		3.90	
		Range		.80	
		Interquartile Range		.51	
		Skewness		-.045	.845
		Kurtosis		-.726	1.741
	I/R-MSCC	Mean		2.6500	.04282
		95% Confidence Interval for Mean	Lower Bound	2.5399	
			Upper Bound	2.7601	
		5% Trimmed Mean		2.6500	
		Median		2.6500	
		Variance		.011	
		Std. Deviation		.10488	
		Minimum		2.50	
		Maximum		2.80	
		Range		.30	
		Interquartile Range		.15	
		Skewness		.000	.845
		Kurtosis		-.248	1.741
	I/R-NC-MSCC	Mean		1.7233	.03712
		95% Confidence Interval for Mean	Lower Bound	1.6279	
			Upper Bound	1.8187	
		5% Trimmed Mean		1.7226	
		Median		1.7200	
		Variance		.008	
		Std. Deviation		.09092	
		Minimum		1.62	
		Maximum		1.84	
		Range		.22	
		Interquartile Range		.19	
		Skewness		.126	.845
		Kurtosis		-1.825	1.741
BCL2	Control	Mean		1.0050	.01565

Descriptives

Groups				Statistic	Std. Error
		95% Confidence Interval for Mean	Lower Bound	.9648	
			Upper Bound	1.0452	
		5% Trimmed Mean		1.0050	
		Median		1.0050	
		Variance		.001	
		Std. Deviation		.03834	
		Minimum		.95	
		Maximum		1.06	
		Range		.11	
		Interquartile Range		.07	
		Skewness		.000	.845
		Kurtosis		-.068	1.741
	I/R	Mean		.2650	.00992
		95% Confidence Interval for Mean	Lower Bound	.2395	
			Upper Bound	.2905	
		5% Trimmed Mean		.2644	
		Median		.2550	
		Variance		.001	
		Std. Deviation		.02429	
		Minimum		.24	
		Maximum		.30	
		Range		.06	
		Interquartile Range		.04	
		Skewness		.754	.845
		Kurtosis		-1.456	1.741
	I/R -NC	Mean		.4150	.00847
		95% Confidence Interval for Mean	Lower Bound	.3932	
			Upper Bound	.4368	
		5% Trimmed Mean		.4156	
		Median		.4150	
		Variance		.000	
		Std. Deviation		.02074	
		Minimum		.38	
		Maximum		.44	
		Range		.06	

Descriptives

Groups				Statistic	Std. Error
		Interquartile Range		.03	
		Skewness		-.807	.845
		Kurtosis		1.109	1.741
	I/R-MSCs	Mean		.5500	.01483
		95% Confidence Interval for Mean	Lower Bound	.5119	
			Upper Bound	.5881	
		5% Trimmed Mean		.5511	
		Median		.5550	
		Variance		.001	
		Std. Deviation		.03633	
		Minimum		.49	
		Maximum		.59	
		Range		.10	
		Interquartile Range		.06	
		Skewness		-.826	.845
		Kurtosis		.379	1.741
	I/R-MSCC	Mean		.6950	.02125
		95% Confidence Interval for Mean	Lower Bound	.6404	
			Upper Bound	.7496	
		5% Trimmed Mean		.6944	
		Median		.6950	
		Variance		.003	
		Std. Deviation		.05206	
		Minimum		.63	
		Maximum		.77	
		Range		.14	
		Interquartile Range		.09	
		Skewness		.211	.845
		Kurtosis		-.989	1.741
	I/R-NC-MSCC	Mean		.8433	.03676
		95% Confidence Interval for Mean	Lower Bound	.7488	
			Upper Bound	.9378	
		5% Trimmed Mean		.8437	
		Median		.8400	
		Variance		.008	

Descriptives

	Groups			Statistic	Std. Error
		Std. Deviation		.09004	
		Minimum		.72	
		Maximum		.96	
		Range		.24	
		Interquartile Range		.17	
		Skewness		-.045	.845
		Kurtosis		-1.254	1.741
PI3K	Control	Mean		1.0217	.01138
		95% Confidence Interval for Mean	Lower Bound	.9924	
			Upper Bound	1.0509	
		5% Trimmed Mean		1.0213	
		Median		1.0250	
		Variance		.001	
		Std. Deviation		.02787	
		Minimum		.99	
		Maximum		1.06	
		Range		.07	
		Interquartile Range		.05	
		Skewness		-.006	.845
		Kurtosis		-1.274	1.741
	I/R	Mean		.2117	.00477
		95% Confidence Interval for Mean	Lower Bound	.1994	
			Upper Bound	.2239	
		5% Trimmed Mean		.2113	
		Median		.2100	
		Variance		.000	
		Std. Deviation		.01169	
		Minimum		.20	
		Maximum		.23	
		Range		.03	
		Interquartile Range		.02	
		Skewness		.668	.845
		Kurtosis		-.446	1.741
	I/R -NC	Mean		.5417	.01014

Descriptives

Groups				Statistic	Std. Error
		95% Confidence Interval for Mean	Lower Bound	.5156	
			Upper Bound	.5677	
		5% Trimmed Mean		.5424	
		Median		.5450	
		Variance		.001	
		Std. Deviation		.02483	
		Minimum		.50	
		Maximum		.57	
		Range		.07	
		Interquartile Range		.04	
		Skewness		-.871	.845
		Kurtosis		.735	1.741
	I/R-MSCs	Mean		.5050	.00764
		95% Confidence Interval for Mean	Lower Bound	.4854	
			Upper Bound	.5246	
		5% Trimmed Mean		.5050	
		Median		.5050	
		Variance		.000	
		Std. Deviation		.01871	
		Minimum		.48	
		Maximum		.53	
		Range		.05	
		Interquartile Range		.03	
		Skewness		.000	.845
		Kurtosis		-1.200	1.741
	I/R-MSCC	Mean		.6367	.00882
		95% Confidence Interval for Mean	Lower Bound	.6140	
			Upper Bound	.6593	
		5% Trimmed Mean		.6363	
		Median		.6350	
		Variance		.000	
		Std. Deviation		.02160	
		Minimum		.61	
		Maximum		.67	
		Range		.06	

Descriptives

	Groups			Statistic	Std. Error
		Interquartile Range		.04	
		Skewness		.463	.845
		Kurtosis		-.300	1.741
	I/R-NC-MSCC	Mean		.8983	.00601
		95% Confidence Interval for Mean	Lower Bound	.8829	
			Upper Bound	.9138	
		5% Trimmed Mean		.8981	
		Median		.8950	
		Variance		.000	
		Std. Deviation		.01472	
		Minimum		.88	
		Maximum		.92	
		Range		.04	
		Interquartile Range		.03	
		Skewness		.418	.845
		Kurtosis		-.859	1.741
AKT	Control	Mean		1.0200	.00365
		95% Confidence Interval for Mean	Lower Bound	1.0106	
			Upper Bound	1.0294	
		5% Trimmed Mean		1.0200	
		Median		1.0200	
		Variance		.000	
		Std. Deviation		.00894	
		Minimum		1.01	
		Maximum		1.03	
		Range		.02	
		Interquartile Range		.02	
		Skewness		.000	.845
		Kurtosis		-1.875	1.741
	I/R	Mean		.1950	.01335
		95% Confidence Interval for Mean	Lower Bound	.1607	
			Upper Bound	.2293	
		5% Trimmed Mean		.1944	
		Median		.1950	
		Variance		.001	

Descriptives

Groups				Statistic	Std. Error
		Std. Deviation		.03271	
		Minimum		.15	
		Maximum		.25	
		Range		.10	
		Interquartile Range		.04	
		Skewness		.617	.845
		Kurtosis		1.884	1.741
	I/R -NC	Mean		.4967	.07770
		95% Confidence Interval for Mean	Lower Bound	.2969	
			Upper Bound	.6964	
		5% Trimmed Mean		.4974	
		Median		.5000	
		Variance		.036	
		Std. Deviation		.19033	
		Minimum		.19	
		Maximum		.79	
		Range		.60	
		Interquartile Range		.18	
		Skewness		-.156	.845
		Kurtosis		2.417	1.741
	I/R-MSCs	Mean		.5233	.07779
		95% Confidence Interval for Mean	Lower Bound	.3234	
			Upper Bound	.7233	
		5% Trimmed Mean		.5226	
		Median		.5250	
		Variance		.036	
		Std. Deviation		.19054	
		Minimum		.23	
		Maximum		.83	
		Range		.60	
		Interquartile Range		.19	
		Skewness		.155	.845
		Kurtosis		2.379	1.741
	I/R-MSCC	Mean		.6083	.01249

Descriptives

	Groups			Statistic	Std. Error
		95% Confidence Interval for Mean	Lower Bound	.5762	
			Upper Bound	.6405	
		5% Trimmed Mean		.6098	
		Median		.6150	
		Variance		.001	
		Std. Deviation		.03061	
		Minimum		.55	
		Maximum		.64	
		Range		.09	
		Interquartile Range		.03	
		Skewness		-1.712	.845
		Kurtosis		3.820	1.741
	I/R-NC-MSCC	Mean		.8917	.00833
		95% Confidence Interval for Mean	Lower Bound	.8702	
			Upper Bound	.9131	
		5% Trimmed Mean		.8907	
		Median		.8900	
		Variance		.000	
		Std. Deviation		.02041	
		Minimum		.87	
		Maximum		.93	
		Range		.06	
		Interquartile Range		.02	
		Skewness		1.572	.845
		Kurtosis		3.420	1.741
mTOR	Control	Mean		1.0050	.12912
		95% Confidence Interval for Mean	Lower Bound	.6731	
			Upper Bound	1.3369	
		5% Trimmed Mean		1.0044	
		Median		1.0050	
		Variance		.100	
		Std. Deviation		.31628	
		Minimum		.61	
		Maximum		1.41	
		Range		.80	

Descriptives

Groups			Statistic	Std. Error
	Interquartile Range		.65	
	Skewness		.020	.845
	Kurtosis		-1.530	1.741
I/R	Mean		.1883	.01447
	95% Confidence Interval for Mean	Lower Bound	.1511	
		Upper Bound	.2255	
	5% Trimmed Mean		.1898	
	Median		.2050	
	Variance		.001	
	Std. Deviation		.03545	
	Minimum		.13	
	Maximum		.22	
	Range		.09	
	Interquartile Range		.06	
	Skewness		-1.128	.845
	Kurtosis		-.183	1.741
I/R -NC	Mean		.5017	.07765
	95% Confidence Interval for Mean	Lower Bound	.3021	
		Upper Bound	.7013	
	5% Trimmed Mean		.5030	
	Median		.5100	
	Variance		.036	
	Std. Deviation		.19020	
	Minimum		.19	
	Maximum		.79	
	Range		.60	
	Interquartile Range		.17	
	Skewness		-.275	.845
	Kurtosis		2.493	1.741
I/R-MSCs	Mean		.4400	.07776
	95% Confidence Interval for Mean	Lower Bound	.2401	
		Upper Bound	.6399	
	5% Trimmed Mean		.4411	
	Median		.4400	
	Variance		.036	

Descriptives

	Groups			Statistic	Std. Error
		Std. Deviation		.19047	
		Minimum		.13	
		Maximum		.73	
		Range		.60	
		Interquartile Range		.18	
		Skewness		-.233	.845
		Kurtosis		2.423	1.741
	I/R-MSCC	Mean		.5950	.01335
		95% Confidence Interval for Mean	Lower Bound	.5607	
			Upper Bound	.6293	
		5% Trimmed Mean		.5939	
		Median		.5900	
		Variance		.001	
		Std. Deviation		.03271	
		Minimum		.56	
		Maximum		.65	
		Range		.09	
		Interquartile Range		.05	
		Skewness		.926	.845
		Kurtosis		.563	1.741
	I/R-NC-MSCC	Mean		.8433	.01174
		95% Confidence Interval for Mean	Lower Bound	.8132	
			Upper Bound	.8735	
		5% Trimmed Mean		.8448	
		Median		.8500	
		Variance		.001	
		Std. Deviation		.02875	
		Minimum		.79	
		Maximum		.87	
		Range		.08	
		Interquartile Range		.04	
		Skewness		-1.560	.845
		Kurtosis		2.714	1.741
caspaseexocrine	Control	Mean		.1117	.02676

Descriptives

Groups				Statistic	Std. Error
		95% Confidence Interval for Mean	Lower Bound	.0429	
			Upper Bound	.1805	
		5% Trimmed Mean		.1113	
		Median		.0950	
		Variance		.004	
		Std. Deviation		.06555	
		Minimum		.03	
		Maximum		.20	
		Range		.17	
		Interquartile Range		.13	
		Skewness		.404	.845
		Kurtosis		-1.305	1.741
	I/R	Mean		40.5000	1.25831
		95% Confidence Interval for Mean	Lower Bound	37.2654	
			Upper Bound	43.7346	
		5% Trimmed Mean		40.5556	
		Median		41.0000	
		Variance		9.500	
		Std. Deviation		3.08221	
		Minimum		36.00	
		Maximum		44.00	
		Range		8.00	
		Interquartile Range		5.75	
		Skewness		-.461	.845
		Kurtosis		-1.260	1.741
	I/R -NC	Mean		14.1667	1.35195
		95% Confidence Interval for Mean	Lower Bound	10.6914	
			Upper Bound	17.6420	
		5% Trimmed Mean		14.1852	
		Median		14.5000	
		Variance		10.967	
		Std. Deviation		3.31160	
		Minimum		10.00	
		Maximum		18.00	
		Range		8.00	

Descriptives

Groups				Statistic	Std. Error
		Interquartile Range		6.50	
		Skewness		-.169	.845
		Kurtosis		-2.202	1.741
	I/R-MSCs	Mean		6.4333	.19777
		95% Confidence Interval for Mean	Lower Bound	5.9250	
			Upper Bound	6.9417	
		5% Trimmed Mean		6.4370	
		Median		6.4500	
		Variance		.235	
		Std. Deviation		.48442	
		Minimum		5.80	
		Maximum		7.00	
		Range		1.20	
		Interquartile Range		.98	
		Skewness		-.131	.845
		Kurtosis		-1.829	1.741
	I/R-MSCC	Mean		2.5000	.15275
		95% Confidence Interval for Mean	Lower Bound	2.1073	
			Upper Bound	2.8927	
		5% Trimmed Mean		2.5000	
		Median		2.5000	
		Variance		.140	
		Std. Deviation		.37417	
		Minimum		2.00	
		Maximum		3.00	
		Range		1.00	
		Interquartile Range		.70	
		Skewness		.000	.845
		Kurtosis		-1.200	1.741
	I/R-NC-MSCC	Mean		.5833	.13520
		95% Confidence Interval for Mean	Lower Bound	.2358	
			Upper Bound	.9309	
		5% Trimmed Mean		.5870	
		Median		.5500	
		Variance		.110	

Descriptives

	Groups			Statistic	Std. Error
		Std. Deviation		.33116	
		Minimum		.10	
		Maximum		1.00	
		Range		.90	
		Interquartile Range		.60	
		Skewness		-.128	.845
		Kurtosis		-.665	1.741
caspaseendocrine	Control	Mean		.1683	.03497
		95% Confidence Interval for Mean	Lower Bound	.0784	
			Upper Bound	.2582	
		5% Trimmed Mean		.1670	
		Median		.1800	
		Variance		.007	
		Std. Deviation		.08565	
		Minimum		.06	
		Maximum		.30	
		Range		.24	
		Interquartile Range		.14	
		Skewness		.273	.845
		Kurtosis		-.045	1.741
	I/R	Mean		5.6833	.23154
		95% Confidence Interval for Mean	Lower Bound	5.0881	
			Upper Bound	6.2785	
		5% Trimmed Mean		5.6815	
		Median		5.7000	
		Variance		.322	
		Std. Deviation		.56716	
		Minimum		5.00	
		Maximum		6.40	
		Range		1.40	
		Interquartile Range		1.18	
		Skewness		-.017	.845
		Kurtosis		-1.723	1.741
	I/R -NC	Mean		3.2833	.17401

Descriptives

Groups				Statistic	Std. Error
		95% Confidence Interval for Mean	Lower Bound	2.8360	
			Upper Bound	3.7306	
		5% Trimmed Mean		3.2704	
		Median		3.2000	
		Variance		.182	
		Std. Deviation		.42622	
		Minimum		2.80	
		Maximum		4.00	
		Range		1.20	
		Interquartile Range		.67	
		Skewness		.916	.845
		Kurtosis		.710	1.741
	I/R-MSCs	Mean		1.5333	.14530
		95% Confidence Interval for Mean	Lower Bound	1.1598	
			Upper Bound	1.9068	
		5% Trimmed Mean		1.5370	
		Median		1.5500	
		Variance		.127	
		Std. Deviation		.35590	
		Minimum		1.00	
		Maximum		2.00	
		Range		1.00	
		Interquartile Range		.63	
		Skewness		-.290	.845
		Kurtosis		-.300	1.741
	I/R-MSCC	Mean		.6500	.07638
		95% Confidence Interval for Mean	Lower Bound	.4537	
			Upper Bound	.8463	
		5% Trimmed Mean		.6500	
		Median		.6500	
		Variance		.035	
		Std. Deviation		.18708	
		Minimum		.40	
		Maximum		.90	
		Range		.50	

Descriptives

	Groups			Statistic	Std. Error
		Interquartile Range		.35	
		Skewness		.000	.845
		Kurtosis		-1.200	1.741
	I/R-NC-MSCC	Mean		.2400	.02066
		95% Confidence Interval for Mean	Lower Bound	.1869	
			Upper Bound	.2931	
		5% Trimmed Mean		.2406	
		Median		.2400	
		Variance		.003	
		Std. Deviation		.05060	
		Minimum		.17	
		Maximum		.30	
		Range		.13	
		Interquartile Range		.10	
		Skewness		-.153	.845
		Kurtosis		-1.358	1.741
antiinsulinantibody	Control	Mean		13.6683	.44347
		95% Confidence Interval for Mean	Lower Bound	12.5283	
			Upper Bound	14.8083	
		5% Trimmed Mean		13.6537	
		Median		13.4050	
		Variance		1.180	
		Std. Deviation		1.08629	
		Minimum		12.50	
		Maximum		15.10	
		Range		2.60	
		Interquartile Range		2.15	
		Skewness		.433	.845
		Kurtosis		-1.996	1.741
	I/R	Mean		3.2917	.23109
		95% Confidence Interval for Mean	Lower Bound	2.6976	
			Upper Bound	3.8857	
		5% Trimmed Mean		3.2713	
		Median		3.2500	
		Variance		.320	

Descriptives

Groups				Statistic	Std. Error
		Std. Deviation		.56605	
		Minimum		2.70	
		Maximum		4.25	
		Range		1.55	
		Interquartile Range		.91	
		Skewness		.922	.845
		Kurtosis		.758	1.741
	I/R -NC	Mean		7.8000	.19833
		95% Confidence Interval for Mean	Lower Bound	7.2902	
			Upper Bound	8.3098	
		5% Trimmed Mean		7.8167	
		Median		7.9000	
		Variance		.236	
		Std. Deviation		.48580	
		Minimum		7.00	
		Maximum		8.30	
		Range		1.30	
		Interquartile Range		.85	
		Skewness		-.895	.845
		Kurtosis		.127	1.741
	I/R-MSCs	Mean		8.9333	.15202
		95% Confidence Interval for Mean	Lower Bound	8.5425	
			Upper Bound	9.3241	
		5% Trimmed Mean		8.9315	
		Median		8.9500	
		Variance		.139	
		Std. Deviation		.37238	
		Minimum		8.40	
		Maximum		9.50	
		Range		1.10	
		Interquartile Range		.57	
		Skewness		.130	.845
		Kurtosis		.586	1.741
	I/R-MSCC	Mean		10.9667	.25517

Descriptives

	Groups			Statistic	Std. Error
		95% Confidence Interval for Mean	Lower Bound	10.3107	
			Upper Bound	11.6226	
		5% Trimmed Mean		10.9685	
		Median		10.9500	
		Variance		.391	
		Std. Deviation		.62503	
		Minimum		10.10	
		Maximum		11.80	
		Range		1.70	
		Interquartile Range		1.17	
		Skewness		-.028	.845
		Kurtosis		-.862	1.741
	I/R-NC-MSCC	Mean		13.0833	.31981
		95% Confidence Interval for Mean	Lower Bound	12.2612	
			Upper Bound	13.9054	
		5% Trimmed Mean		13.0926	
		Median		13.0500	
		Variance		.614	
		Std. Deviation		.78337	
		Minimum		12.00	
		Maximum		14.00	
		Range		2.00	
		Interquartile Range		1.55	
		Skewness		-.100	.845
		Kurtosis		-1.333	1.741
pancreasescore	Control	Mean		.1667	.16667
		95% Confidence Interval for Mean	Lower Bound	-.2618	
			Upper Bound	.5951	
		5% Trimmed Mean		.1296	
		Median		.0000	
		Variance		.167	
		Std. Deviation		.40825	
		Minimum		.00	
		Maximum		1.00	
		Range		1.00	

Descriptives

Groups				Statistic	Std. Error
		Interquartile Range		.25	
		Skewness		2.449	.845
		Kurtosis		6.000	1.741
	I/R	Mean		3.6667	.21082
		95% Confidence Interval for Mean	Lower Bound	3.1247	
			Upper Bound	4.2086	
		5% Trimmed Mean		3.6852	
		Median		4.0000	
		Variance		.267	
		Std. Deviation		.51640	
		Minimum		3.00	
		Maximum		4.00	
		Range		1.00	
		Interquartile Range		1.00	
		Skewness		-.968	.845
		Kurtosis		-1.875	1.741
	I/R -NC	Mean		3.3333	.21082
		95% Confidence Interval for Mean	Lower Bound	2.7914	
			Upper Bound	3.8753	
		5% Trimmed Mean		3.3148	
		Median		3.0000	
		Variance		.267	
		Std. Deviation		.51640	
		Minimum		3.00	
		Maximum		4.00	
		Range		1.00	
		Interquartile Range		1.00	
		Skewness		.968	.845
		Kurtosis		-1.875	1.741
	I/R-MSCs	Mean		2.1667	.30732
		95% Confidence Interval for Mean	Lower Bound	1.3767	
			Upper Bound	2.9567	
		5% Trimmed Mean		2.1852	
		Median		2.0000	
		Variance		.567	

Descriptives

Groups				Statistic	Std. Error
		Std. Deviation		.75277	
		Minimum		1.00	
		Maximum		3.00	
		Range		2.00	
		Interquartile Range		1.25	
		Skewness		-.313	.845
		Kurtosis		-.104	1.741
	I/R-MSCC	Mean		1.6667	.21082
		95% Confidence Interval for Mean	Lower Bound	1.1247	
			Upper Bound	2.2086	
		5% Trimmed Mean		1.6852	
		Median		2.0000	
		Variance		.267	
		Std. Deviation		.51640	
		Minimum		1.00	
		Maximum		2.00	
		Range		1.00	
		Interquartile Range		1.00	
		Skewness		-.968	.845
		Kurtosis		-1.875	1.741
	I/R-NC-MSCC	Mean		1.3333	.21082
		95% Confidence Interval for Mean	Lower Bound	.7914	
			Upper Bound	1.8753	
		5% Trimmed Mean		1.3148	
		Median		1.0000	
		Variance		.267	
		Std. Deviation		.51640	
		Minimum		1.00	
		Maximum		2.00	
		Range		1.00	
		Interquartile Range		1.00	
		Skewness		.968	.845
		Kurtosis		-1.875	1.741

Tests of Normality

	Groups	Kolmogorov-Smirnov[a]			Shapiro-Wilk		
		Statistic	df	Sig.	Statistic	df	Sig.
UREA	Control	.124	6	.200*	.988	6	.983
	I/R	.215	6	.200*	.951	6	.749
	I/R -NC	.229	6	.200*	.908	6	.421
	I/R-MSCs	.254	6	.200*	.937	6	.639
	I/R-MSCC	.267	6	.200*	.860	6	.189
	I/R-NC-MSCC	.189	6	.200*	.934	6	.611
CREATININE	Control	.102	6	.200*	1.000	6	1.000
	I/R	.255	6	.200*	.927	6	.555
	I/R -NC	.147	6	.200*	.992	6	.994
	I/R-MSCs	.231	6	.200*	.808	6	.070
	I/R-MSCC	.333	6	.036	.858	6	.183
	I/R-NC-MSCC	.181	6	.200*	.942	6	.678
GLUCOSE	Control	.233	6	.200*	.873	6	.237
	I/R	.168	6	.200*	.965	6	.857
	I/R -NC	.192	6	.200*	.954	6	.776
	I/R-MSCs	.225	6	.200*	.938	6	.647
	I/R-MSCC	.174	6	.200*	.941	6	.664
	I/R-NC-MSCC	.274	6	.181	.840	6	.131
LDH	Control	.107	6	.200*	.999	6	1.000
	I/R	.209	6	.200*	.958	6	.805
	I/R -NC	.276	6	.169	.901	6	.381
	I/R-MSCs	.272	6	.185	.867	6	.216
	I/R-MSCC	.275	6	.173	.805	6	.065
	I/R-NC-MSCC	.201	6	.200*	.930	6	.583
AMYLASE	Control	.112	6	.200*	.997	6	.999
	I/R	.184	6	.200*	.944	6	.693
	I/R -NC	.273	6	.185	.936	6	.628
	I/R-MSCs	.183	6	.200*	.961	6	.828
	I/R-MSCC	.249	6	.200*	.925	6	.542
	I/R-NC-MSCC	.262	6	.200*	.927	6	.560
MPO	Control	.105	6	.200*	1.000	6	1.000
	I/R	.152	6	.200*	.945	6	.698

Tests of Normality

	Groups	Kolmogorov-Smirnov[a]			Shapiro-Wilk		
		Statistic	df	Sig.	Statistic	df	Sig.
	I/R -NC	.176	6	.200*	.953	6	.768
	I/R-MSCs	.211	6	.200*	.944	6	.694
	I/R-MSCC	.261	6	.200*	.932	6	.597
	I/R-NC-MSCC	.209	6	.200*	.944	6	.690
TNF	Control	.274	6	.177	.845	6	.144
	I/R	.239	6	.200*	.928	6	.568
	I/R -NC	.362	6	.014	.735	6	.014
	I/R-MSCs	.265	6	.200*	.796	6	.054
	I/R-MSCC	.189	6	.200*	.930	6	.579
	I/R-NC-MSCC	.236	6	.200*	.913	6	.460
Bax	Control	.183	6	.200*	.960	6	.820
	I/R	.242	6	.200*	.857	6	.178
	I/R -NC	.230	6	.200*	.876	6	.253
	I/R-MSCs	.191	6	.200*	.939	6	.648
	I/R-MSCC	.183	6	.200*	.960	6	.820
	I/R-NC-MSCC	.181	6	.200*	.925	6	.541
BCL2	Control	.115	6	.200*	.998	6	1.000
	I/R	.248	6	.200*	.871	6	.230
	I/R -NC	.238	6	.200*	.945	6	.700
	I/R-MSCs	.167	6	.200*	.951	6	.746
	I/R-MSCC	.140	6	.200*	.978	6	.942
	I/R-NC-MSCC	.144	6	.200*	.976	6	.931
PI3K	Control	.205	6	.200*	.925	6	.540
	I/R	.223	6	.200*	.908	6	.421
	I/R -NC	.153	6	.200*	.957	6	.794
	I/R-MSCs	.122	6	.200*	.982	6	.961
	I/R-MSCC	.121	6	.200*	.983	6	.964
	I/R-NC-MSCC	.214	6	.200*	.958	6	.804
AKT	Control	.202	6	.200*	.853	6	.167
	I/R	.273	6	.185	.937	6	.633
	I/R -NC	.298	6	.102	.878	6	.260
	I/R-MSCs	.298	6	.102	.884	6	.290

Tests of Normality

	Groups	Kolmogorov-Smirnov[a] Statistic	df	Sig.	Shapiro-Wilk Statistic	df	Sig.
	I/R-MSCC	.355	6	.017	.809	6	.071
	I/R-NC-MSCC	.366	6	.012	.822	6	.092
mTOR	Control	.166	6	.200*	.935	6	.616
	I/R	.296	6	.110	.843	6	.138
	I/R -NC	.309	6	.076	.859	6	.186
	I/R-MSCs	.312	6	.069	.877	6	.254
	I/R-MSCC	.177	6	.200*	.939	6	.648
	I/R-NC-MSCC	.287	6	.133	.837	6	.123
caspaseexocrine	Control	.237	6	.200*	.926	6	.552
	I/R	.187	6	.200*	.952	6	.755
	I/R -NC	.210	6	.200*	.917	6	.487
	I/R-MSCs	.166	6	.200*	.941	6	.666
	I/R-MSCC	.122	6	.200*	.982	6	.961
	I/R-NC-MSCC	.164	6	.200*	.966	6	.866
caspaseendocrine	Control	.189	6	.200*	.953	6	.763
	I/R	.181	6	.200*	.935	6	.623
	I/R -NC	.166	6	.200*	.951	6	.751
	I/R-MSCs	.129	6	.200*	.991	6	.991
	I/R-MSCC	.122	6	.200*	.982	6	.961
	I/R-NC-MSCC	.172	6	.200*	.956	6	.788
antiinsulinantibody	Control	.228	6	.200*	.899	6	.367
	I/R	.190	6	.200*	.928	6	.568
	I/R -NC	.167	6	.200*	.934	6	.614
	I/R-MSCs	.161	6	.200*	.990	6	.988
	I/R-MSCC	.145	6	.200*	.979	6	.944
	I/R-NC-MSCC	.185	6	.200*	.948	6	.723
pancreasescore	Control	.492	6	.000	.496	6	.000
	I/R	.407	6	.002	.640	6	.001
	I/R -NC	.407	6	.002	.640	6	.001
	I/R-MSCs	.254	6	.200*	.866	6	.212
	I/R-MSCC	.407	6	.002	.640	6	.001
	I/R-NC-MSCC	.407	6	.002	.640	6	.001

*. This is a lower bound of the true significance.

a. Lilliefors Significance Correction

UREA

Normal Q-Q Plots

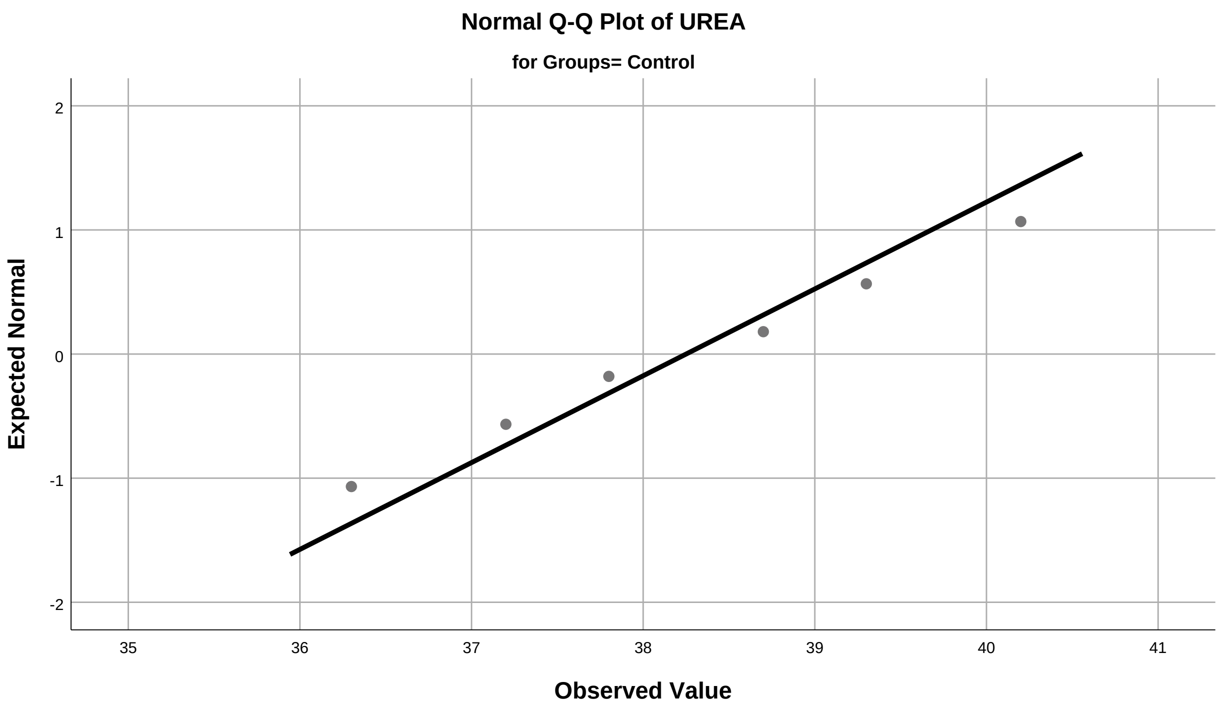

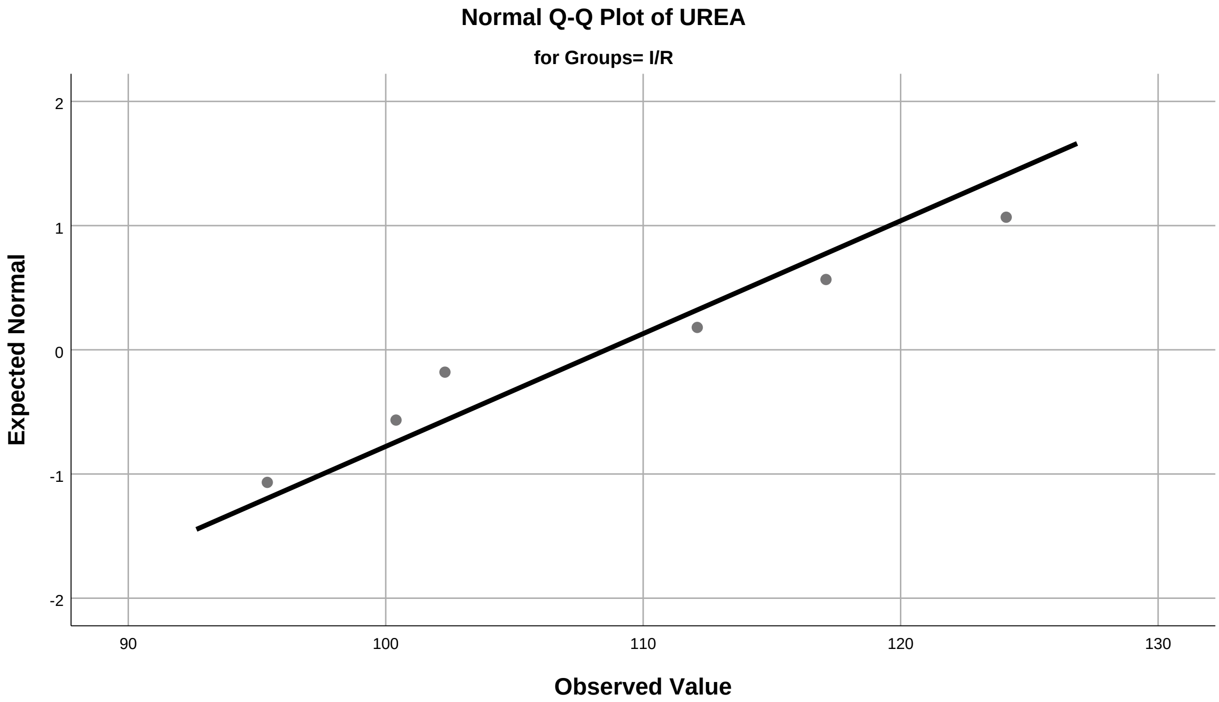
Normal Q-Q Plot of UREA
for Groups= I/R
Expected Normal
2
1
0
-1
-2
90
100
110
120
130
Observed Value

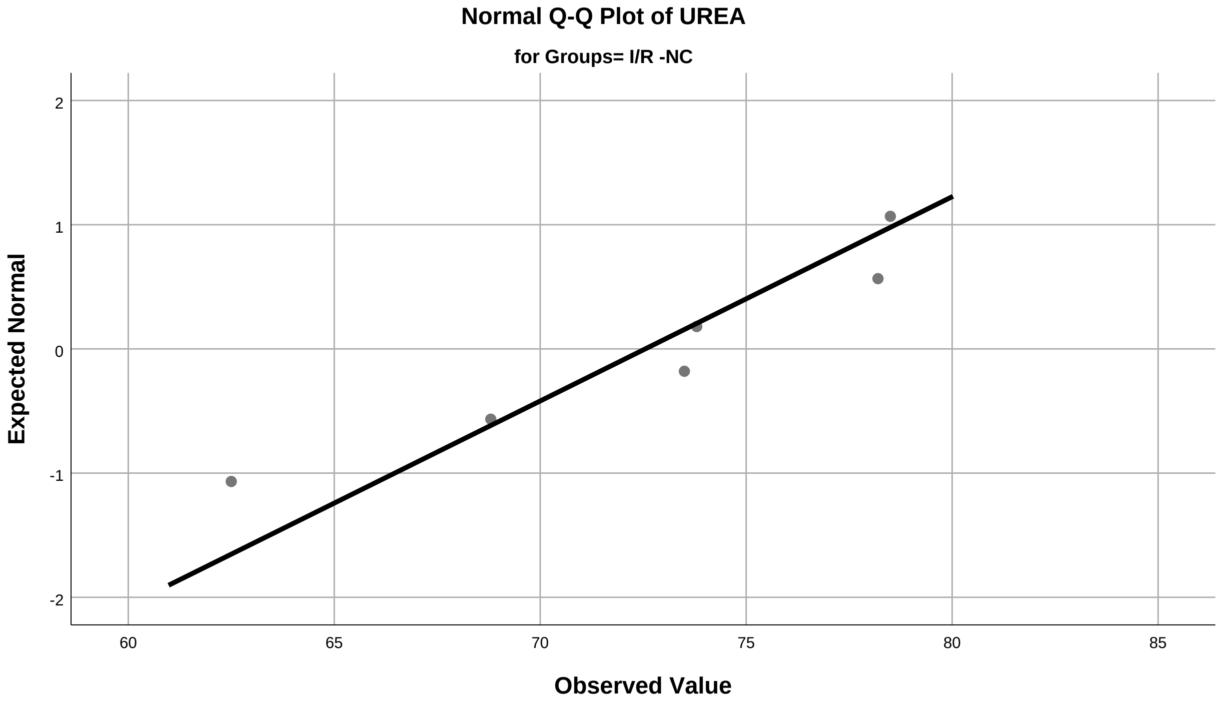
Normal Q-Q Plot of UREA
for Groups= I/R -NC
Expected Normal
2
1
0
-1
-2
60
65
70
75
80
85
Observed Value

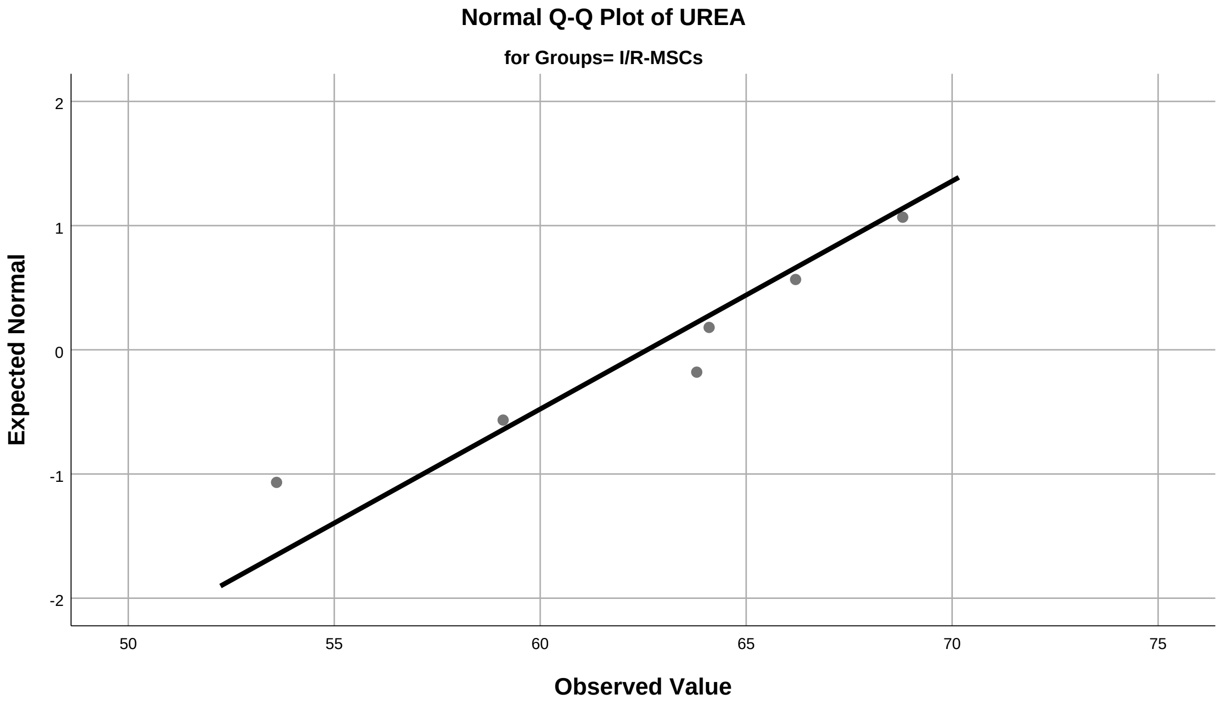
Normal Q-Q Plot of UREA
for Groups= I/R-MSCs
Expected Normal
2
1
0
-1
-2
50
55
60
65
70
75
Observed Value

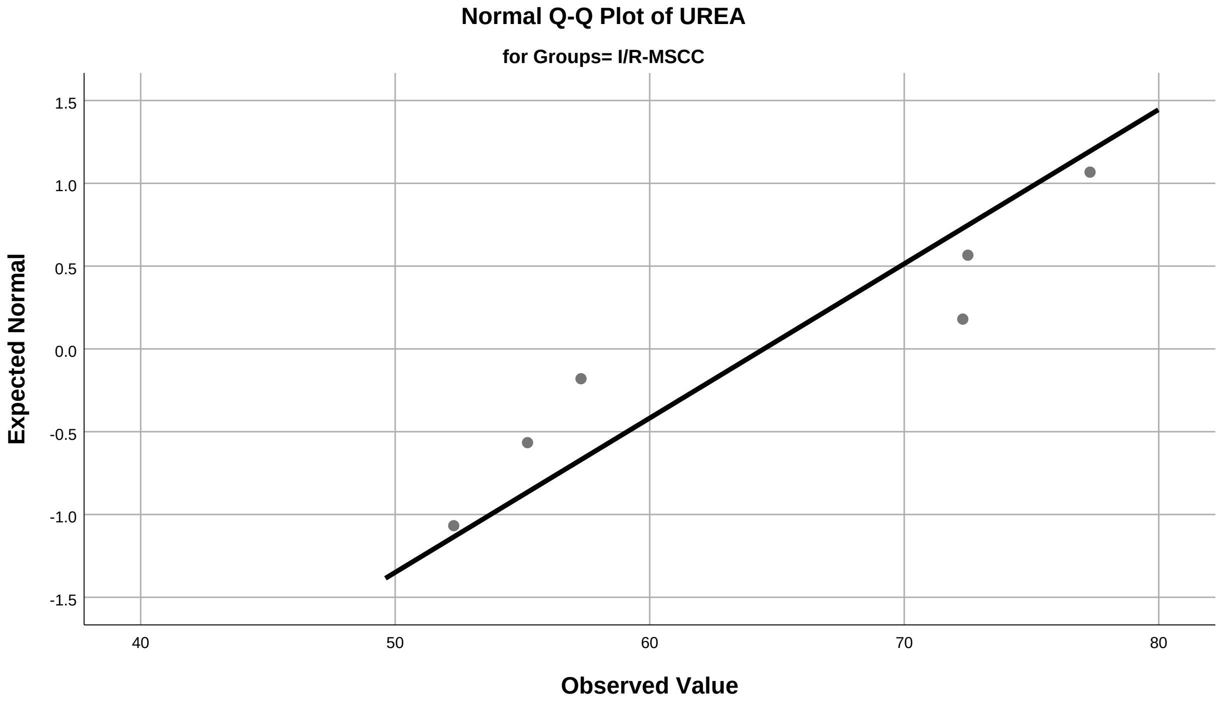
Normal Q-Q Plot of UREA
for Groups= I/R-MSCC
Expected Normal
1.5
1.0
0.5
0.0
-0.5
-1.0
-1.5
40
50
60
70
80
Observed Value

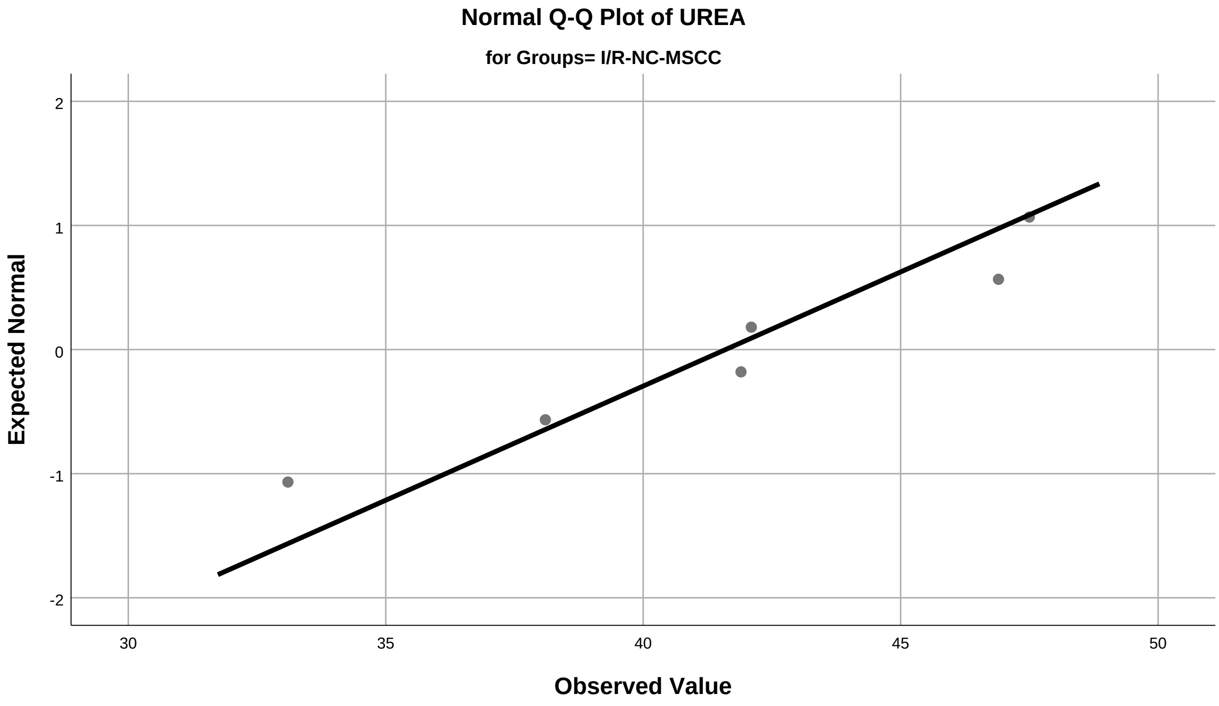

Detrended Normal Q-Q Plots

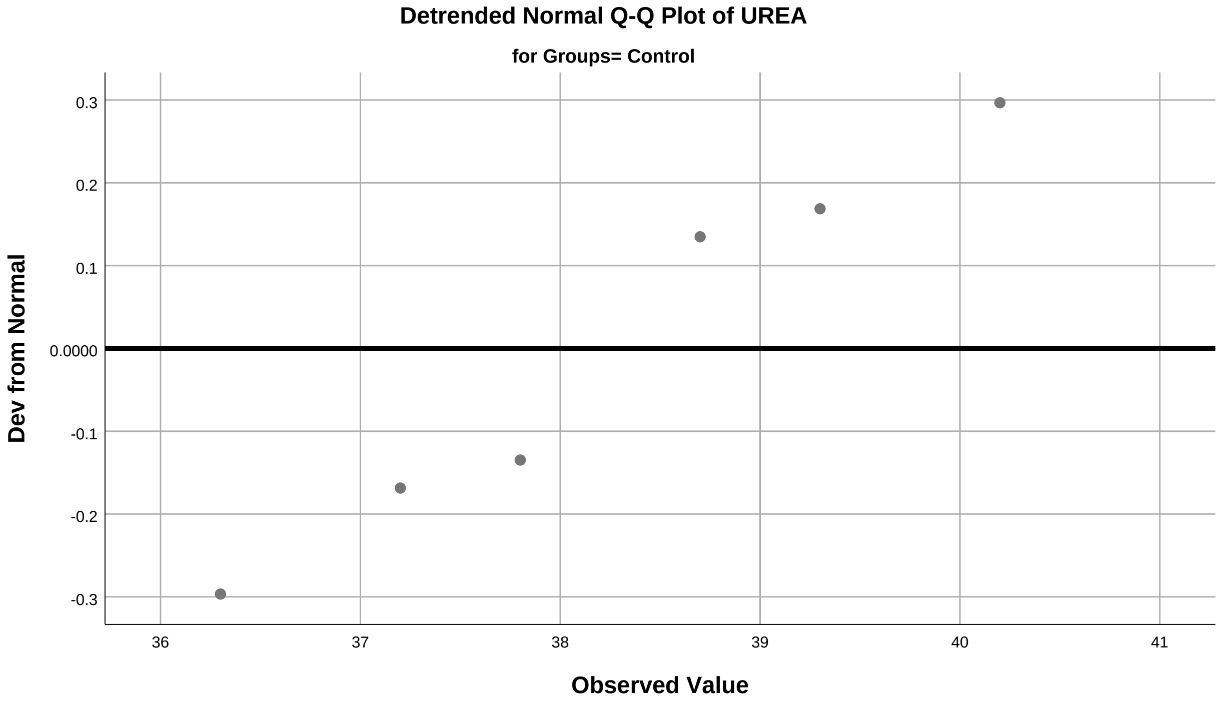

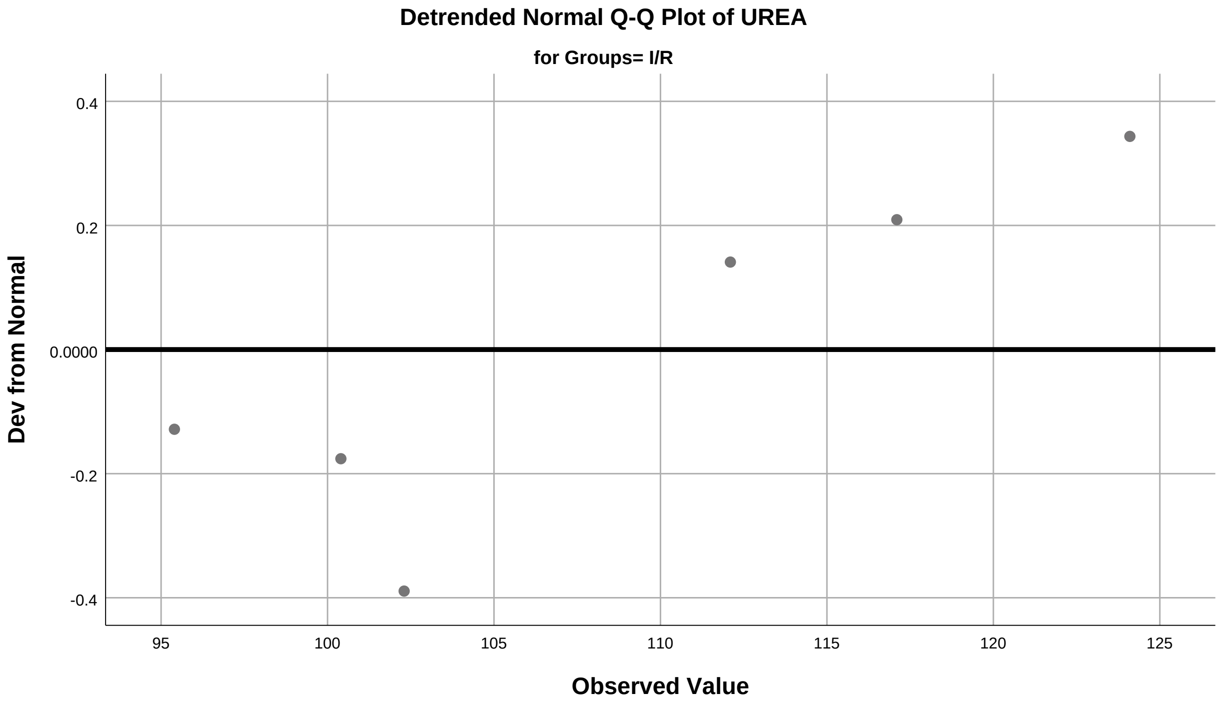
Detrended Normal Q-Q Plot of UREA
for Groups= I/R
Dev from Normal
0.4
0.2
0.0000
-0.2
-0.4
95
100
105
110
115
120
125
Observed Value

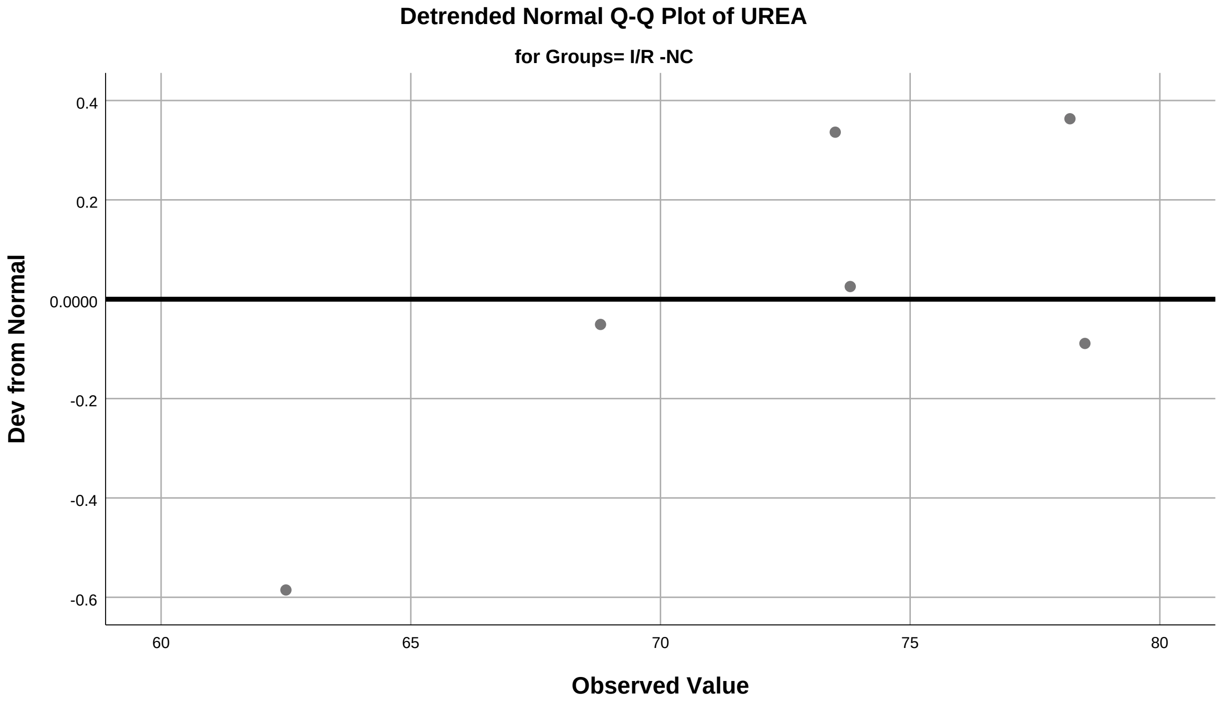
Detrended Normal Q-Q Plot of UREA
for Groups= I/R -NC
Dev from Normal
0.4
0.2
0.0000
-0.2
-0.4
-0.6
60
65
70
75
80
Observed Value

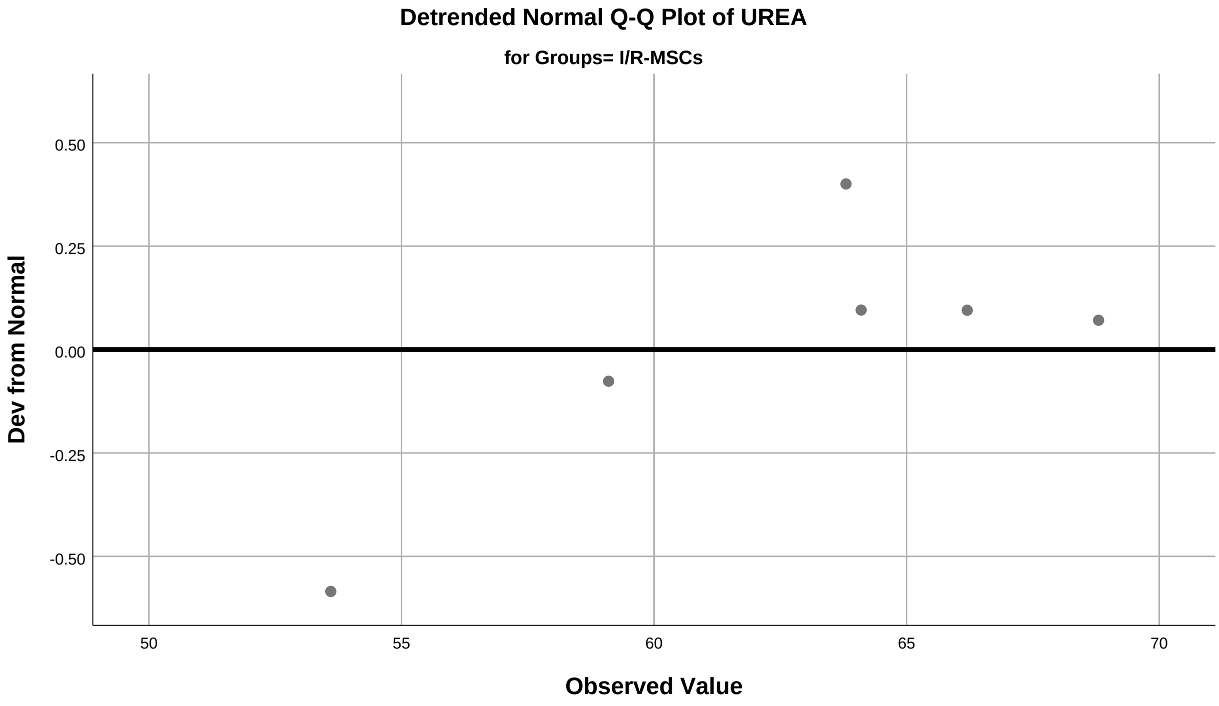
Detrended Normal Q-Q Plot of UREA
for Groups= I/R-MSCs
Dev from Normal
0.50
0.25
0.00
-0.25
-0.50
50
55
60
65
70
Observed Value

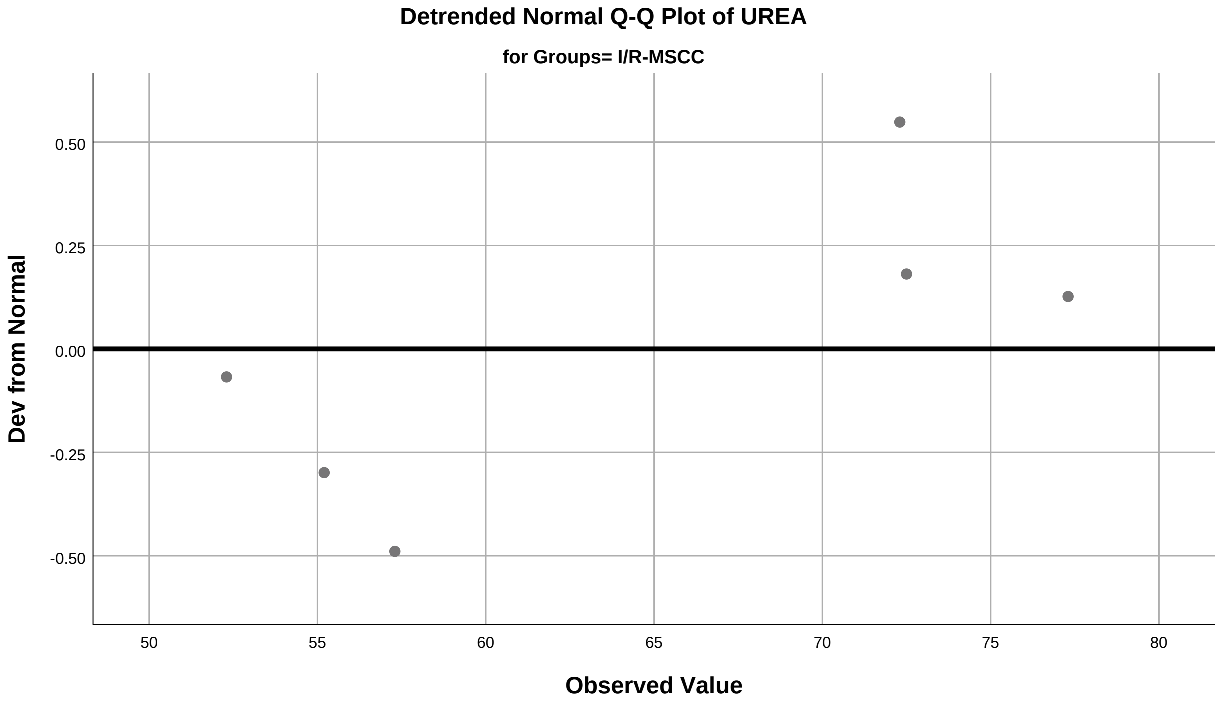
Detrended Normal Q-Q Plot of UREA
for Groups= I/R-MSCC
Dev from Normal
0.50
0.25
0.00
-0.25
-0.50
50
55
60
65
70
75
80
Observed Value

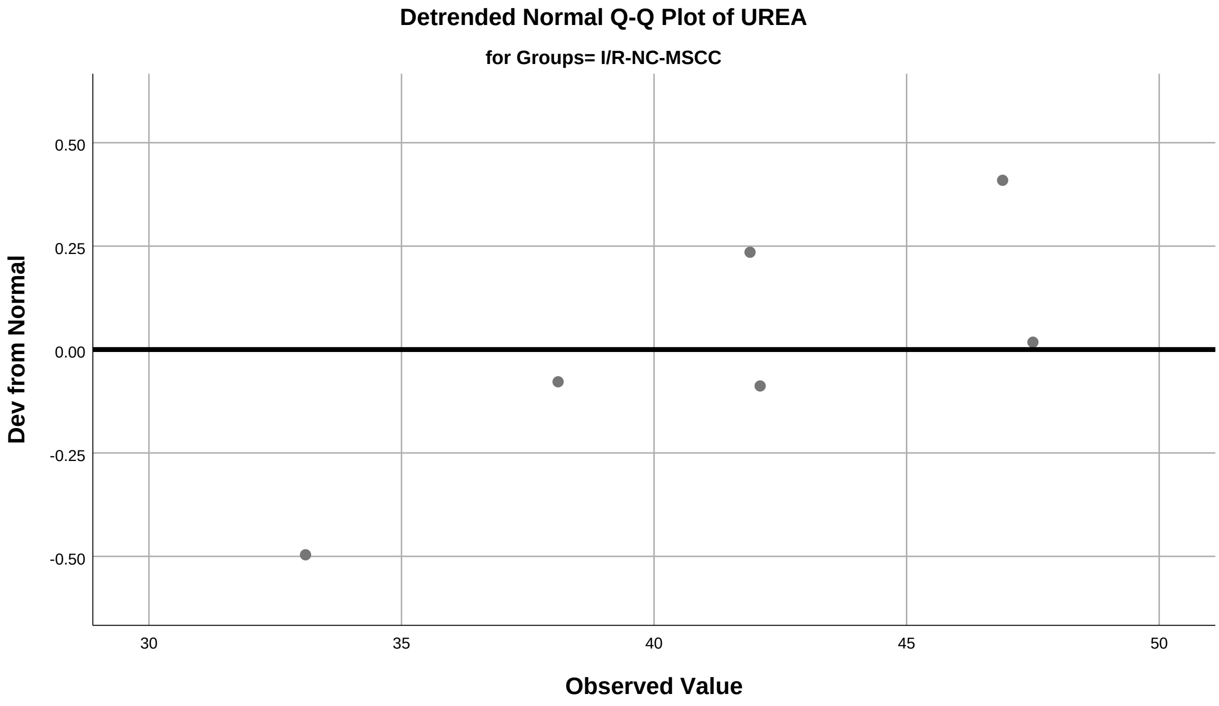

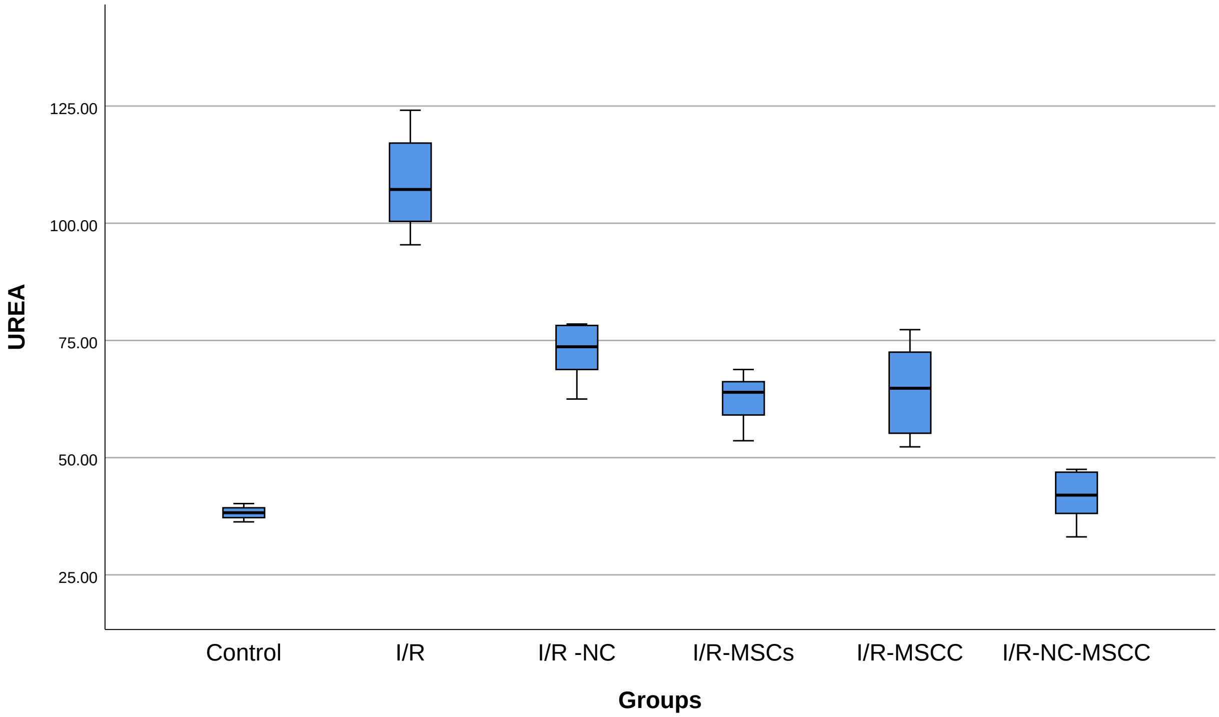

CREATININE

Normal Q-Q Plots

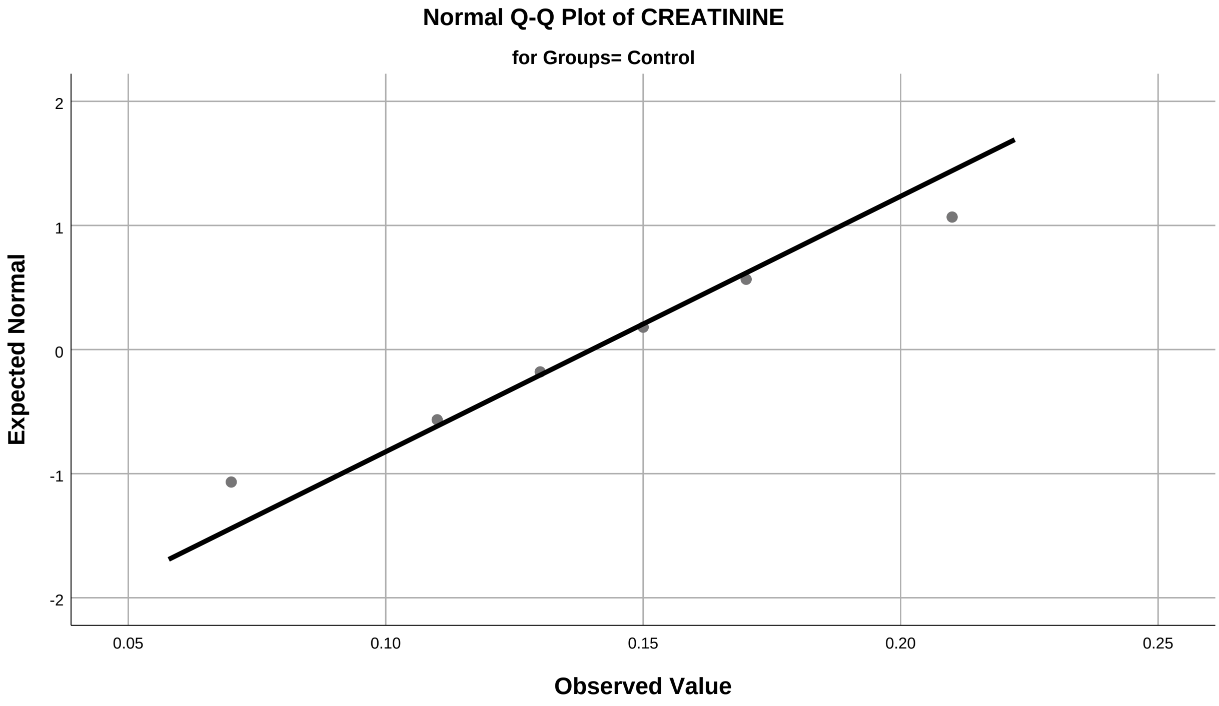
Normal Q-Q Plot of CREATININE
for Groups= Control
Expected Normal
2
1
0
-1
-2
0.05
0.10
0.15
0.20
0.25
Observed Value

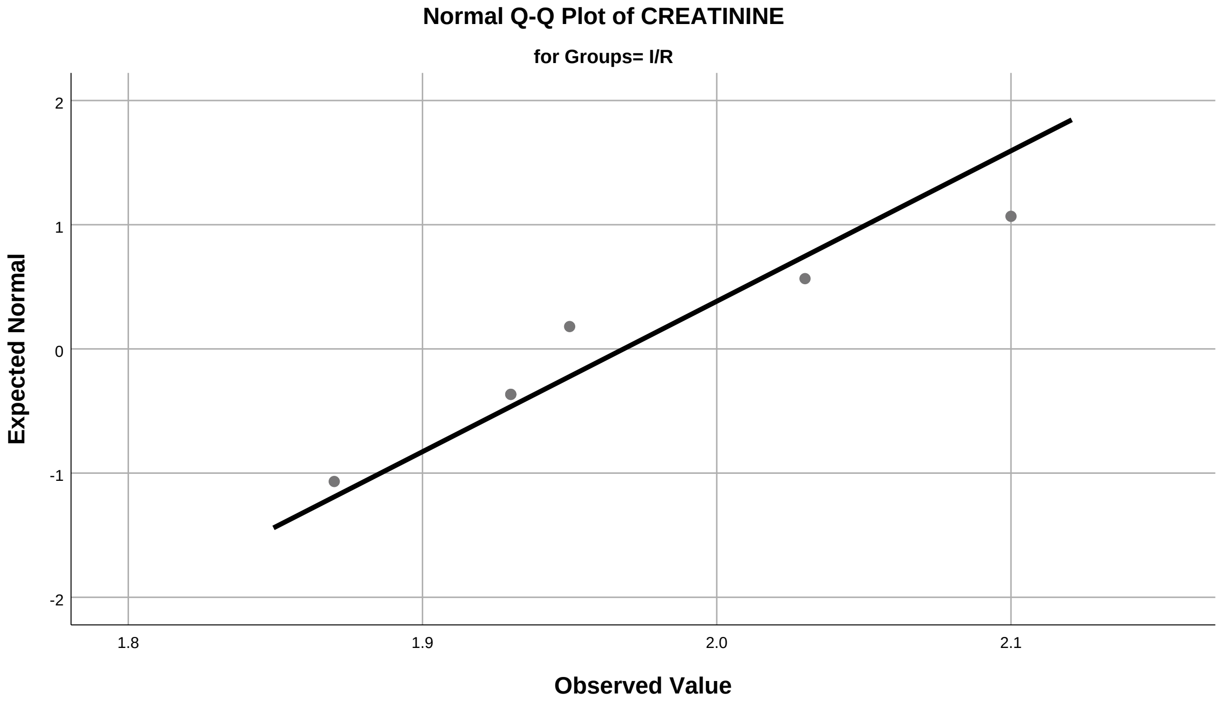
Normal Q-Q Plot of CREATININE
for Groups= I/R
Expected Normal
2
1
0
-1
-2
1.8
1.9
2.0
2.1
Observed Value

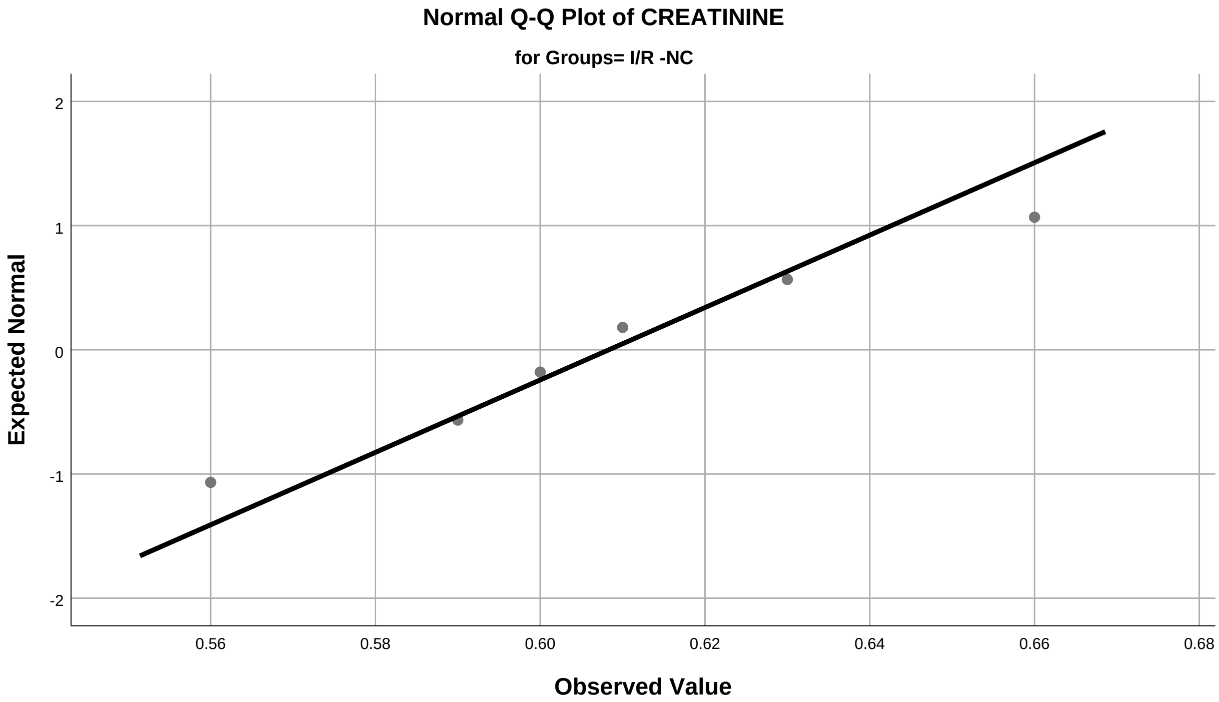
Normal Q-Q Plot of CREATININE
for Groups= I/R -NC
Expected Normal
2
1
0
-1
-2
0.56
0.58
0.60
0.62
0.64
0.66
0.68
Observed Value

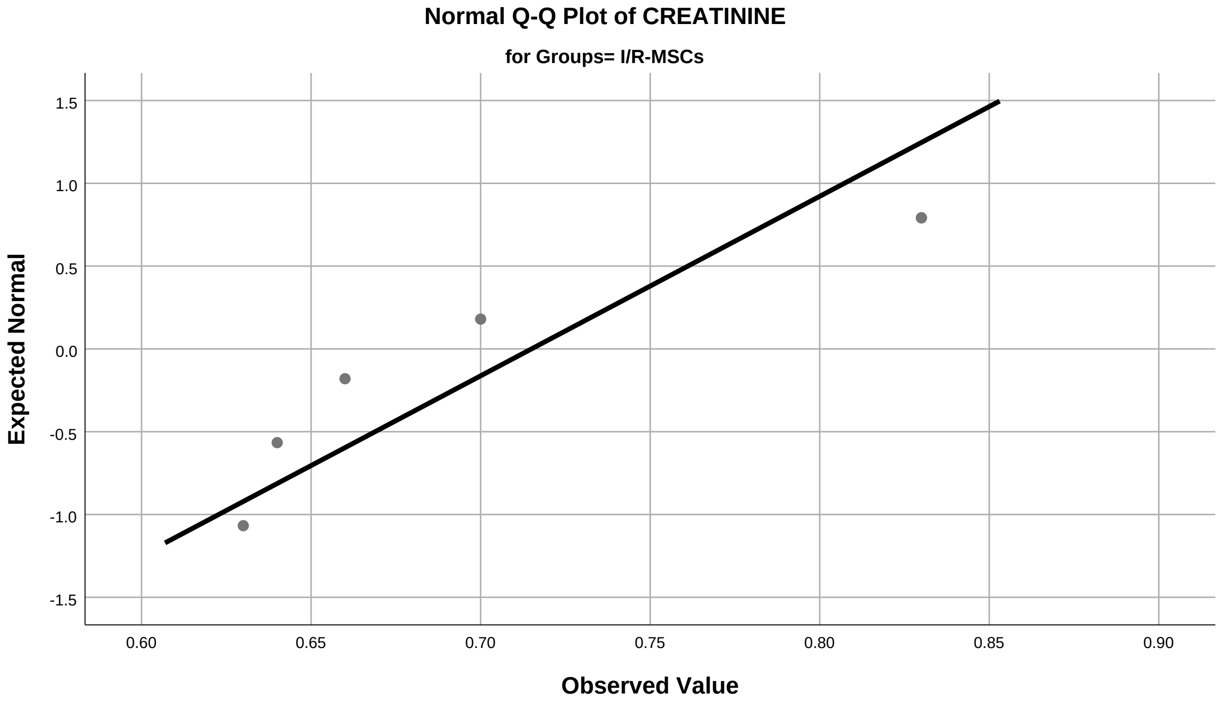
Normal Q-Q Plot of CREATININE
for Groups= I/R-MSCs
Expected Normal
1.5
1.0
0.5
0.0
-0.5
-1.0
-1.5
0.60
0.65
0.70
0.75
0.80
0.85
0.90
Observed Value

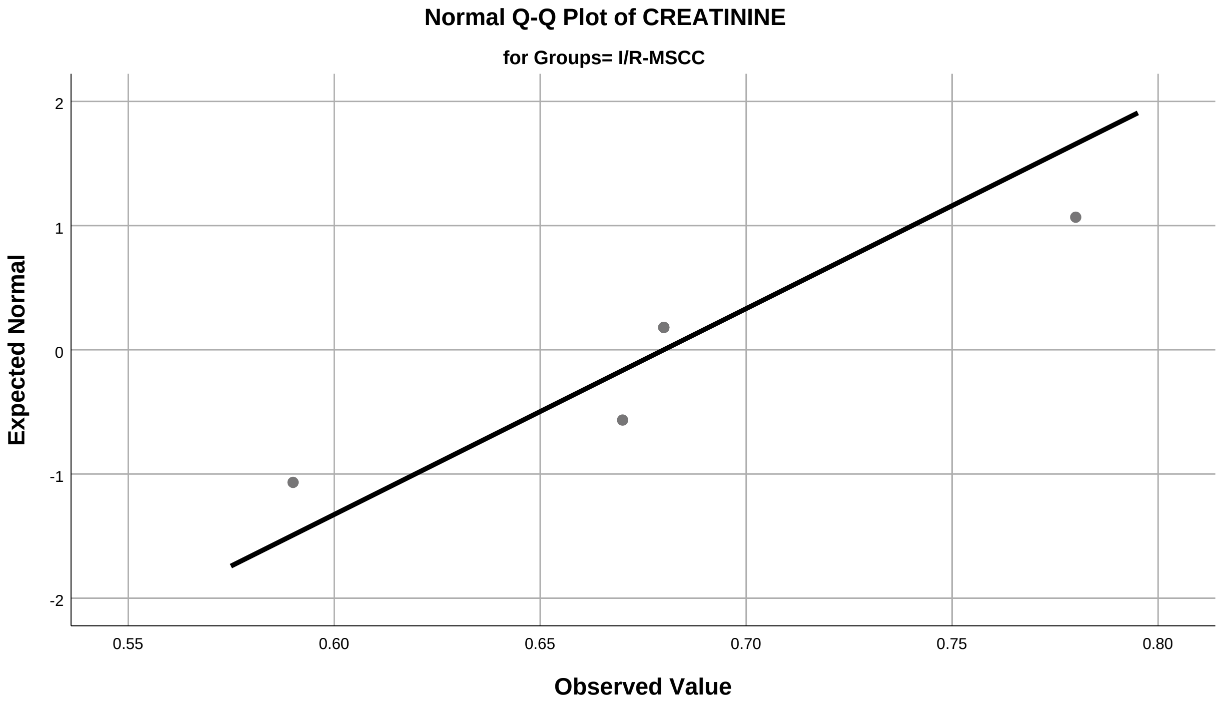

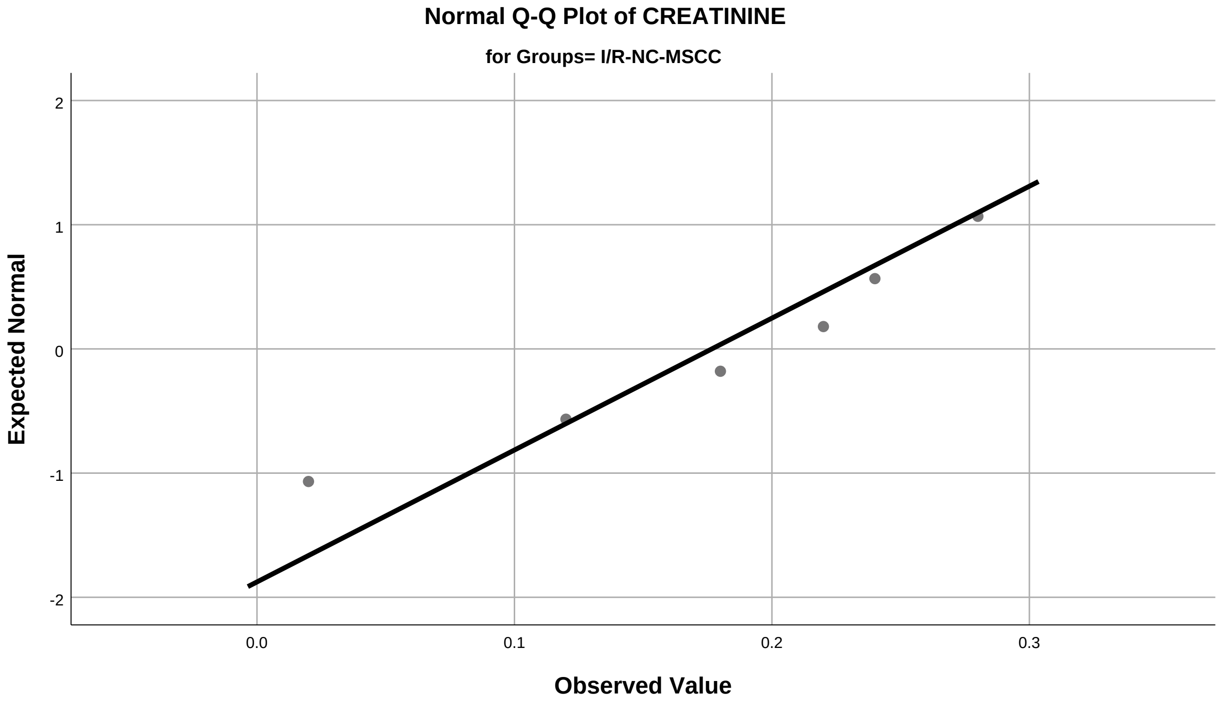

Detrended Normal Q-Q Plots

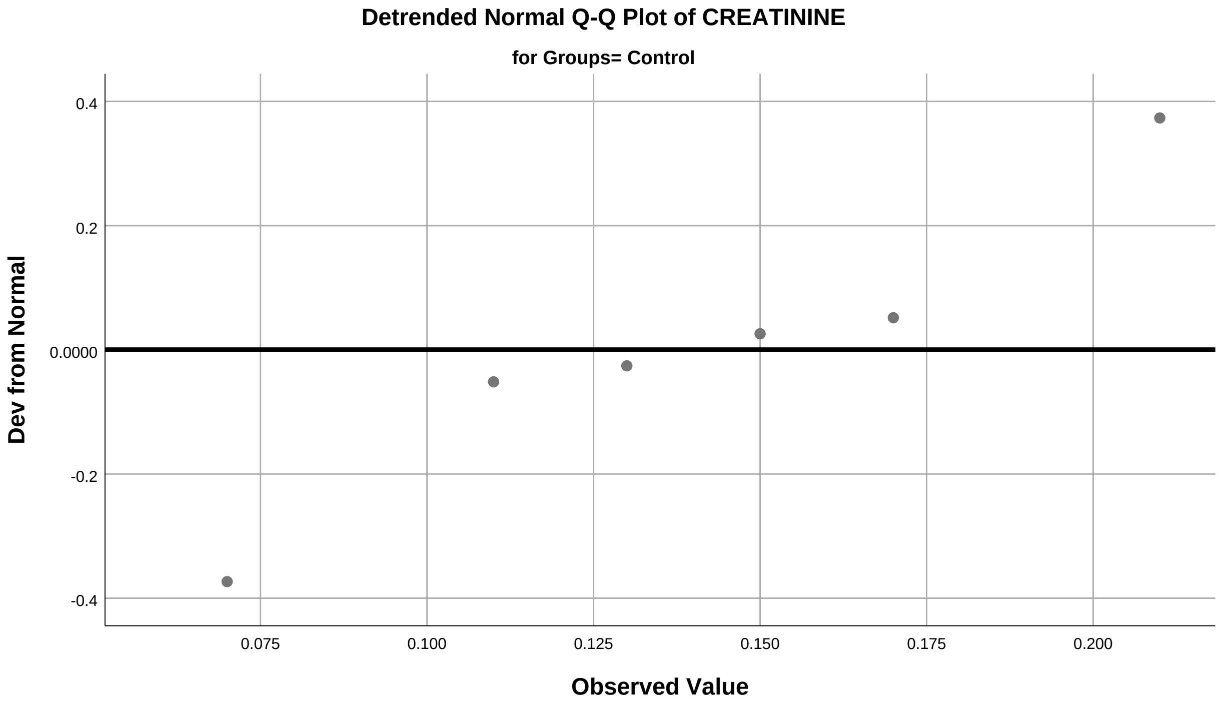
Detrended Normal Q-Q Plot of CREATININE
for Groups= Control
Dev from Normal
0.4
0.2
0.0000
-0.2
-0.4
0.075
0.100
0.125
0.150
0.175
0.200
Observed Value

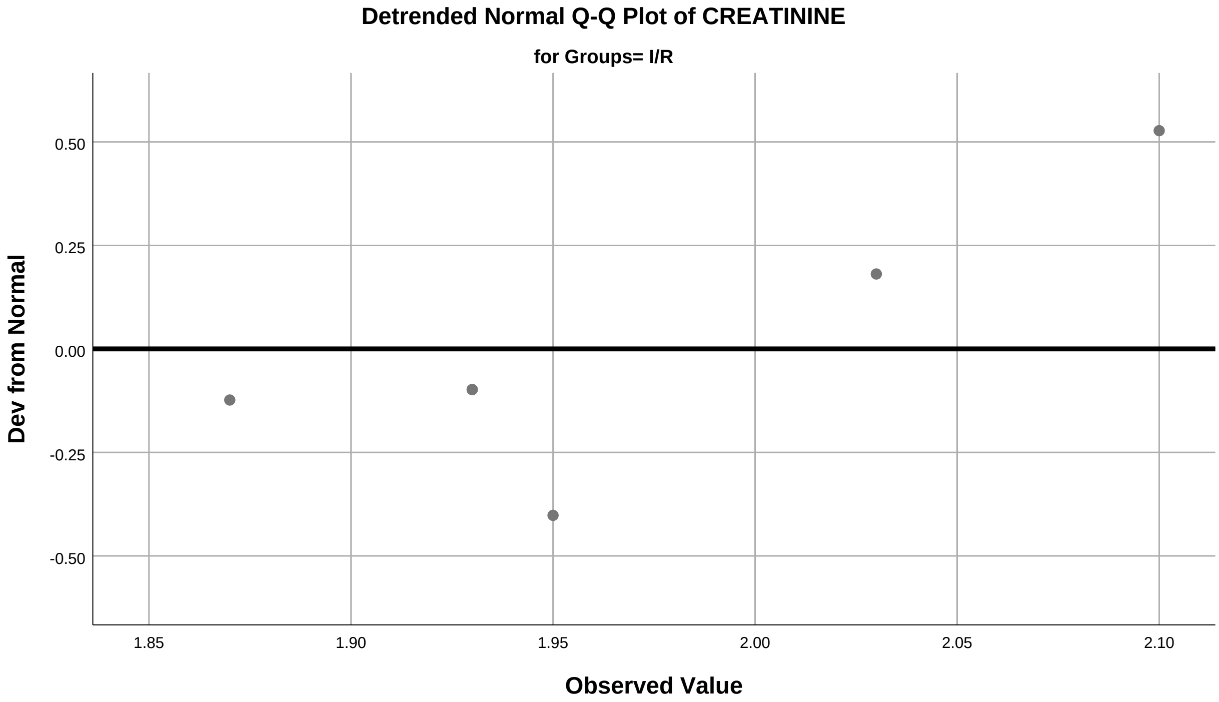
Detrended Normal Q-Q Plot of CREATININE
for Groups= I/R
Dev from Normal
0.50
0.25
0.00
-0.25
-0.50
1.85
1.90
1.95
2.00
2.05
2.10
Observed Value

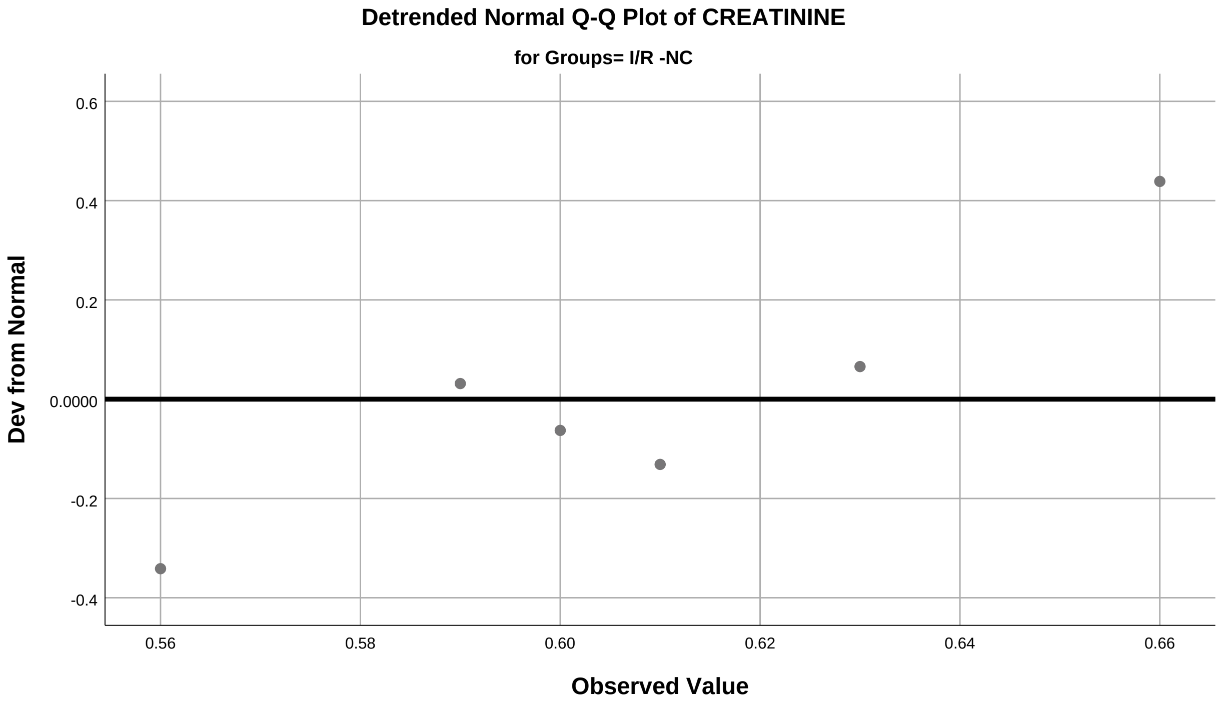
Detrended Normal Q-Q Plot of CREATININE
for Groups= I/R -NC
Dev from Normal
0.6
0.4
0.2
0.0000
-0.2
-0.4
0.56
0.58
0.60
0.62
0.64
0.66
Observed Value

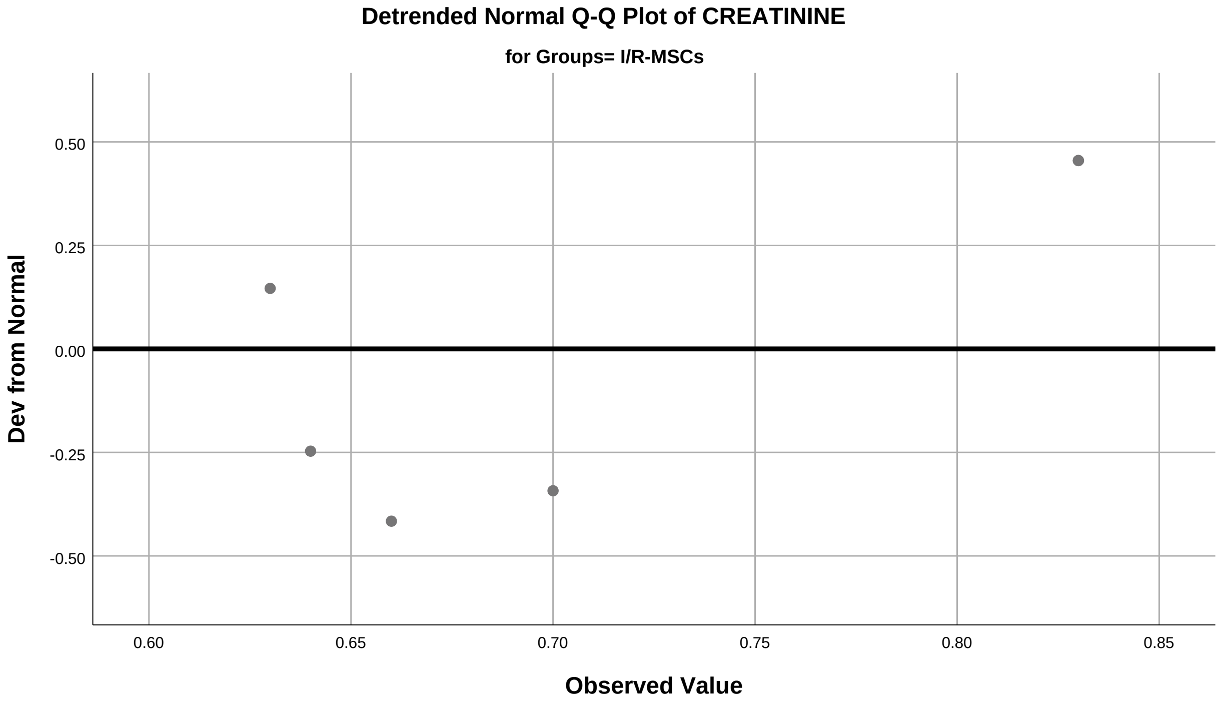
Detrended Normal Q-Q Plot of CREATININE
for Groups= I/R-MSCs
Dev from Normal
0.50
0.25
0.00
-0.25
-0.50
0.60
0.65
0.70
0.75
0.80
0.85
Observed Value

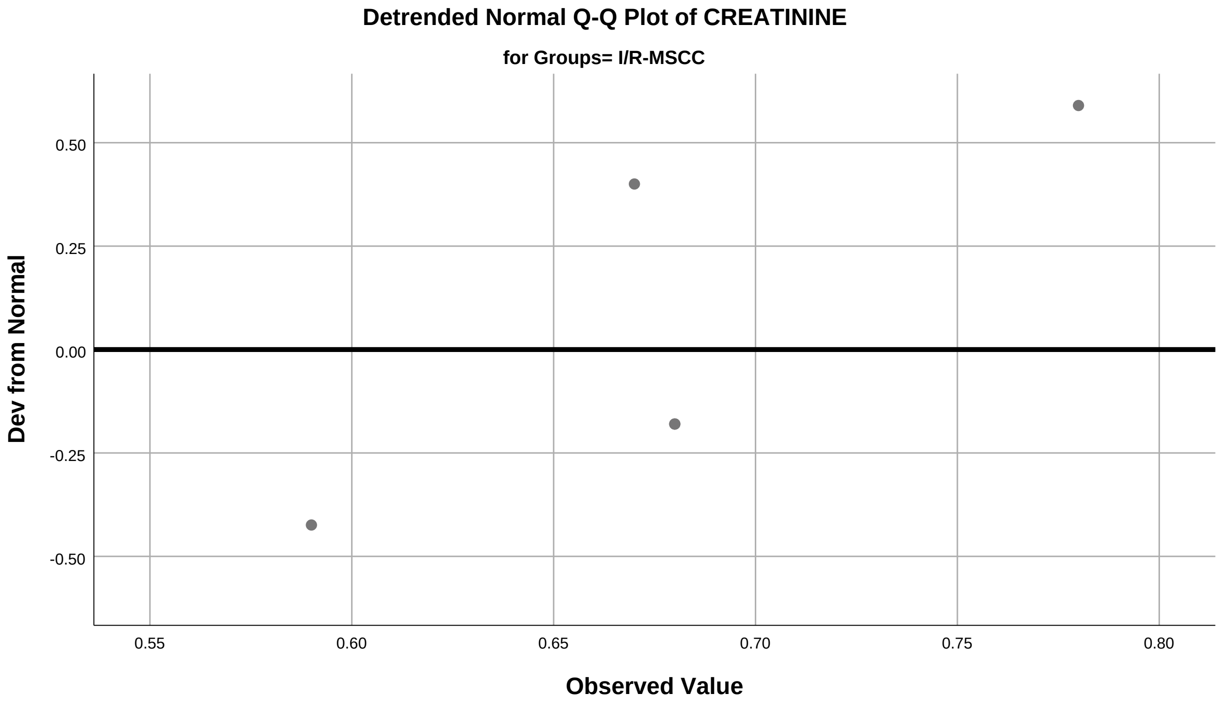
Detrended Normal Q-Q Plot of CREATININE
for Groups= I/R-MSCC
Dev from Normal
0.50
0.25
0.00
-0.25
-0.50
0.55
0.60
0.65
0.70
0.75
0.80
Observed Value

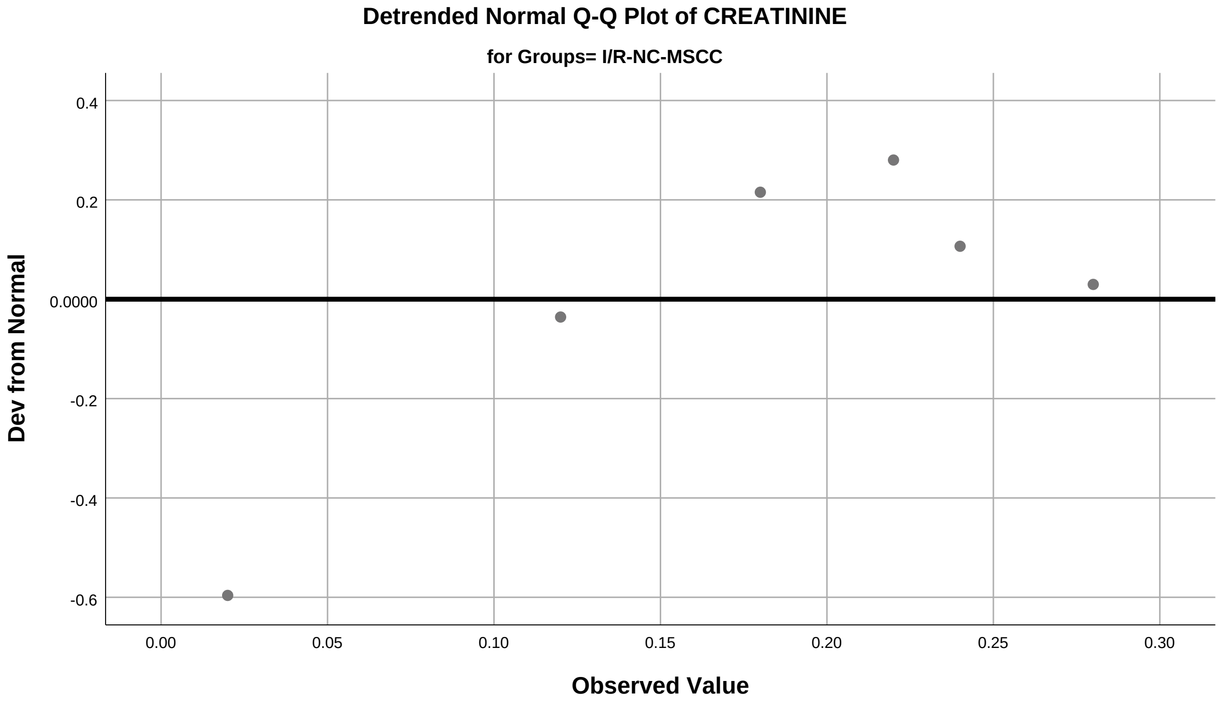
Detrended Normal Q-Q Plot of CREATININE
for Groups= I/R-NC-MSCC
Dev from Normal
0.4
0.2
0.0000
-0.2
-0.4
-0.6
0.00
0.05
0.10
0.15
0.20
0.25
0.30
Observed Value

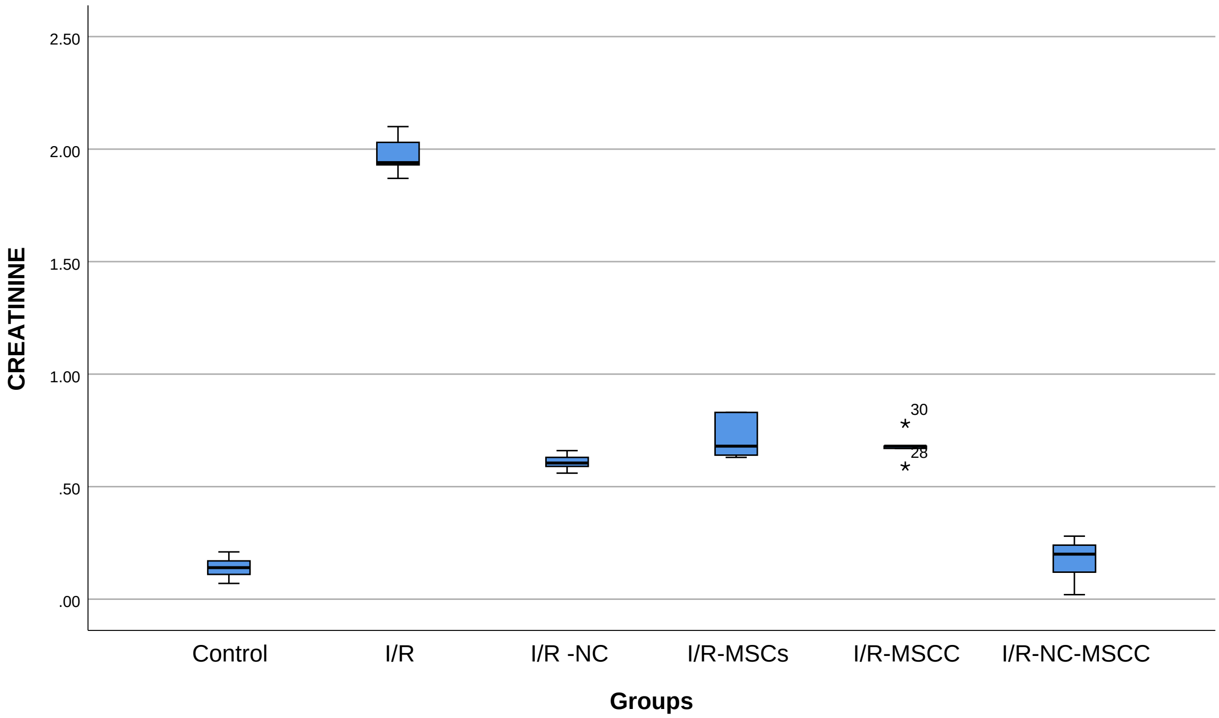

GLUCOSE

Normal Q-Q Plots

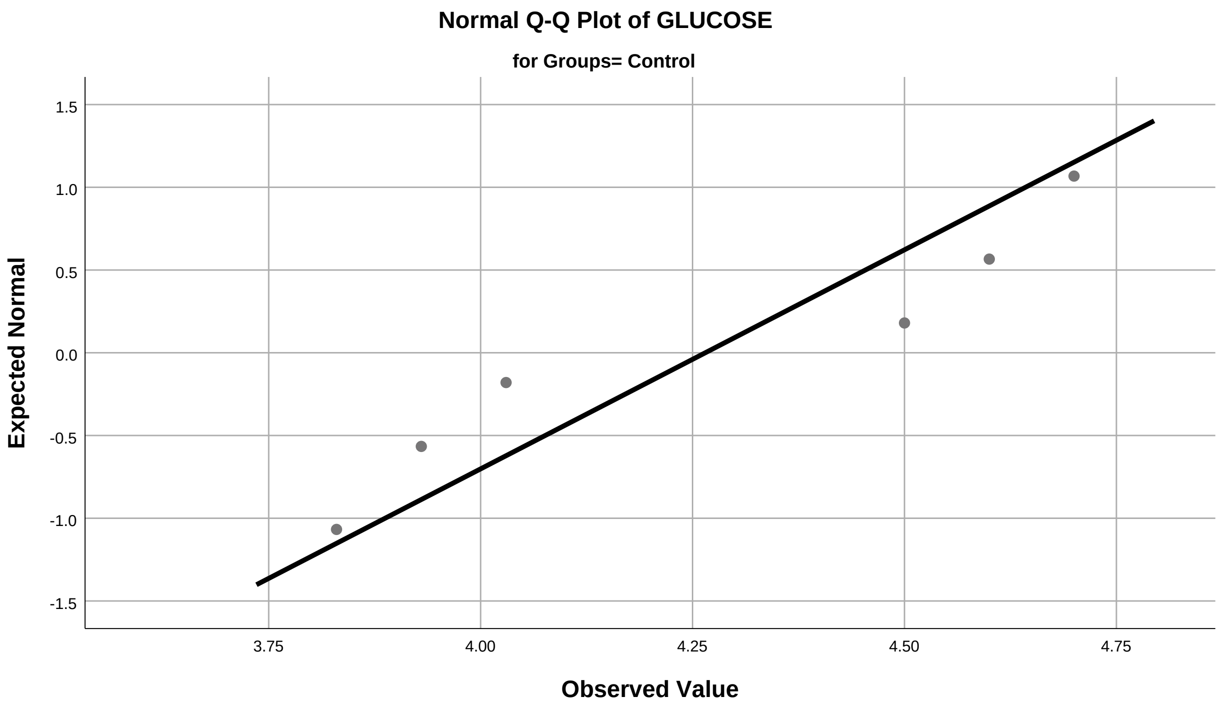

Normal Q-Q Plot of GLUCOSE

for Groups= I/R

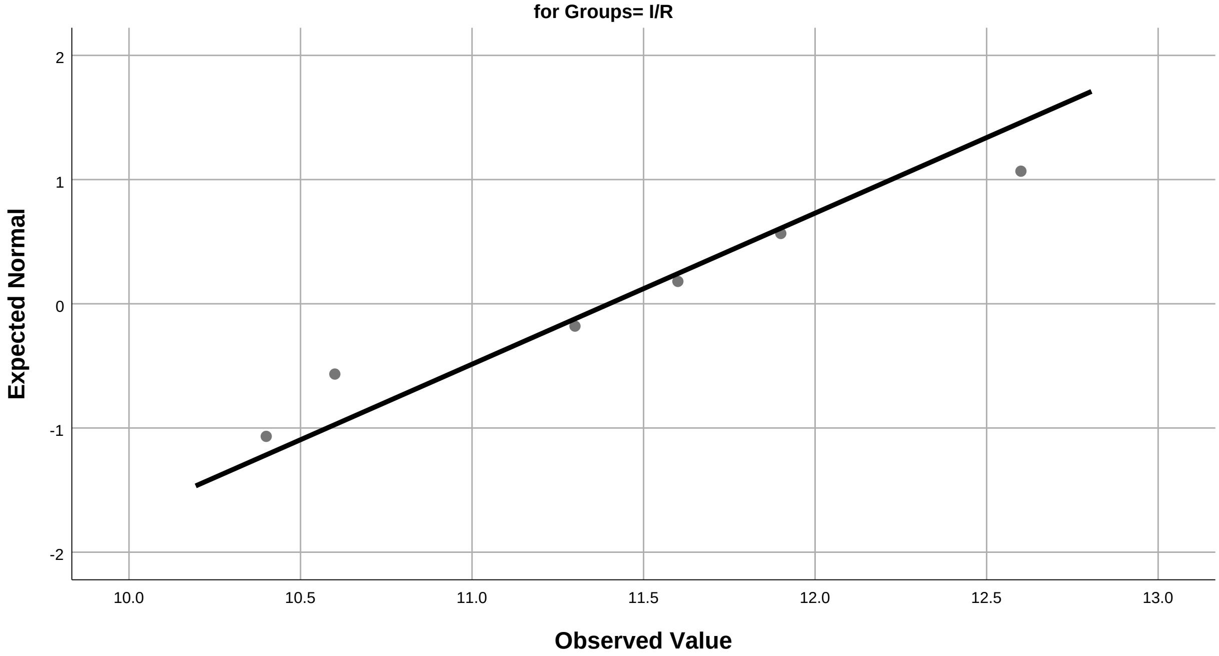

Normal Q-Q Plot of GLUCOSE

for Groups= I/R -NC

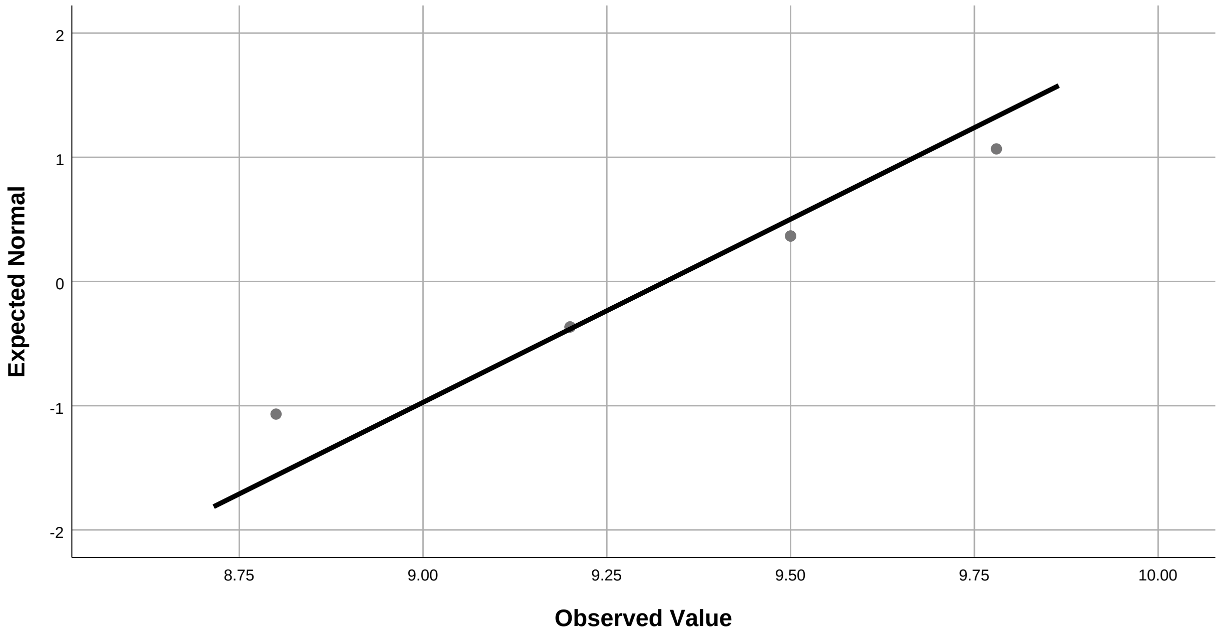

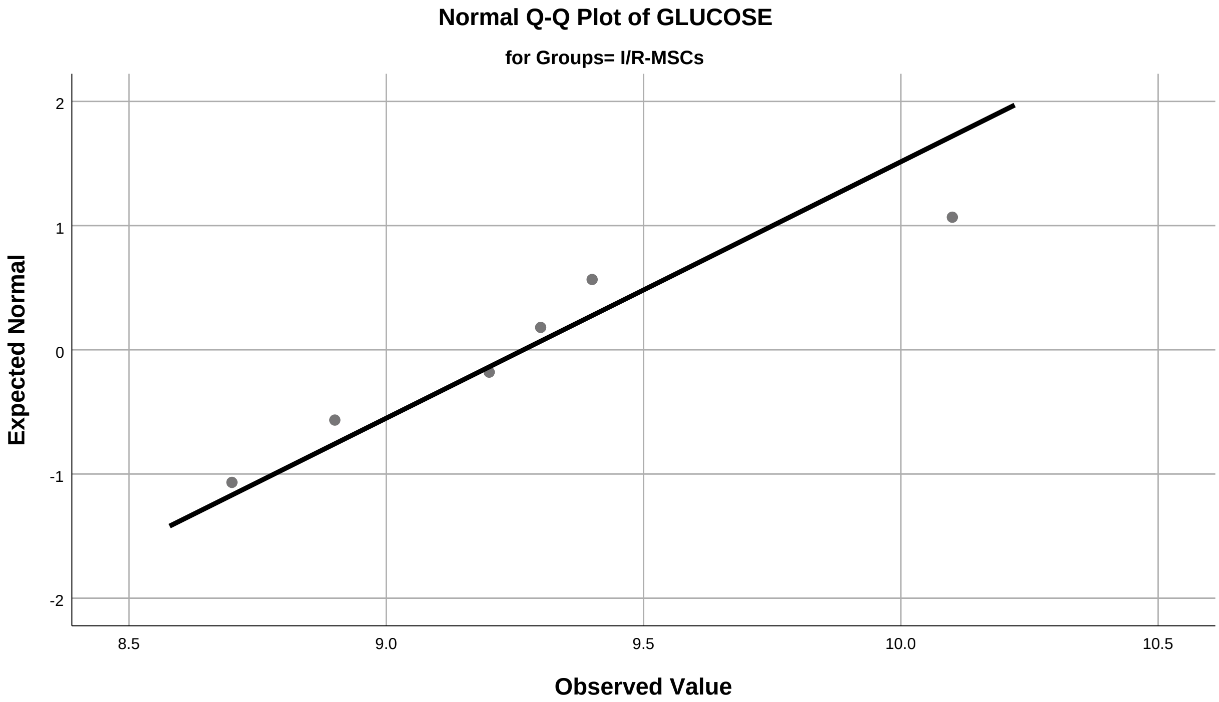
Normal Q-Q Plot of GLUCOSE
for Groups= I/R-MSCs
Expected Normal
2
1
0
-1
-2
8.5
9.0
9.5
10.0
10.5
Observed Value

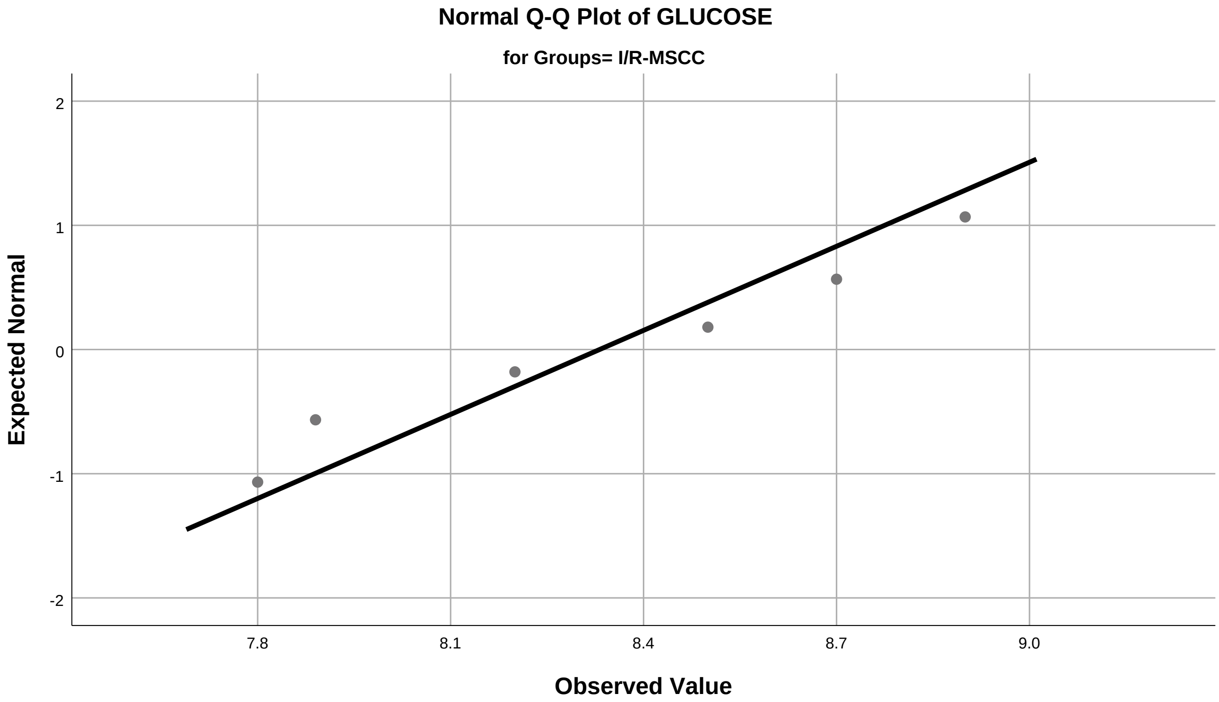
Normal Q-Q Plot of GLUCOSE
for Groups= I/R-MSCC
Expected Normal
2
1
0
-1
-2
7.8
8.1
8.4
8.7
9.0
Observed Value

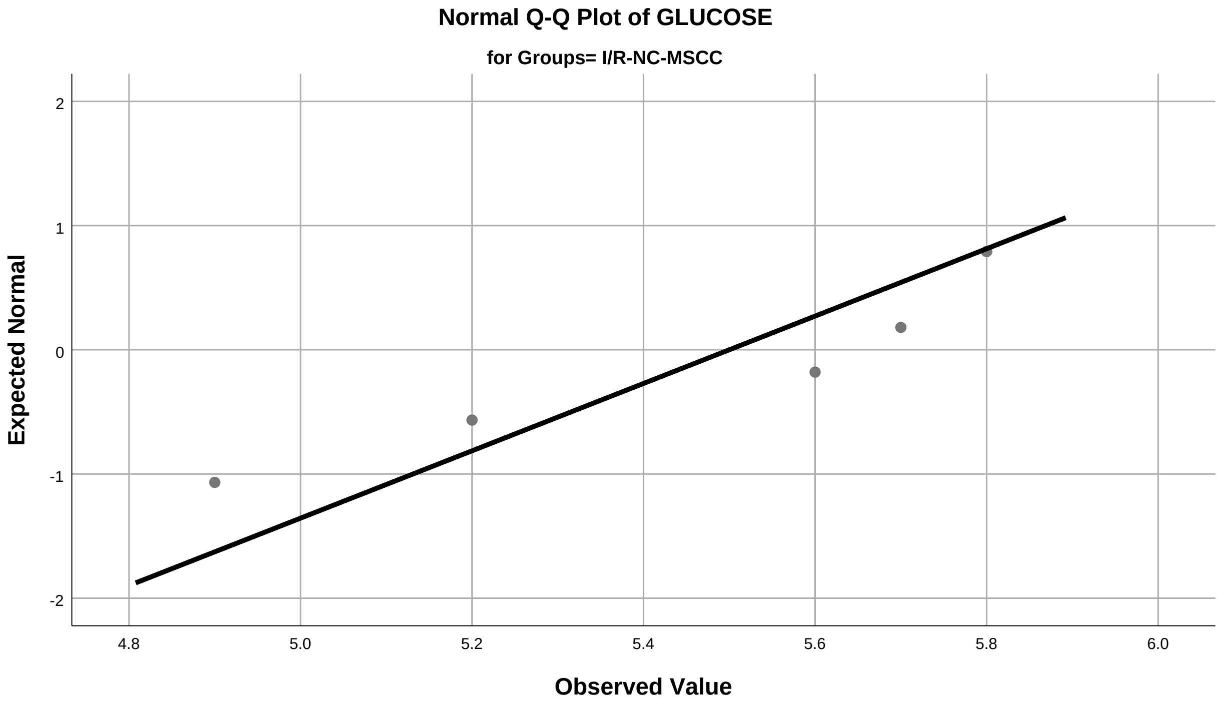

Detrended Normal Q-Q Plots

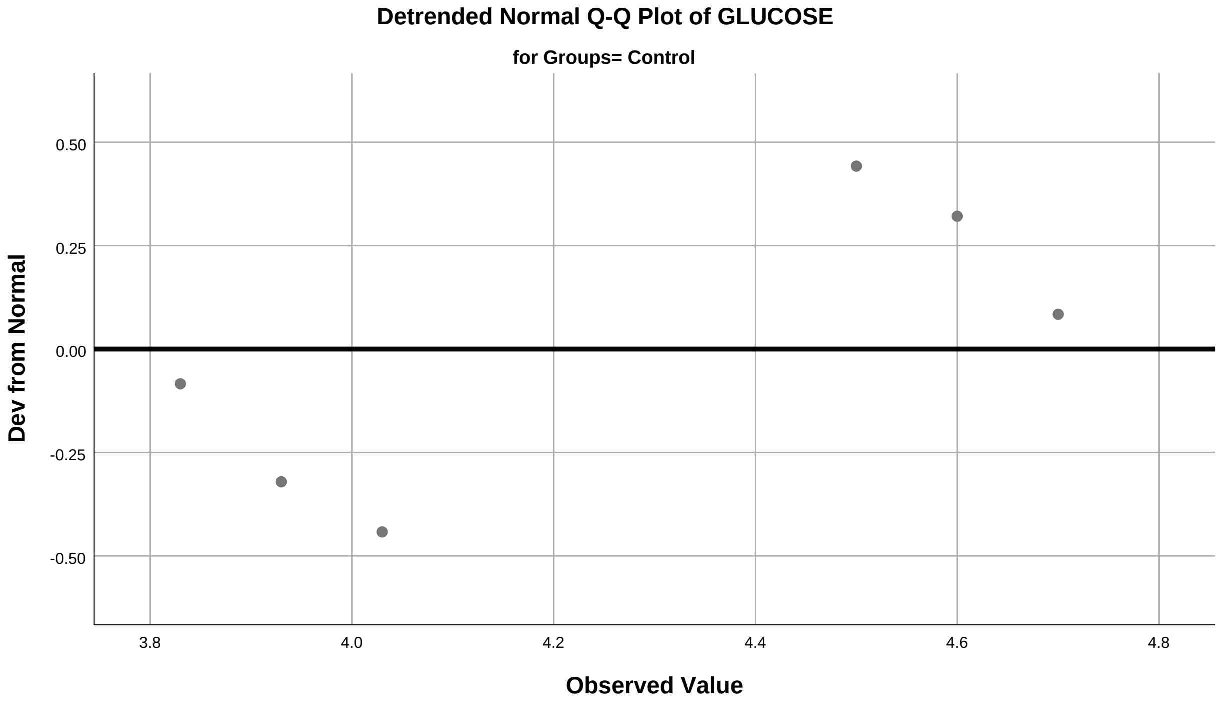

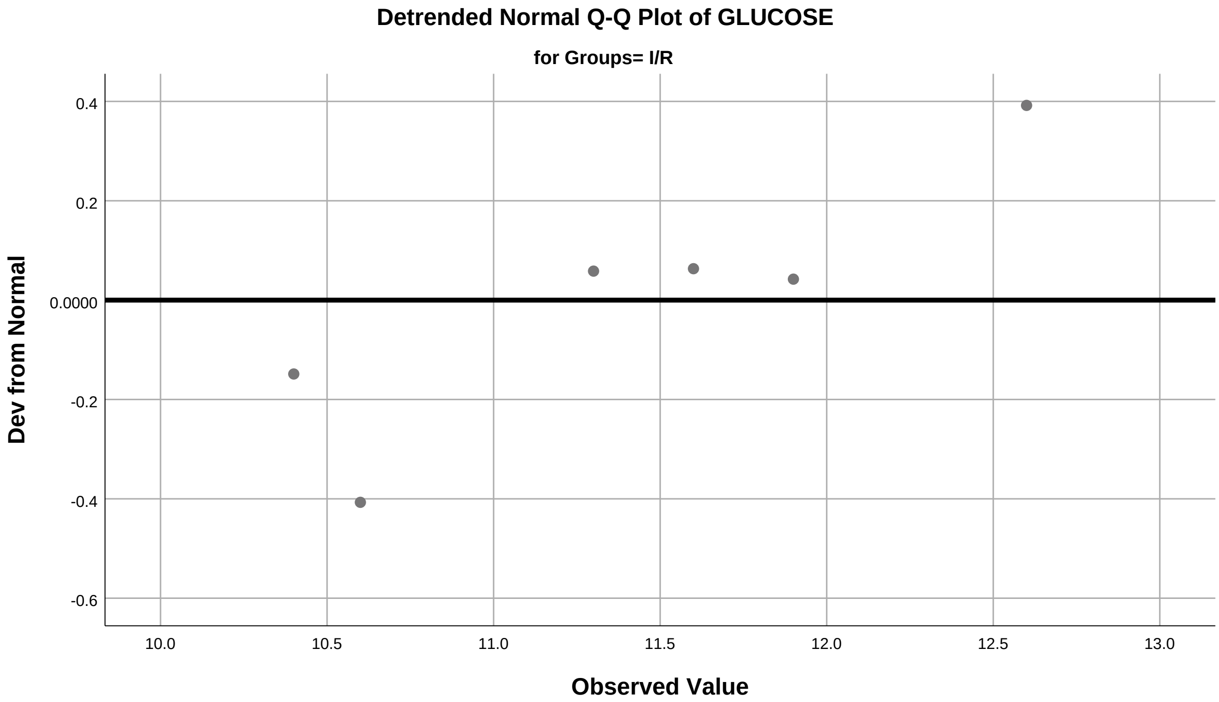

Detrended Normal Q-Q Plot of GLUCOSE
for Groups= I/R
Dev from Normal
0.4
0.2
0.0000
-0.2
-0.4
-0.6
10.0
10.5
11.0
11.5
12.0
12.5
13.0
Observed Value

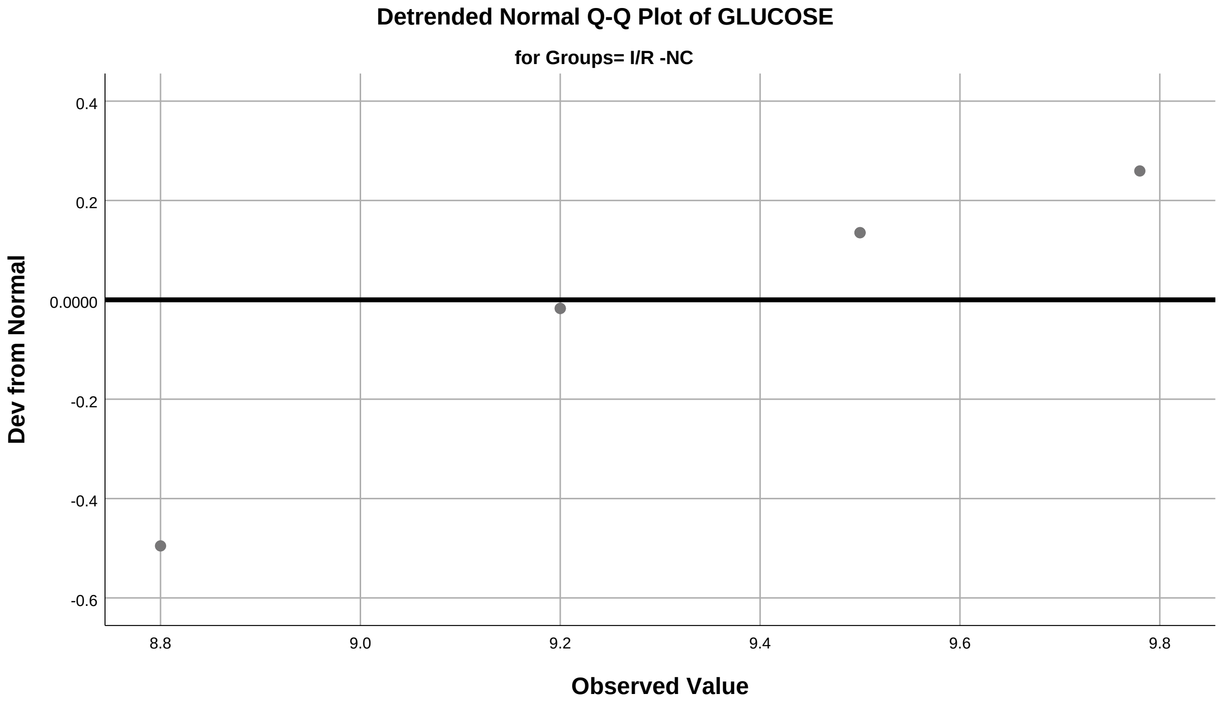

Detrended Normal Q-Q Plot of GLUCOSE
for Groups= I/R -NC
Dev from Normal
0.4
0.2
0.0000
-0.2
-0.4
-0.6
8.8
9.0
9.2
9.4
9.6
9.8
Observed Value

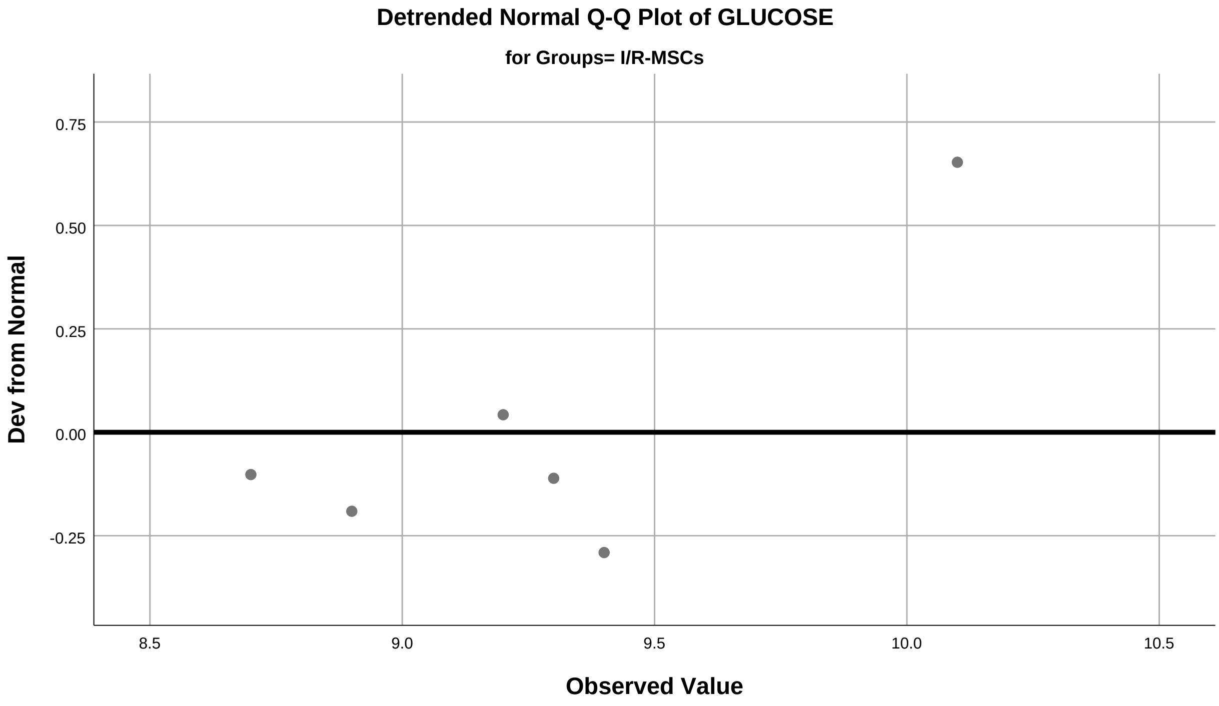

Detrended Normal Q-Q Plot of GLUCOSE
for Groups= I/R-MSCs
Dev from Normal
0.75
0.50
0.25
0.00
-0.25
8.5
9.0
9.5
10.0
10.5
Observed Value

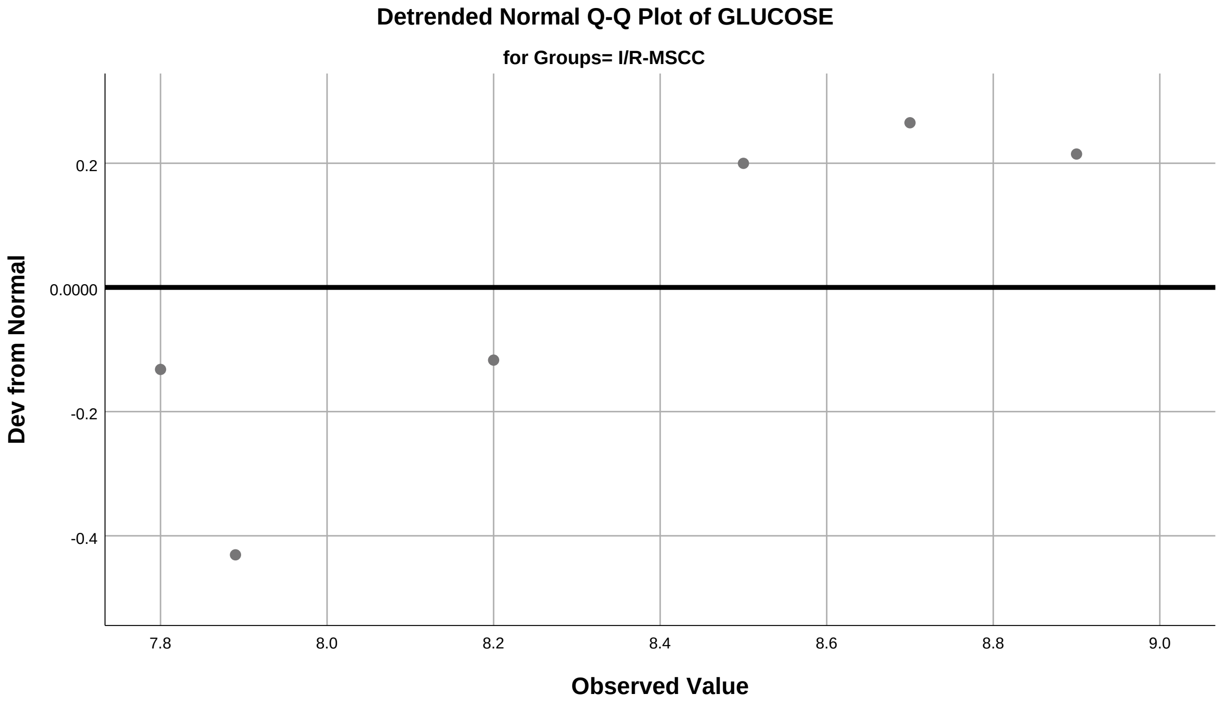

Detrended Normal Q-Q Plot of GLUCOSE
for Groups= I/R-MSCC
Dev from Normal
0.2
0.0000
-0.2
-0.4
7.8
8.0
8.2
8.4
8.6
8.8
9.0
Observed Value

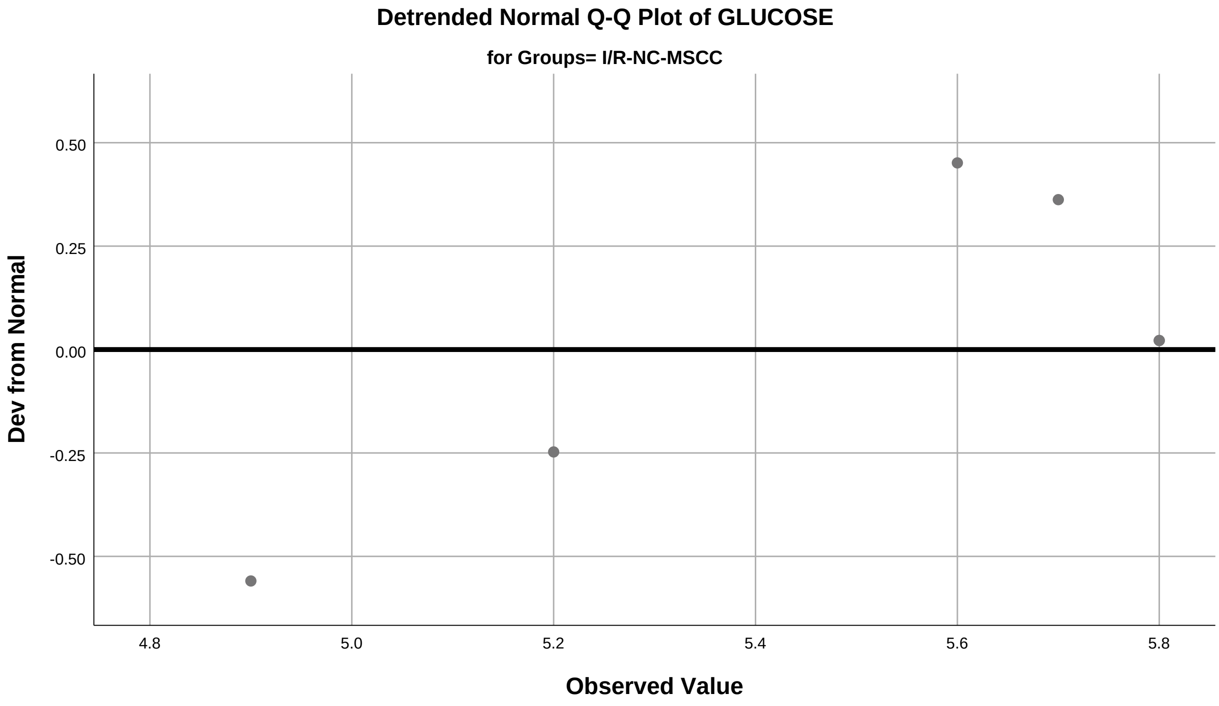

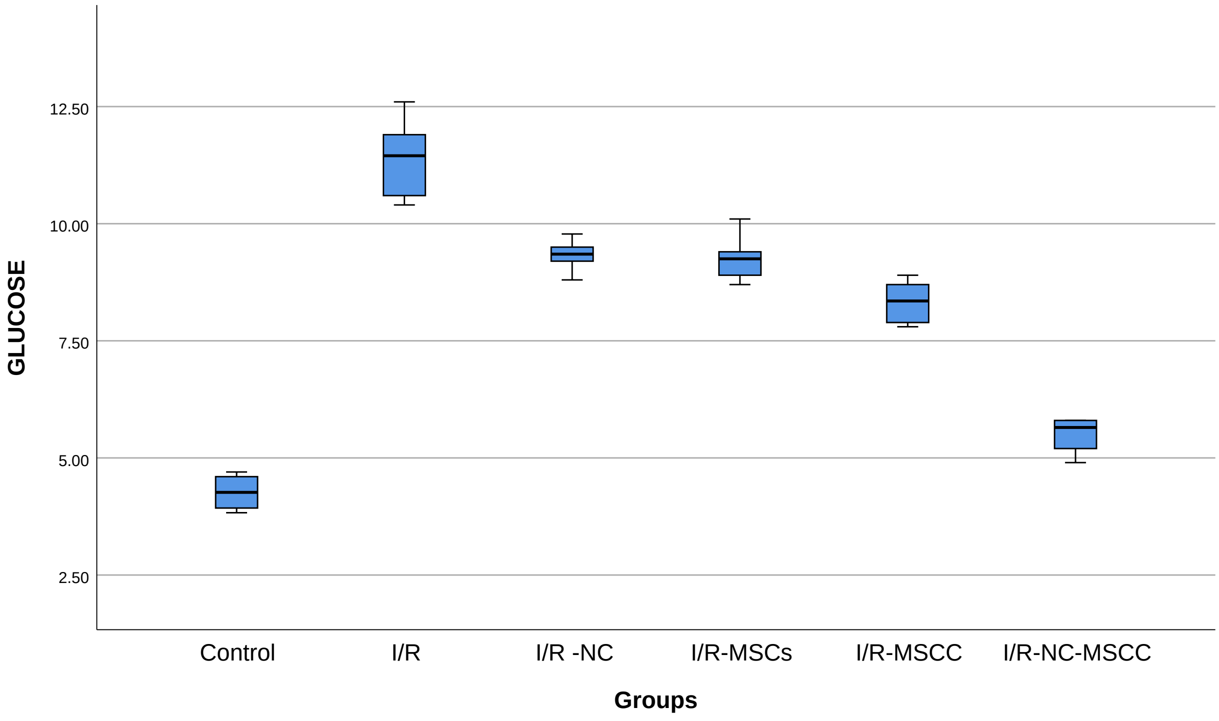

LDH

Normal Q-Q Plots

Normal Q-Q Plot of LDH

for Groups= Control

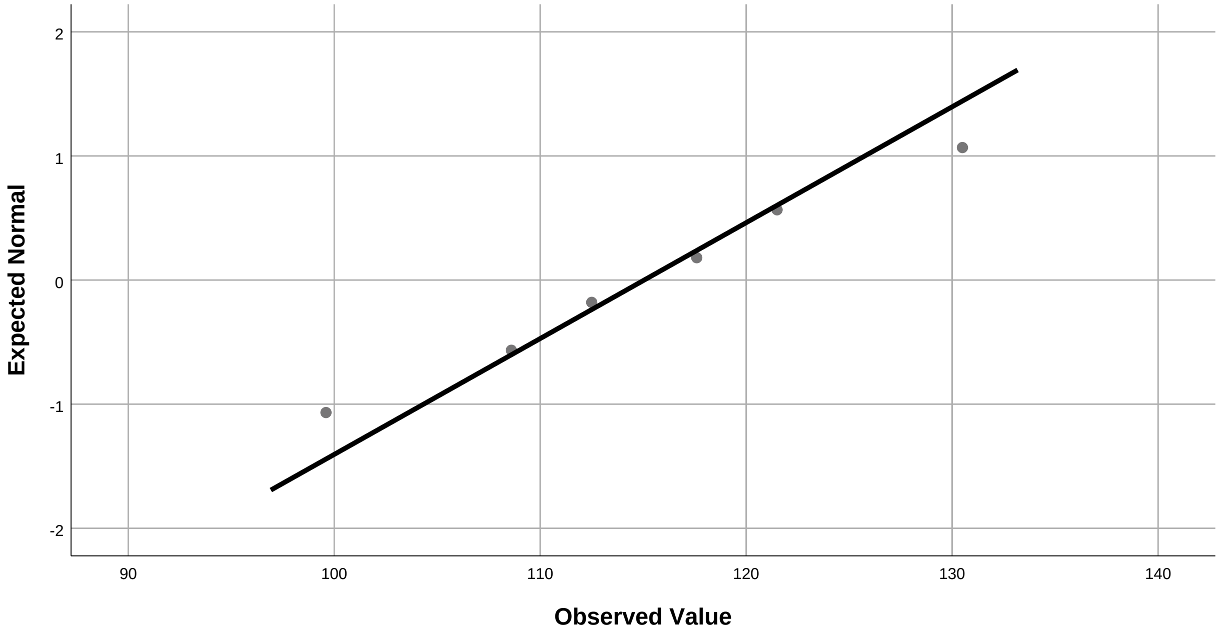

Normal Q-Q Plot of LDH

for Groups= I/R

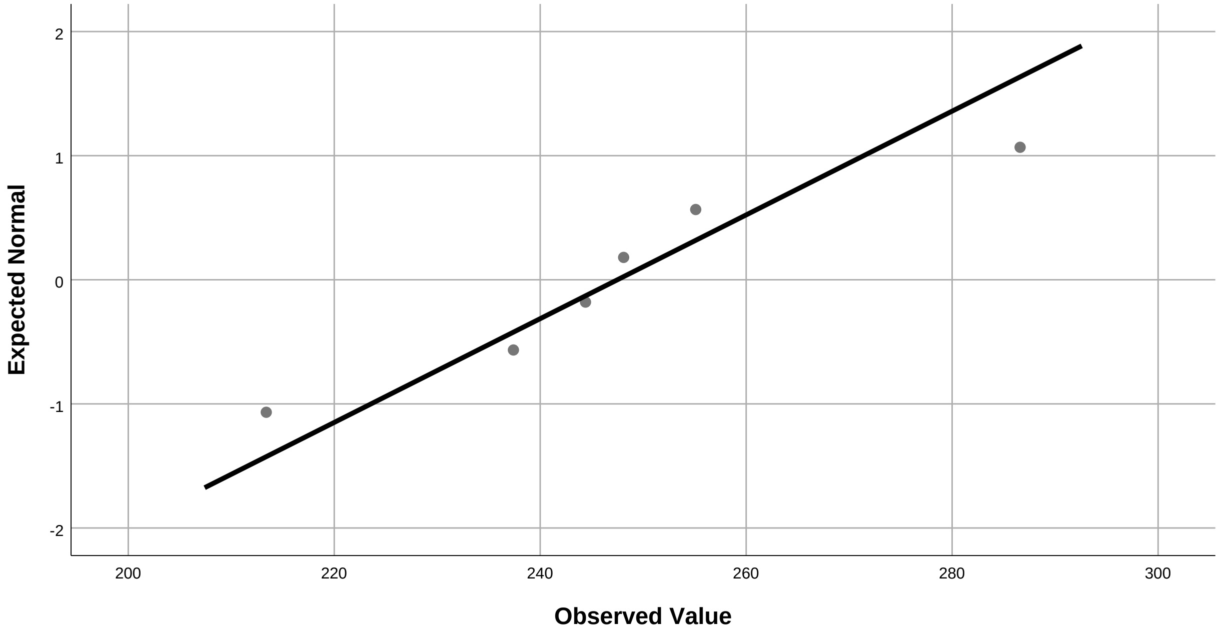

Normal Q-Q Plot of LDH

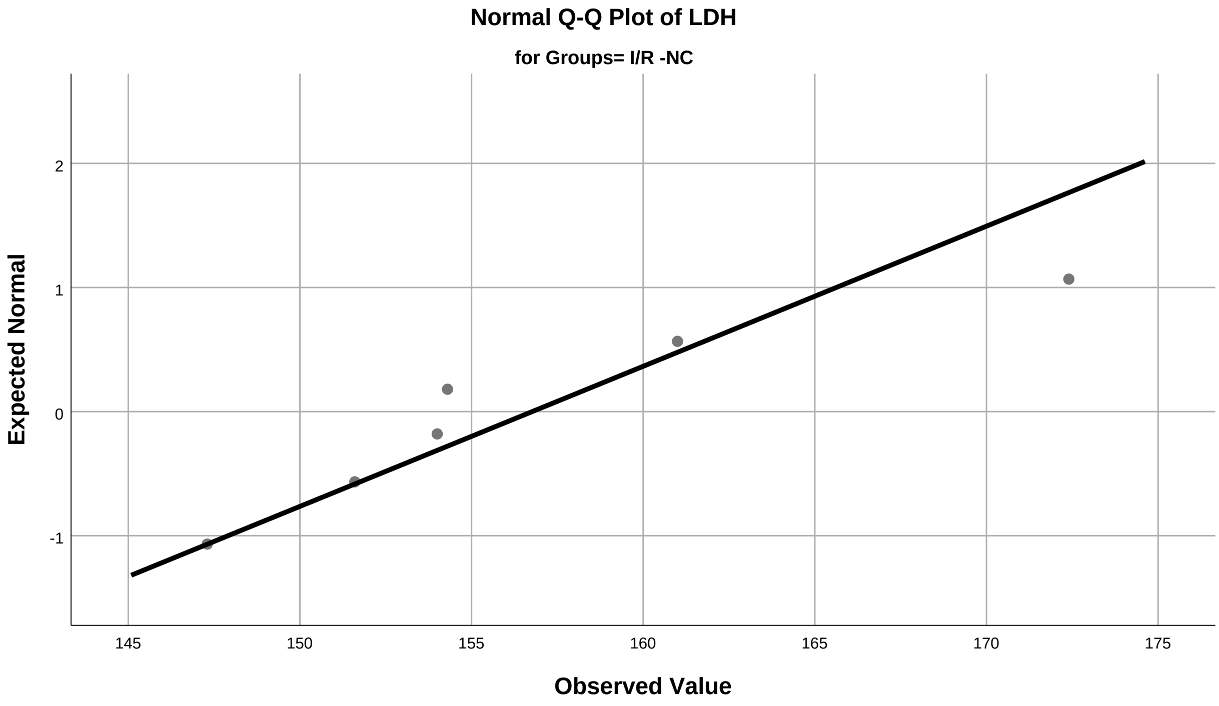

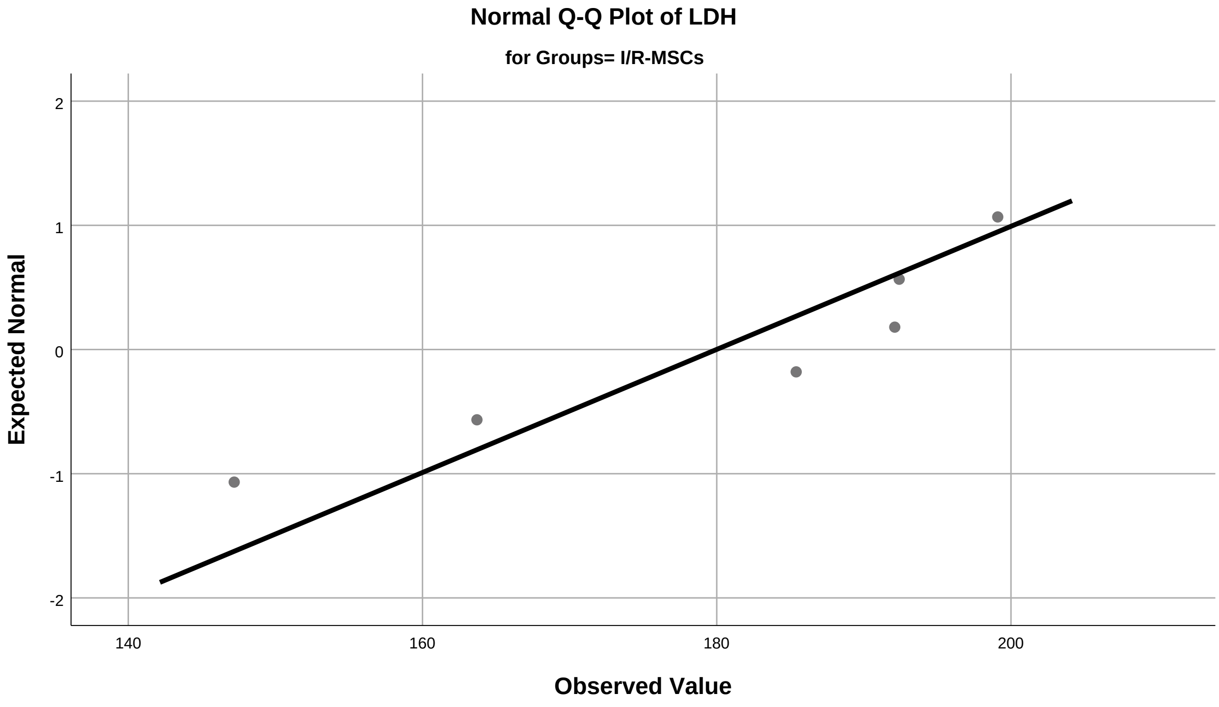

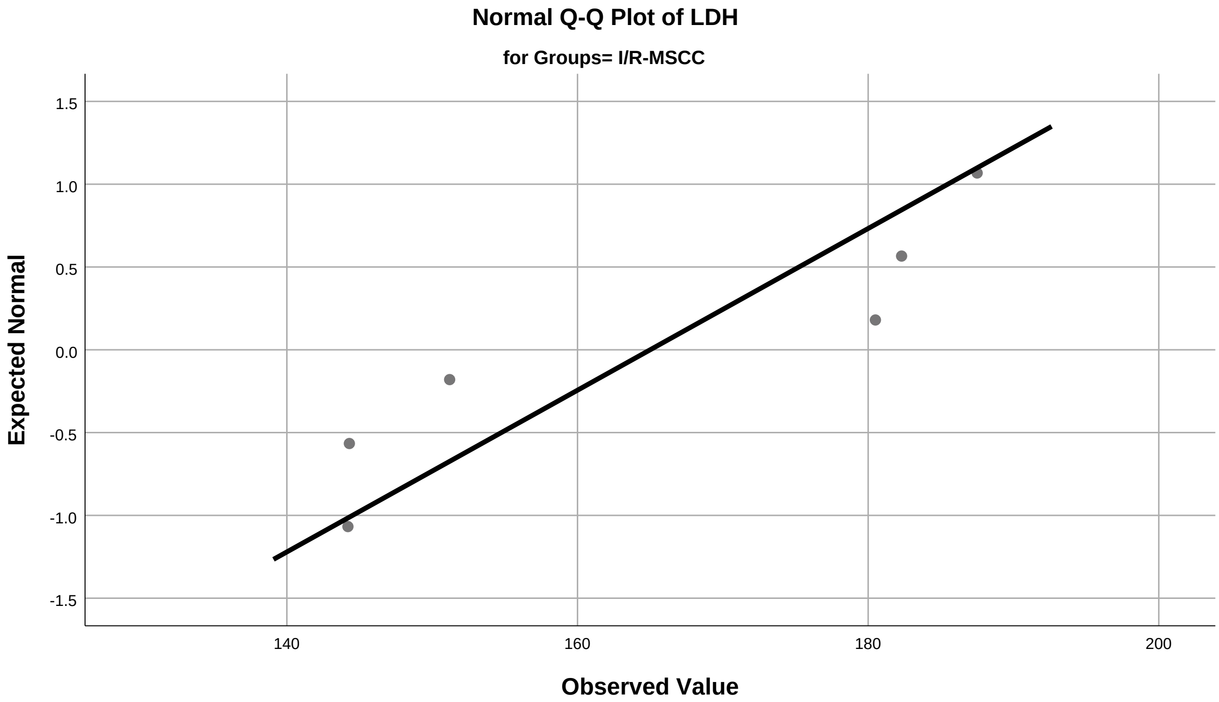

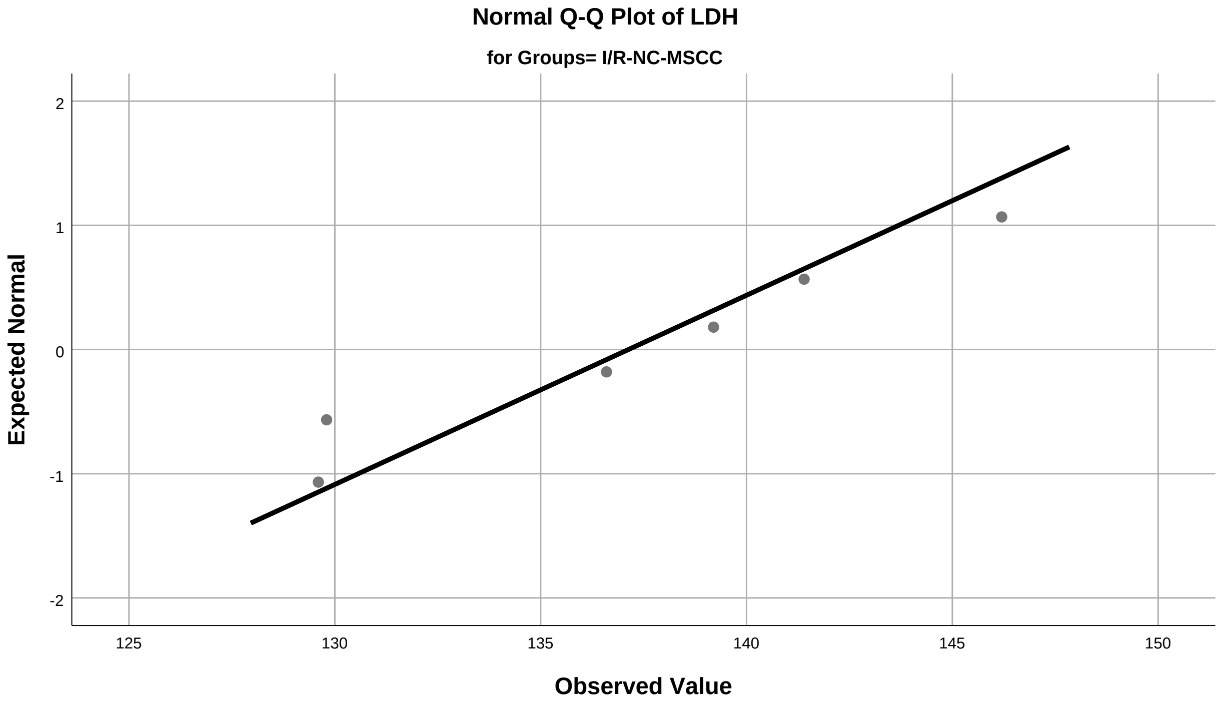

Detrended Normal Q-Q Plots

Detrended Normal Q-Q Plot of LDH

for Groups= Control

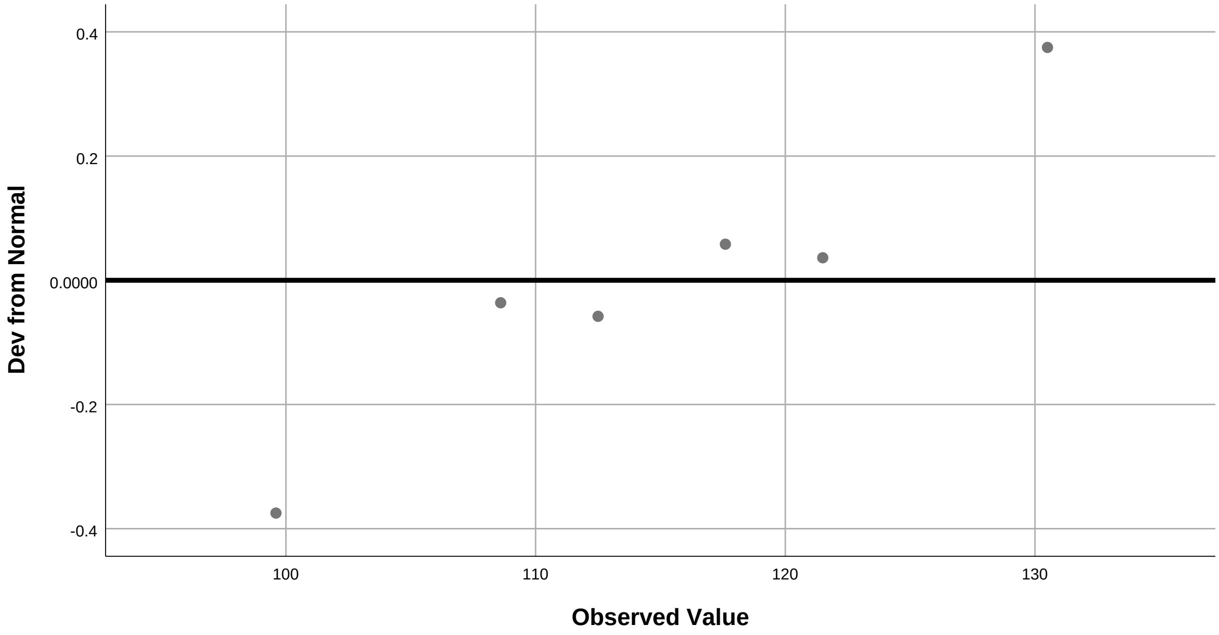

Detrended Normal Q-Q Plot of LDH

for Groups= I/R

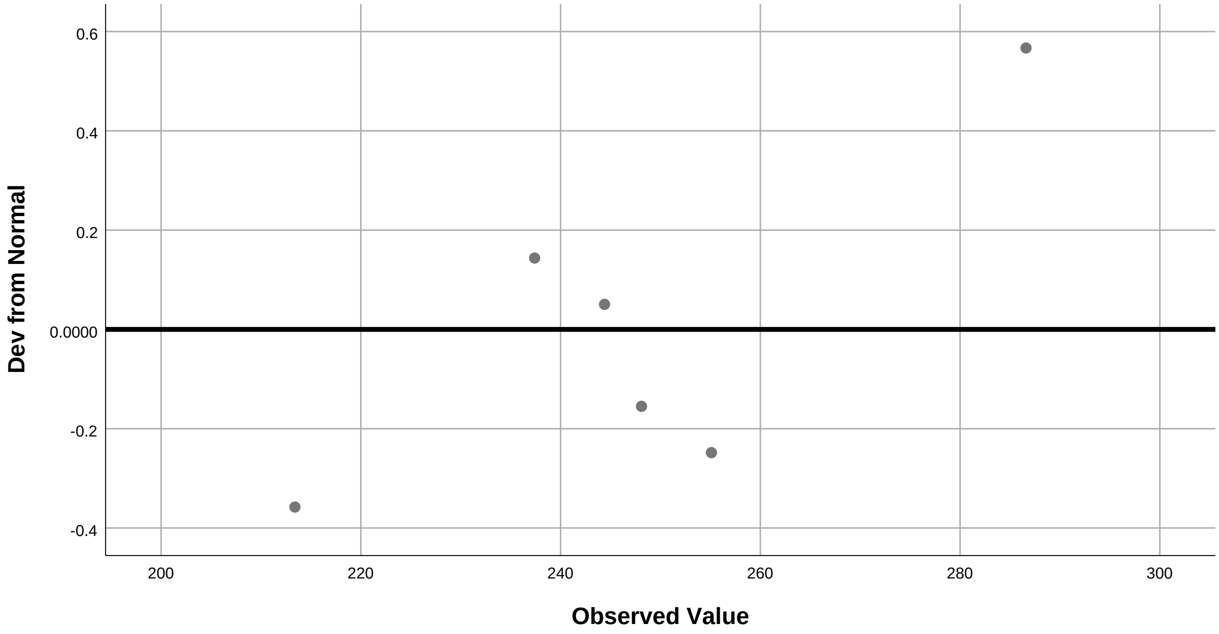

Detrended Normal Q-Q Plot of LDH

for Groups= I/R -NC

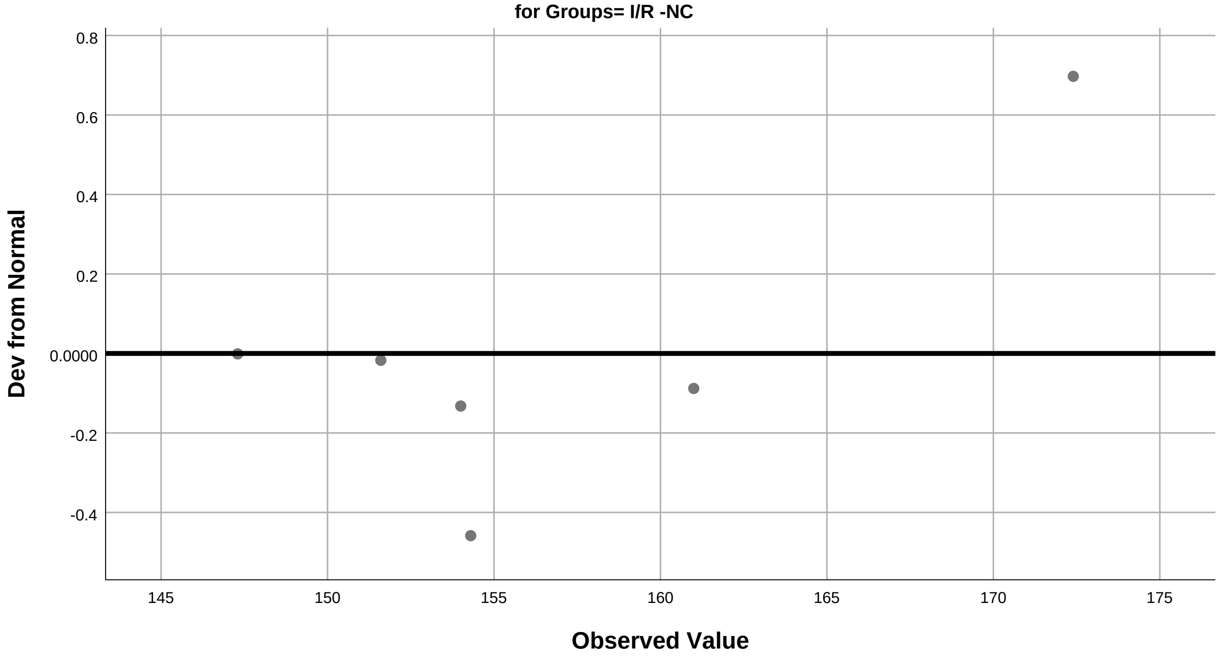

Detrended Normal Q-Q Plot of LDH

for Groups= I/R-MSCs

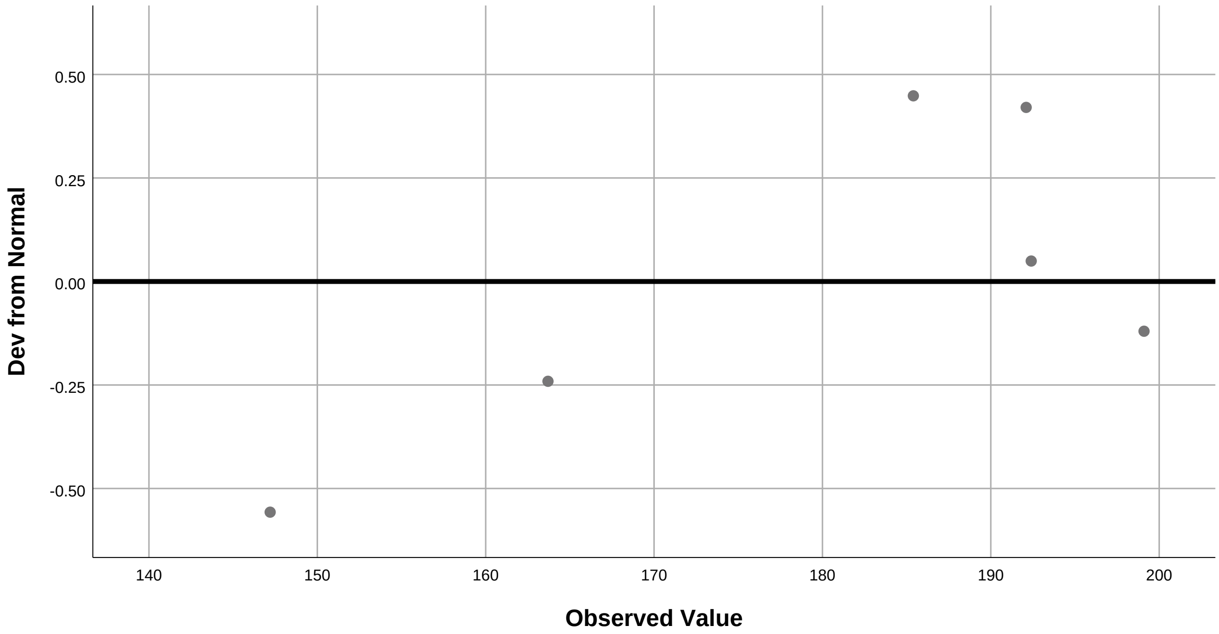

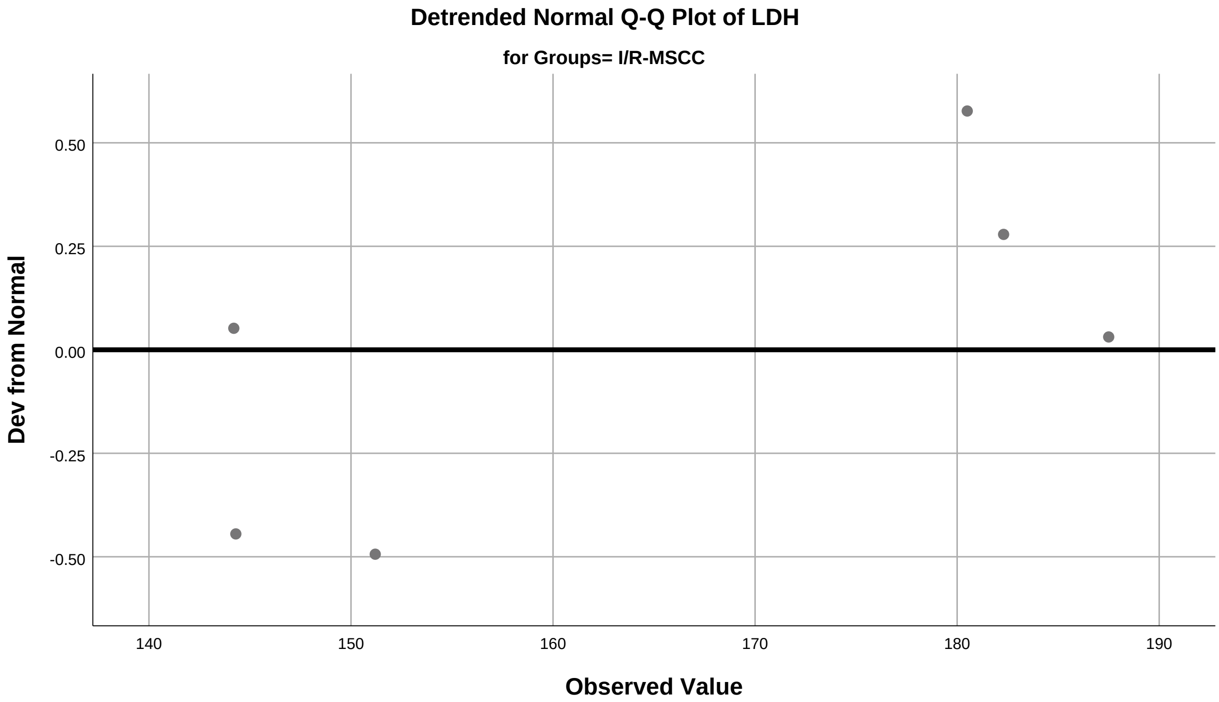

Detrended Normal Q-Q Plot of LDH
for Groups= I/R-MSCC
Dev from Normal
0.50
0.25
0.00
-0.25
-0.50
140
150
160
170
180
190
Observed Value

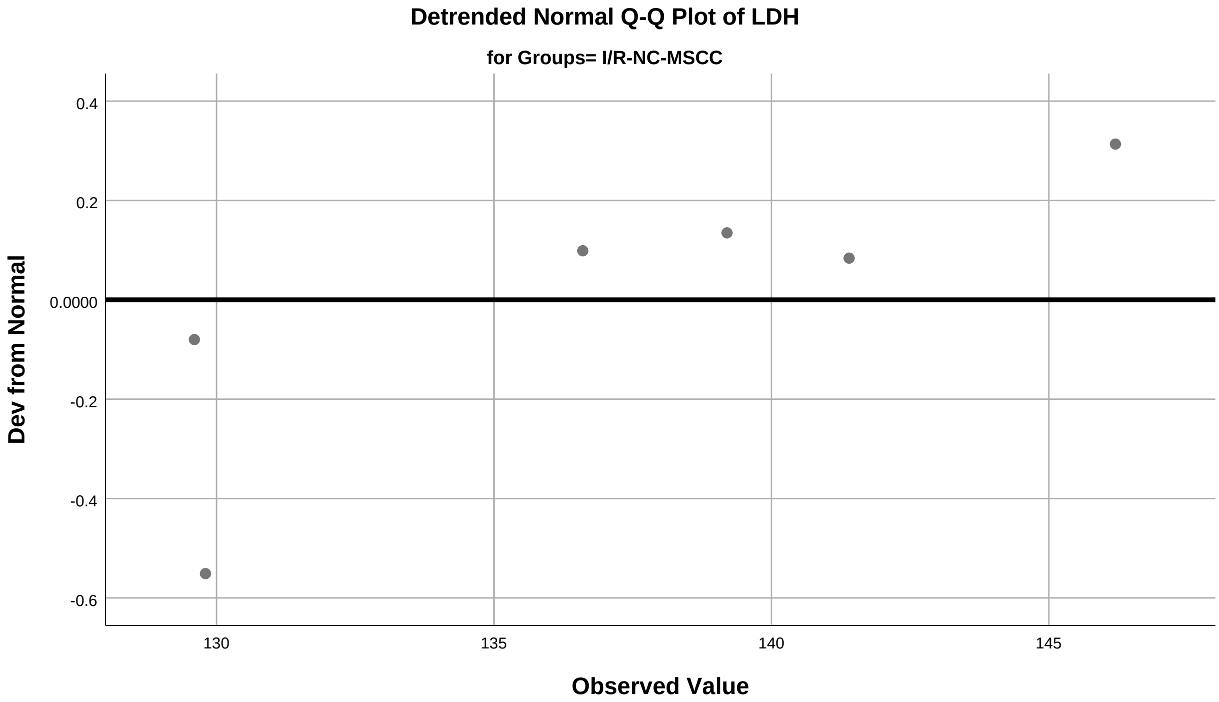

Detrended Normal Q-Q Plot of LDH
for Groups= I/R-NC-MSCC
Dev from Normal
0.4
0.2
0.0000
-0.2
-0.4
-0.6
130
135
140
145
Observed Value

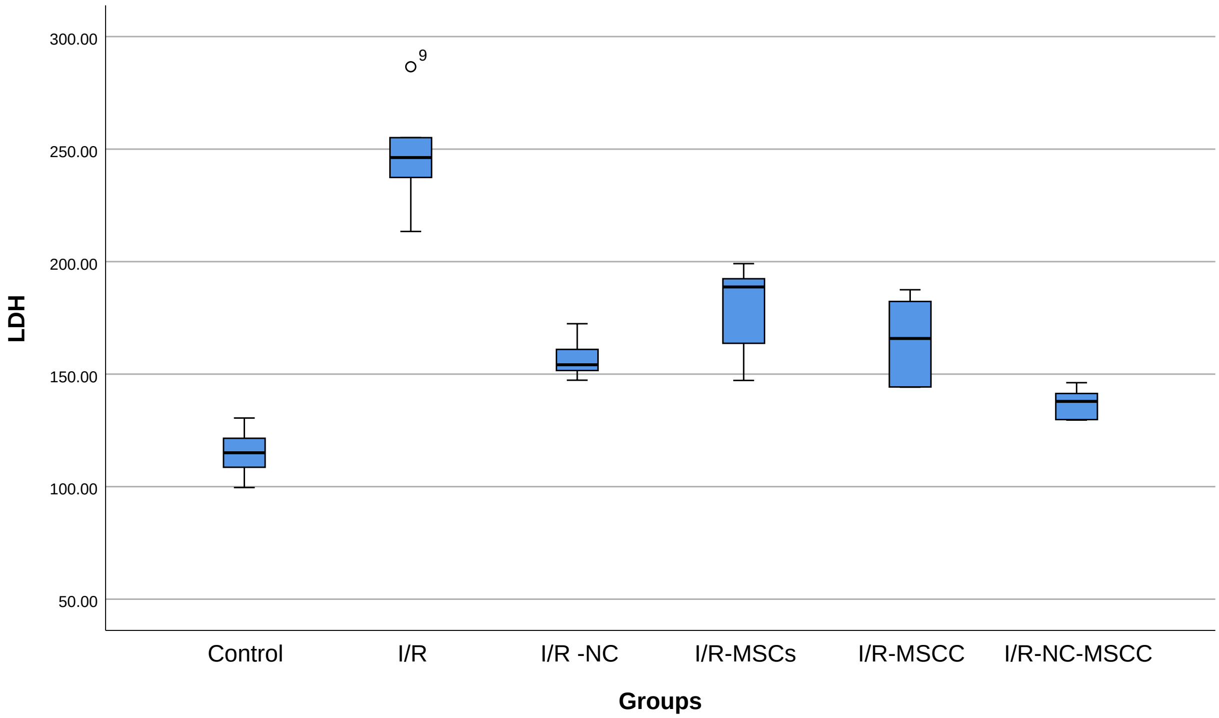

AMYLASE

Normal Q-Q Plots

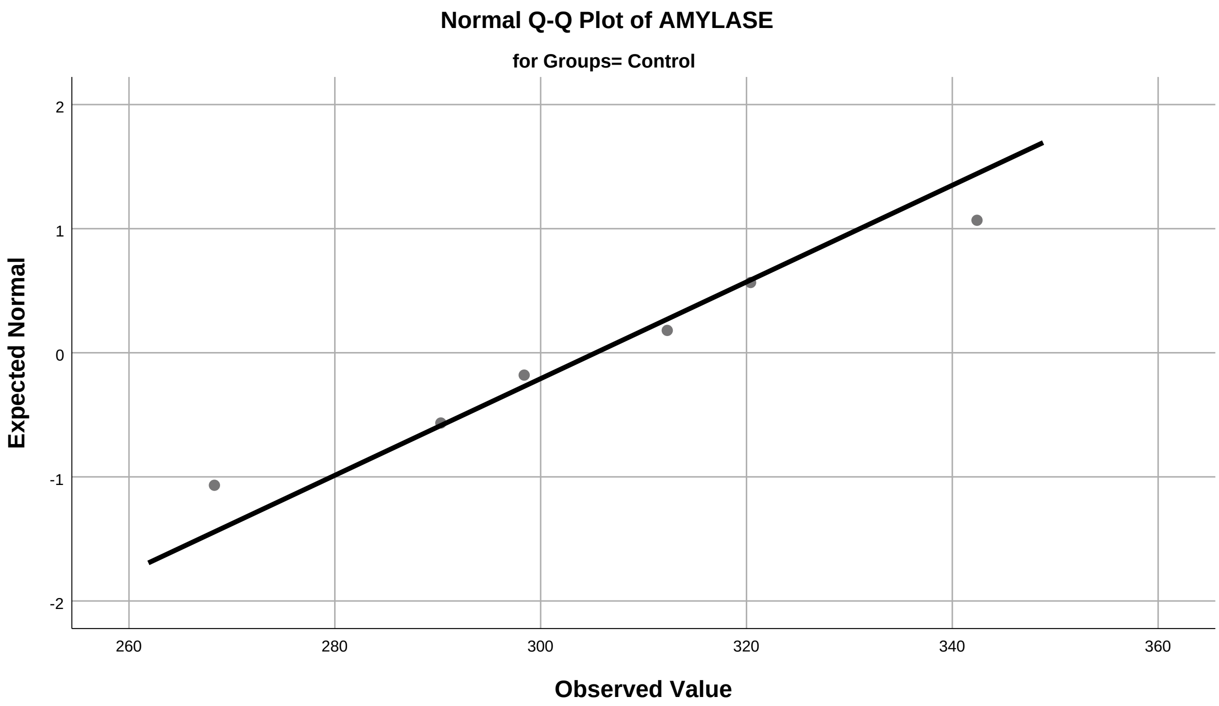

Normal Q-Q Plot of AMYLASE

for Groups= I/R

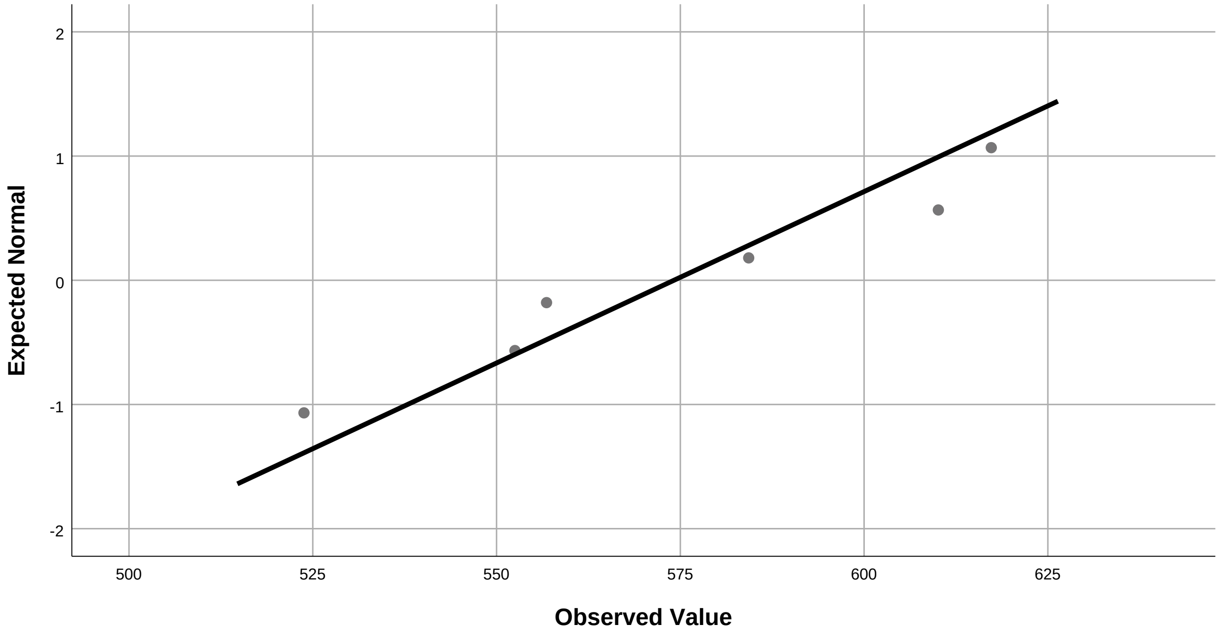

Normal Q-Q Plot of AMYLASE

for Groups= I/R -NC

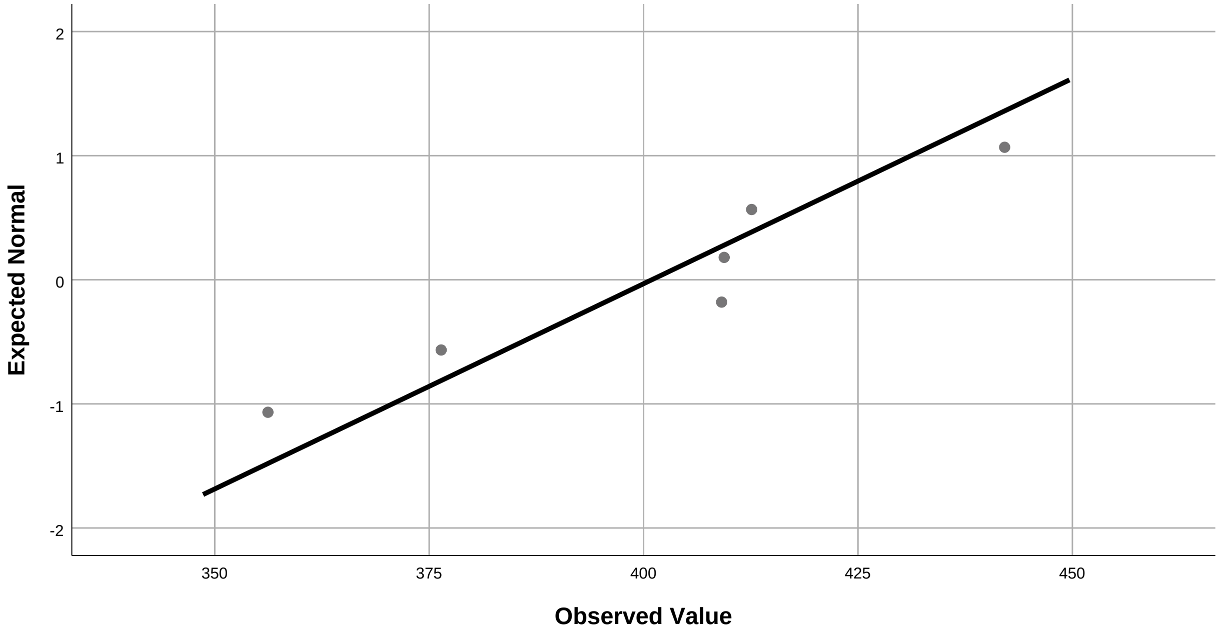

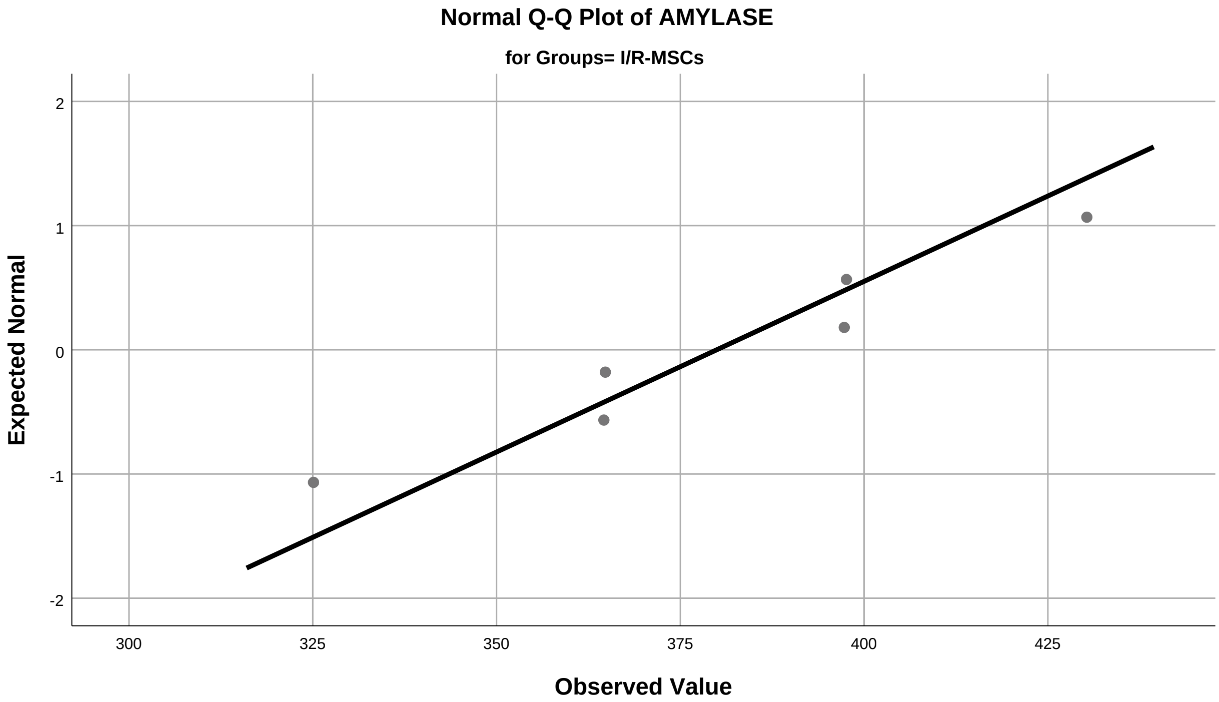
Normal Q-Q Plot of AMYLASE
for Groups= I/R-MSCs
Expected Normal
2
1
0
-1
-2
300
325
350
375
400
425
Observed Value

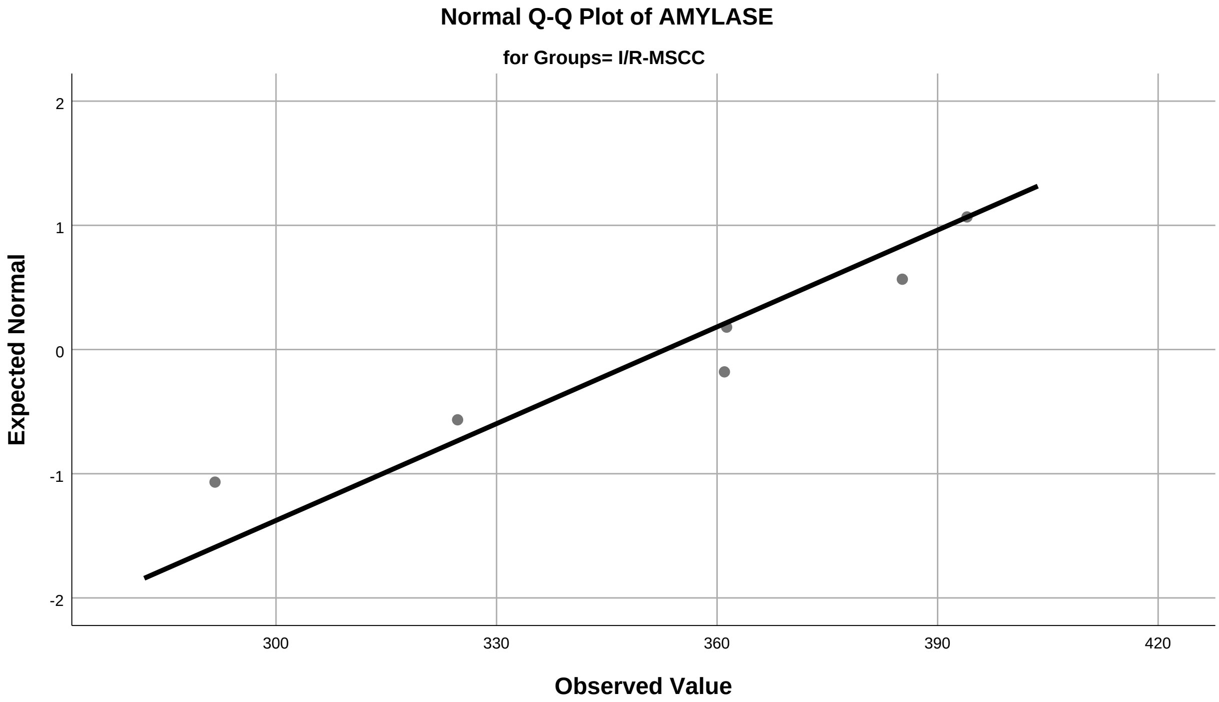
Normal Q-Q Plot of AMYLASE
for Groups= I/R-MSCC
Expected Normal
2
1
0
-1
-2
300
330
360
390
420
Observed Value

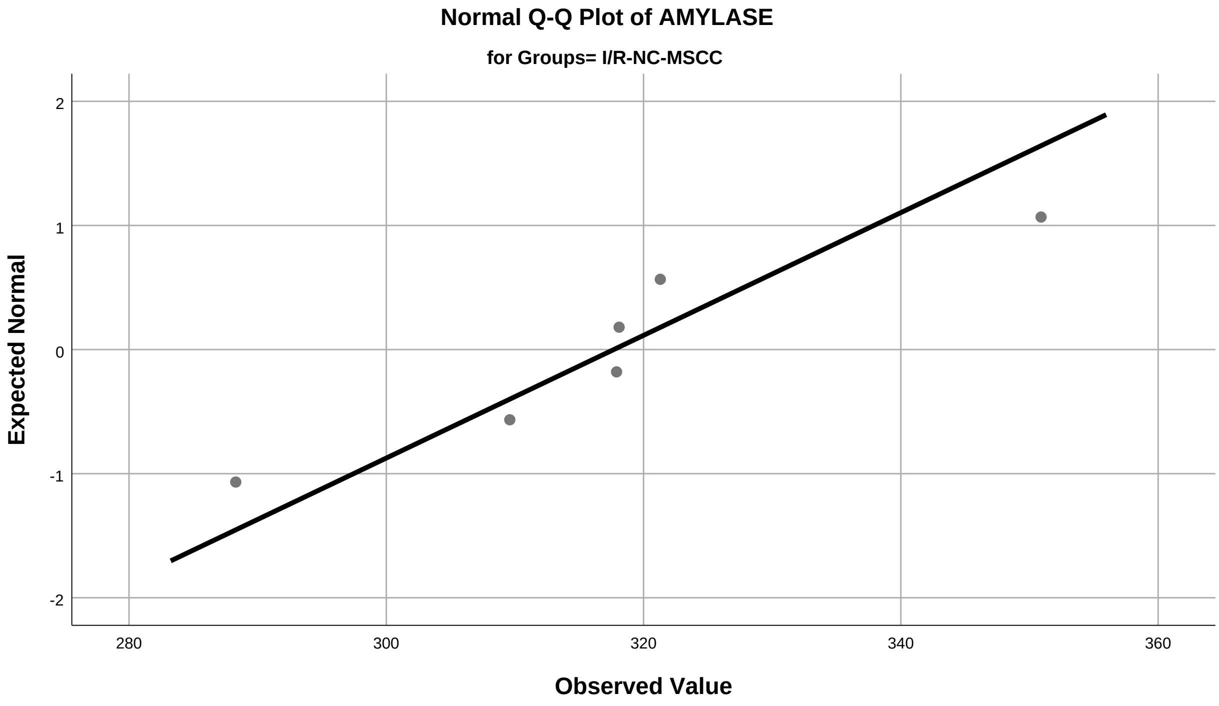

Detrended Normal Q-Q Plots

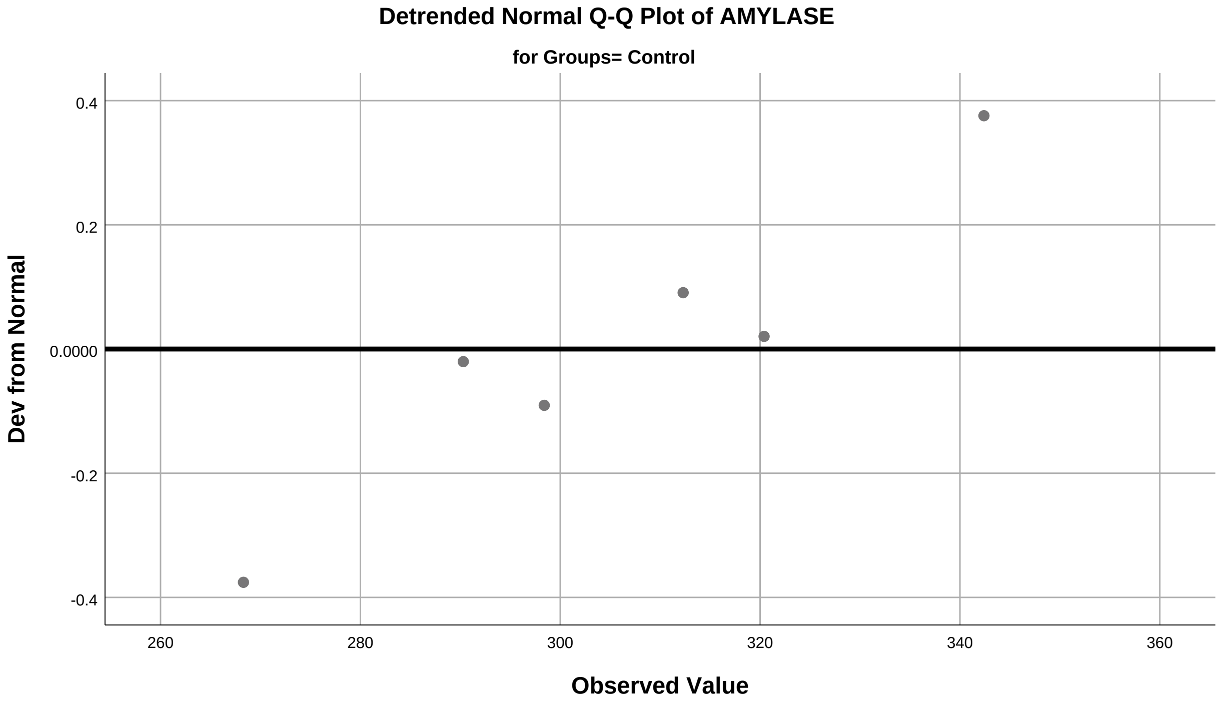

Detrended Normal Q-Q Plot of AMYLASE

for Groups= I/R

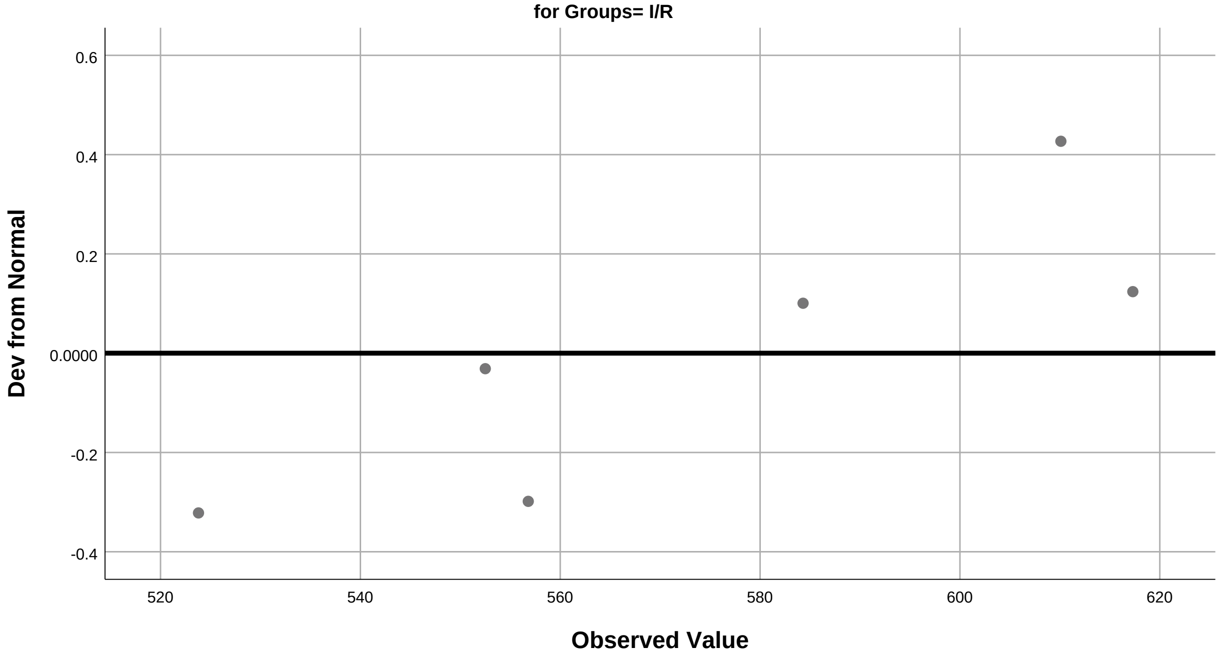

Detrended Normal Q-Q Plot of AMYLASE

for Groups= I/R -NC

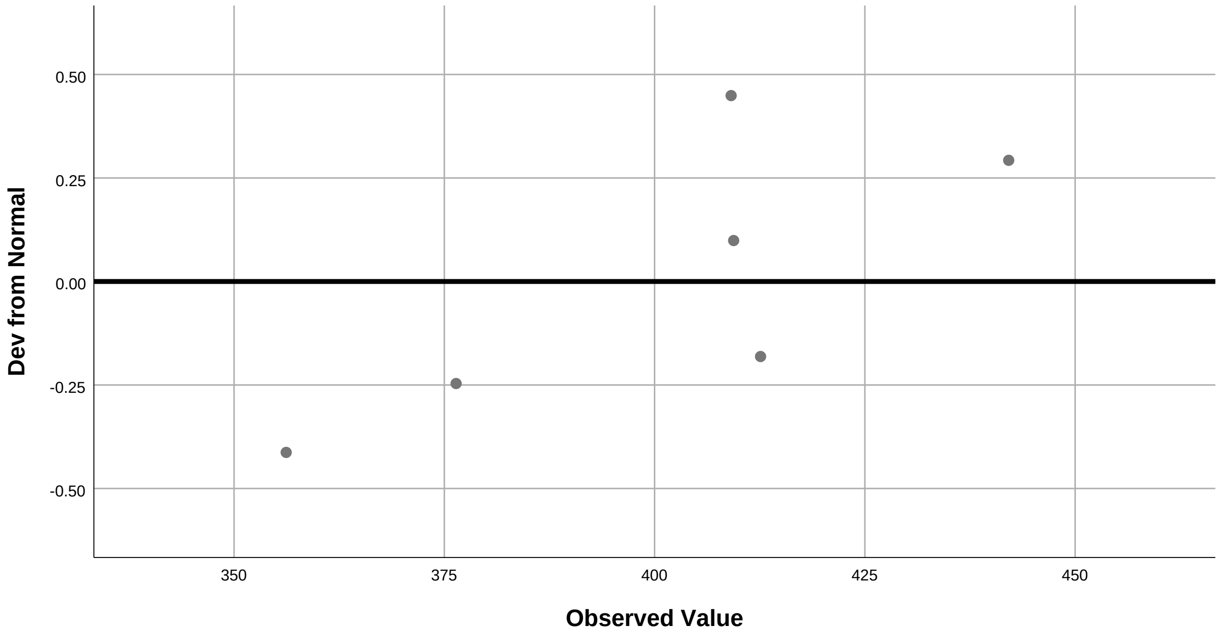

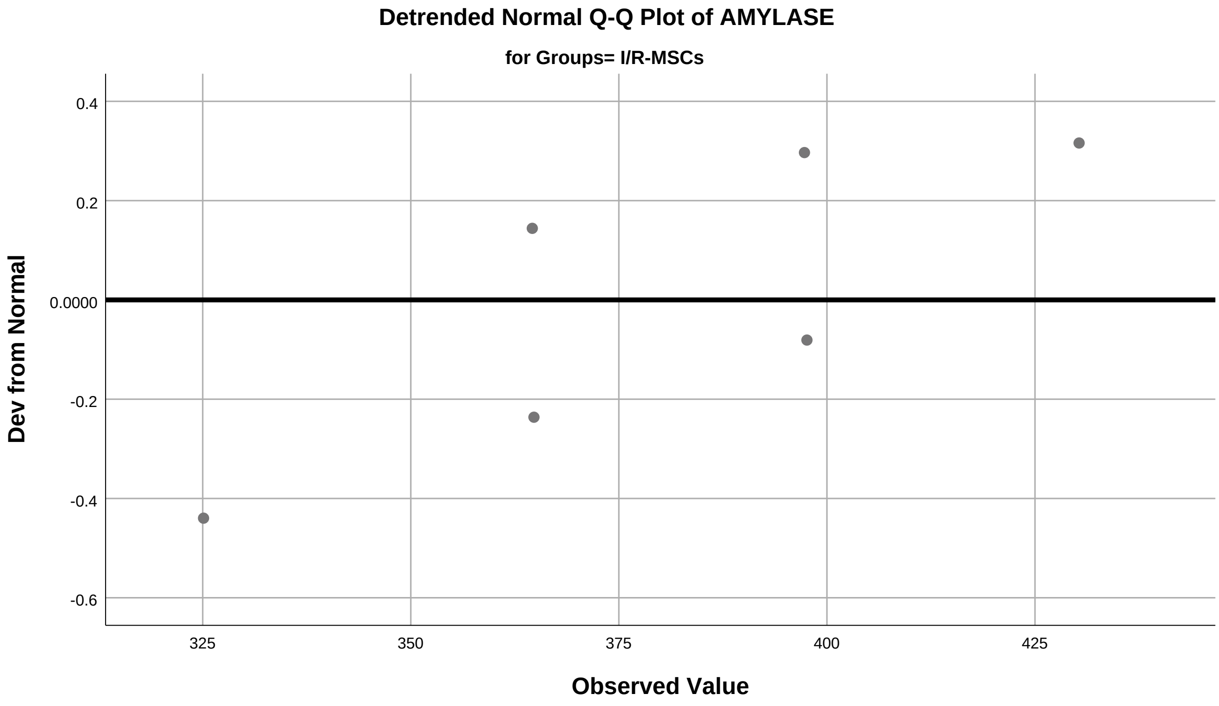
Detrended Normal Q-Q Plot of AMYLASE
for Groups= I/R-MSCs
Dev from Normal
0.4
0.2
0.0000
-0.2
-0.4
-0.6
325
350
375
400
425
Observed Value

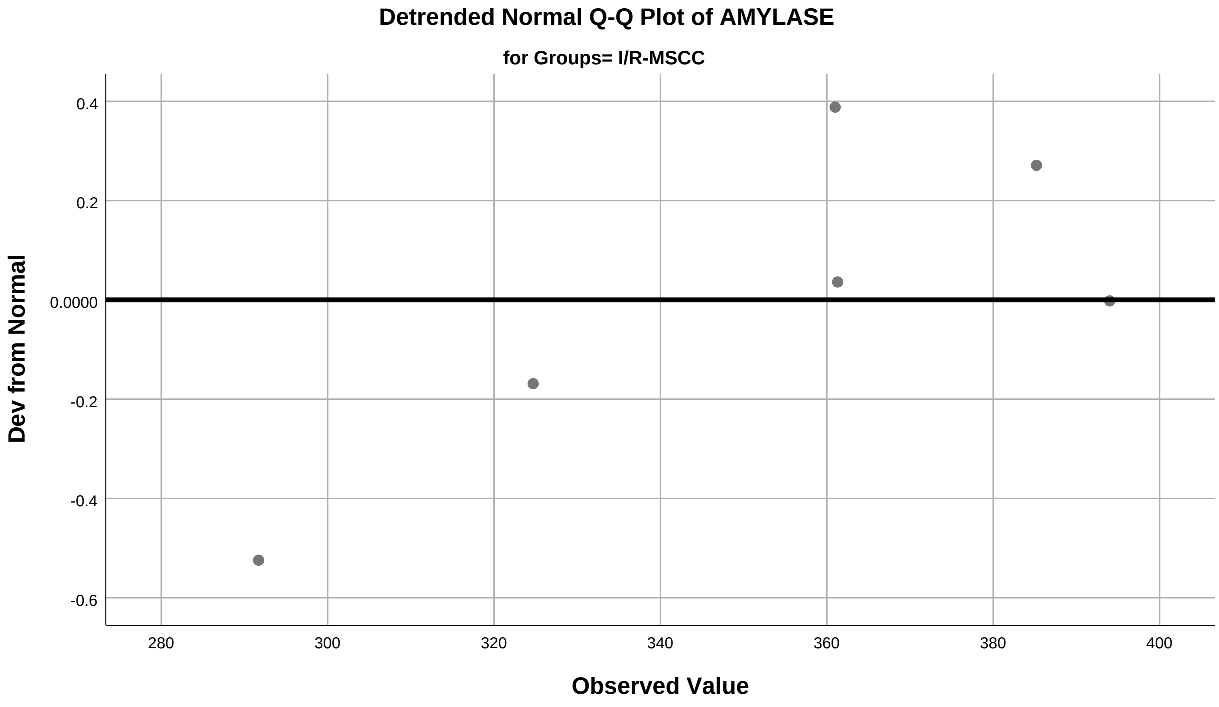
Detrended Normal Q-Q Plot of AMYLASE
for Groups= I/R-MSCC
Dev from Normal
0.4
0.2
0.0000
-0.2
-0.4
-0.6
280
300
320
340
360
380
400
Observed Value

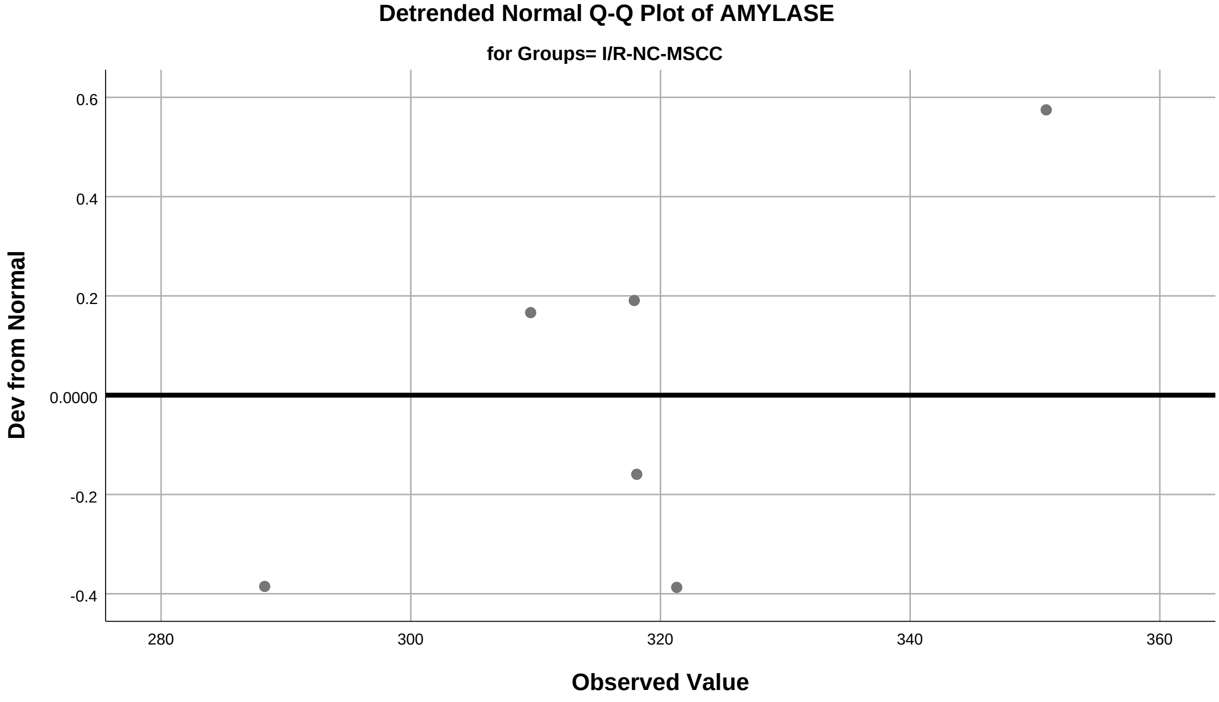

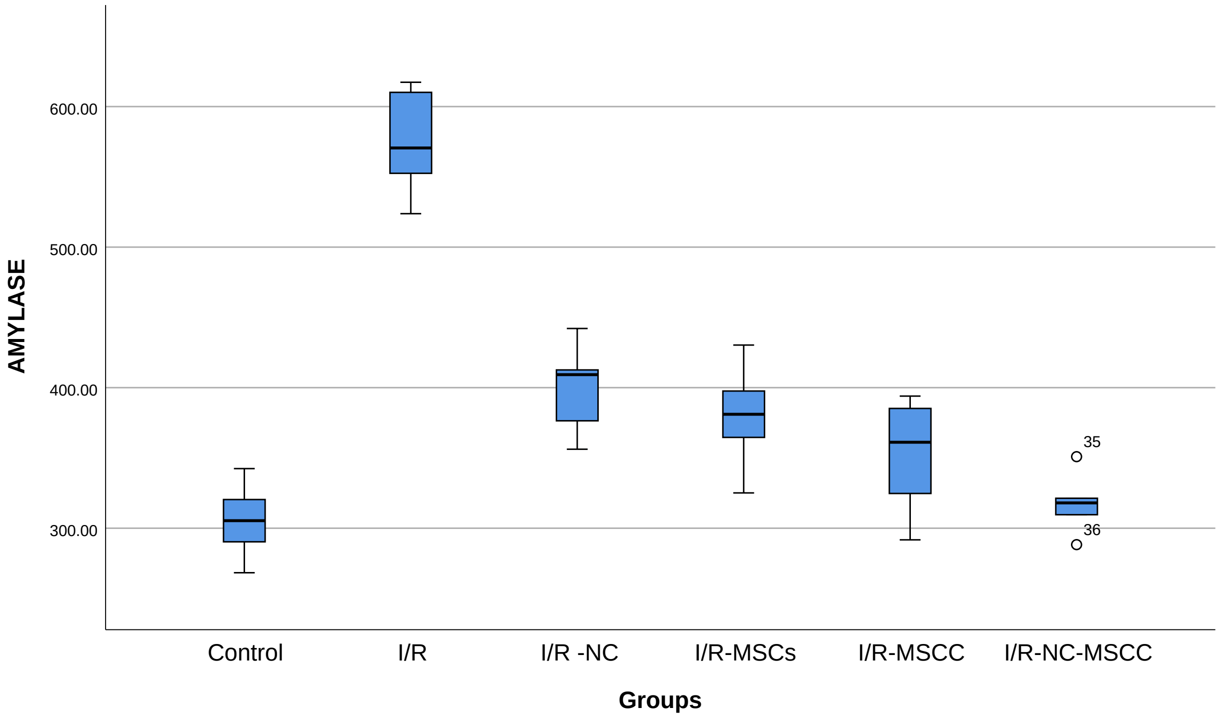

MPO

Normal Q-Q Plots

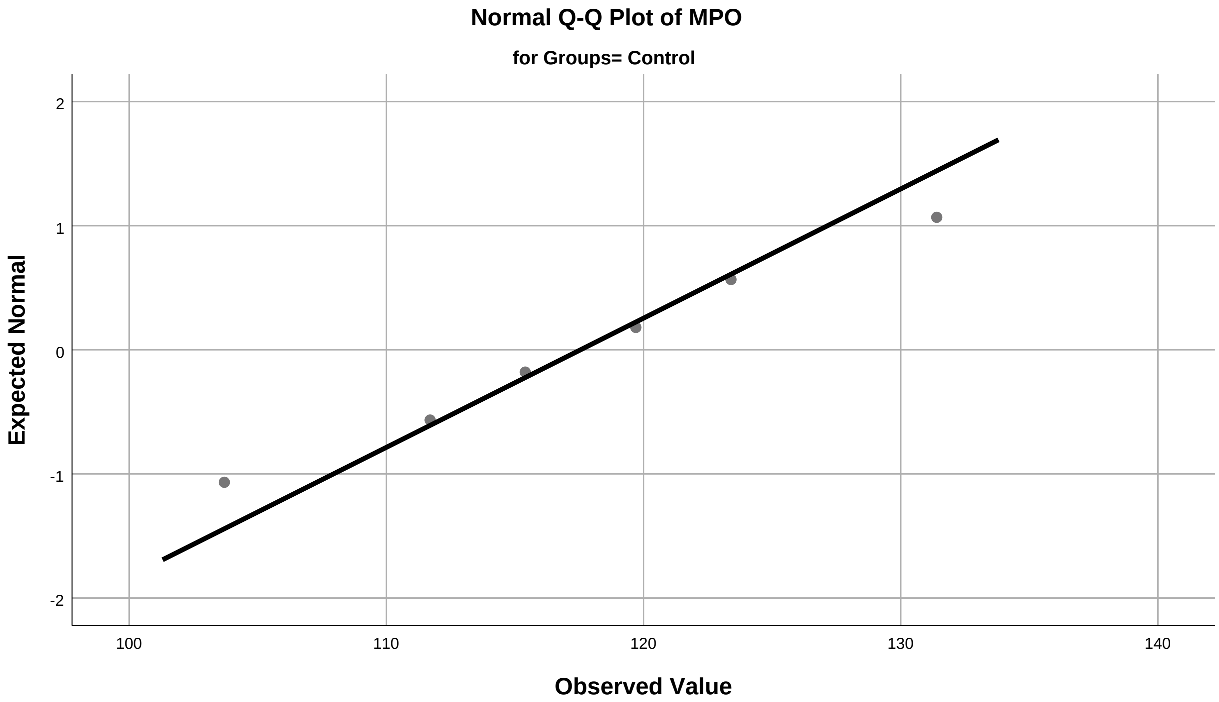
Normal Q-Q Plot of MPO
for Groups= Control
Expected Normal
2
1
0
-1
-2
100
110
120
130
140
Observed Value

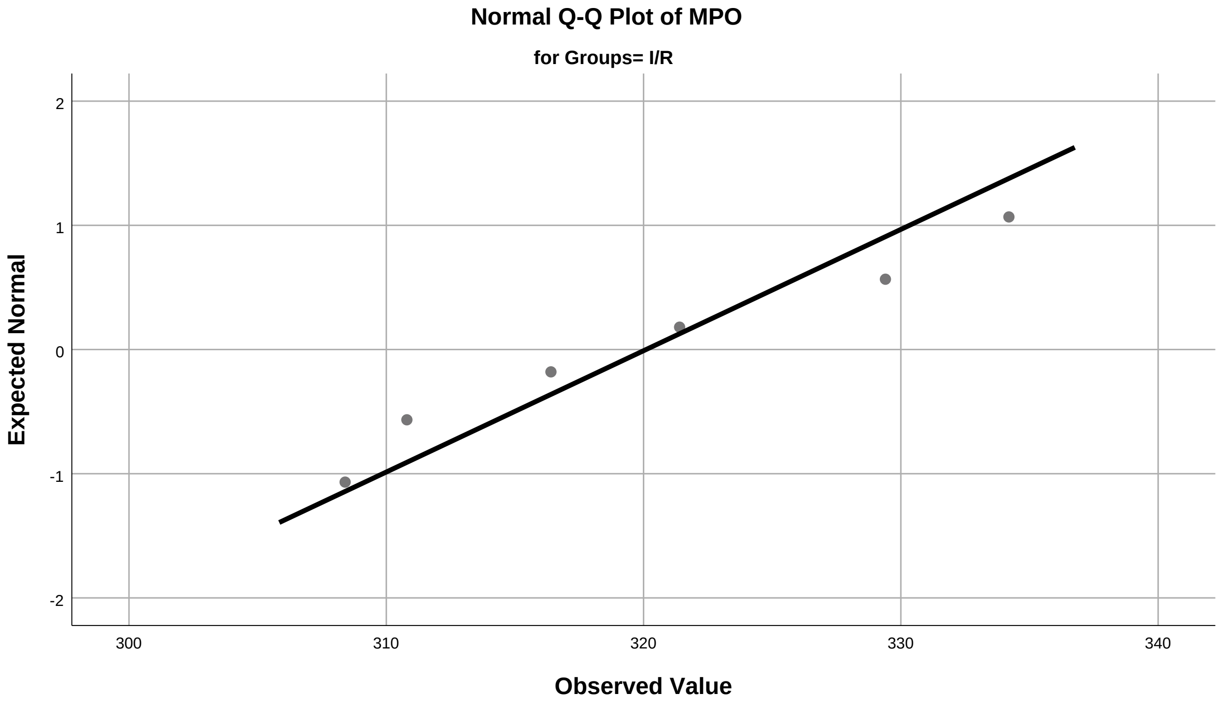
Normal Q-Q Plot of MPO
for Groups= I/R
Expected Normal
2
1
0
-1
-2
300
310
320
330
340
Observed Value

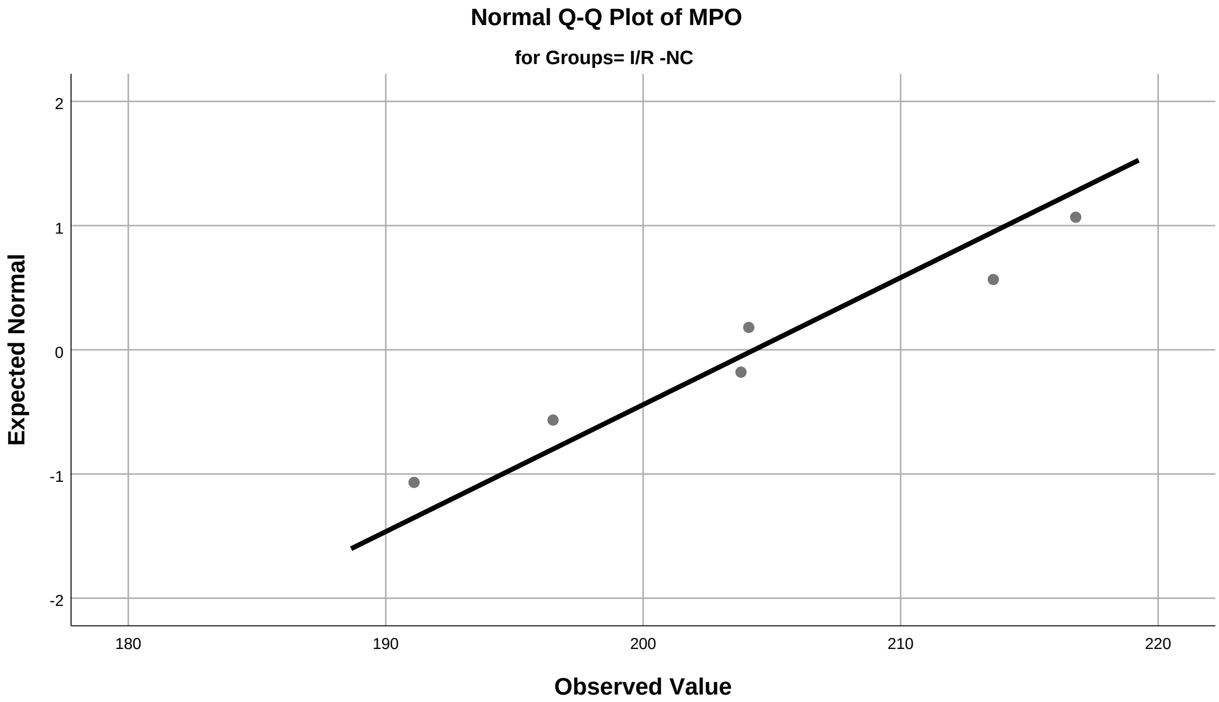
Normal Q-Q Plot of MPO
for Groups= I/R -NC
Expected Normal
2
1
0
-1
-2
180
190
200
210
220
Observed Value

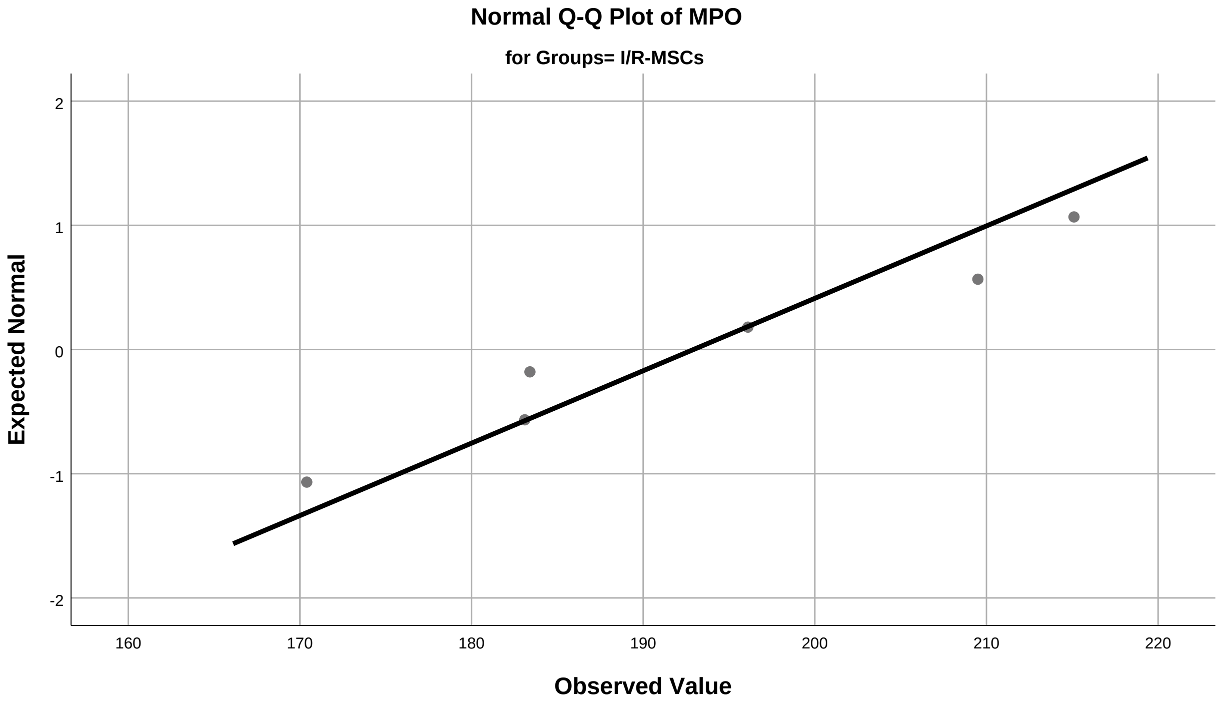
Normal Q-Q Plot of MPO
for Groups= I/R-MSCs
Expected Normal
2
1
0
-1
-2
160
170
180
190
200
210
220
Observed Value

Normal Q-Q Plot of MPO

for Groups= I/R-MSCC

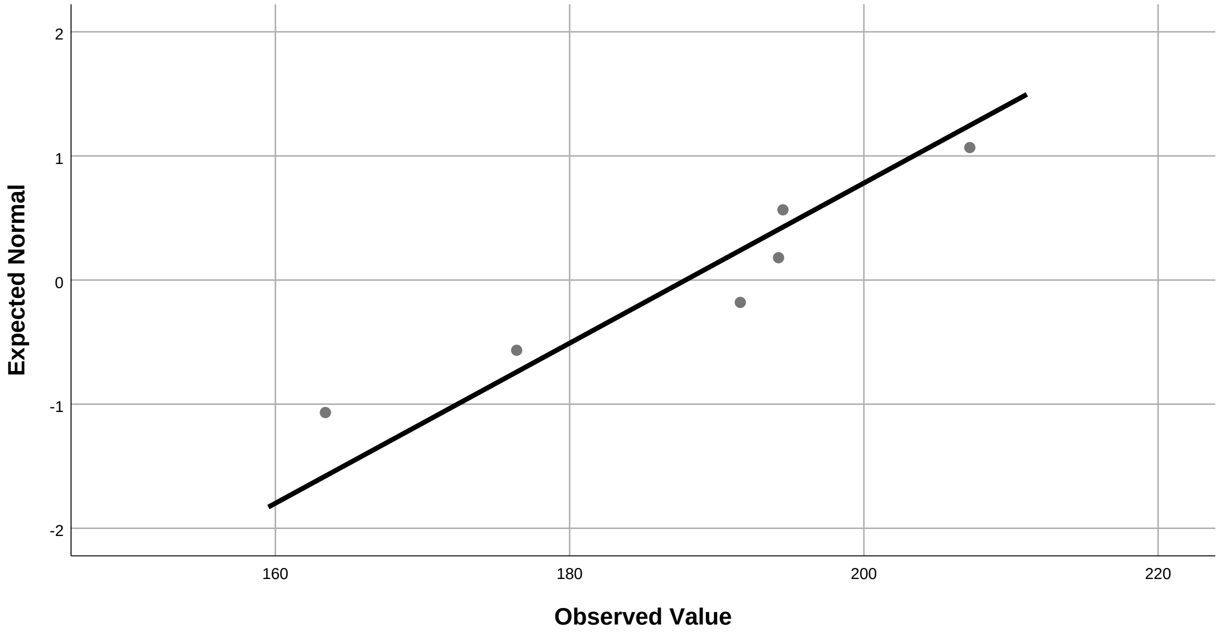

Normal Q-Q Plot of MPO

for Groups= I/R-NC-MSCC

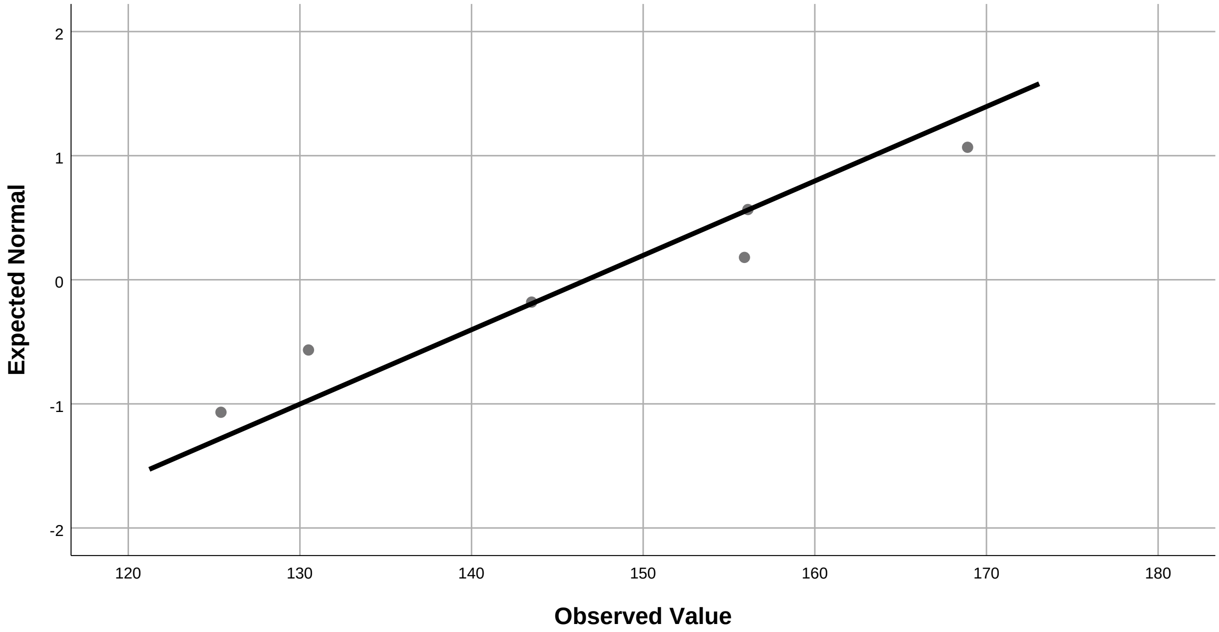

Detrended Normal Q-Q Plots

Detrended Normal Q-Q Plot of MPO

for Groups= Control

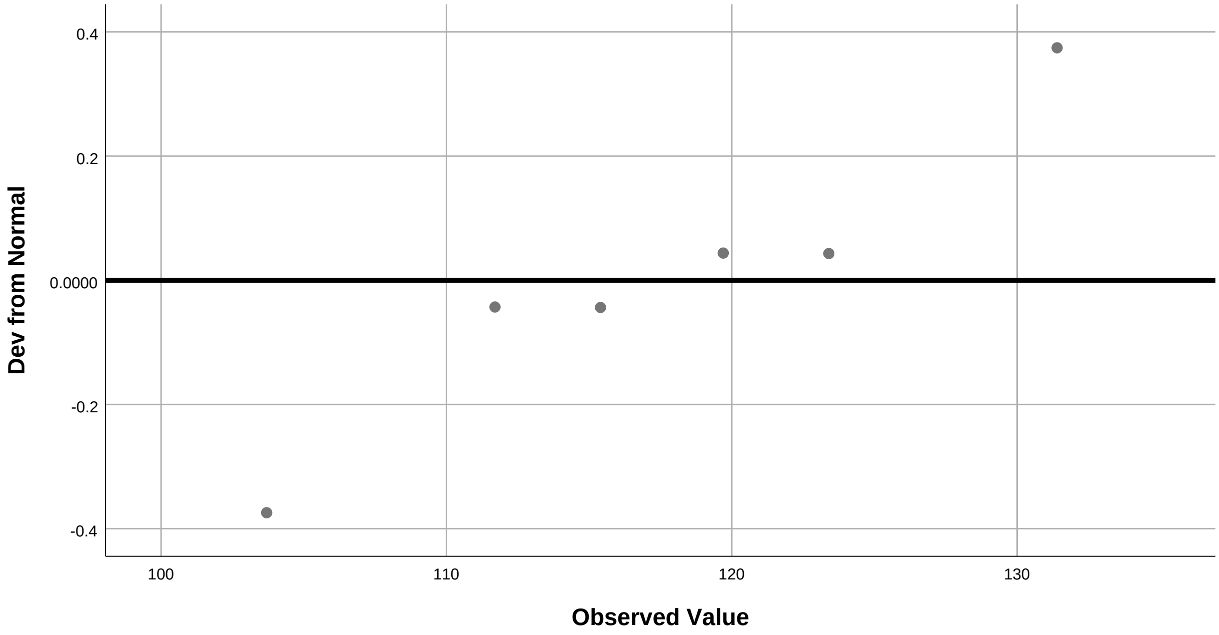

Detrended Normal Q-Q Plot of MPO

for Groups= I/R

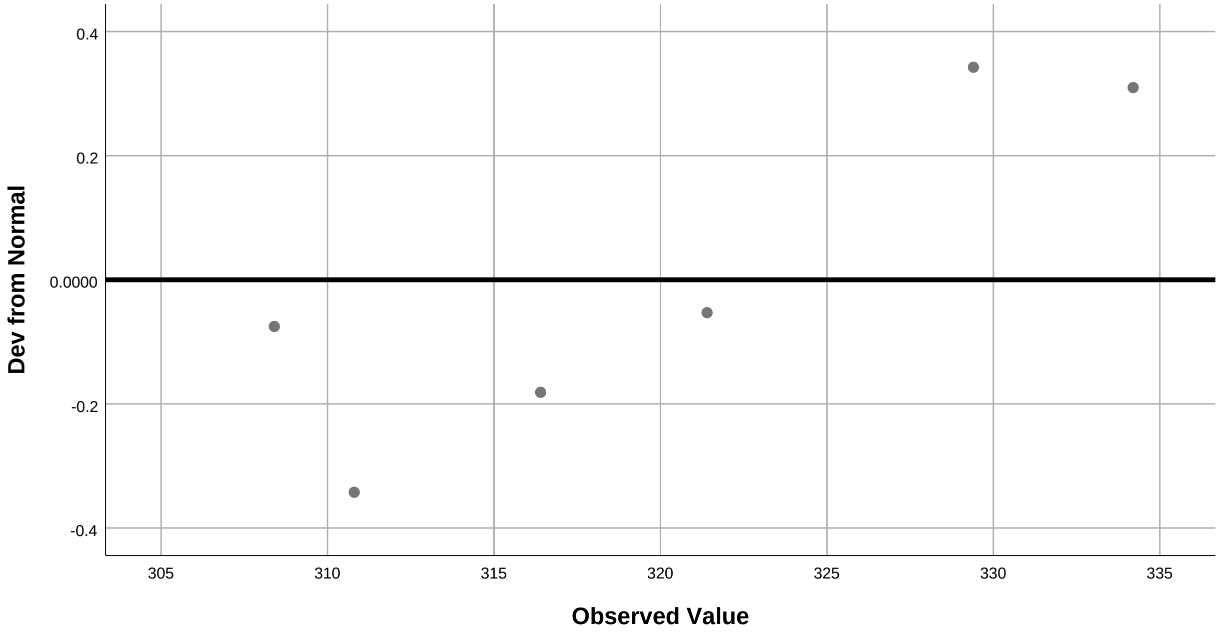

Detrended Normal Q-Q Plot of MPO

for Groups= I/R -NC

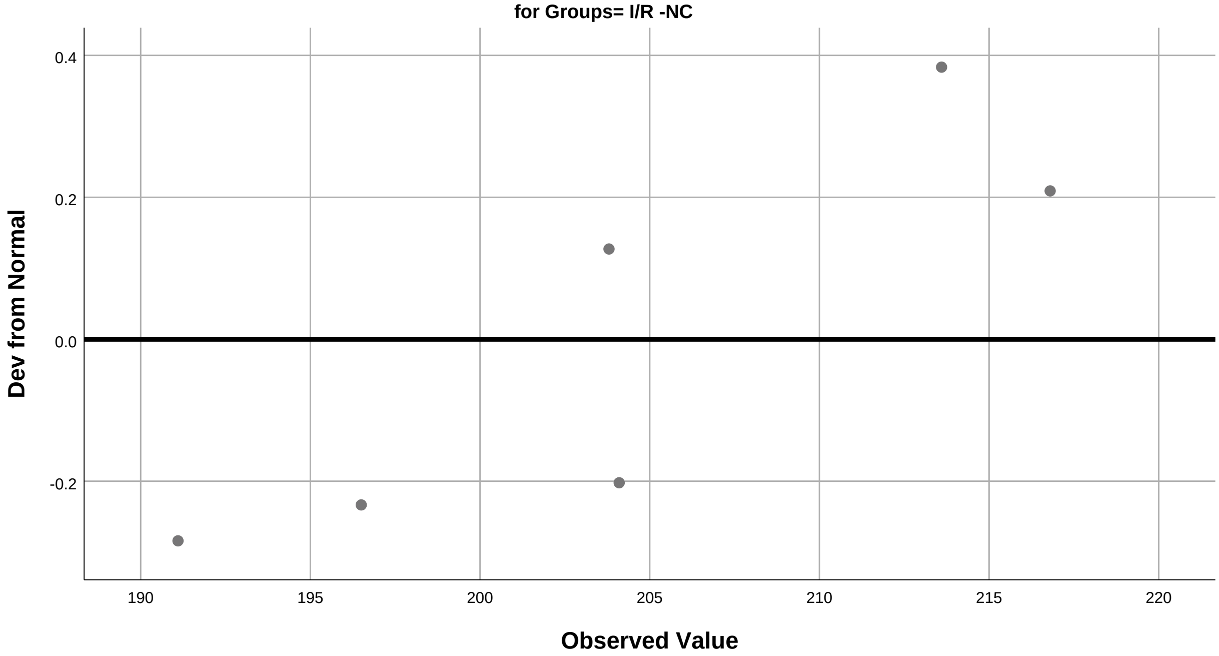

Detrended Normal Q-Q Plot of MPO

for Groups= I/R-MSCs

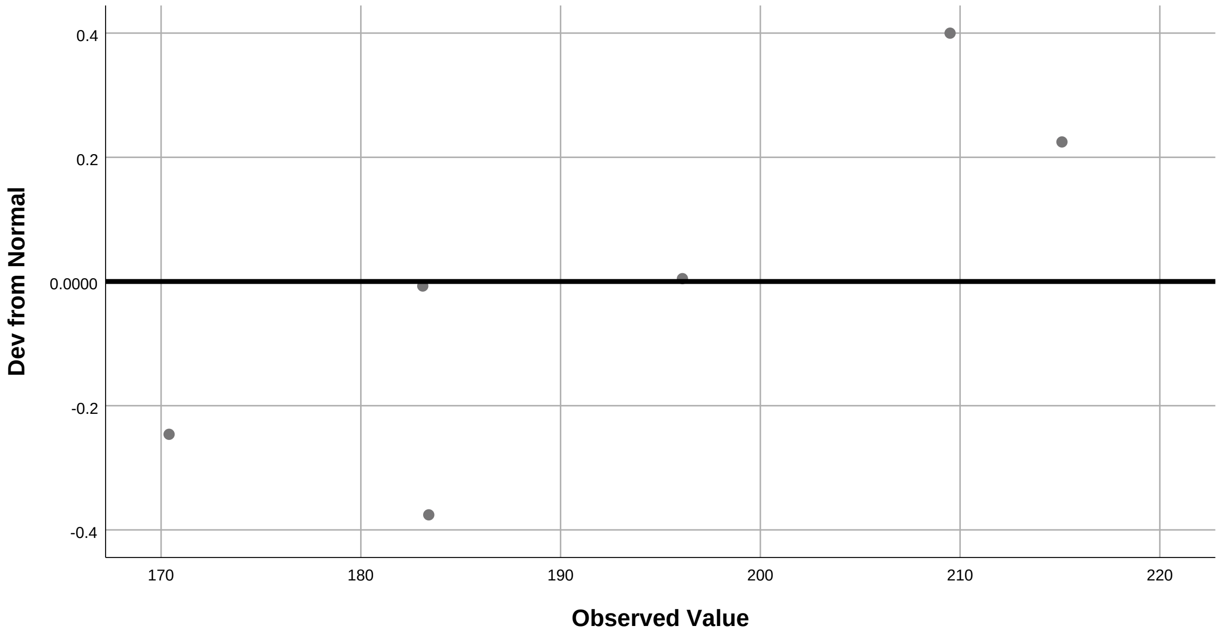

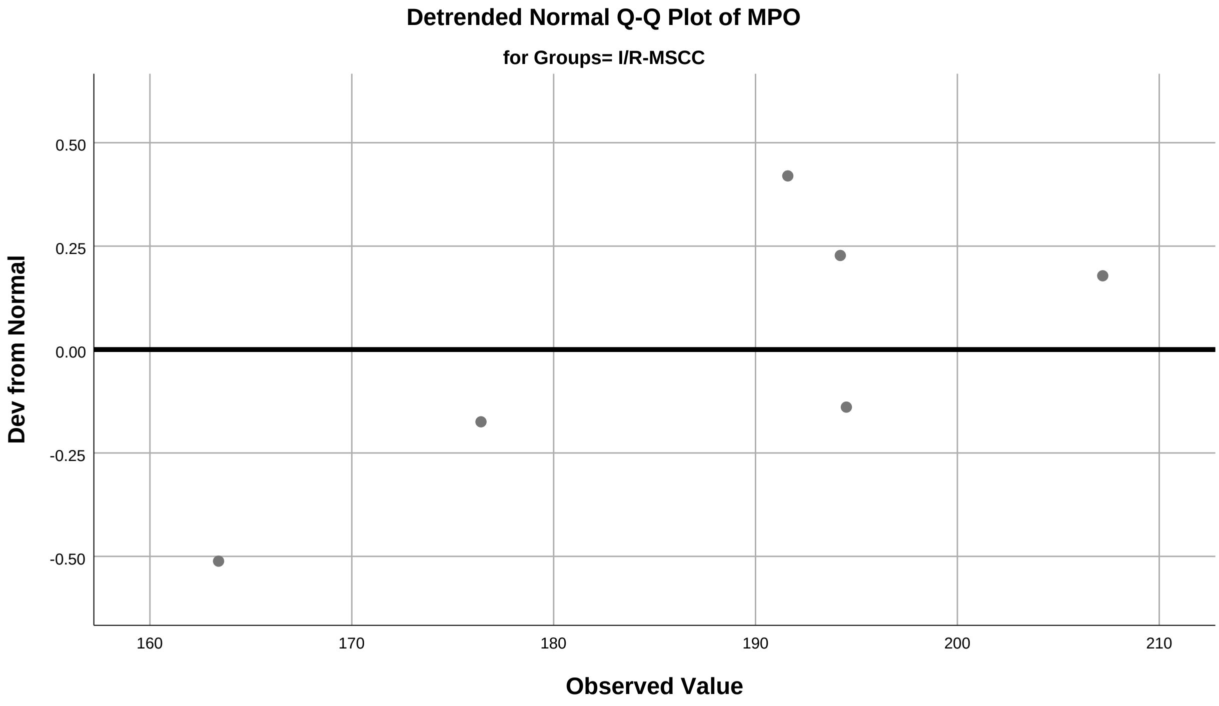
Detrended Normal Q-Q Plot of MPO
for Groups= I/R-MSCC
Dev from Normal
0.50
0.25
0.00
-0.25
-0.50
160
170
180
190
200
210
Observed Value

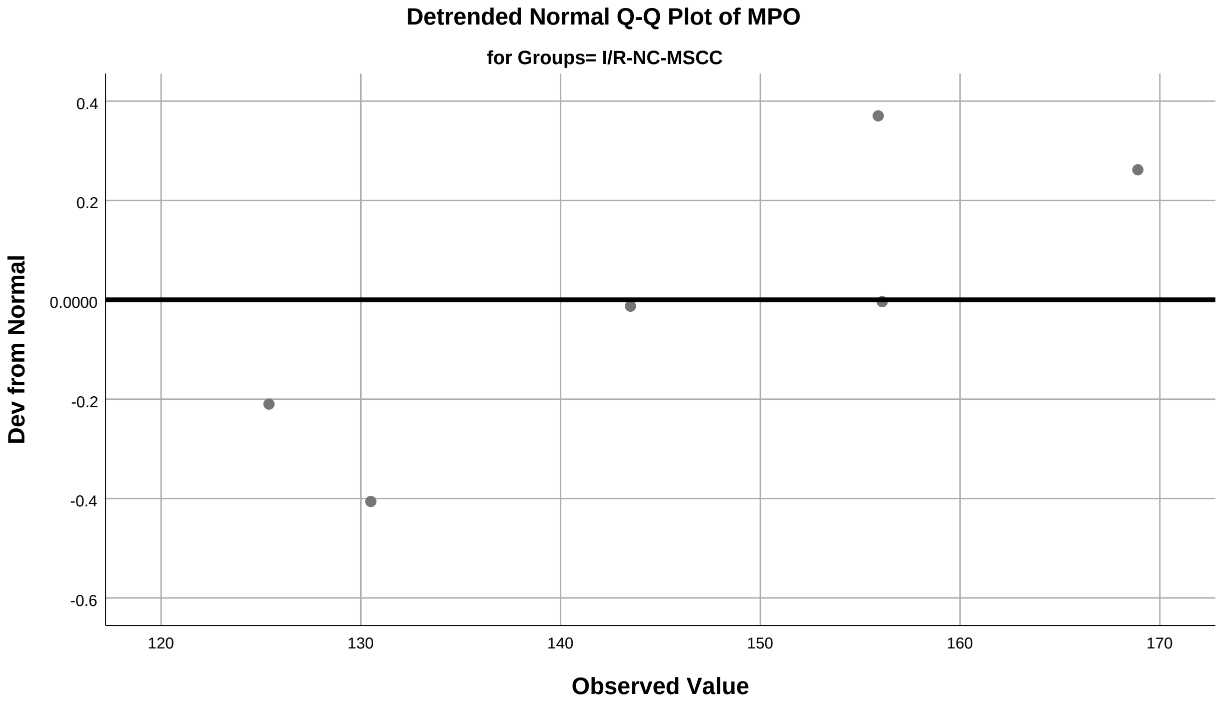
Detrended Normal Q-Q Plot of MPO
for Groups= I/R-NC-MSCC
Dev from Normal
0.4
0.2
0.0000
-0.2
-0.4
-0.6
120
130
140
150
160
170
Observed Value

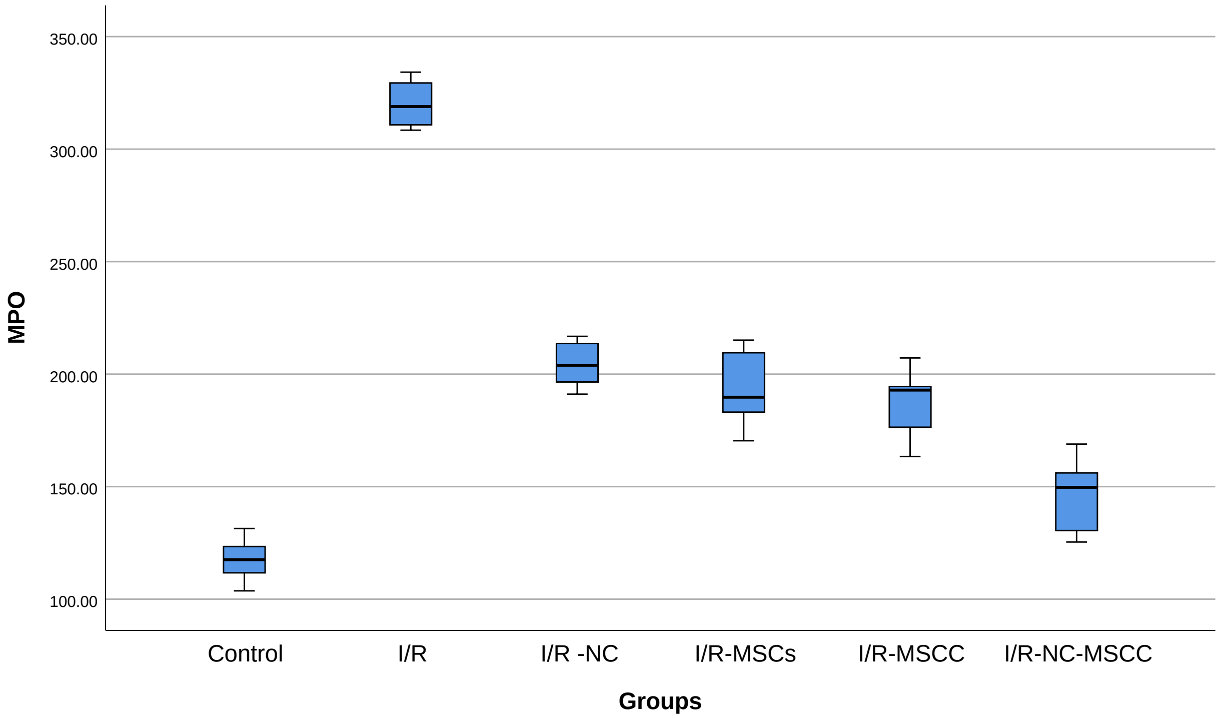

TNF

Normal Q-Q Plots

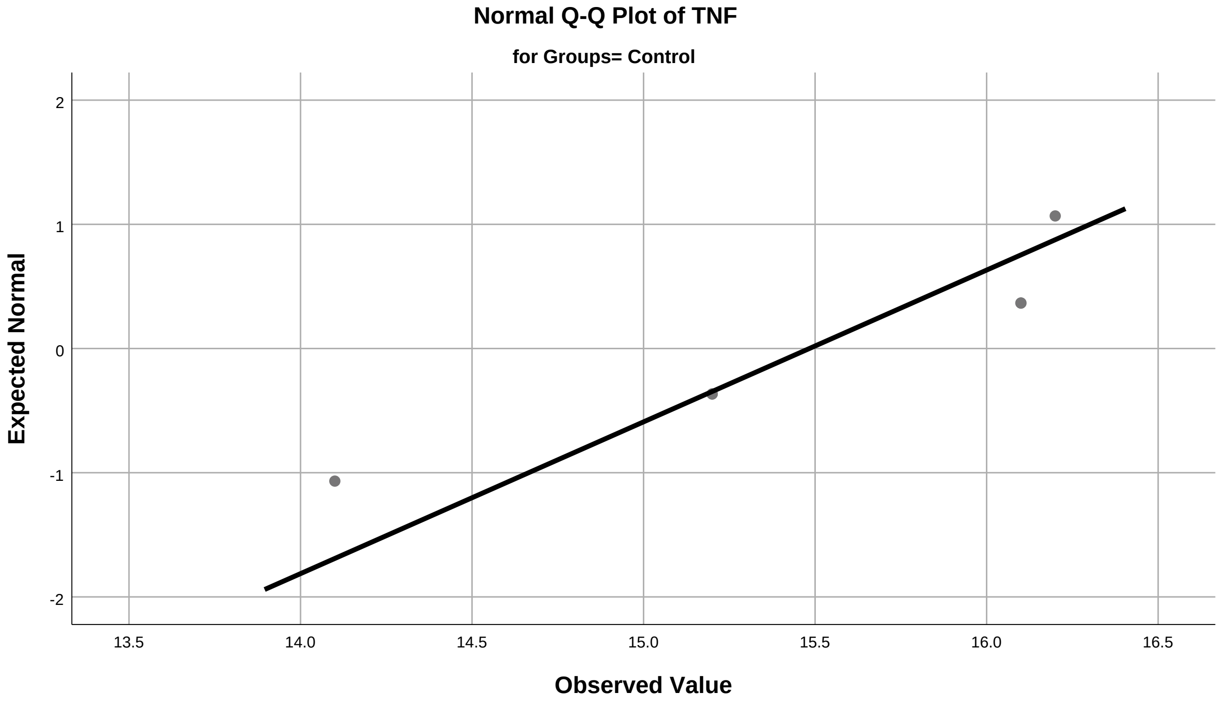

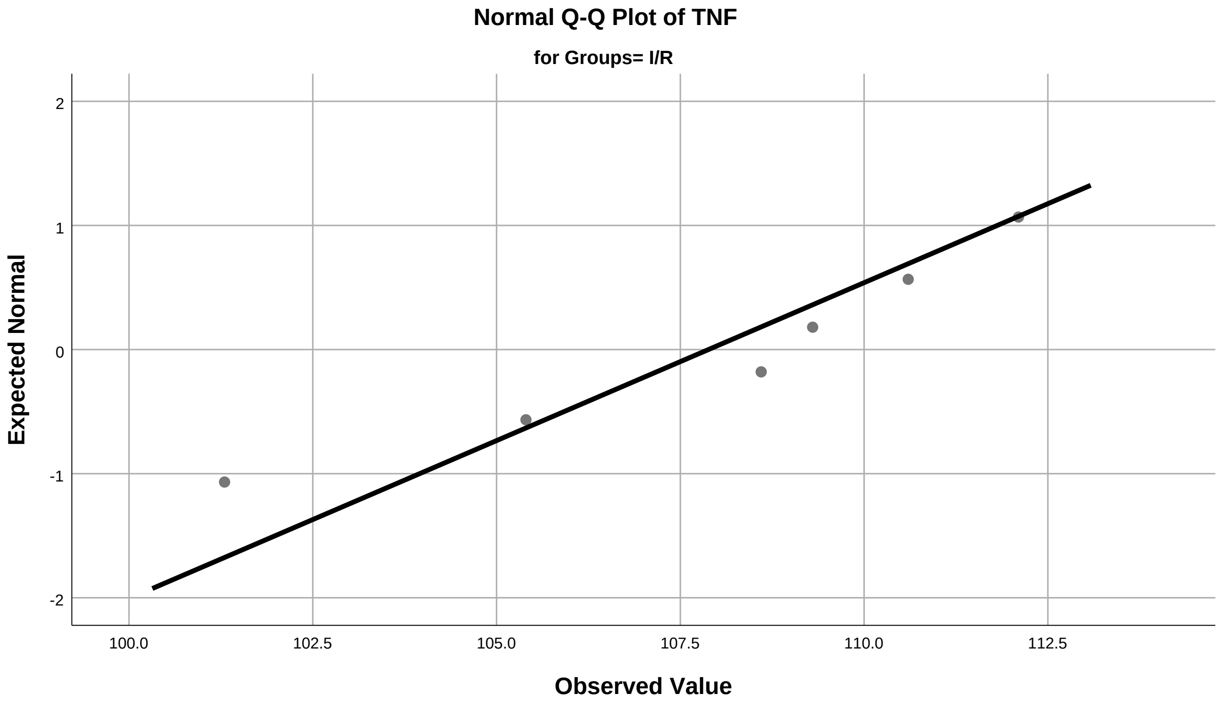
Normal Q-Q Plot of TNF
for Groups= I/R
Expected Normal
2
1
0
-1
-2
100.0
102.5
105.0
107.5
110.0
112.5
Observed Value

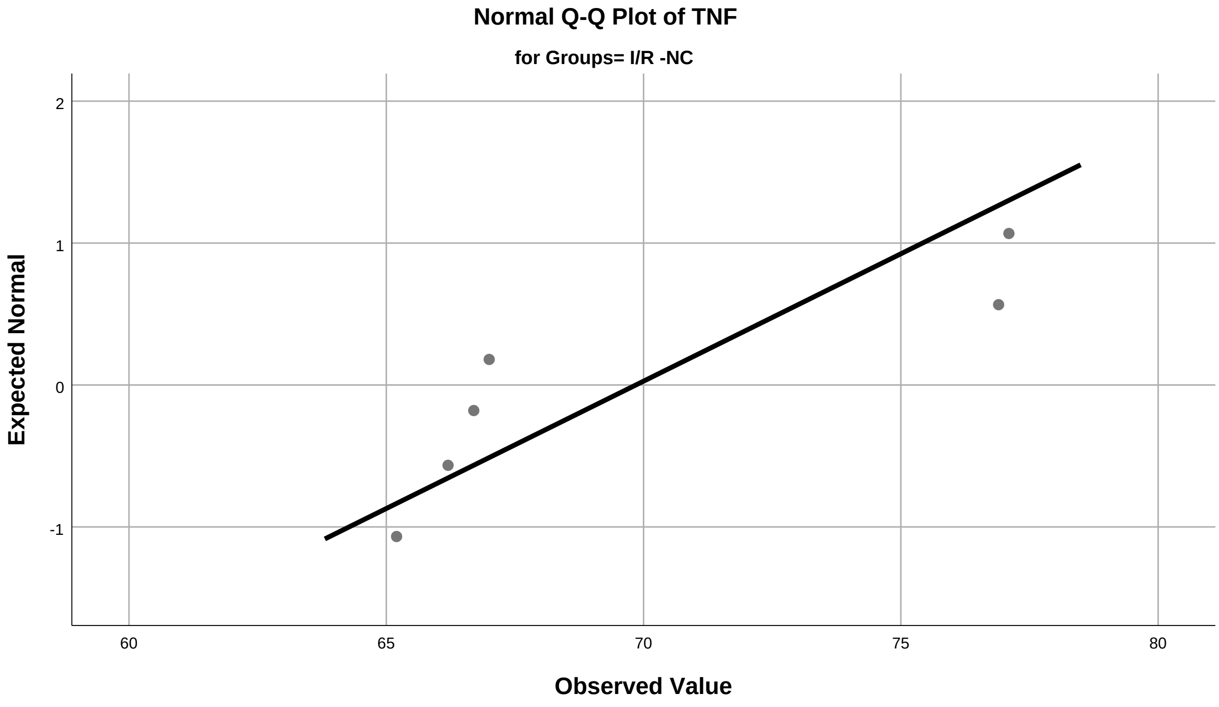
Normal Q-Q Plot of TNF
for Groups= I/R -NC
Expected Normal
2
1
0
-1
60
65
70
75
80
Observed Value

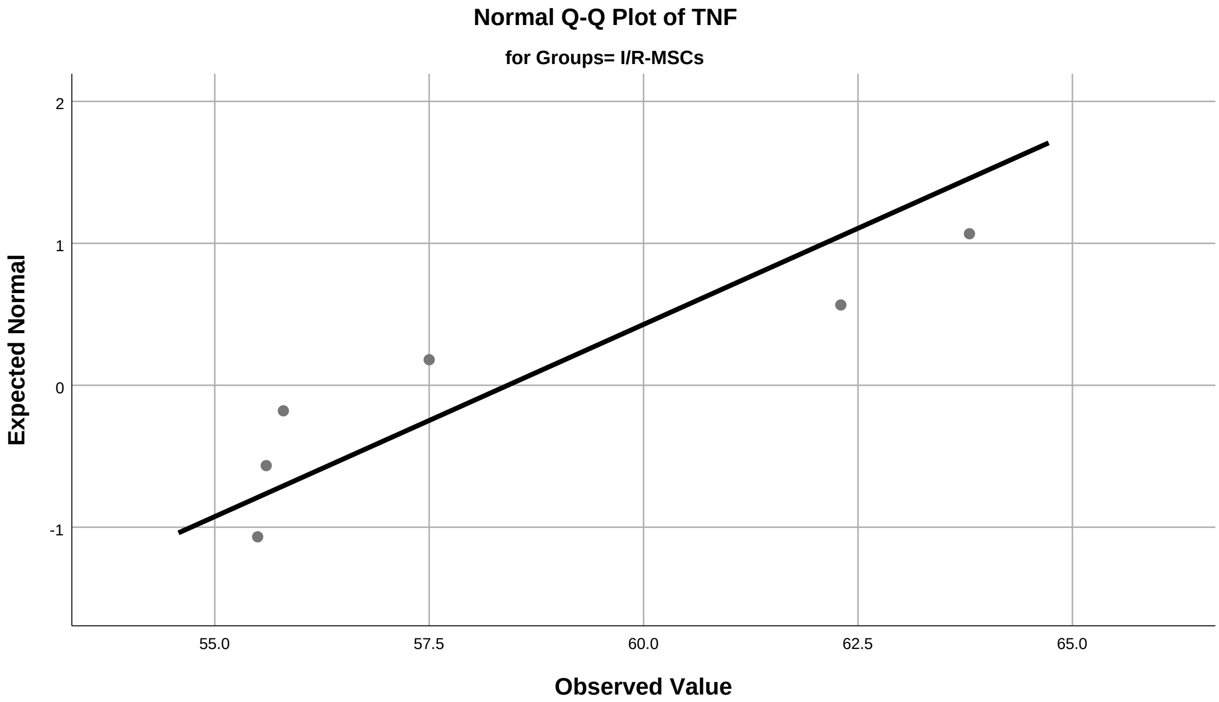
Normal Q-Q Plot of TNF
for Groups= I/R-MSCs
Expected Normal
2
1
0
-1
55.0
57.5
60.0
62.5
65.0
Observed Value

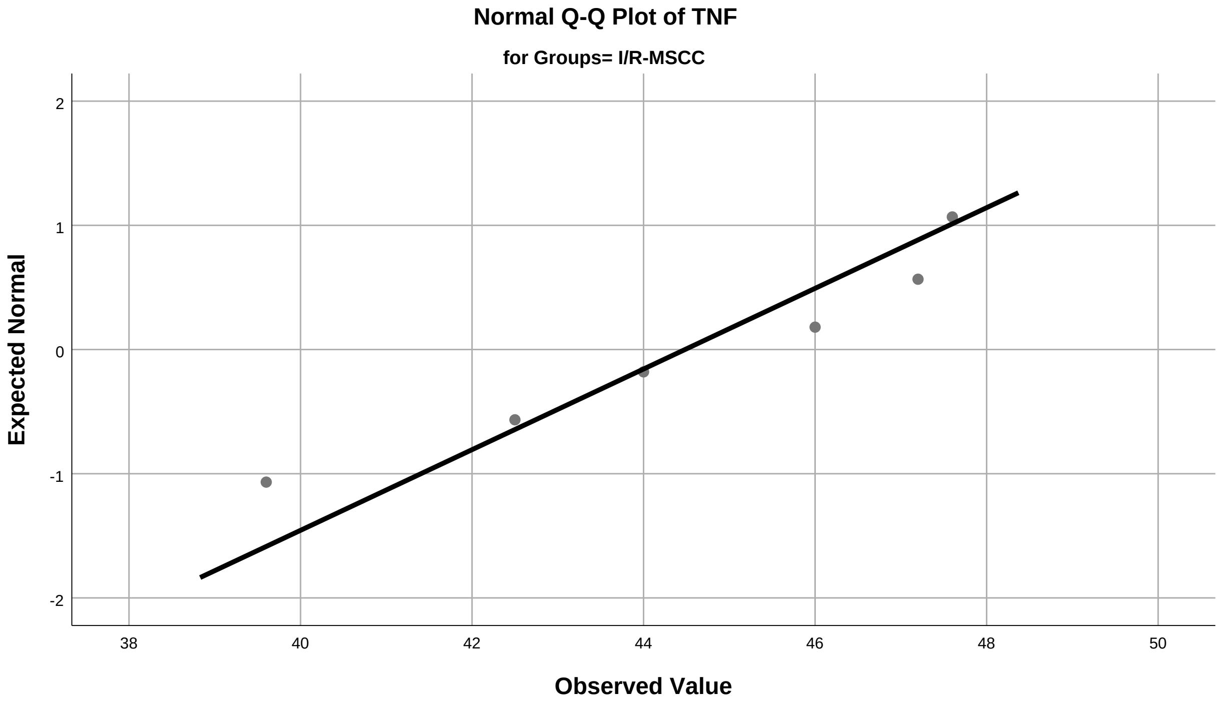
Normal Q-Q Plot of TNF
for Groups= I/R-MSCC
Expected Normal
2
1
0
-1
-2
38
40
42
44
46
48
50
Observed Value

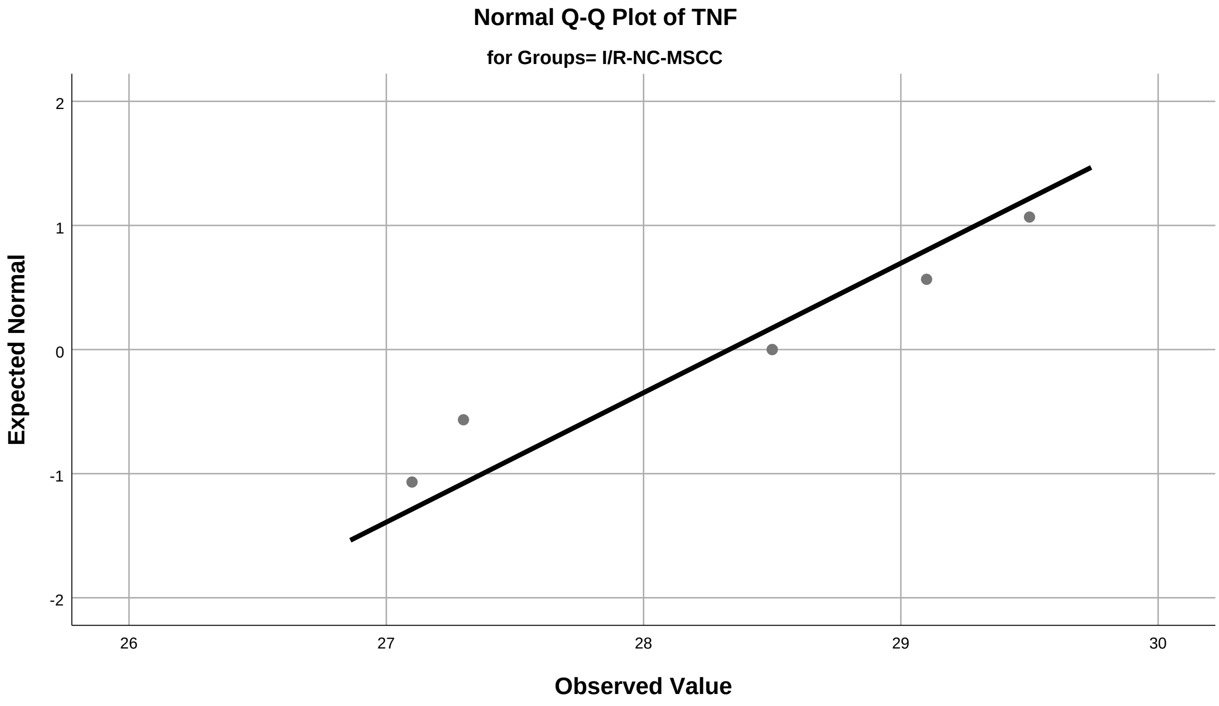

Detrended Normal Q-Q Plots

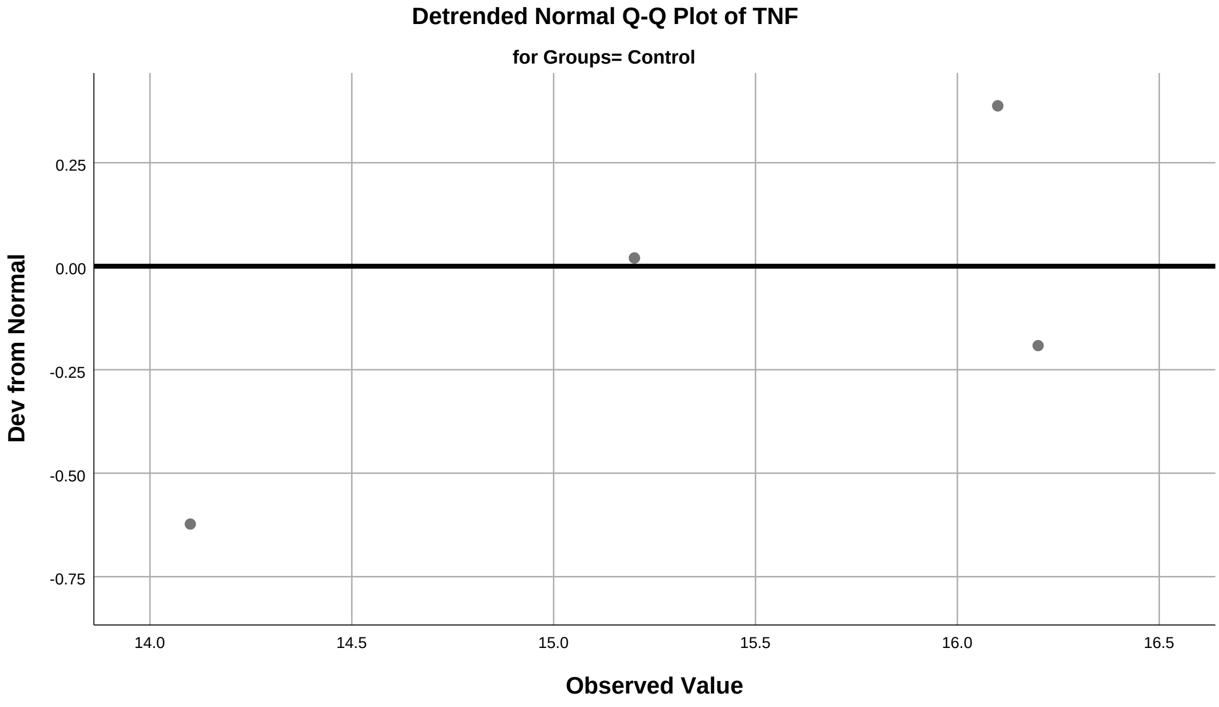

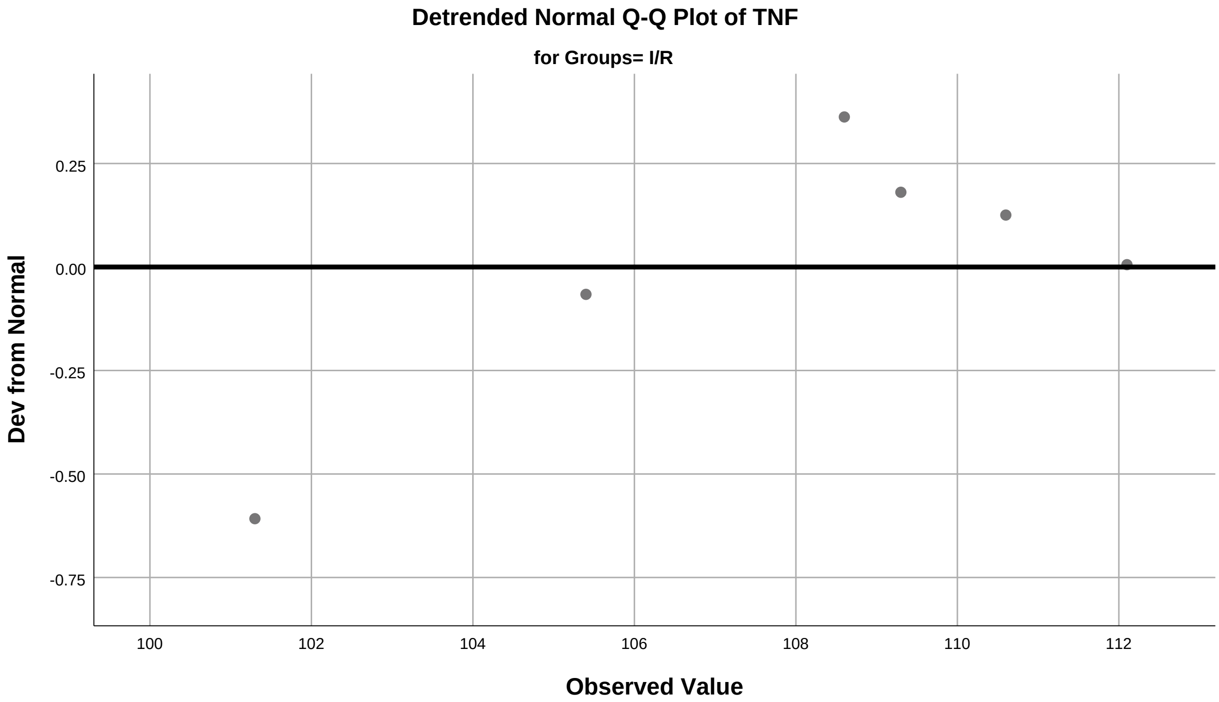
Detrended Normal Q-Q Plot of TNF
for Groups= I/R
Dev from Normal
0.25
0.00
-0.25
-0.50
-0.75
100
102
104
106
108
110
112
Observed Value

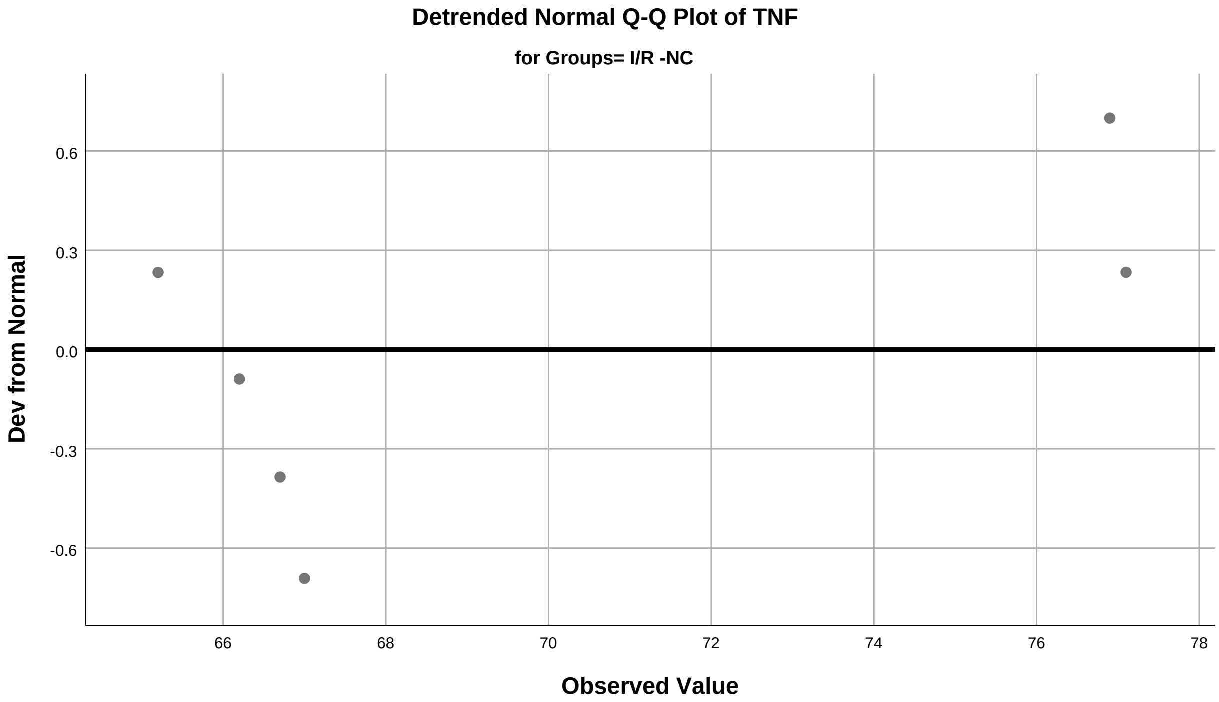
Detrended Normal Q-Q Plot of TNF
for Groups= I/R -NC
Dev from Normal
0.6
0.3
0.0
-0.3
-0.6
66
68
70
72
74
76
78
Observed Value

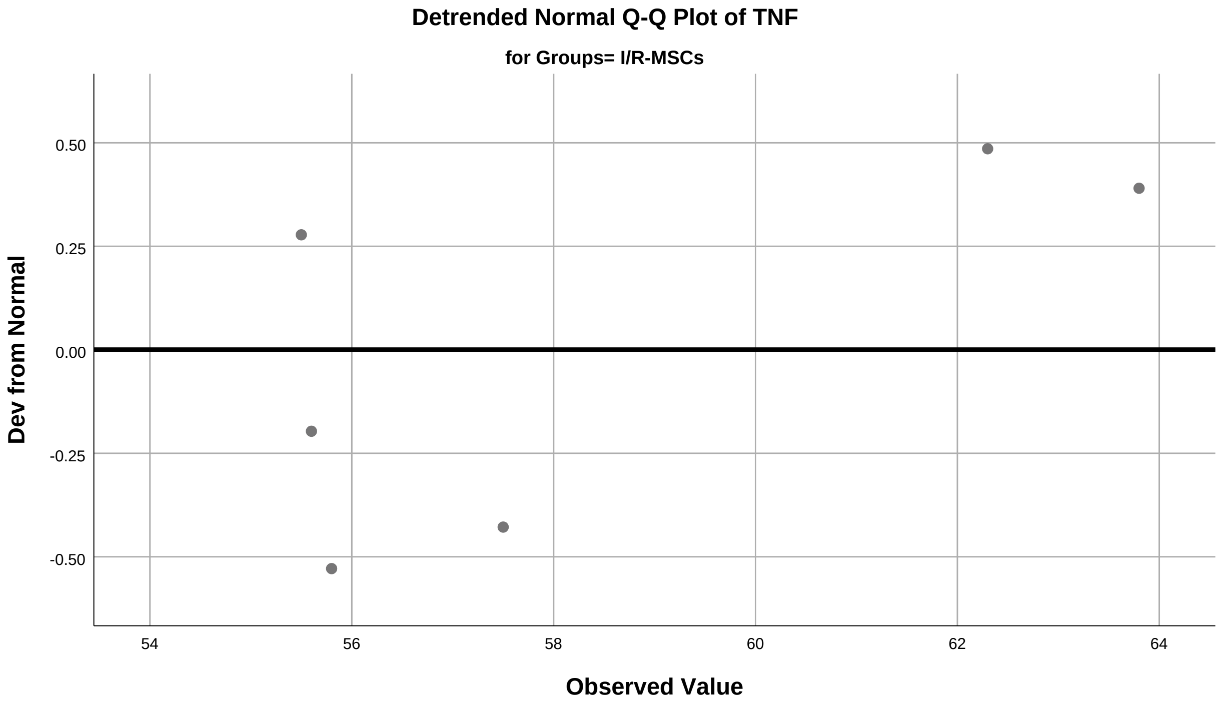
Detrended Normal Q-Q Plot of TNF
for Groups= I/R-MSCs
Dev from Normal
0.50
0.25
0.00
-0.25
-0.50
54
56
58
60
62
64
Observed Value

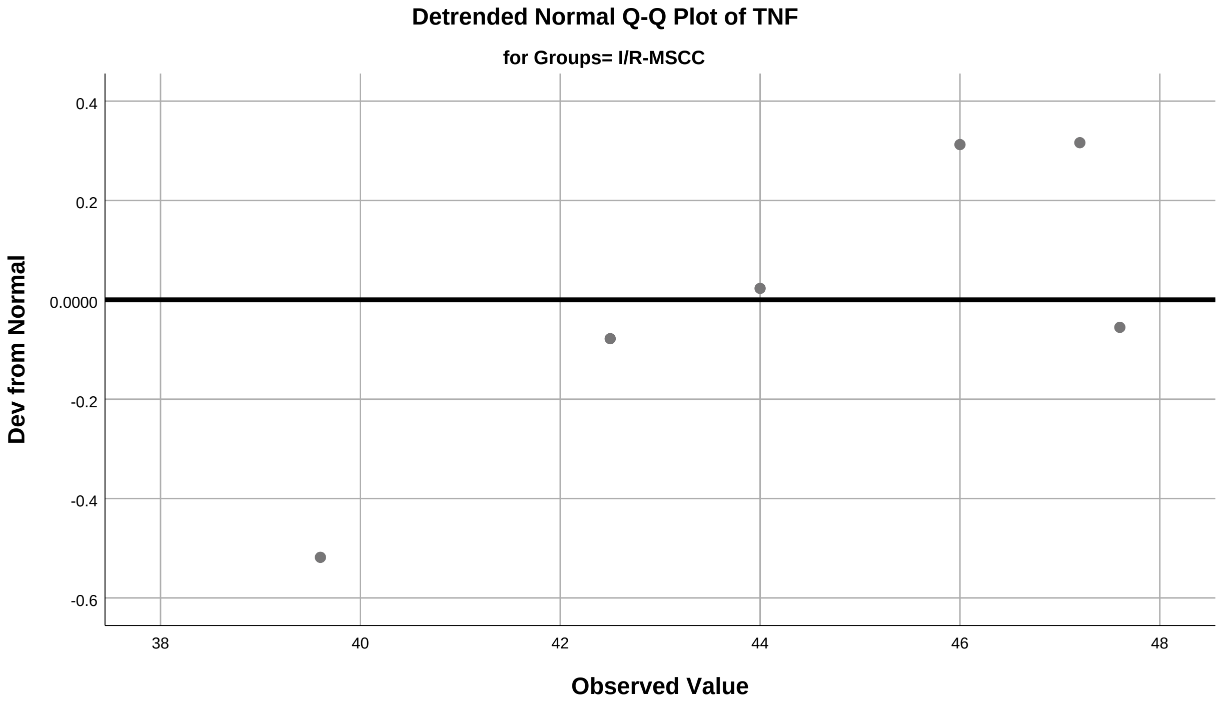
Detrended Normal Q-Q Plot of TNF
for Groups= I/R-MSCC
Dev from Normal
0.4
0.2
0.0000
-0.2
-0.4
-0.6
38
40
42
44
46
48
Observed Value

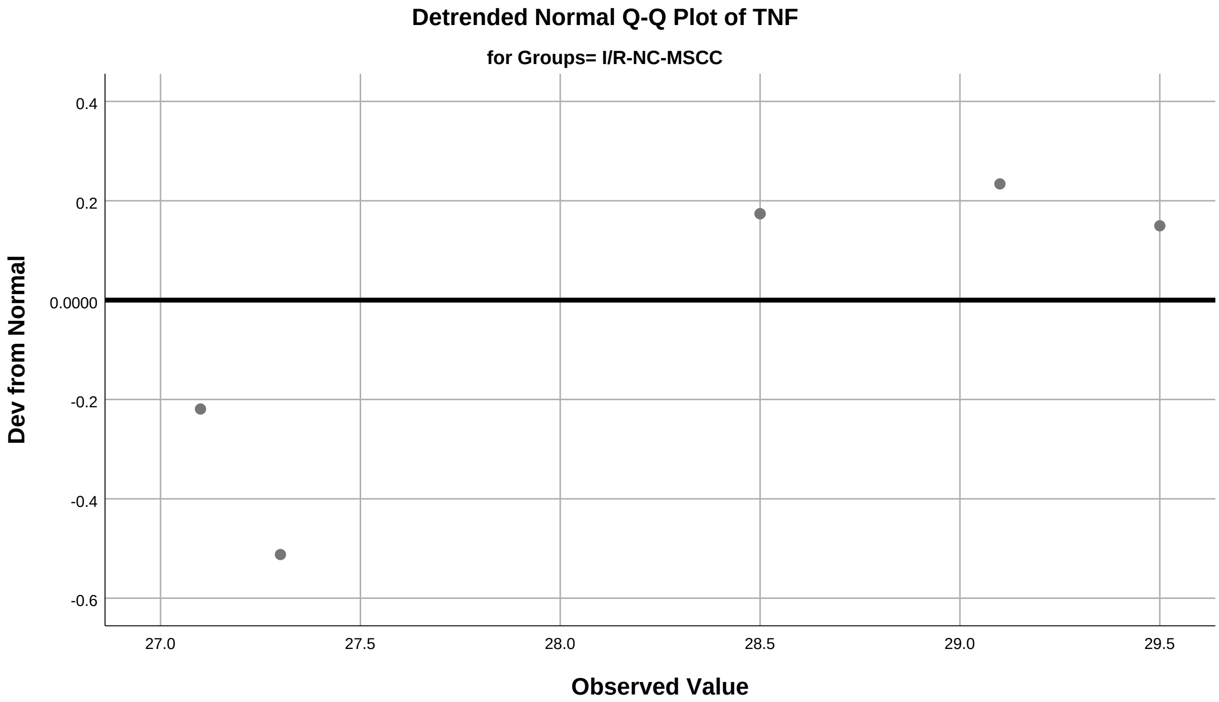

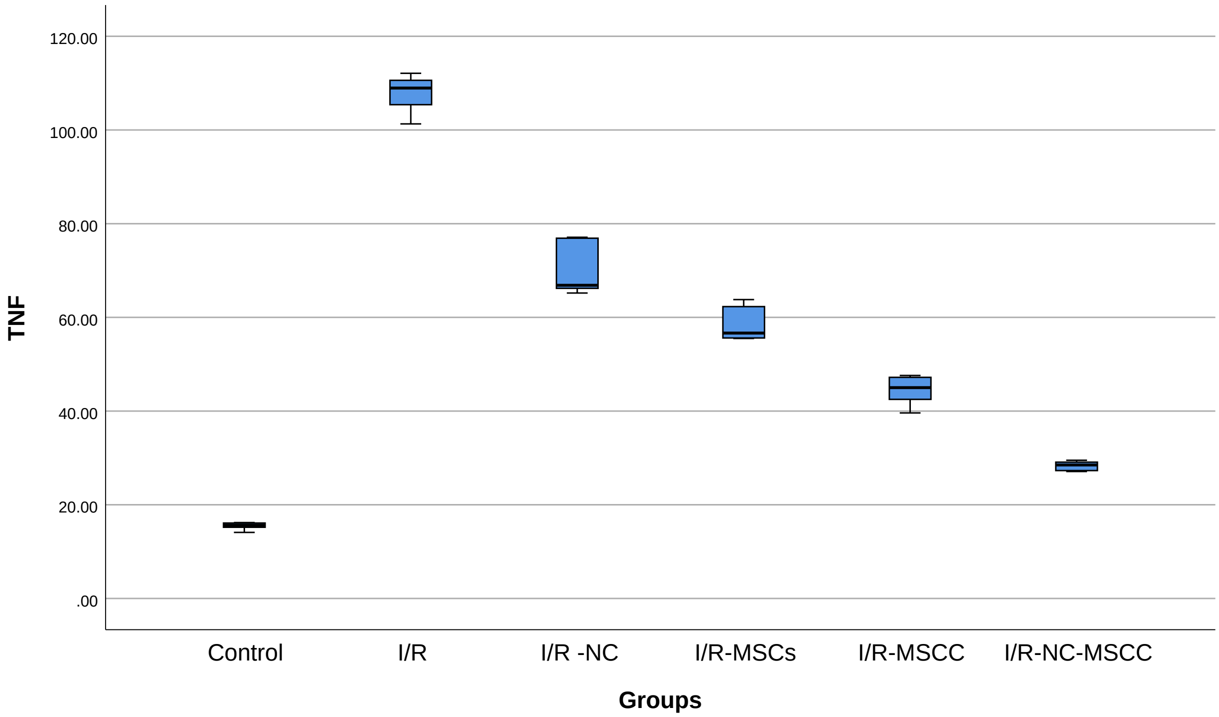

Bax

Normal Q-Q Plots

Normal Q-Q Plot of Bax

for Groups= Control

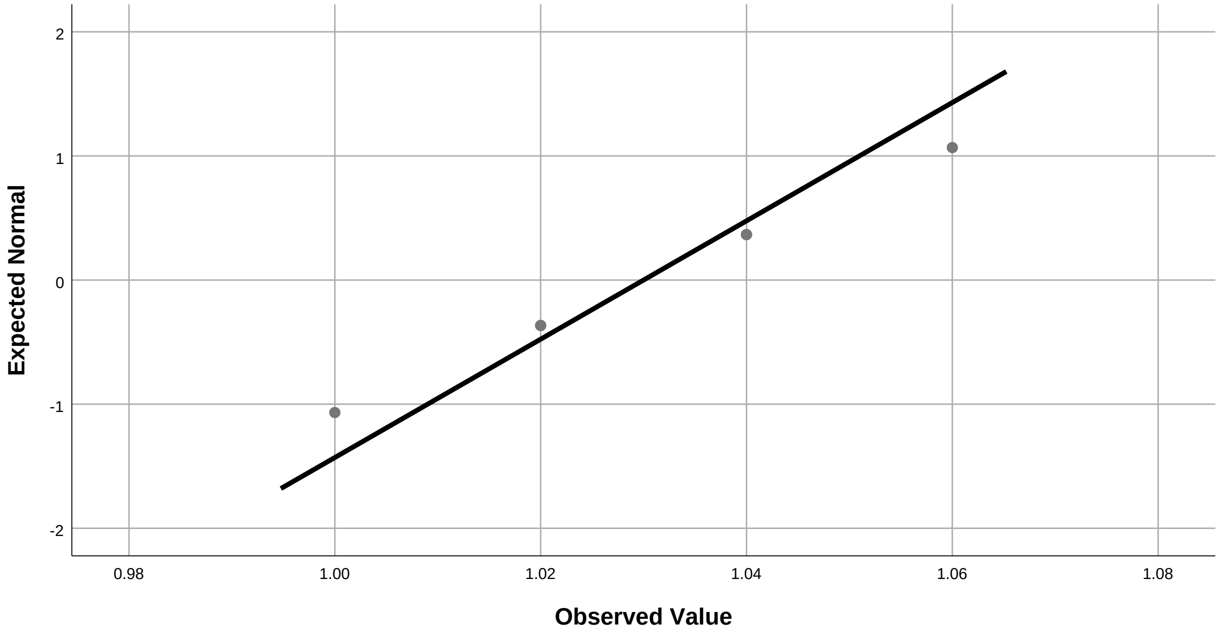

Normal Q-Q Plot of Bax

for Groups= I/R

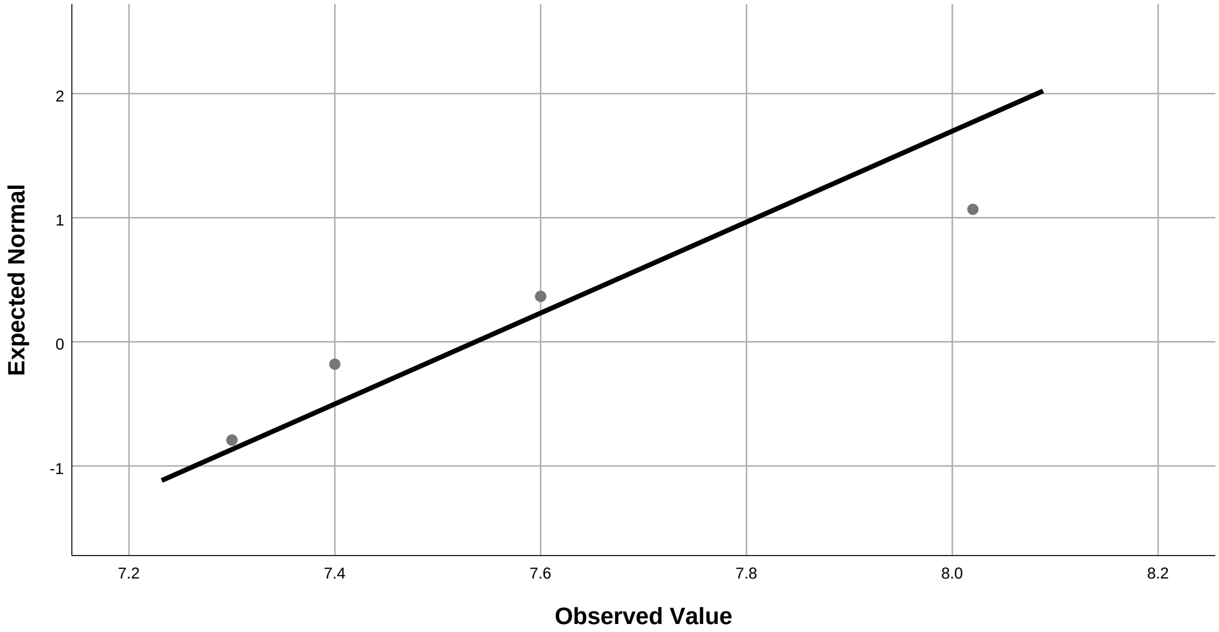

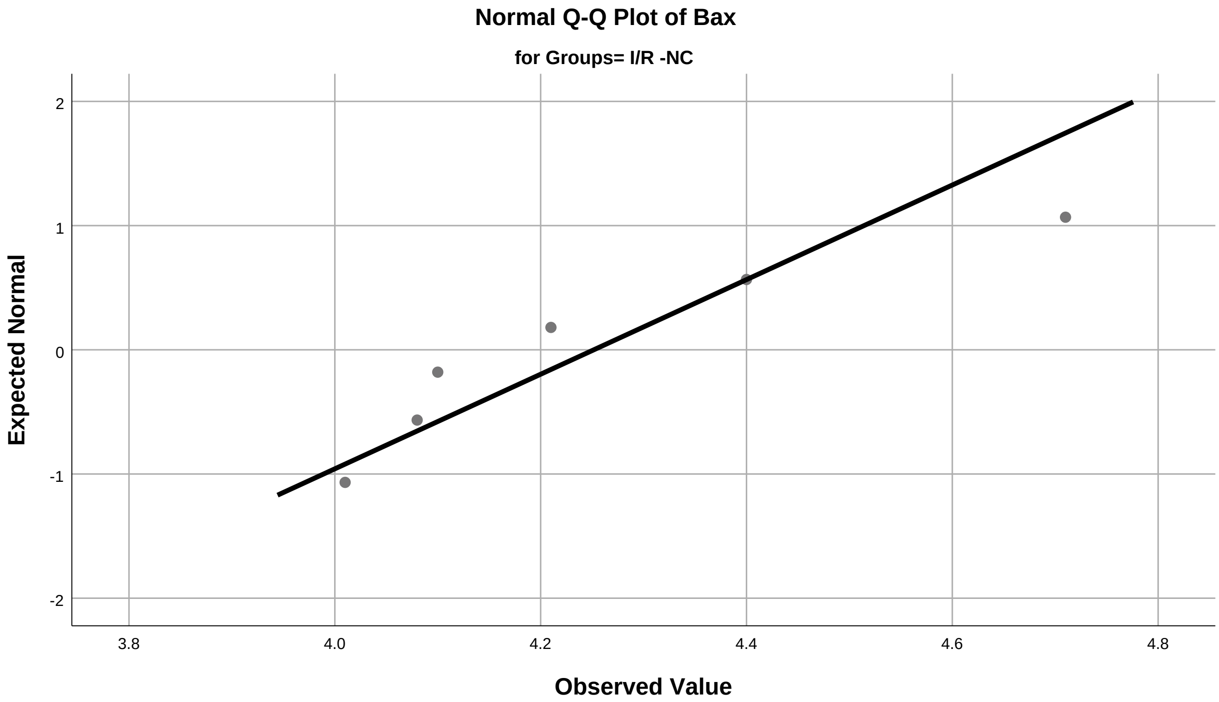
Normal Q-Q Plot of Bax
for Groups= I/R -NC
Expected Normal
2
1
0
-1
-2
3.8
4.0
4.2
4.4
4.6
4.8
Observed Value

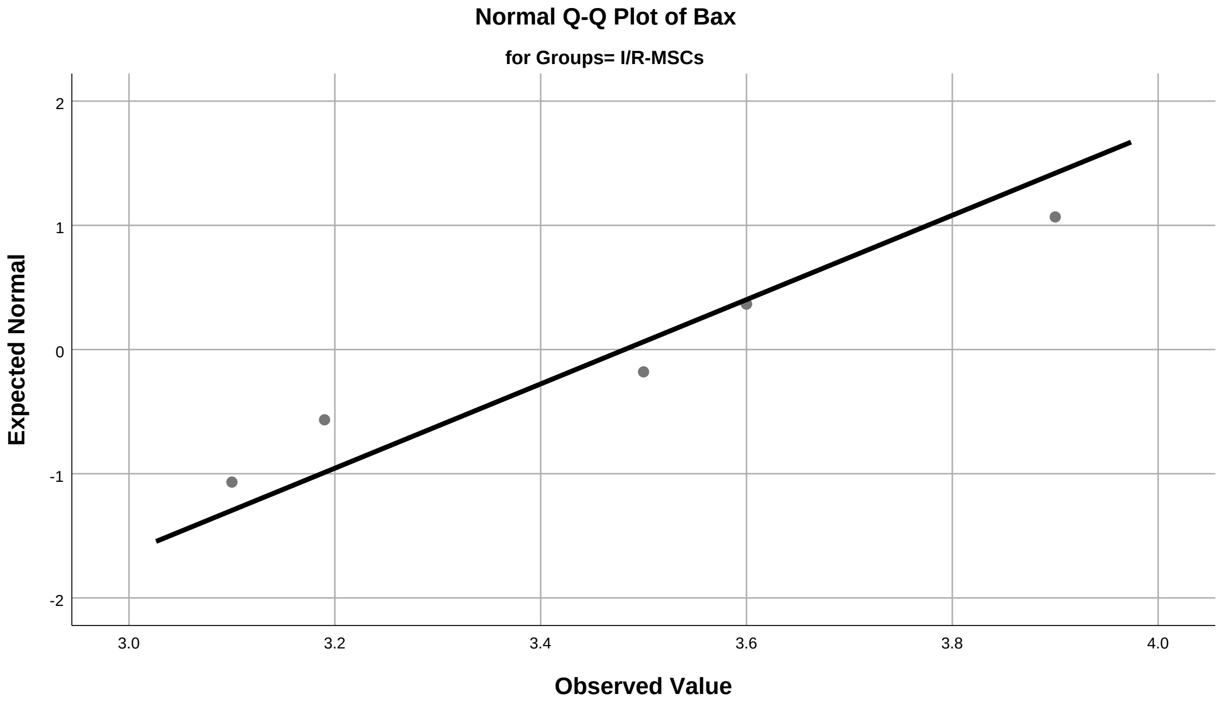
Normal Q-Q Plot of Bax
for Groups= I/R-MSCs
Expected Normal
2
1
0
-1
-2
3.0
3.2
3.4
3.6
3.8
4.0
Observed Value

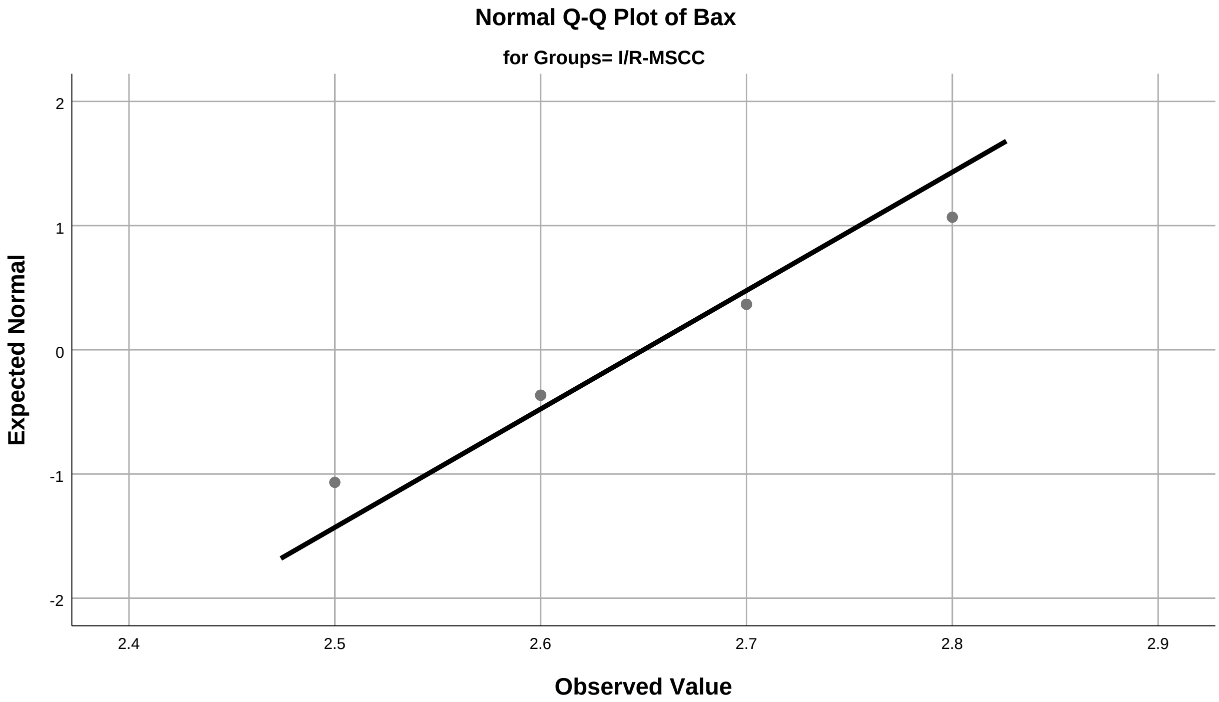

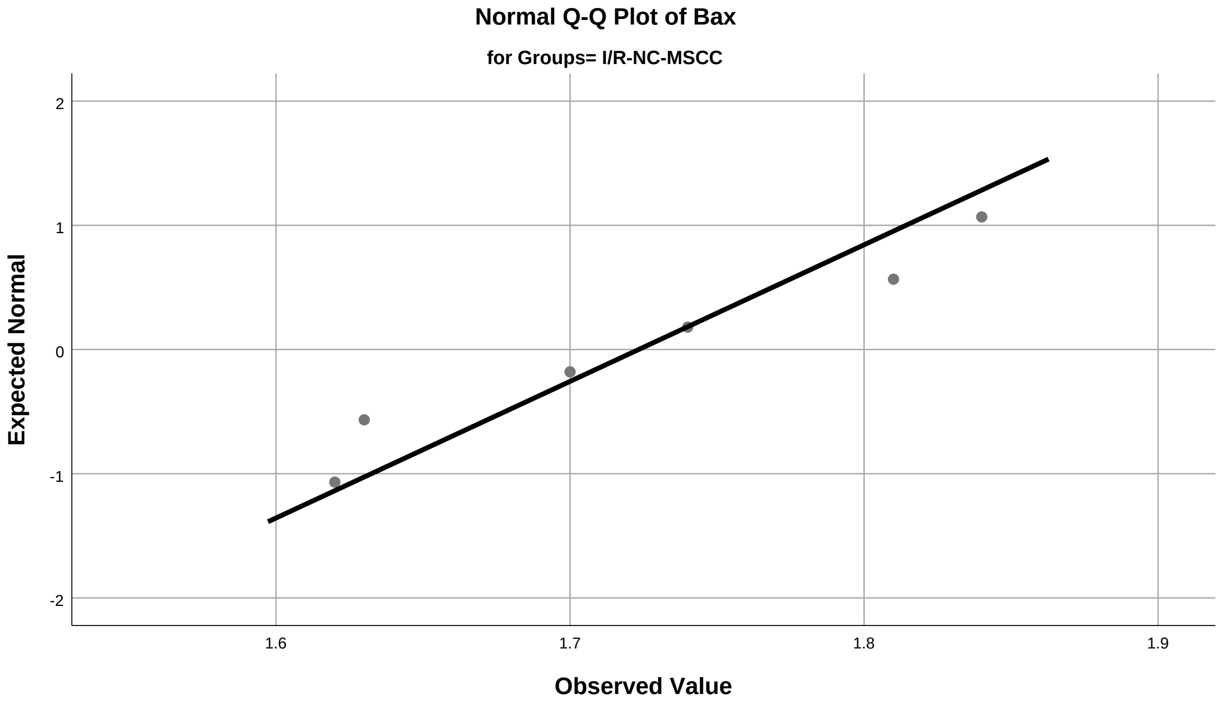

Detrended Normal Q-Q Plots

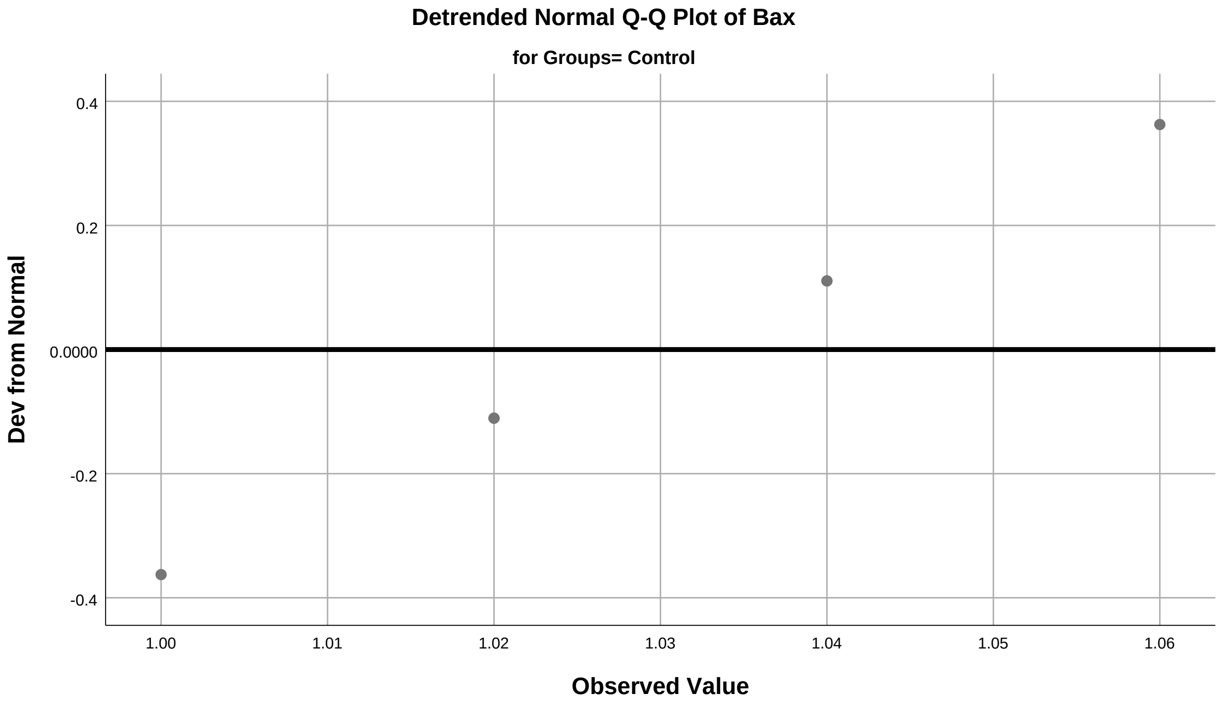
Detrended Normal Q-Q Plot of Bax
for Groups= Control
Dev from Normal
0.4
0.2
0.0000
-0.2
-0.4
1.00
1.01
1.02
1.03
1.04
1.05
1.06
Observed Value

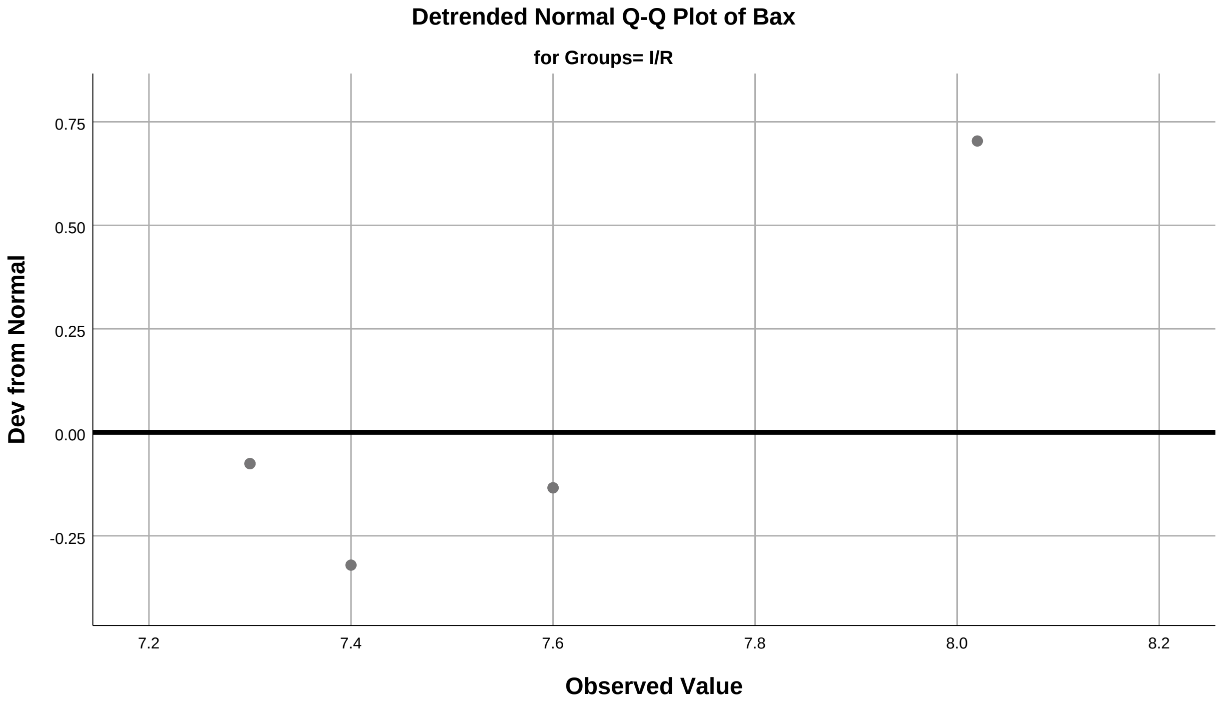
Detrended Normal Q-Q Plot of Bax
for Groups= I/R
Dev from Normal
0.75
0.50
0.25
0.00
-0.25
7.2
7.4
7.6
7.8
8.0
8.2
Observed Value

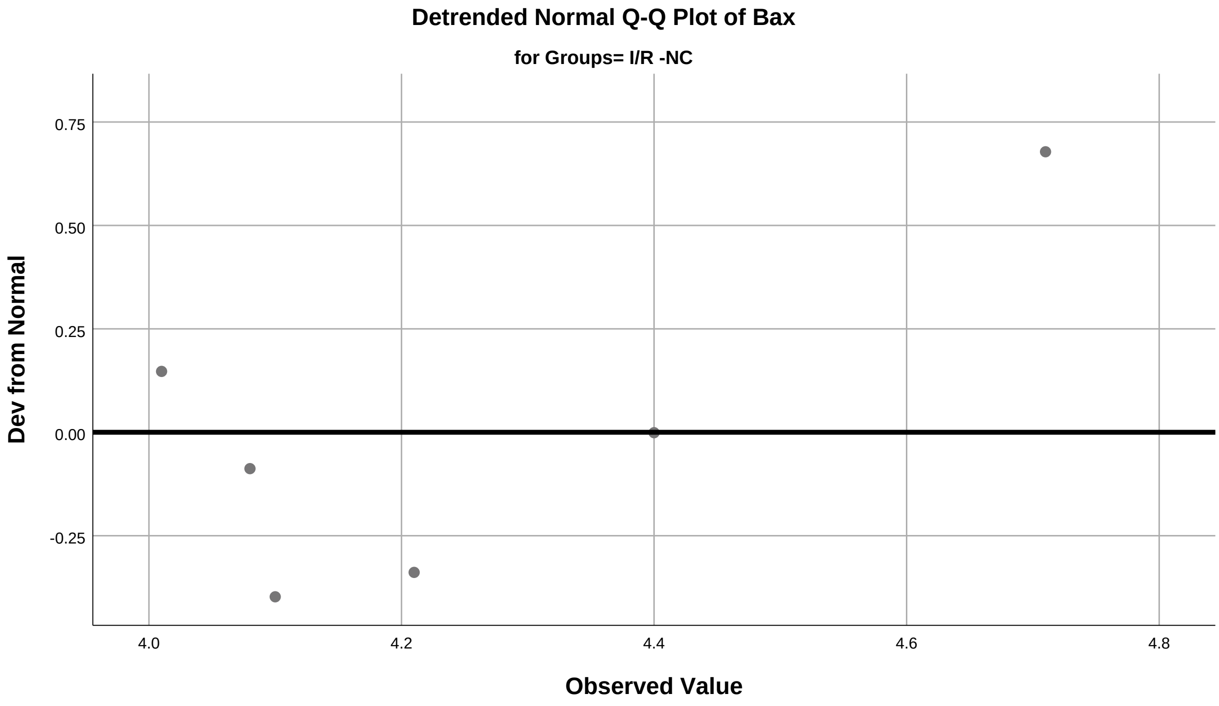

Detrended Normal Q-Q Plot of Bax
for Groups= I/R -NC
Dev from Normal
0.75
0.50
0.25
0.00
-0.25
4.0
4.2
4.4
4.6
4.8
Observed Value

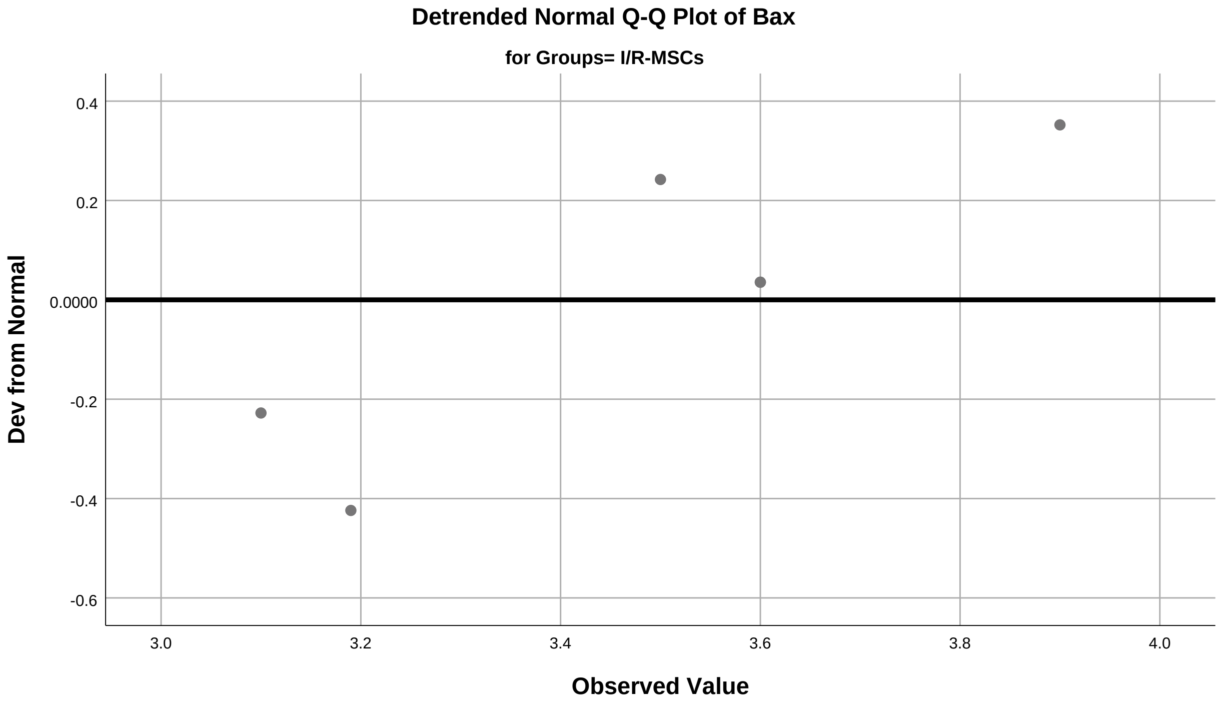

Detrended Normal Q-Q Plot of Bax
for Groups= I/R-MSCs
Dev from Normal
0.4
0.2
0.0000
-0.2
-0.4
-0.6
3.0
3.2
3.4
3.6
3.8
4.0
Observed Value

Detrended Normal Q-Q Plot of Bax

for Groups= I/R-MSCC

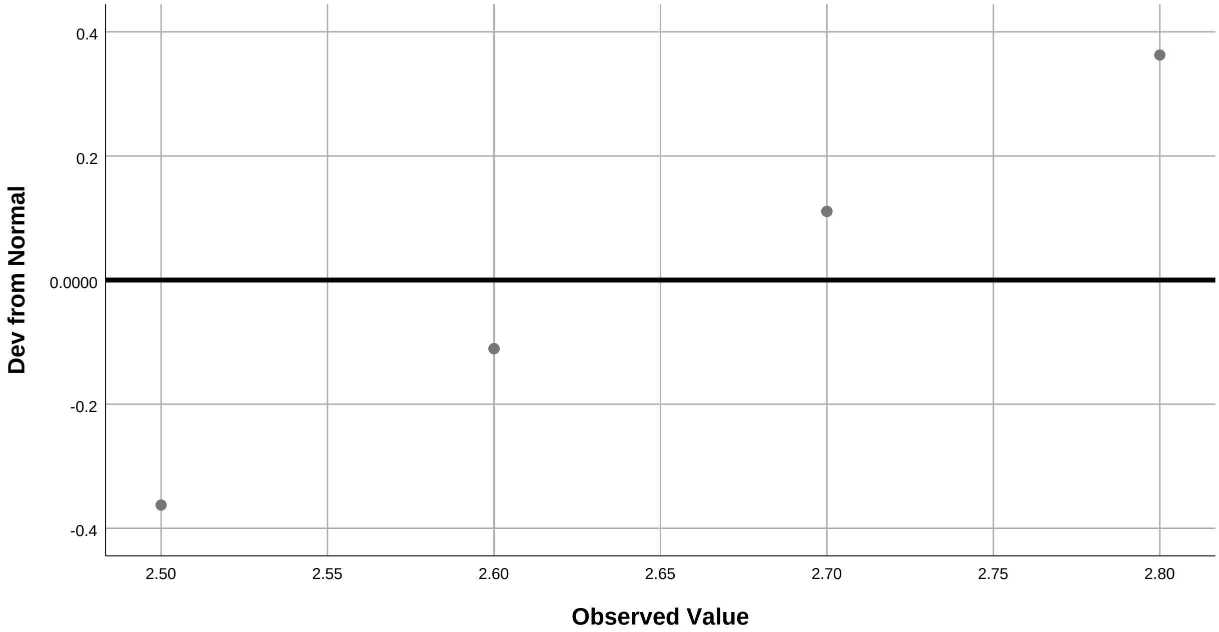

Detrended Normal Q-Q Plot of Bax

for Groups= I/R-NC-MSCC

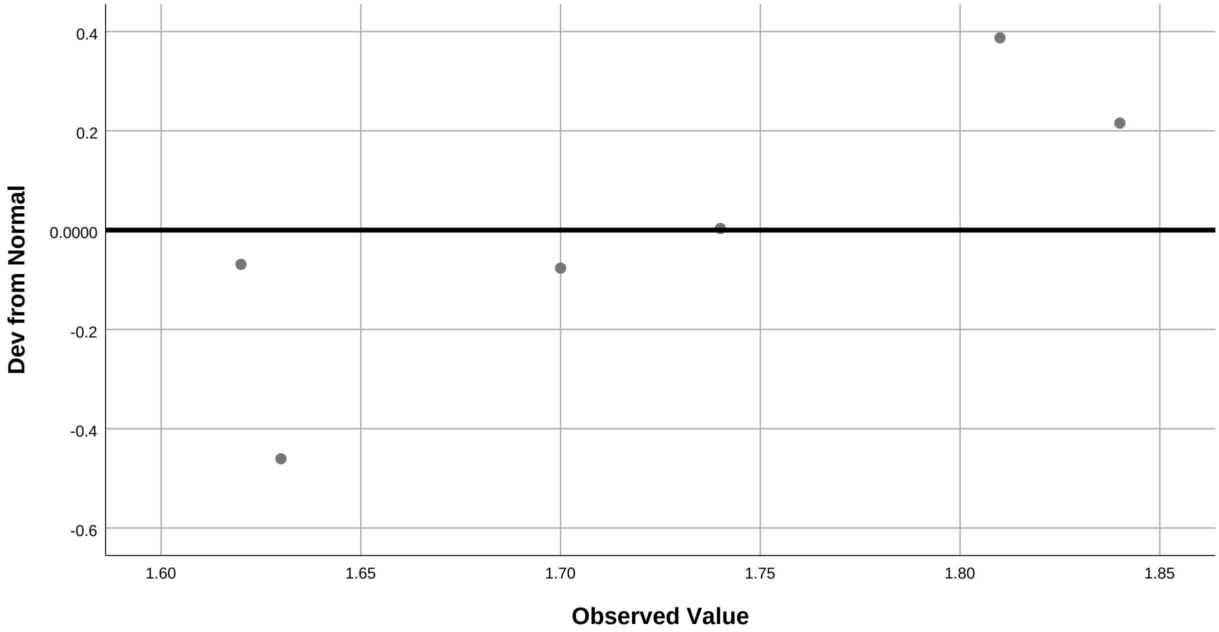

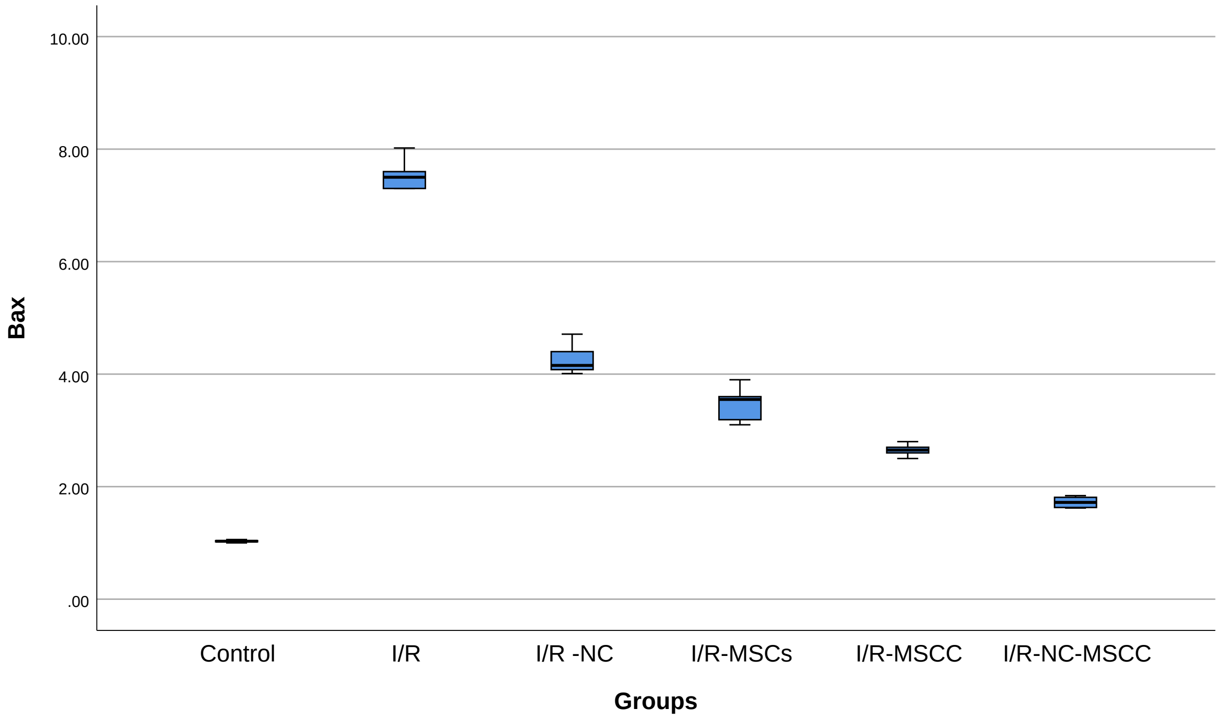

BCL2

Normal Q-Q Plots

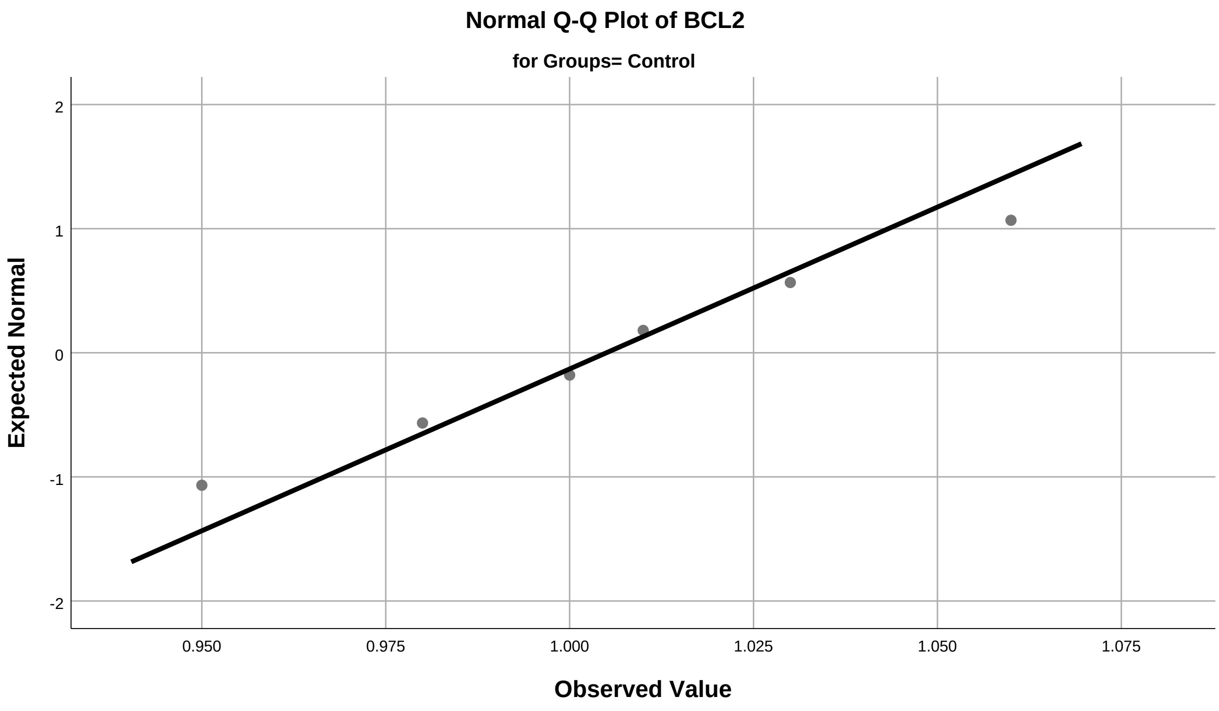

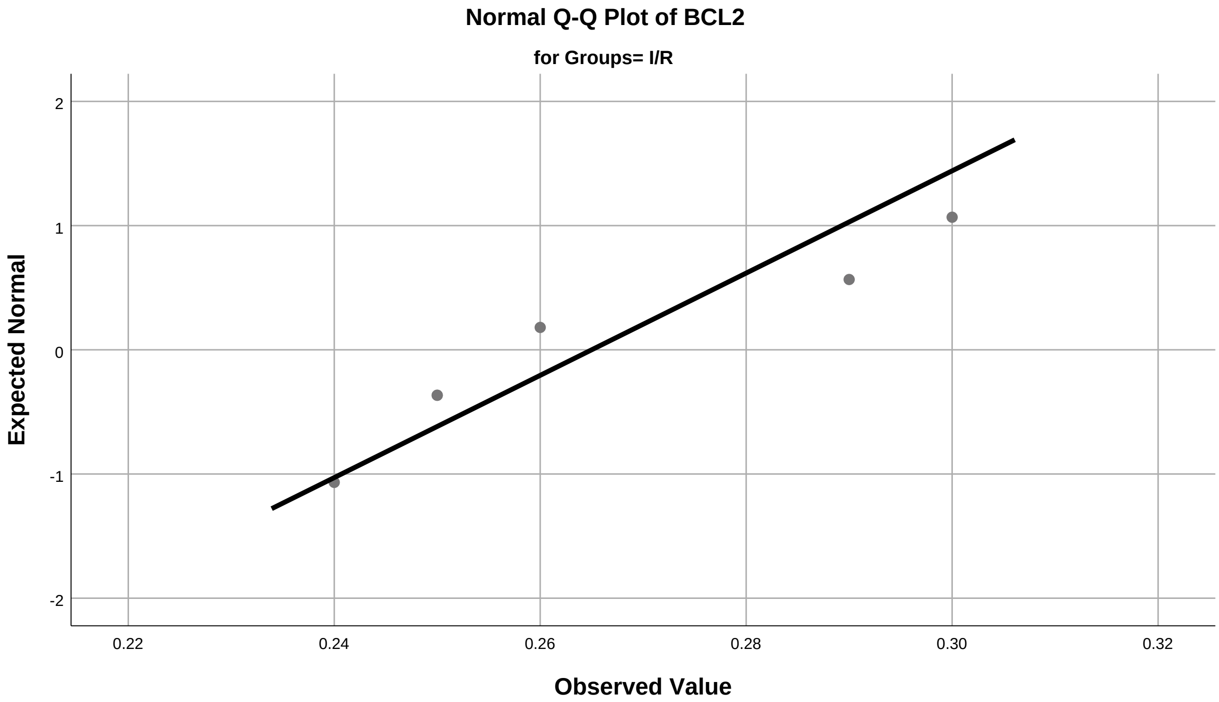
Normal Q-Q Plot of BCL2
for Groups= I/R
Expected Normal
2
1
0
-1
-2
0.22
0.24
0.26
0.28
0.30
0.32
Observed Value

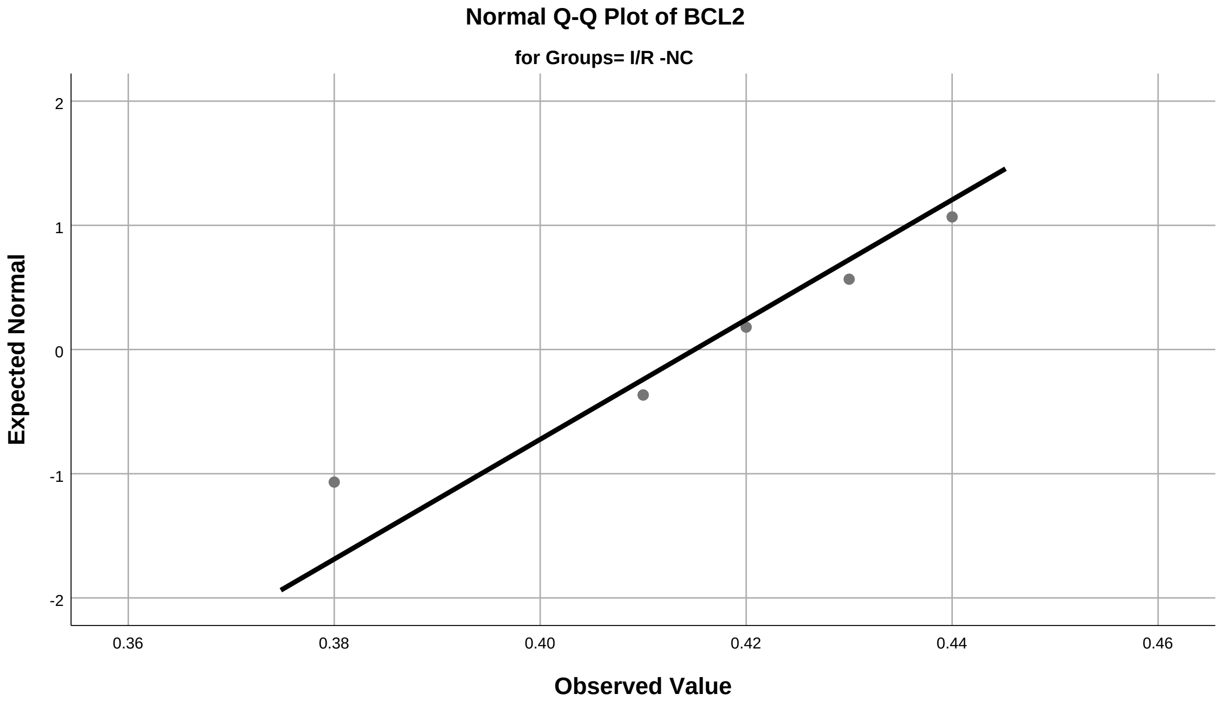
Normal Q-Q Plot of BCL2
for Groups= I/R -NC
Expected Normal
2
1
0
-1
-2
0.36
0.38
0.40
0.42
0.44
0.46
Observed Value

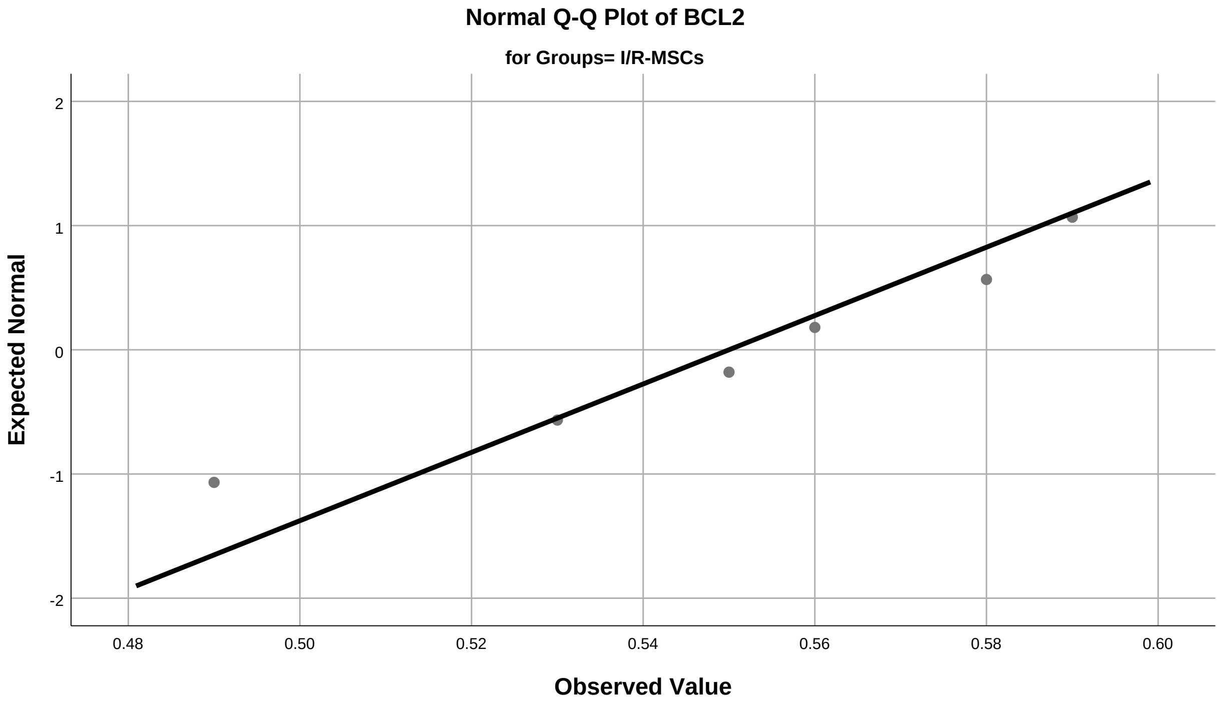
Normal Q-Q Plot of BCL2
for Groups= I/R-MSCs
Expected Normal
2
1
0
-1
-2
0.48
0.50
0.52
0.54
0.56
0.58
0.60
Observed Value

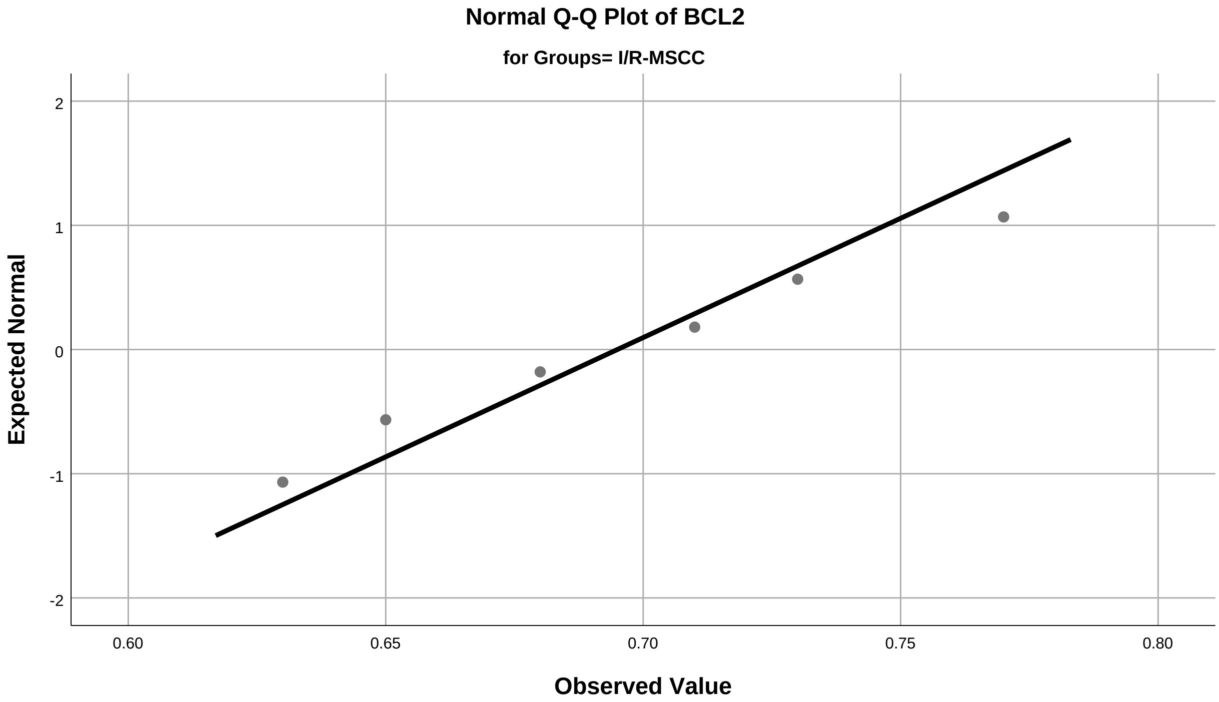
Normal Q-Q Plot of BCL2
for Groups= I/R-MSCC
Expected Normal
2
1
0
-1
-2
0.60
0.65
0.70
0.75
0.80
Observed Value

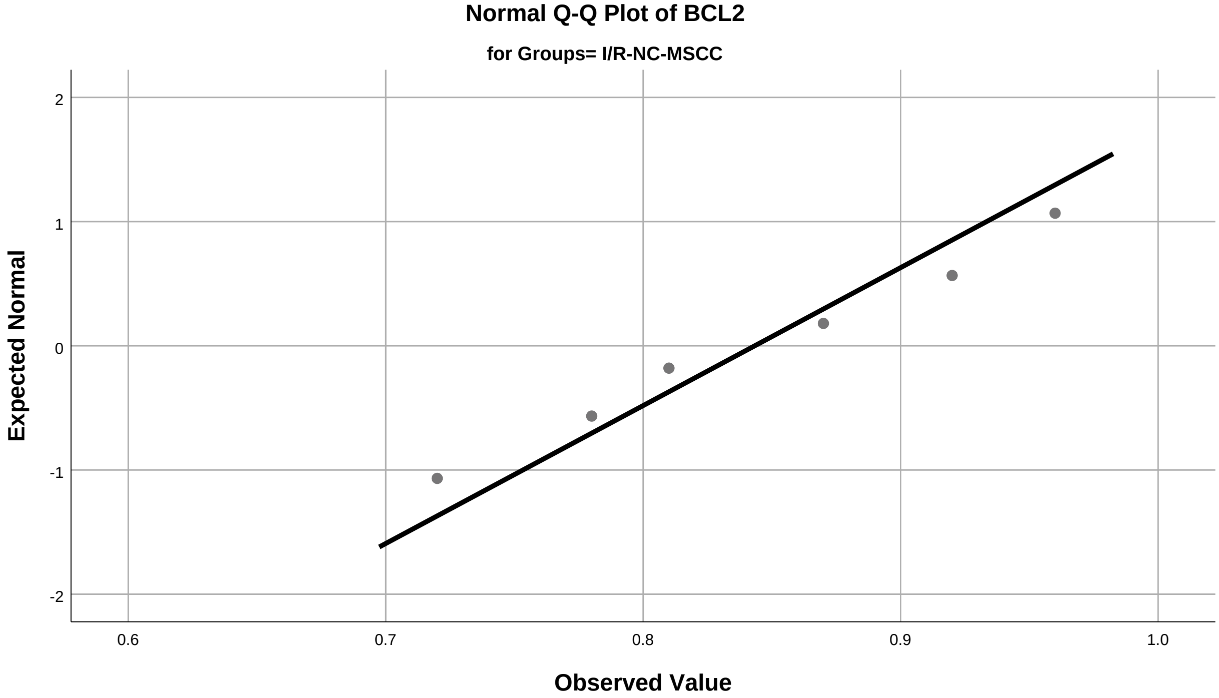

Detrended Normal Q-Q Plots

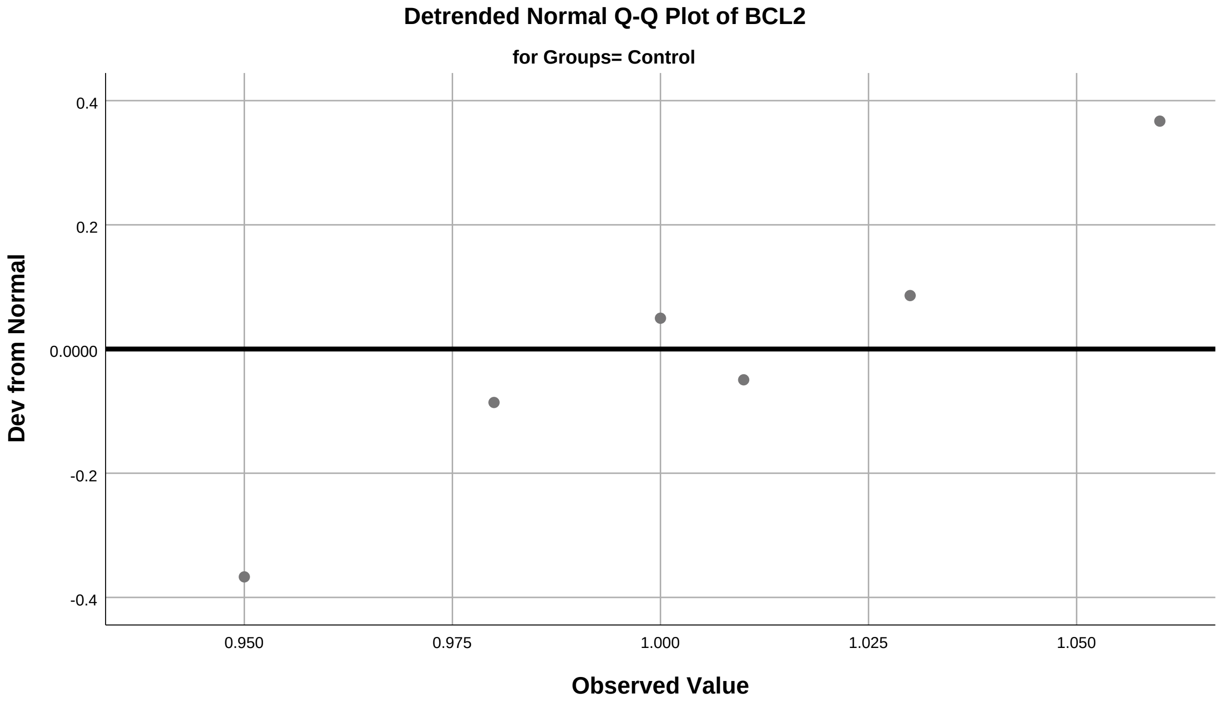

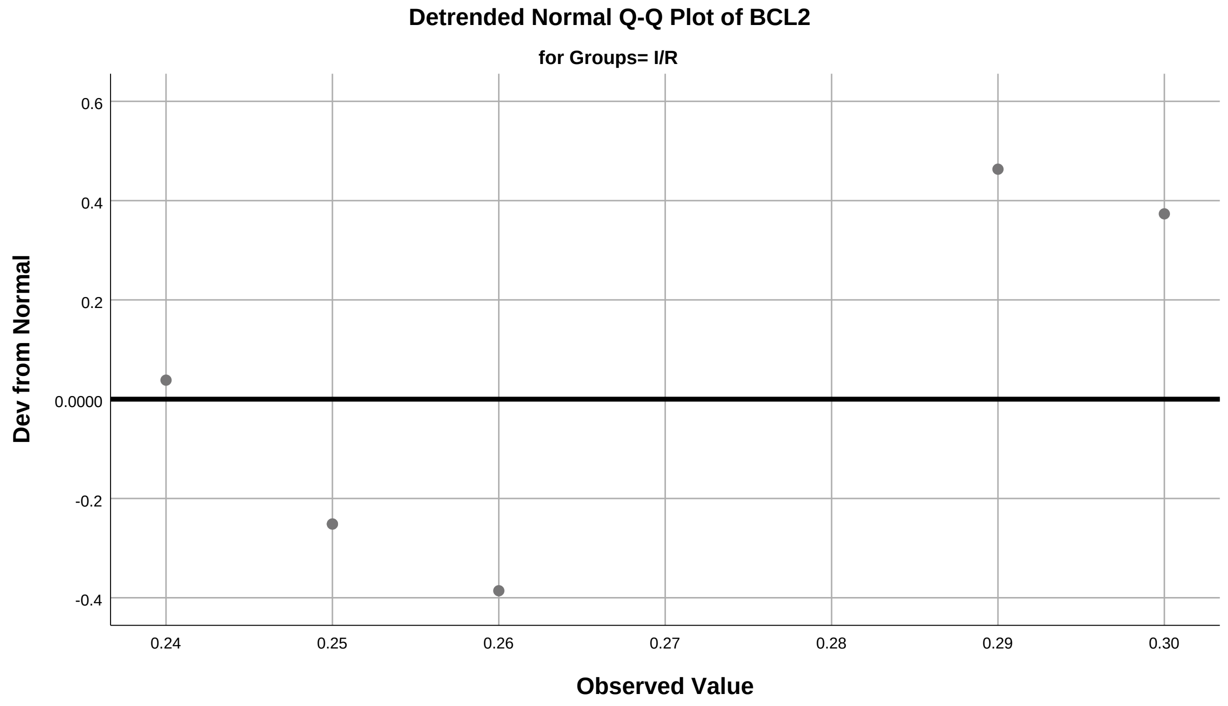
Detrended Normal Q-Q Plot of BCL2
for Groups= I/R
Dev from Normal
0.6
0.4
0.2
0.0000
-0.2
-0.4
0.24
0.25
0.26
0.27
0.28
0.29
0.30
Observed Value

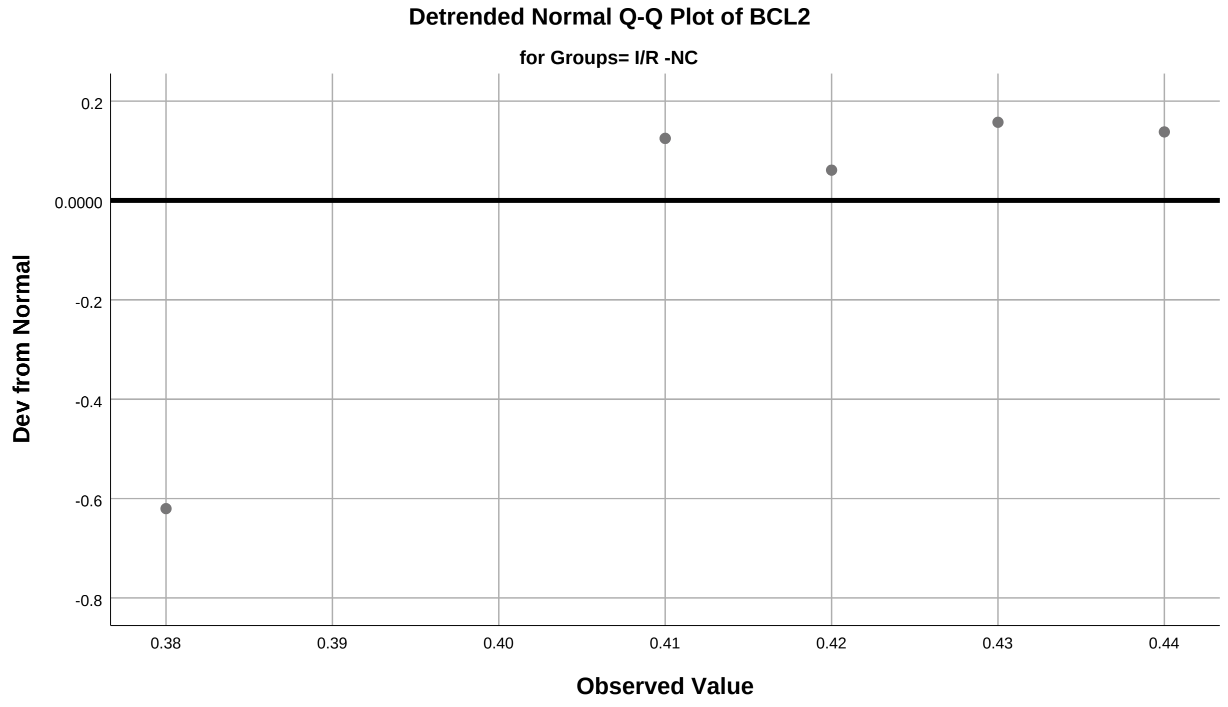
Detrended Normal Q-Q Plot of BCL2
for Groups= I/R -NC
Dev from Normal
0.2
0.0000
-0.2
-0.4
-0.6
-0.8
0.38
0.39
0.40
0.41
0.42
0.43
0.44
Observed Value

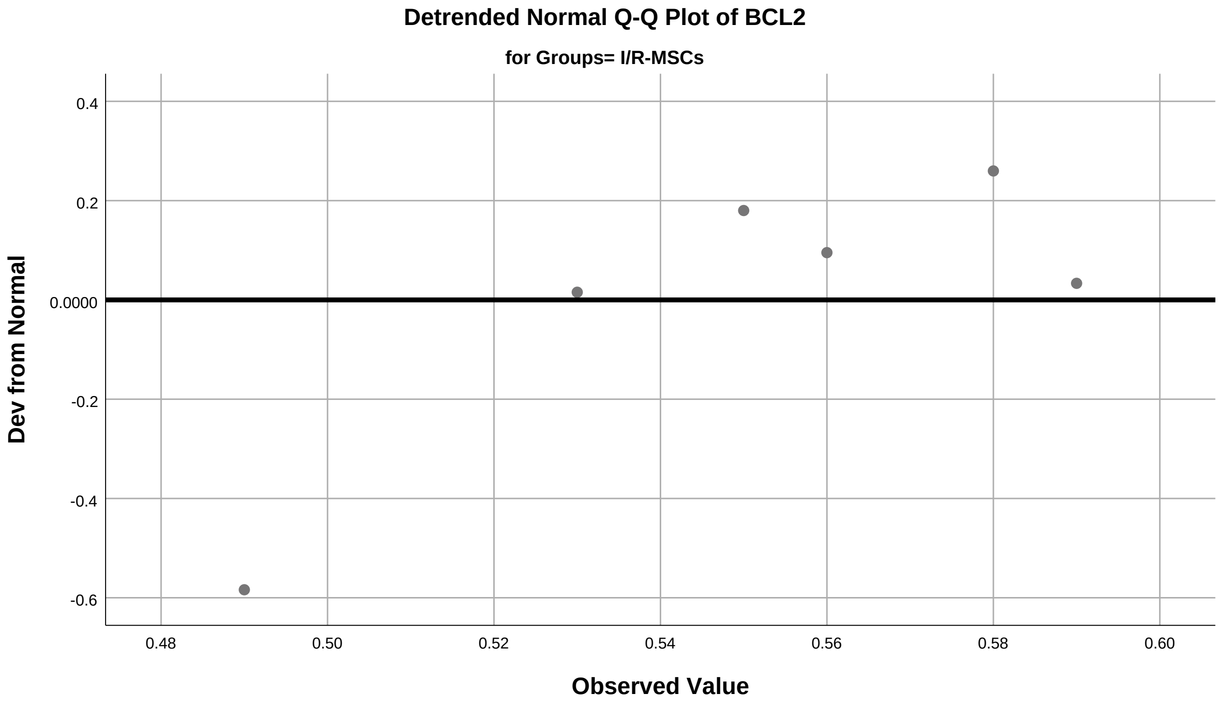
Detrended Normal Q-Q Plot of BCL2
for Groups= I/R-MSCs
Dev from Normal
0.4
0.2
0.0000
-0.2
-0.4
-0.6
0.48
0.50
0.52
0.54
0.56
0.58
0.60
Observed Value

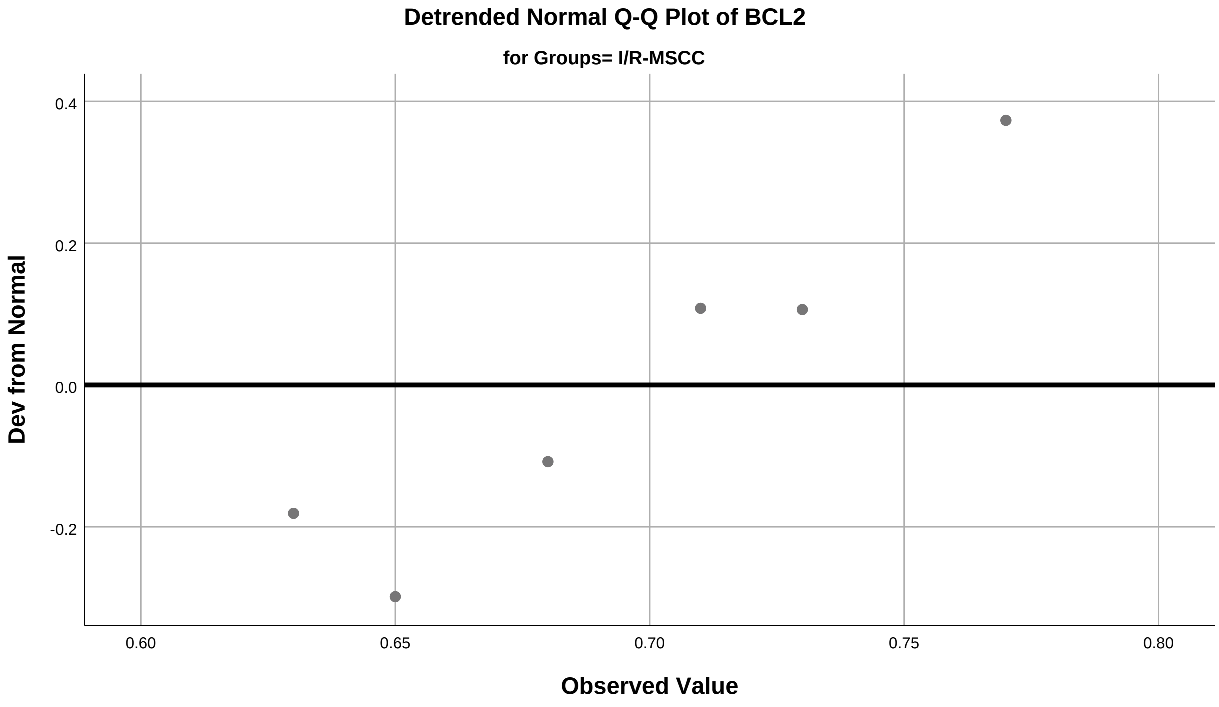
Detrended Normal Q-Q Plot of BCL2
for Groups= I/R-MSCC
Dev from Normal
0.4
0.2
0.0
-0.2
0.60
0.65
0.70
0.75
0.80
Observed Value

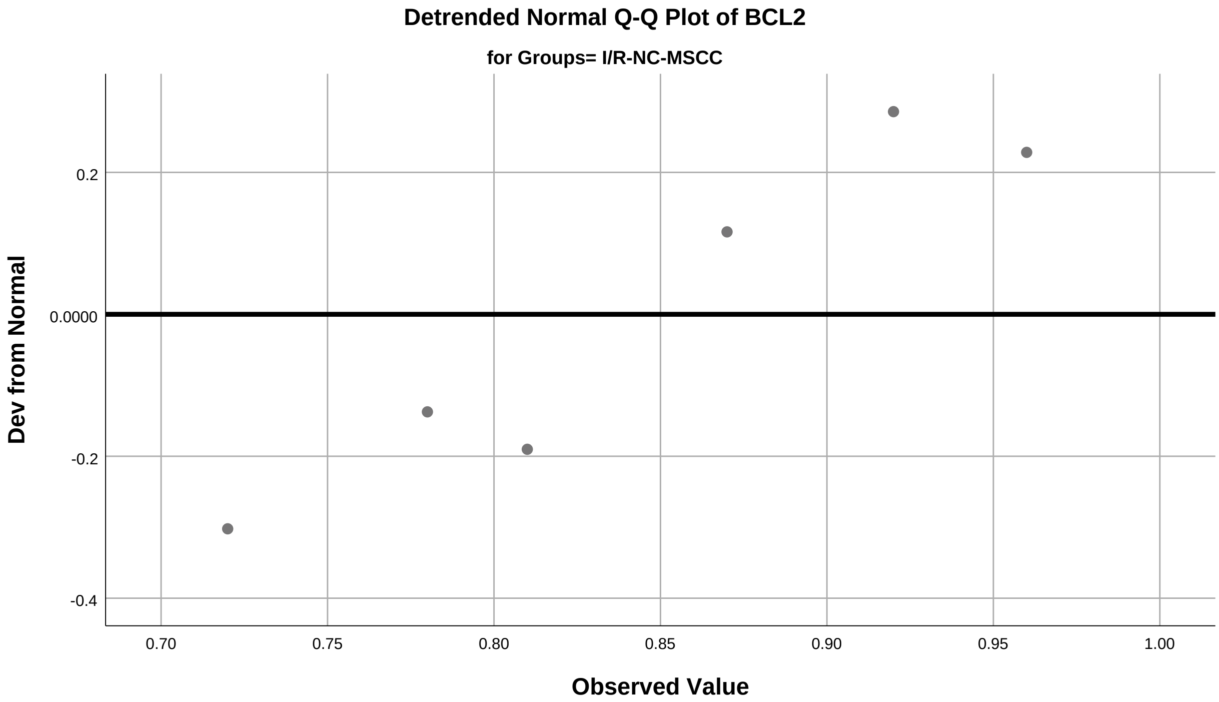

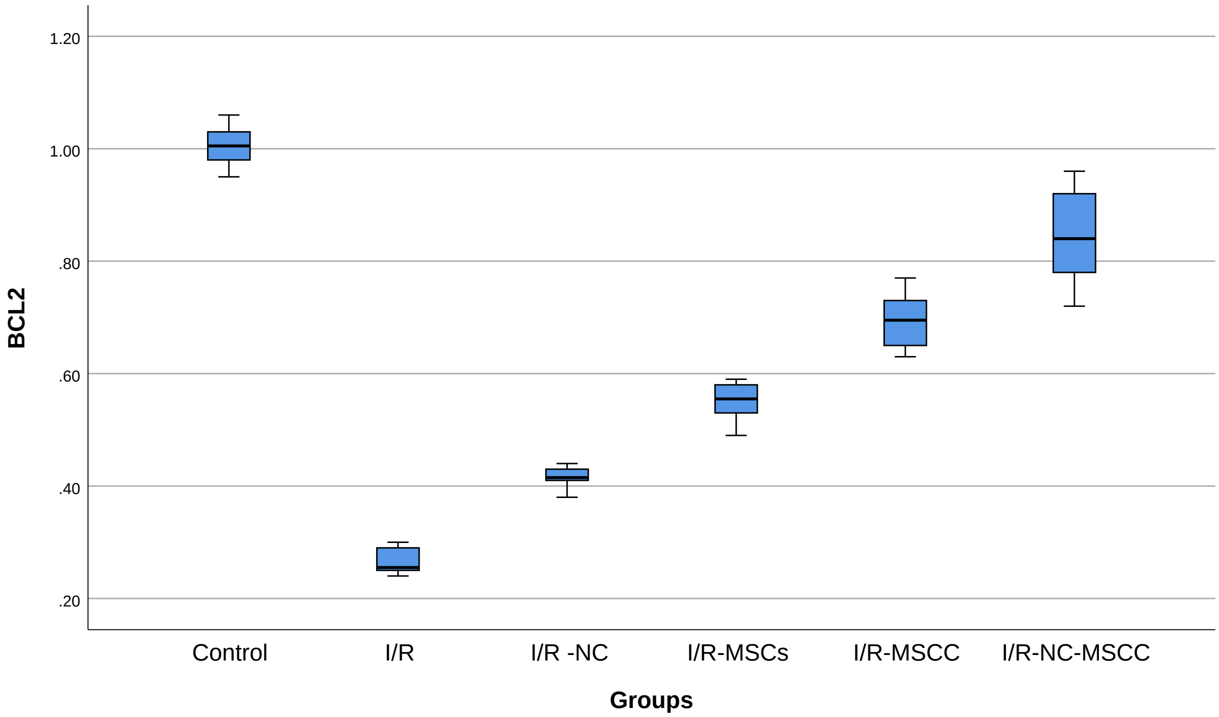

PI3K

Normal Q-Q Plots

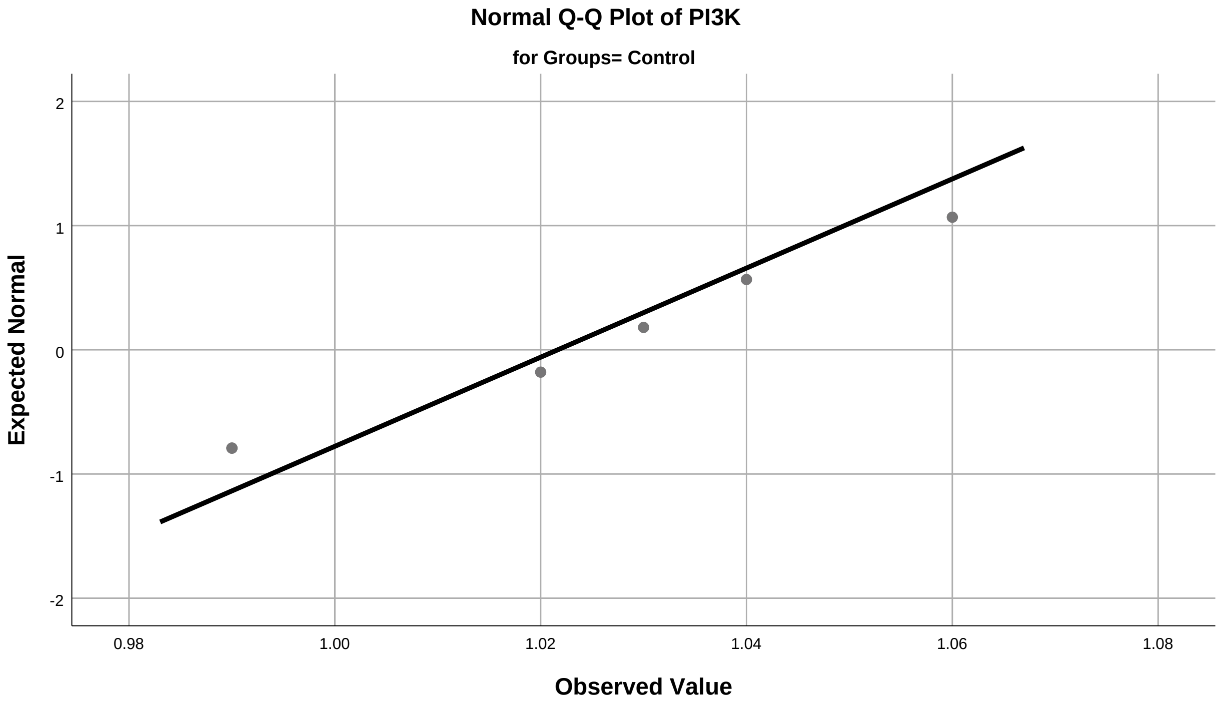
Normal Q-Q Plot of PI3K
for Groups= Control
Expected Normal
2
1
0
-1
-2
0.98
1.00
1.02
1.04
1.06
1.08
Observed Value

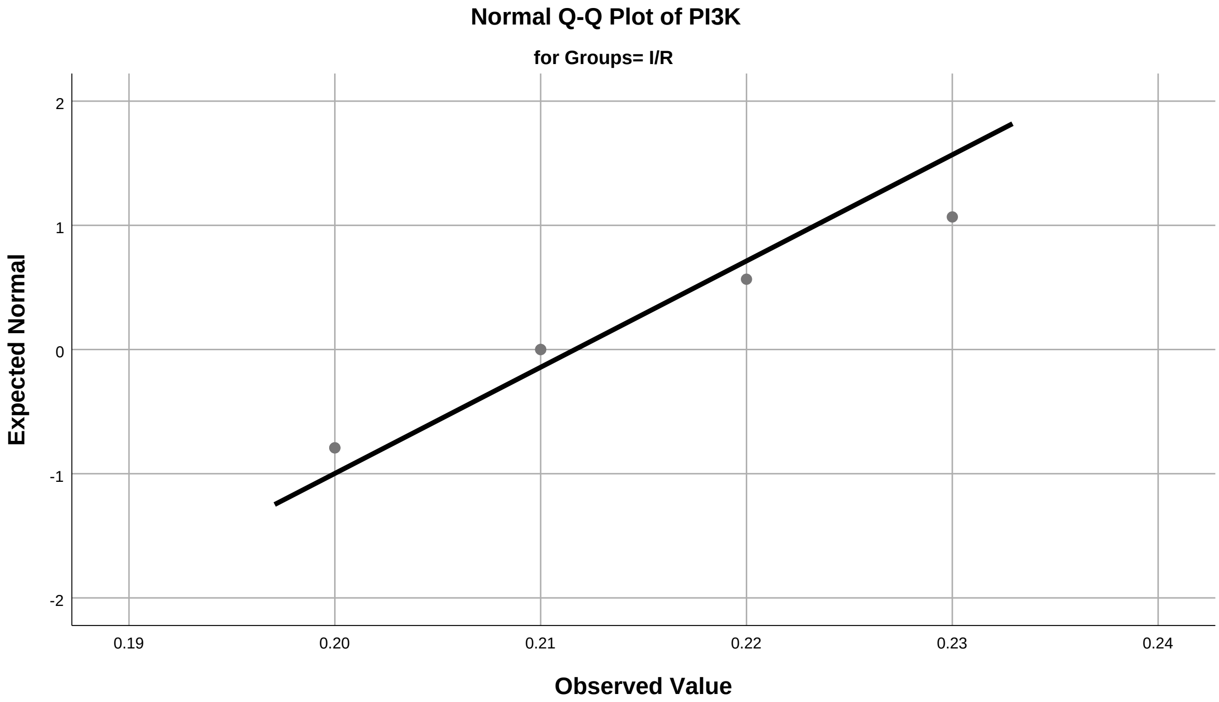
Normal Q-Q Plot of PI3K
for Groups= I/R
Expected Normal
2
1
0
-1
-2
0.19
0.20
0.21
0.22
0.23
0.24
Observed Value

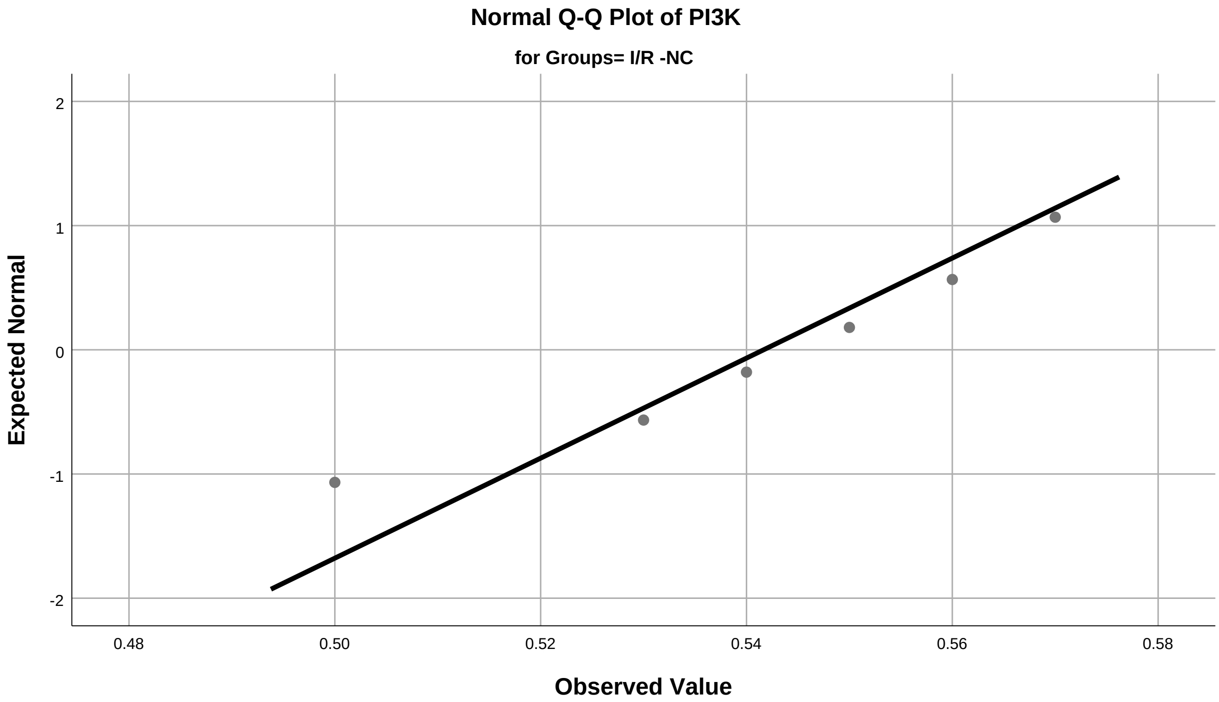

Normal Q-Q Plot of PI3K
for Groups= I/R -NC
Expected Normal
Observed Value
2
1
0
-1
-2
0.48
0.50
0.52
0.54
0.56
0.58

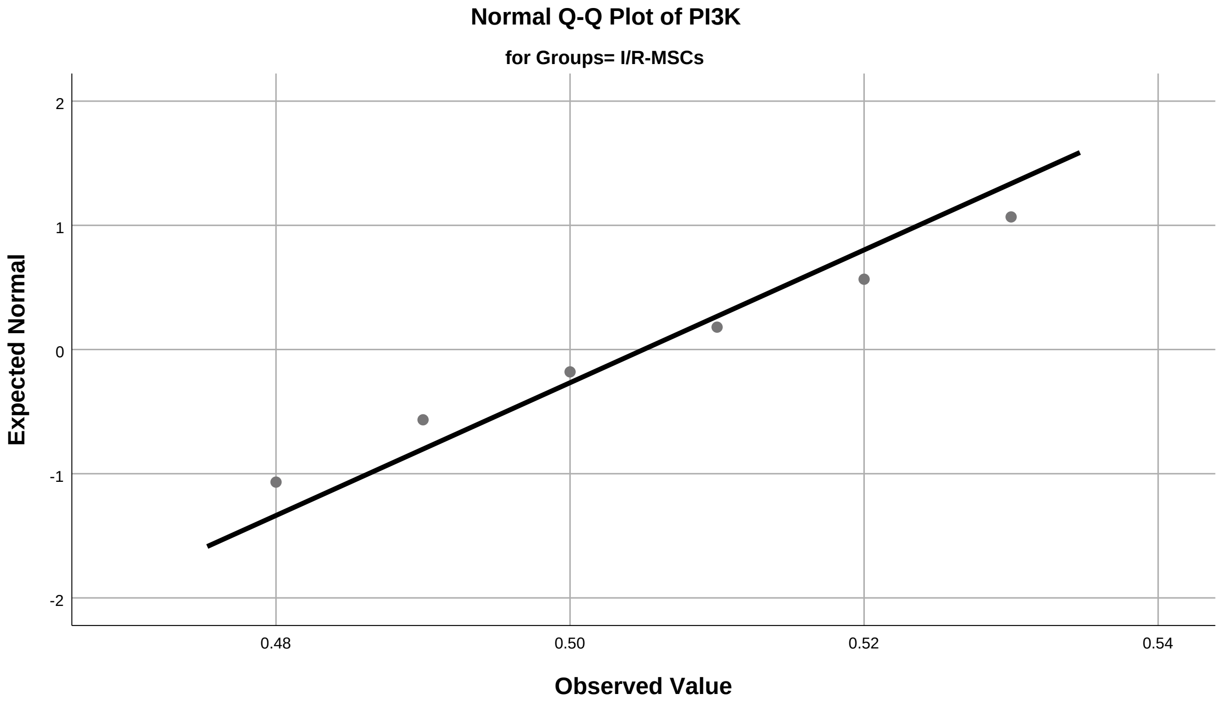

Normal Q-Q Plot of PI3K
for Groups= I/R-MSCs
Expected Normal
Observed Value
2
1
0
-1
-2
0.48
0.50
0.52
0.54

Normal Q-Q Plot of PI3K

for Groups= I/R-MSCC

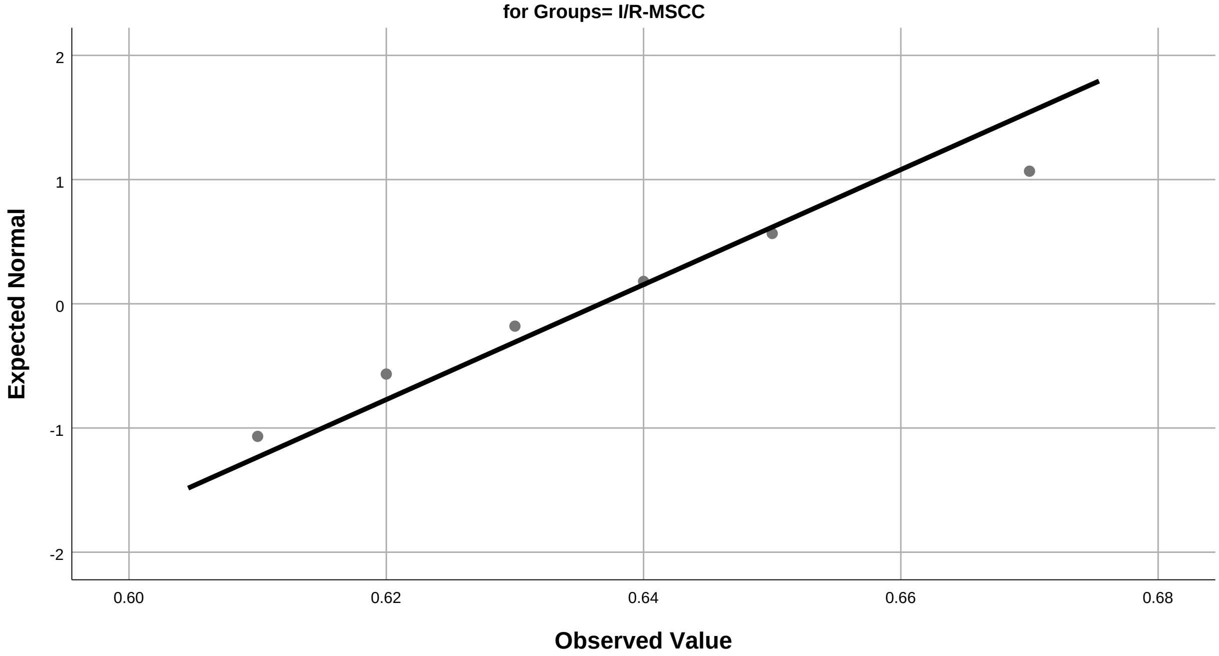

Normal Q-Q Plot of PI3K

for Groups= I/R-NC-MSCC

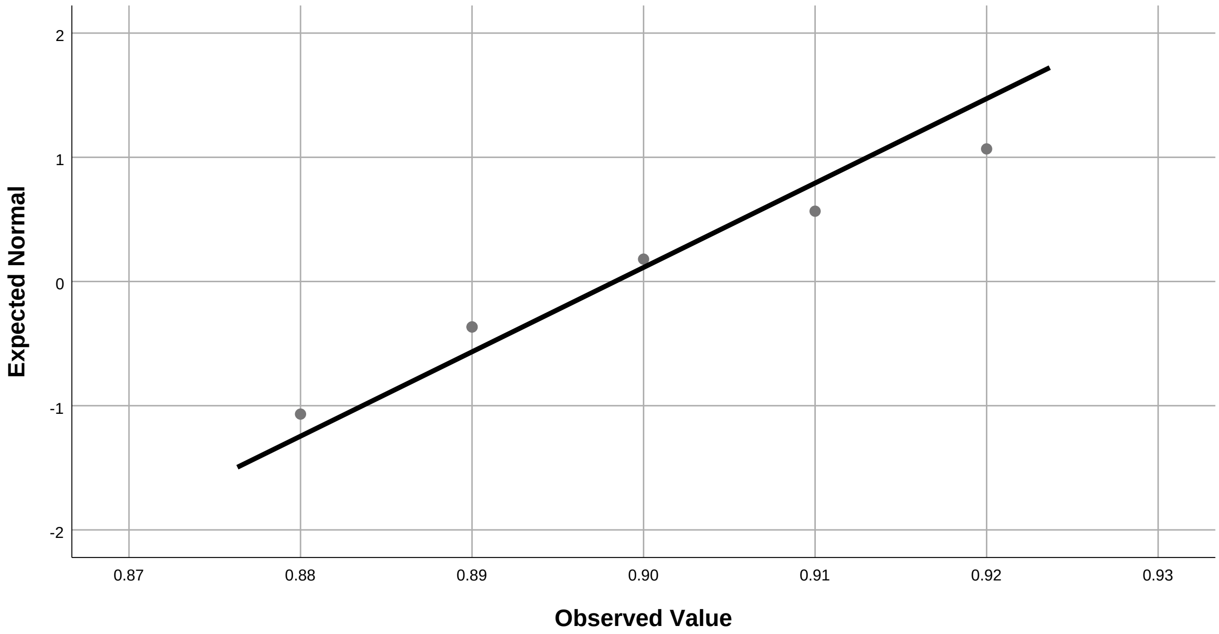

Detrended Normal Q-Q Plots

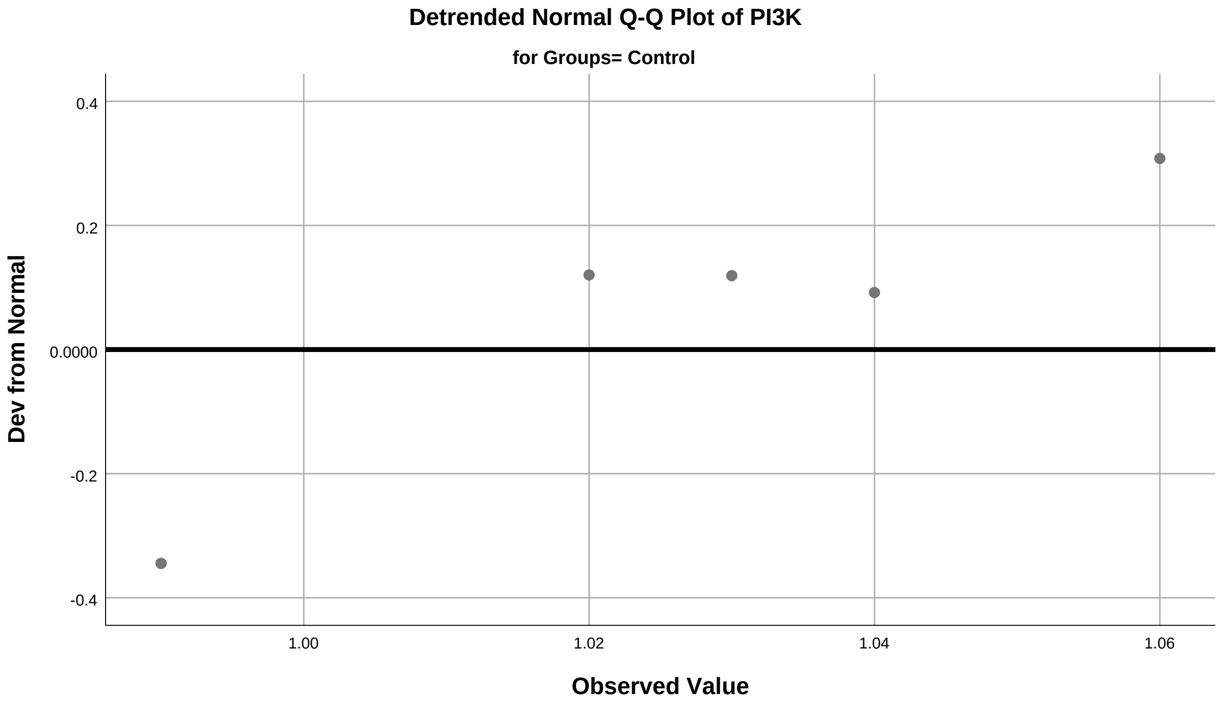
Detrended Normal Q-Q Plot of PI3K
for Groups= Control
Dev from Normal
0.4
0.2
0.0000
-0.2
-0.4
1.00
1.02
1.04
1.06
Observed Value

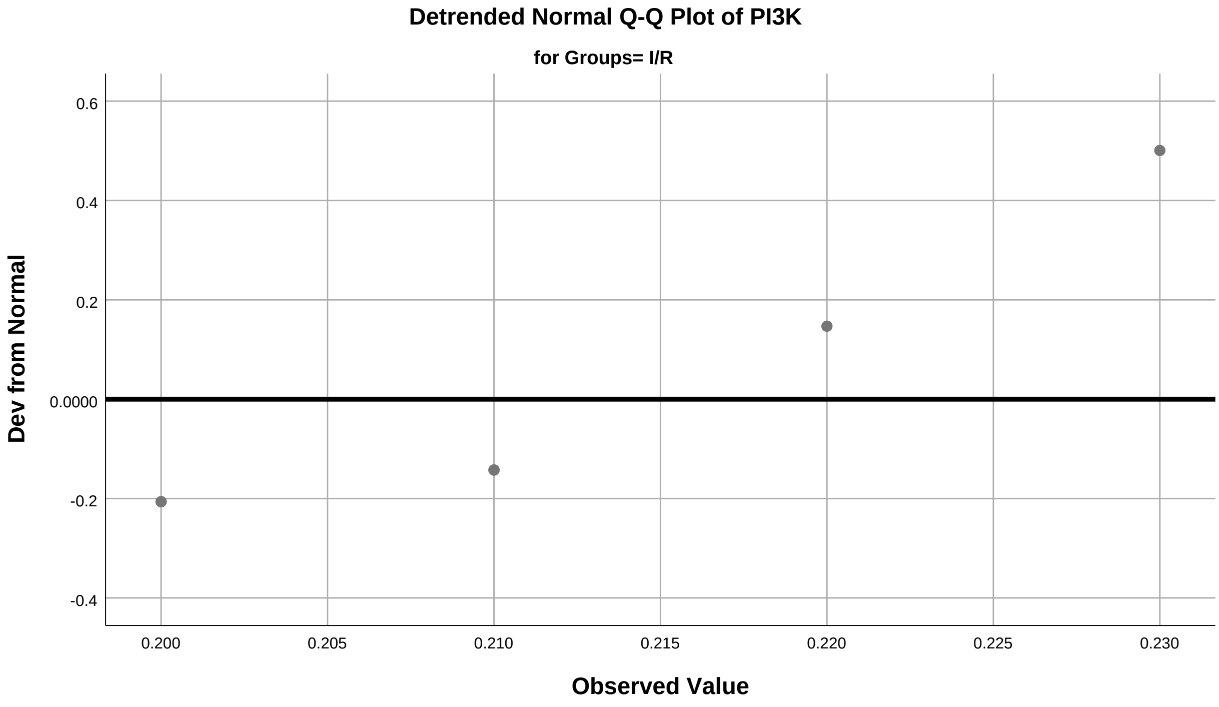
Detrended Normal Q-Q Plot of PI3K
for Groups= I/R
Dev from Normal
0.6
0.4
0.2
0.0000
-0.2
-0.4
0.200
0.205
0.210
0.215
0.220
0.225
0.230
Observed Value

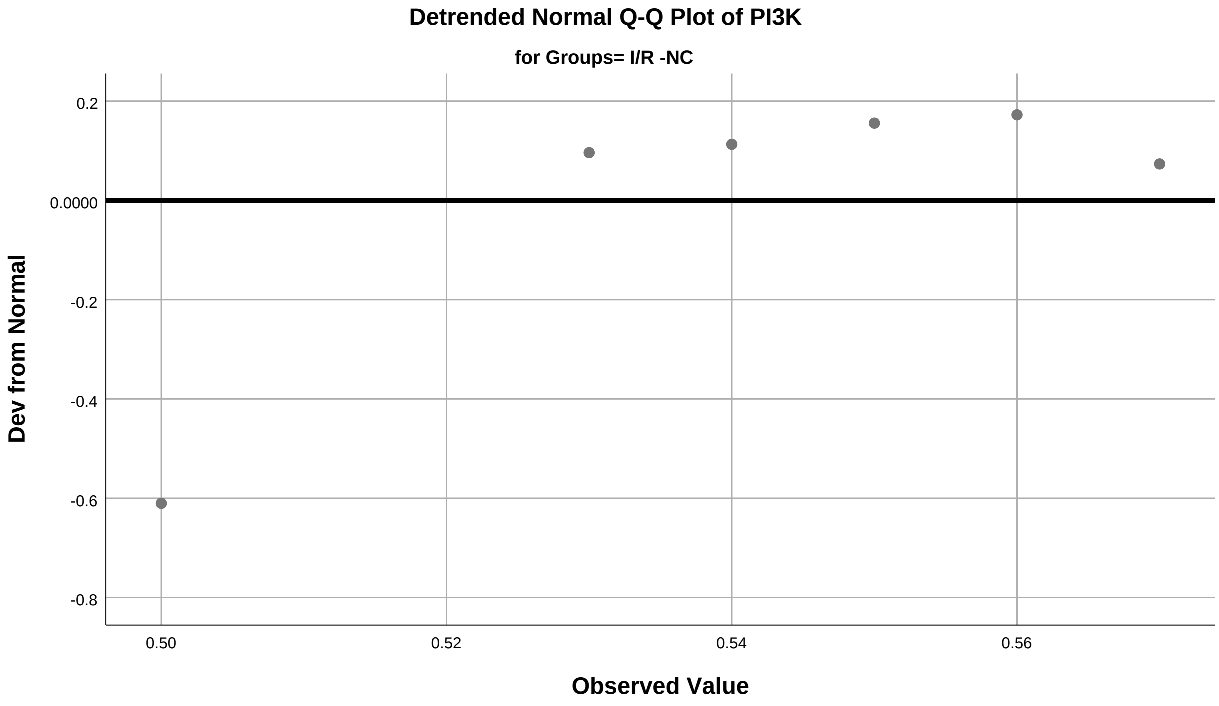
Detrended Normal Q-Q Plot of PI3K
for Groups= I/R -NC
Dev from Normal
0.2
0.0000
-0.2
-0.4
-0.6
-0.8
0.50
0.52
0.54
0.56
Observed Value

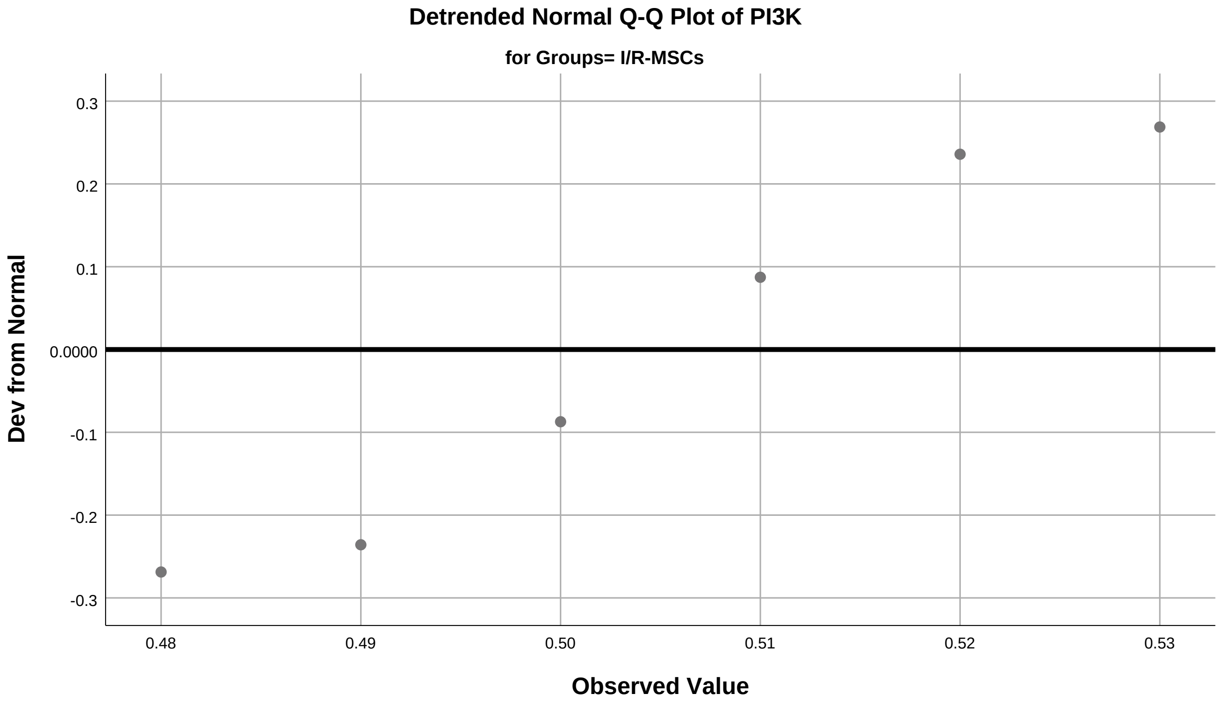
Detrended Normal Q-Q Plot of PI3K
for Groups= I/R-MSCs
Dev from Normal
0.3
0.2
0.1
0.0000
-0.1
-0.2
-0.3
0.48
0.49
0.50
0.51
0.52
0.53
Observed Value

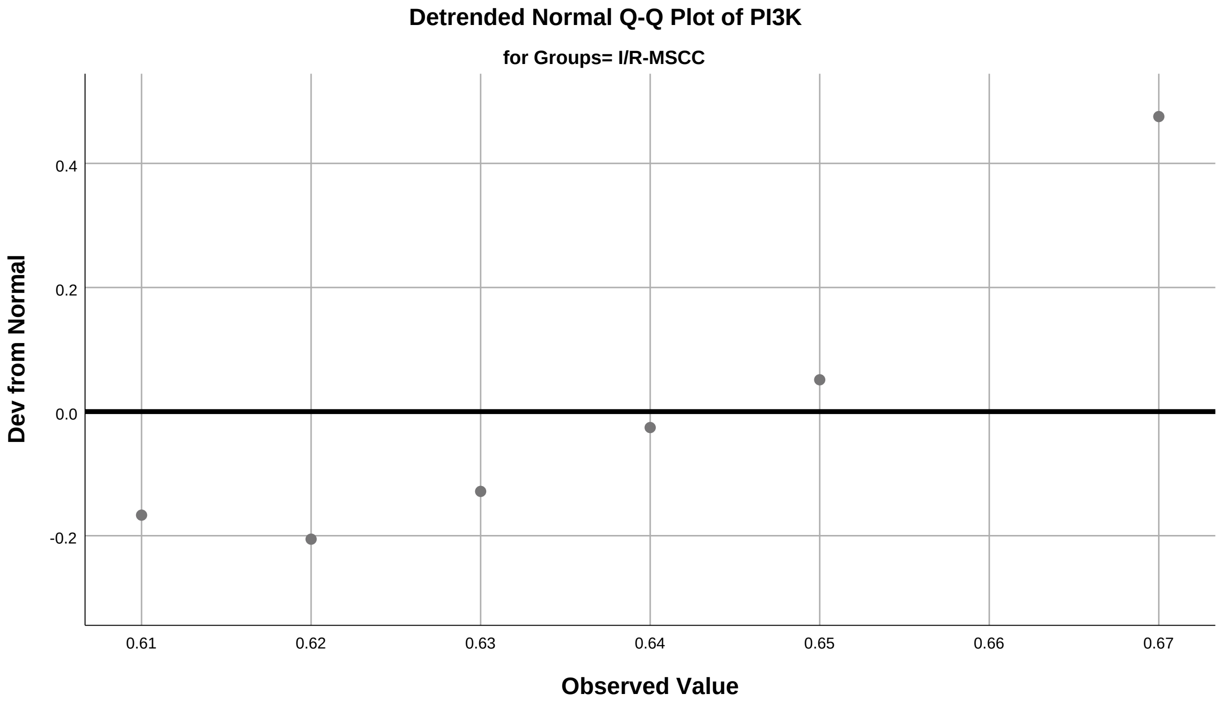

Detrended Normal Q-Q Plot of PI3K
for Groups= I/R-MSCC
Dev from Normal
0.4
0.2
0.0
-0.2
0.61
0.62
0.63
0.64
0.65
0.66
0.67
Observed Value

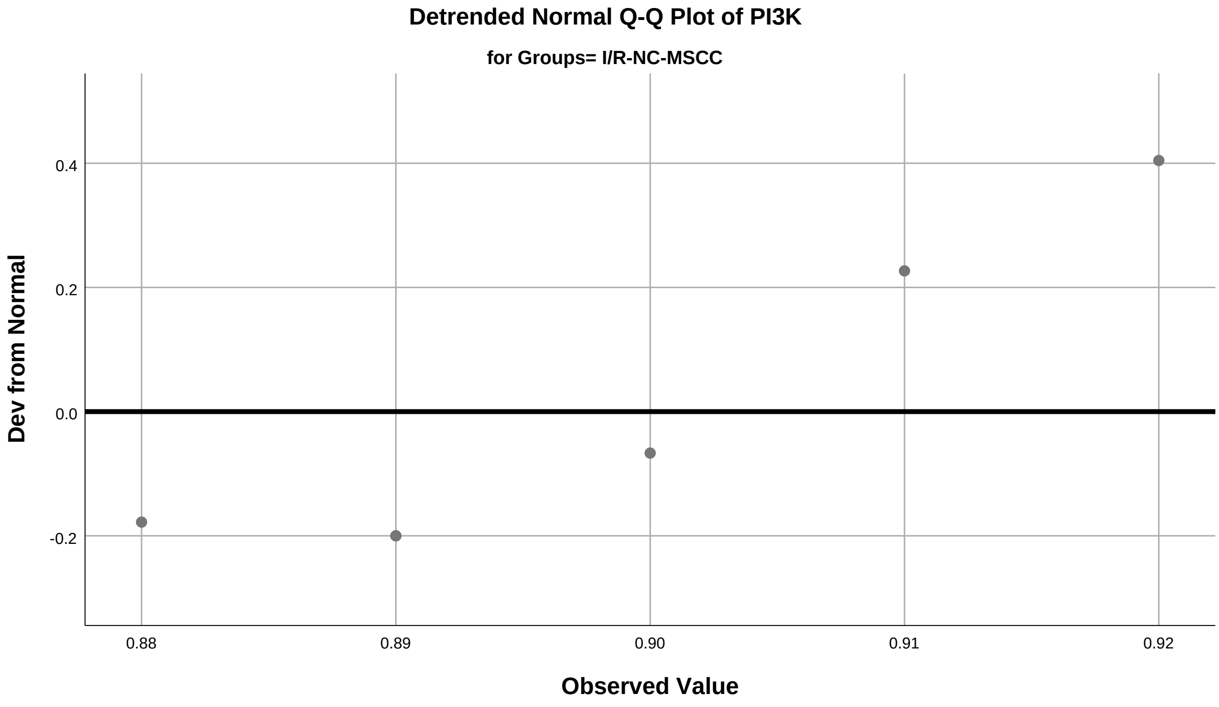

Detrended Normal Q-Q Plot of PI3K
for Groups= I/R-NC-MSCC
Dev from Normal
0.4
0.2
0.0
-0.2
0.88
0.89
0.90
0.91
0.92
Observed Value

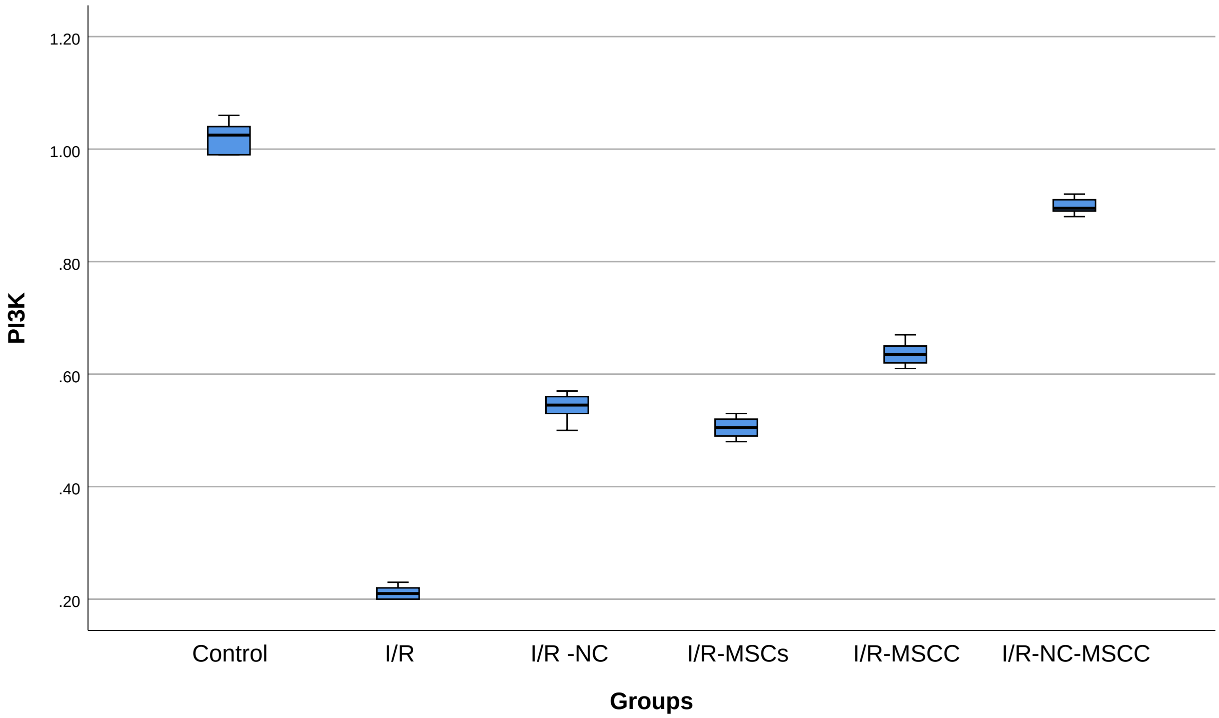

AKT

Normal Q-Q Plots

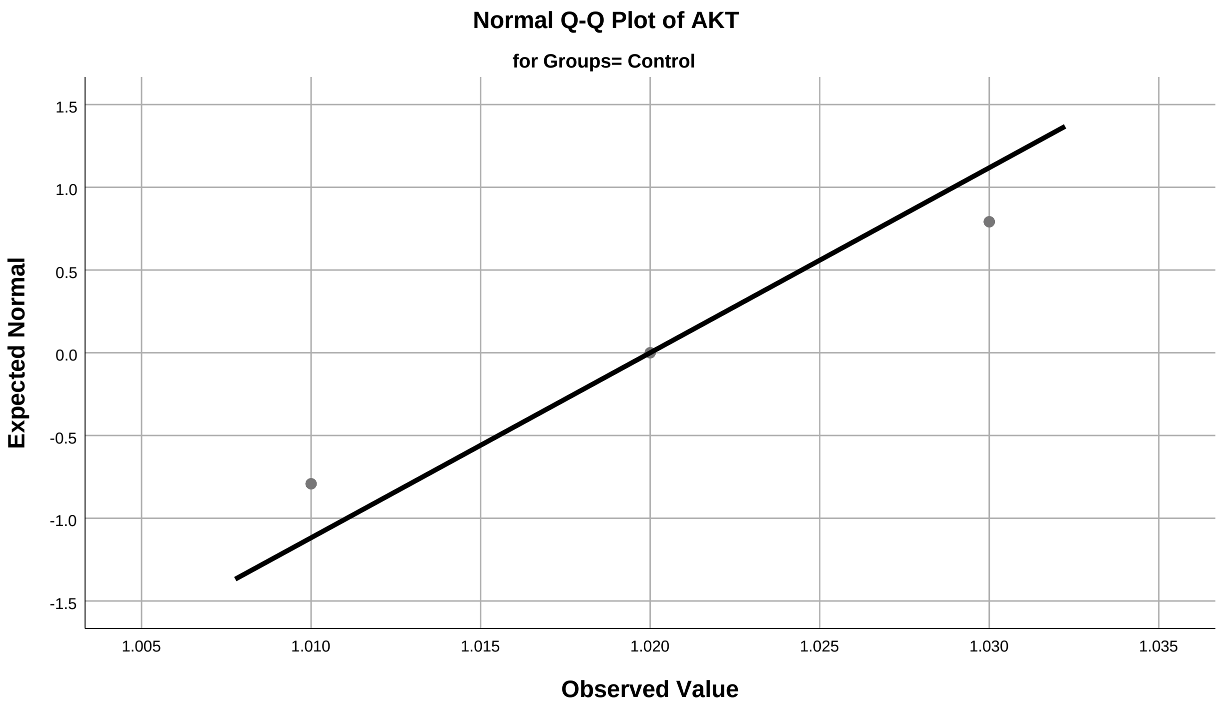

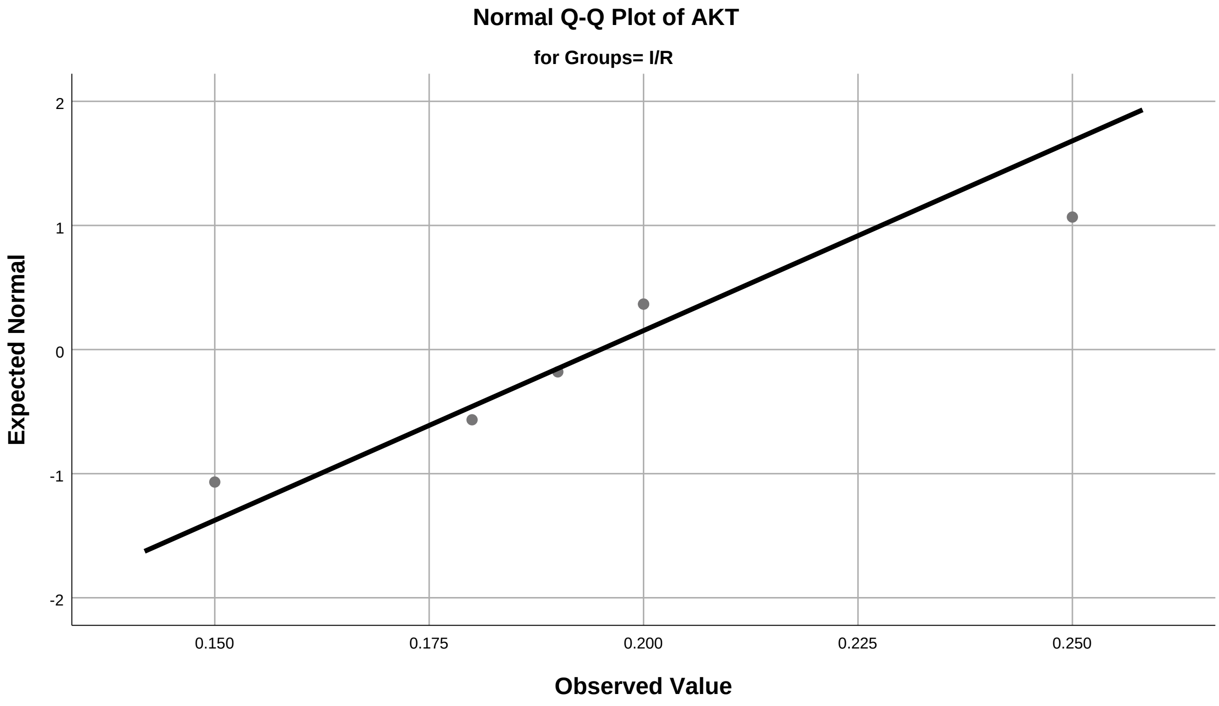
Normal Q-Q Plot of AKT
for Groups= I/R
Expected Normal
2
1
0
-1
-2
0.150
0.175
0.200
0.225
0.250
Observed Value

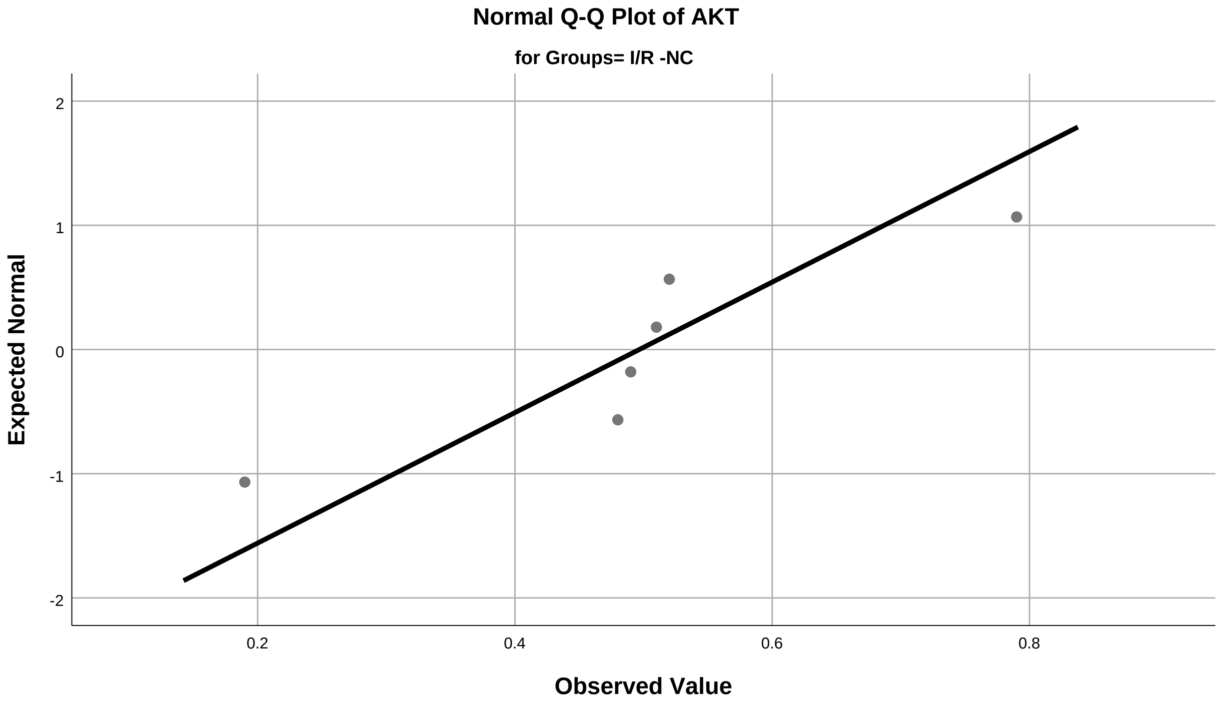
Normal Q-Q Plot of AKT
for Groups= I/R -NC
Expected Normal
2
1
0
-1
-2
0.2
0.4
0.6
0.8
Observed Value

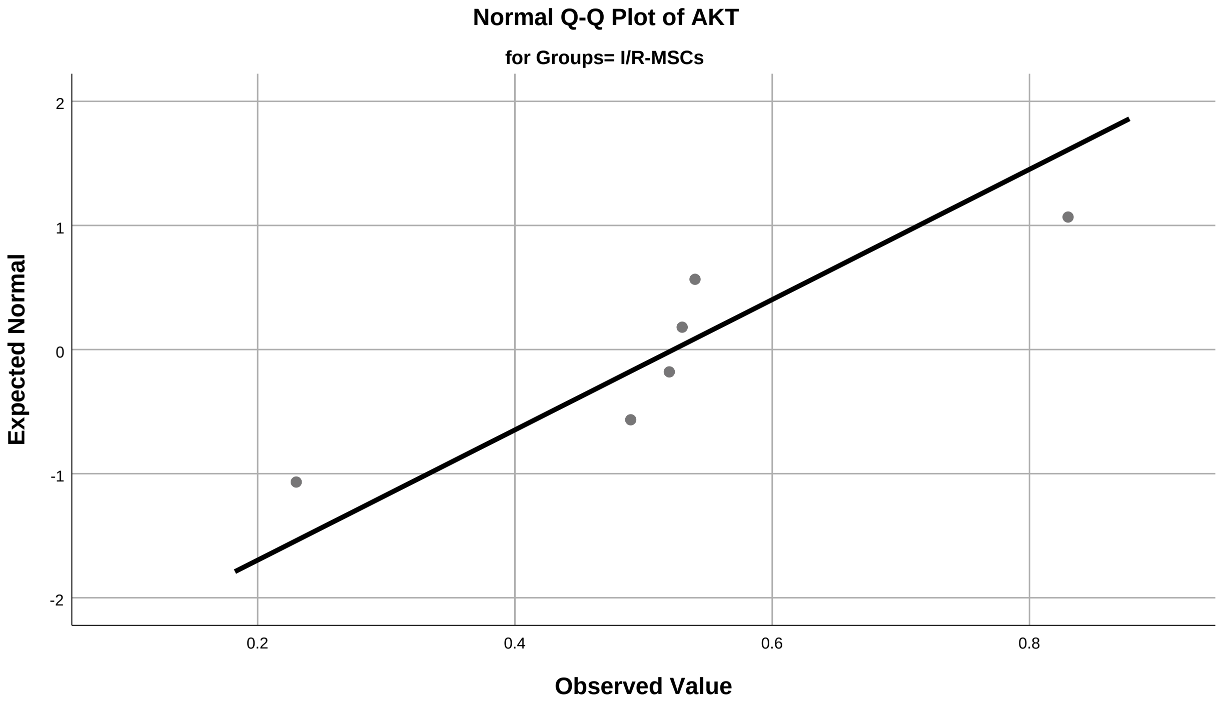
Normal Q-Q Plot of AKT
for Groups= I/R-MSCs
Expected Normal
2
1
0
-1
-2
0.2
0.4
0.6
0.8
Observed Value

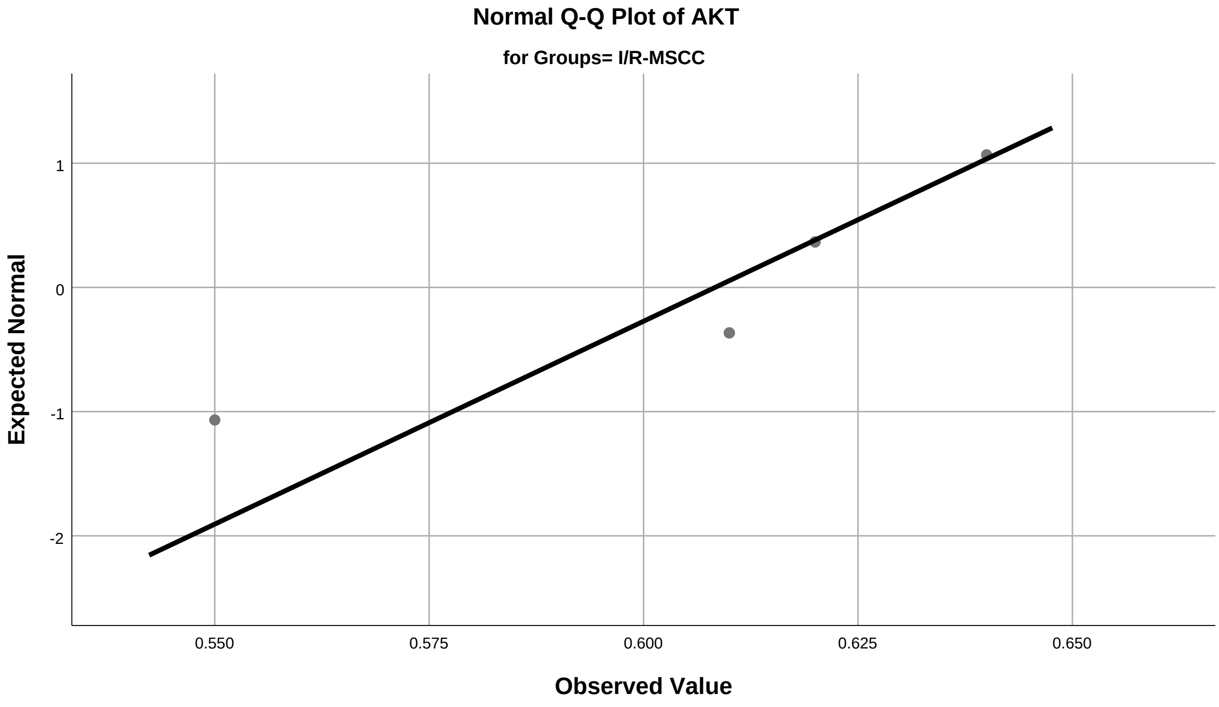
Normal Q-Q Plot of AKT
for Groups= I/R-MSCC
Expected Normal
1
0
-1
-2
0.550
0.575
0.600
0.625
0.650
Observed Value

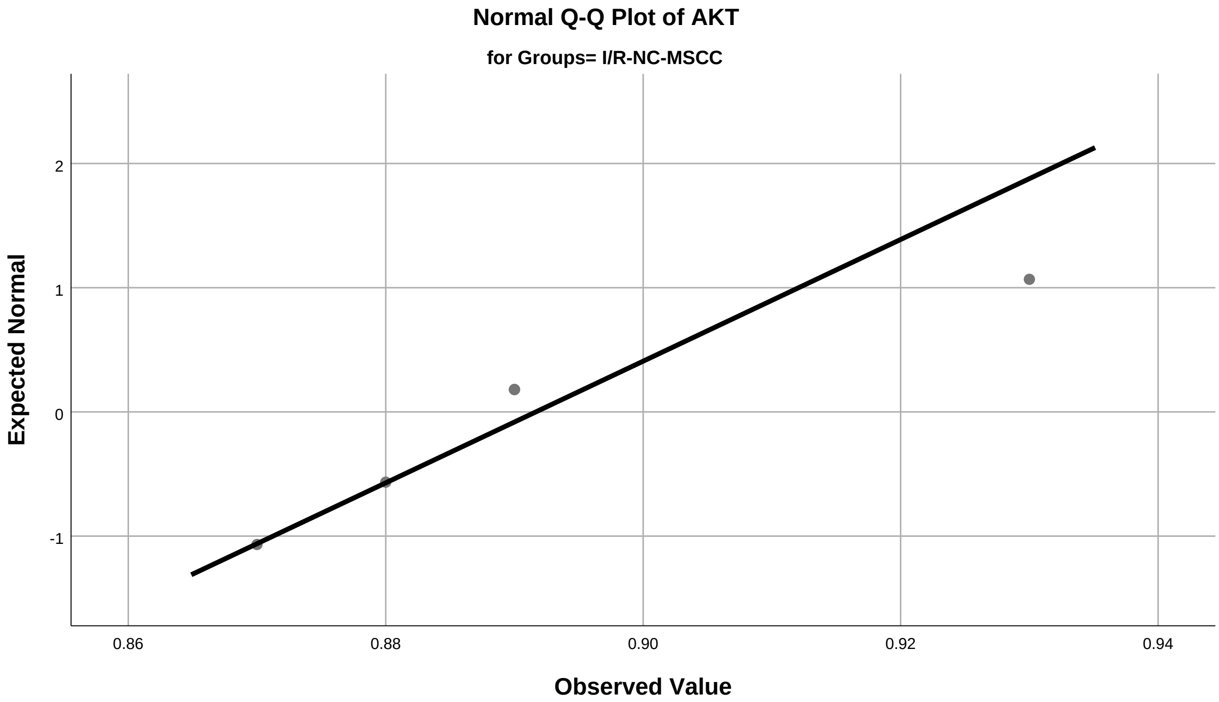

Detrended Normal Q-Q Plots

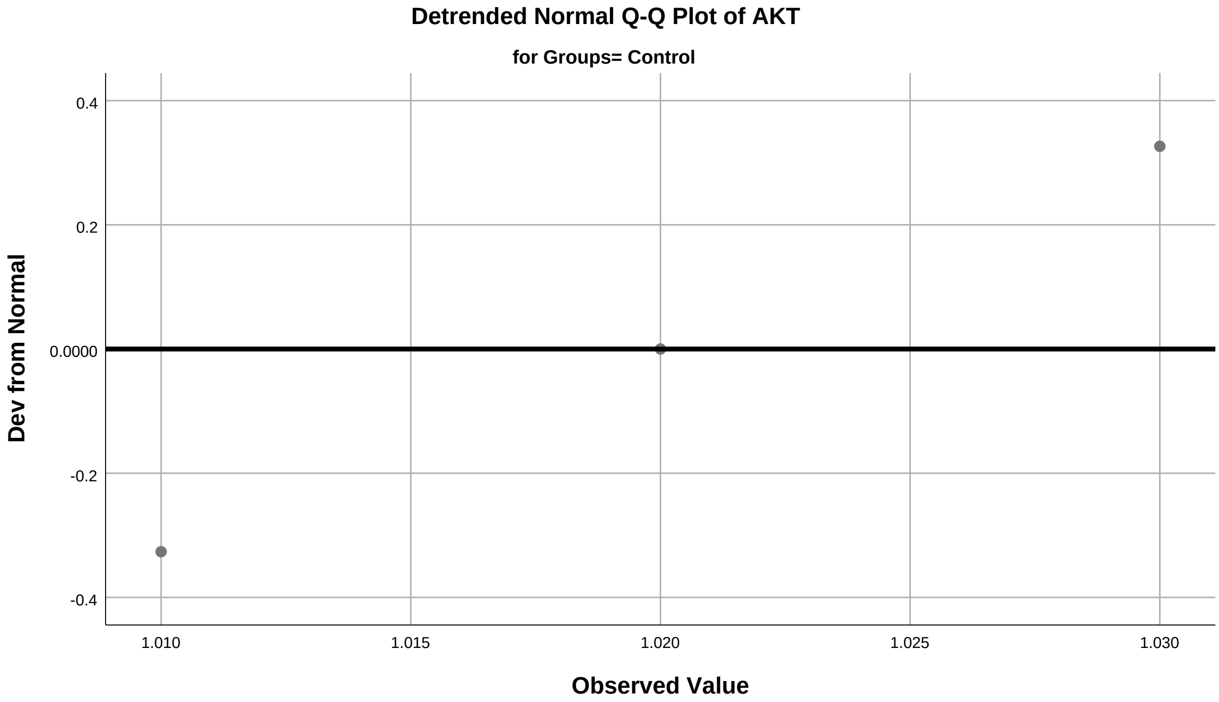

Detrended Normal Q-Q Plot of AKT

for Groups= I/R

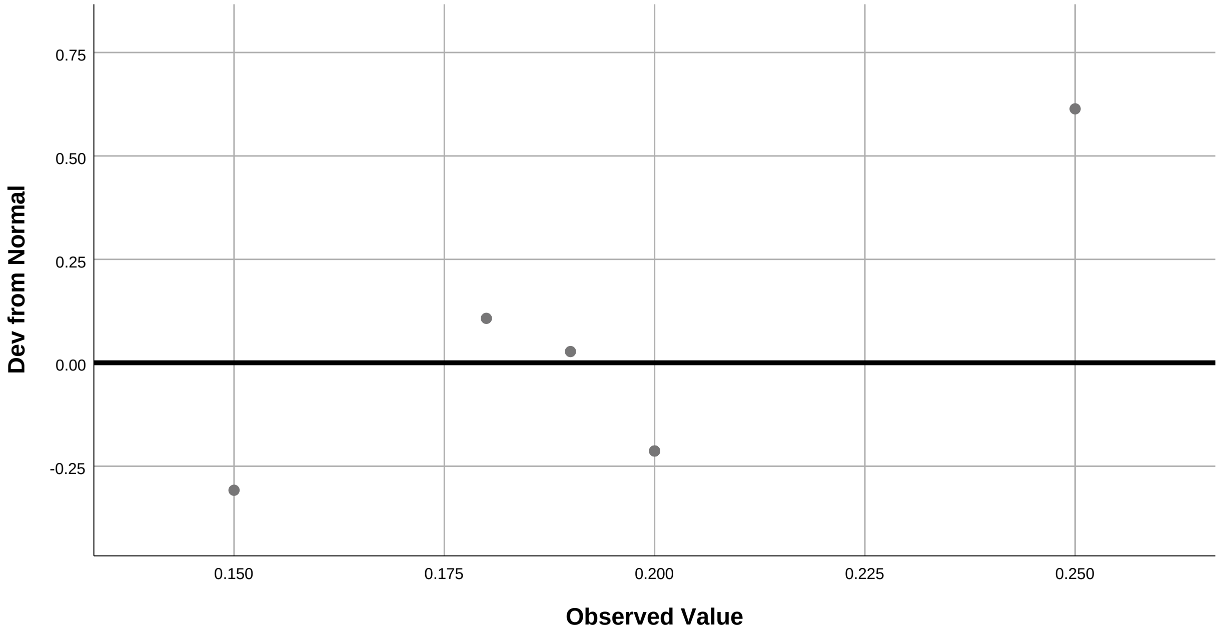

Detrended Normal Q-Q Plot of AKT

for Groups= I/R -NC

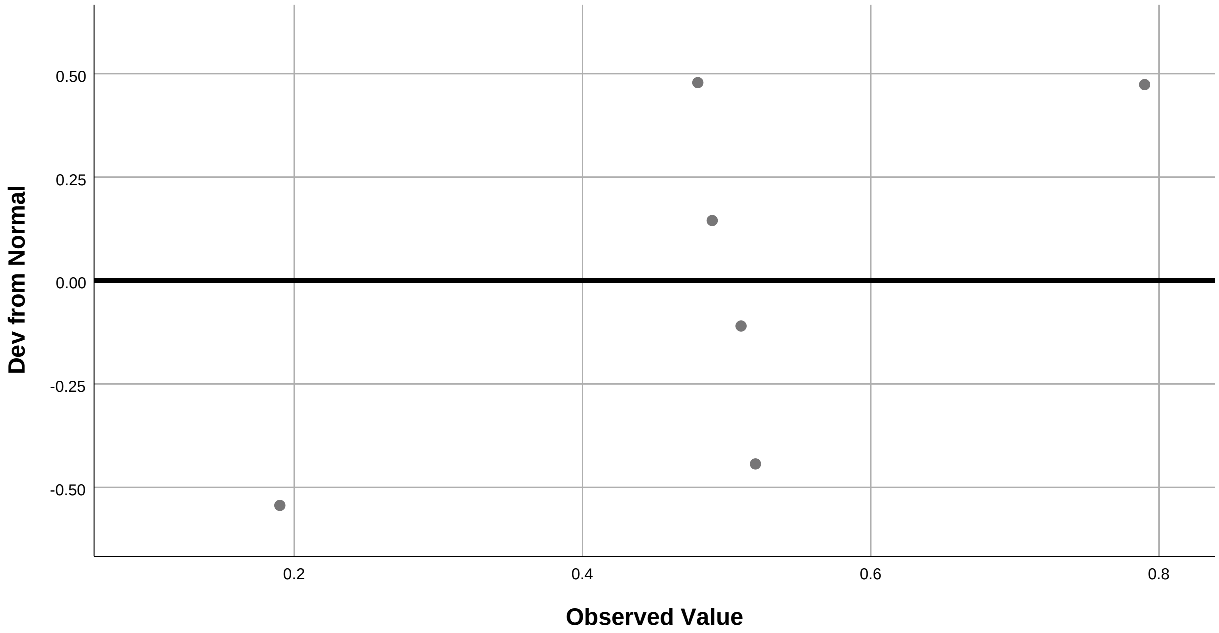

Detrended Normal Q-Q Plot of AKT

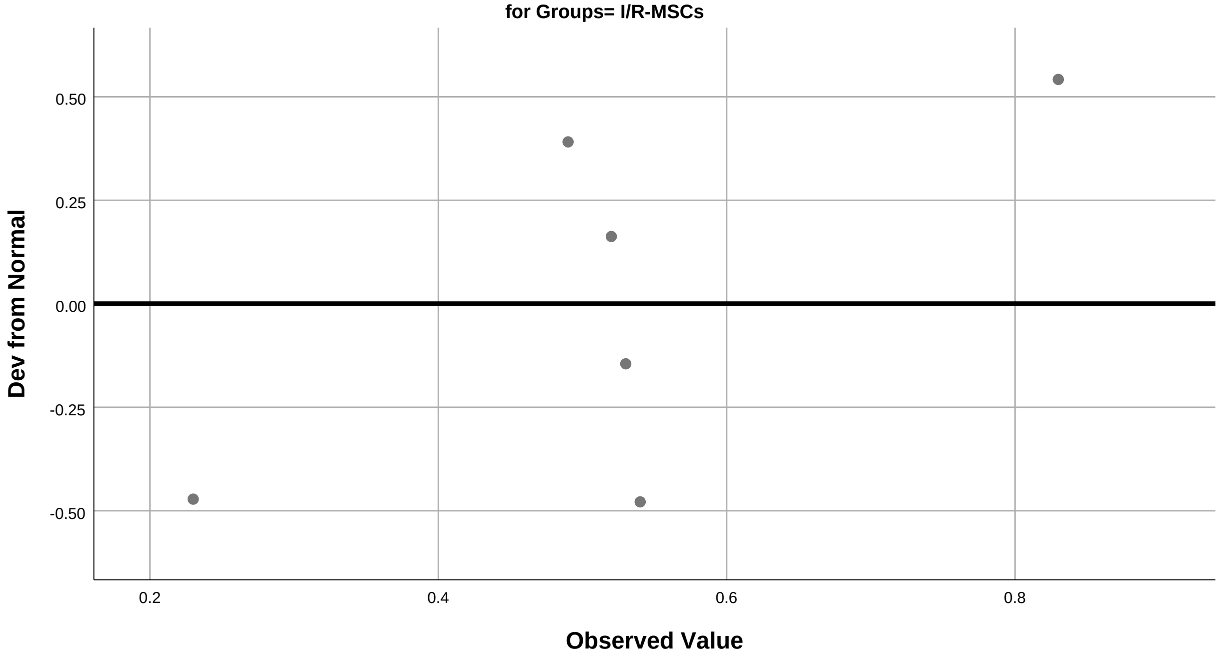

Detrended Normal Q-Q Plot of AKT

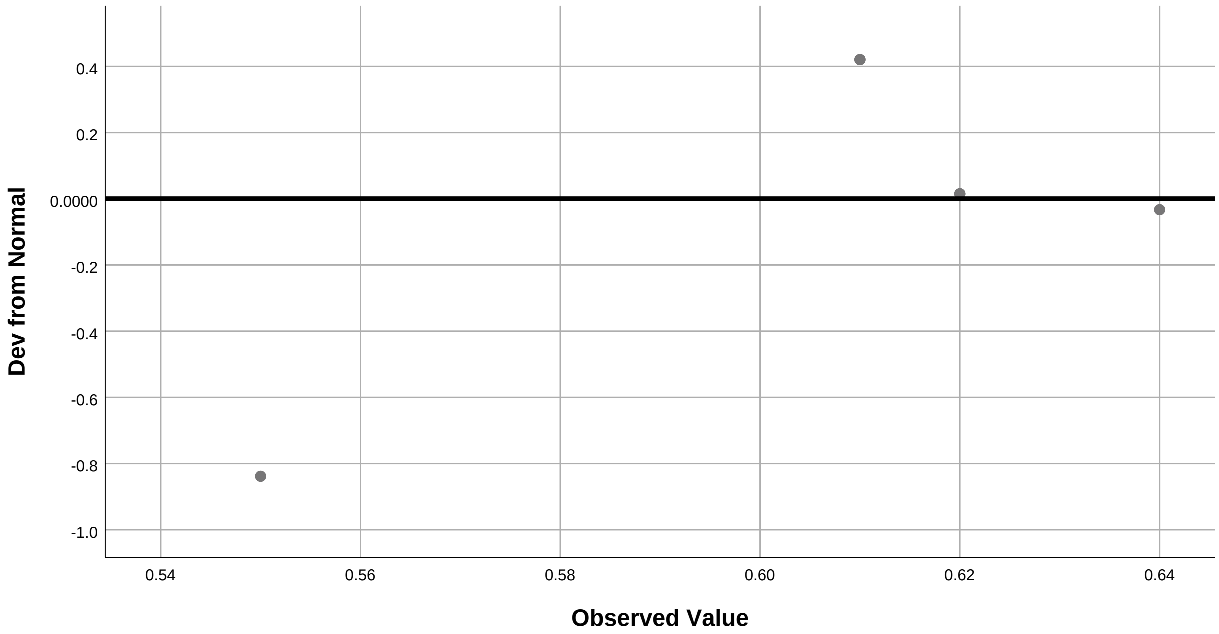

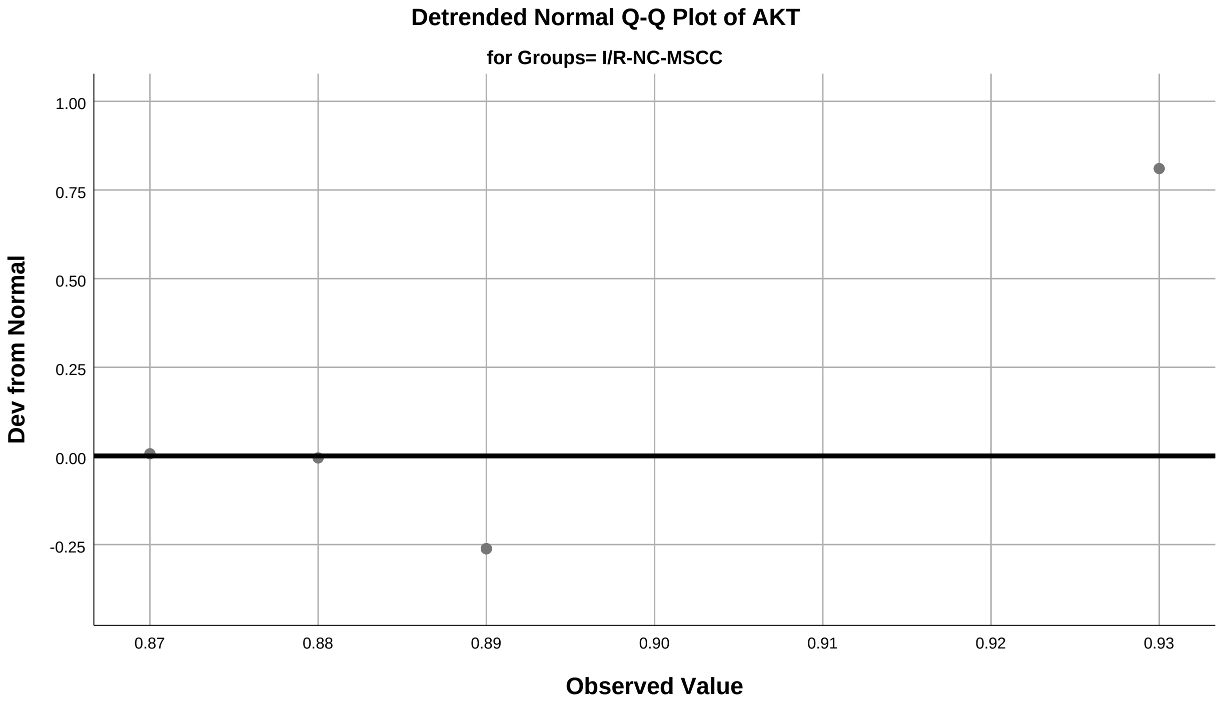

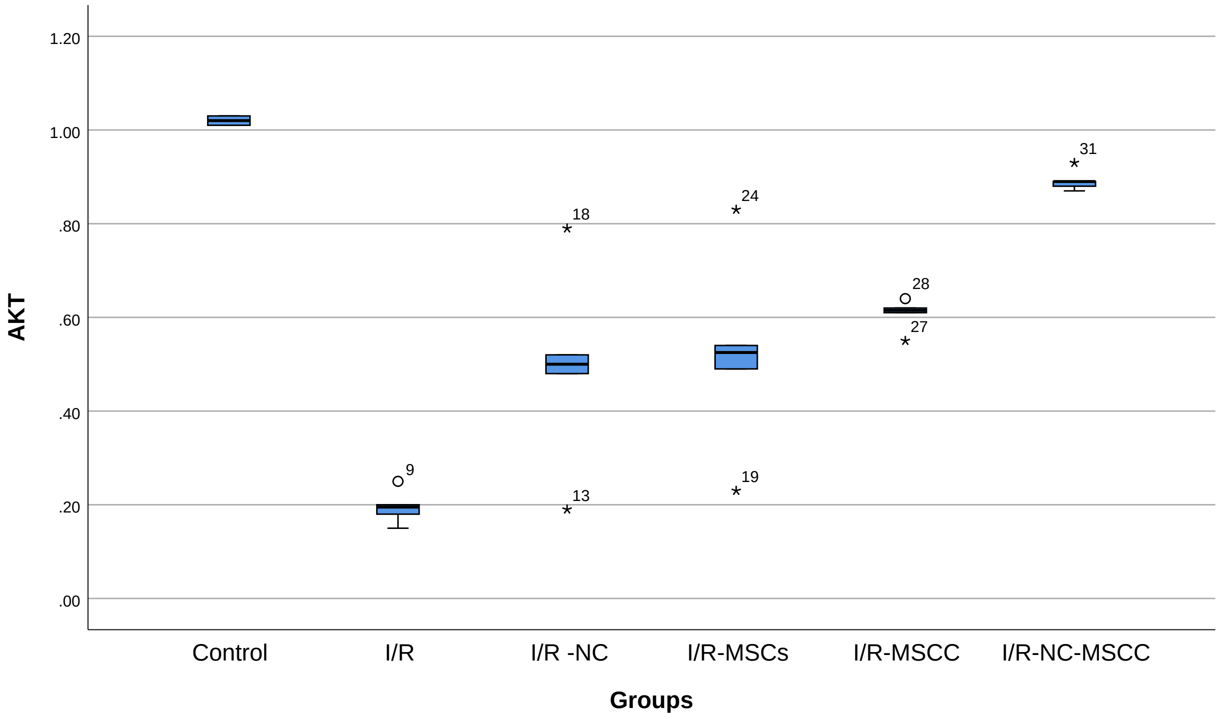

mTOR

Normal Q-Q Plots

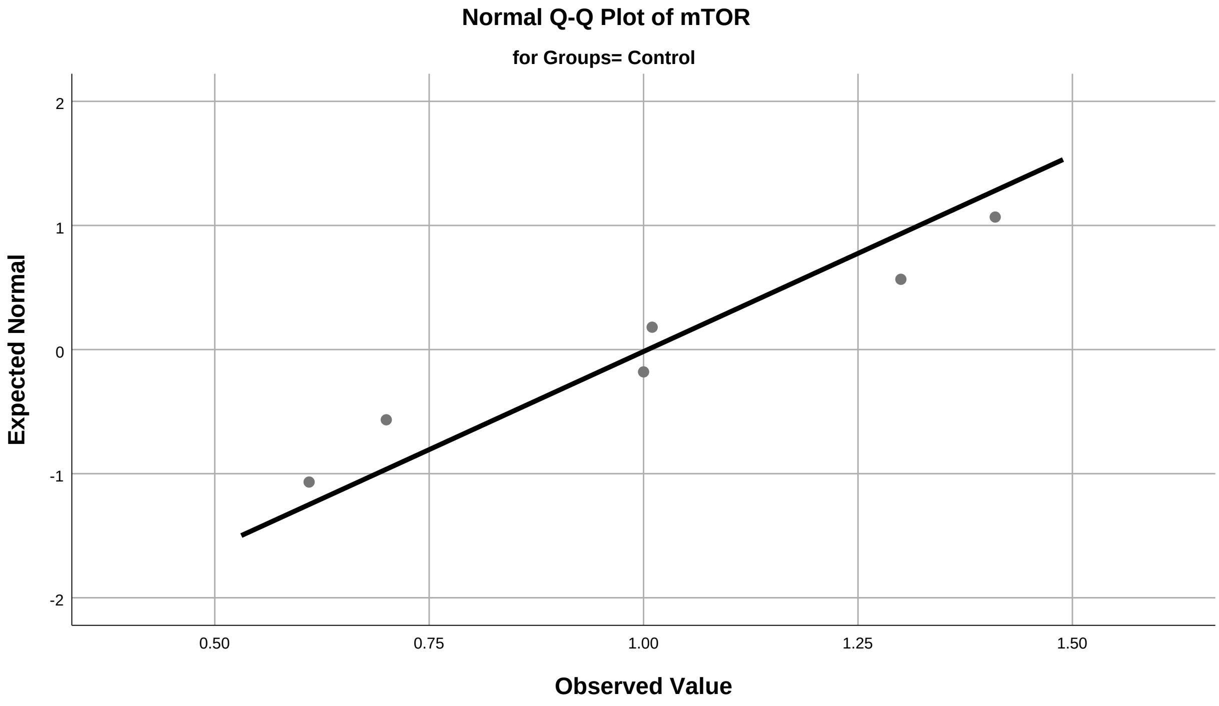
Normal Q-Q Plot of mTOR
for Groups= Control
Expected Normal
Observed Value
2
1
0
-1
-2
0.50
0.75
1.00
1.25
1.50

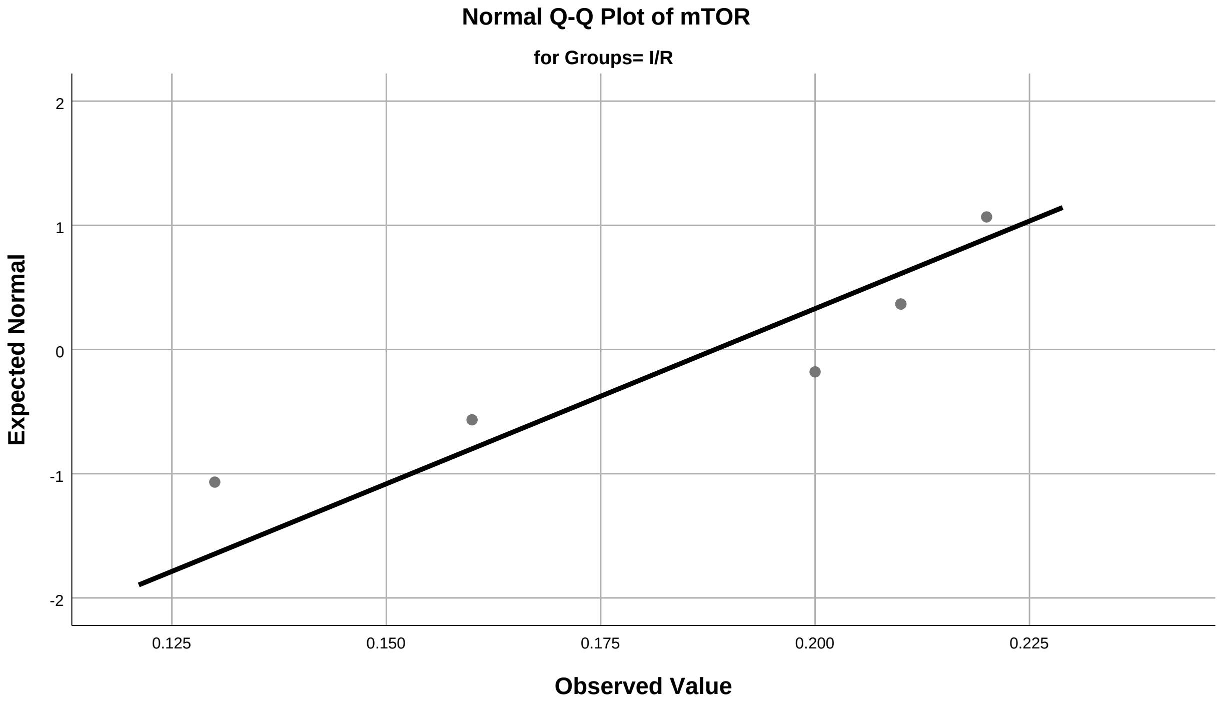
Normal Q-Q Plot of mTOR
for Groups= I/R
Expected Normal
Observed Value
2
1
0
-1
-2
0.125
0.150
0.175
0.200
0.225

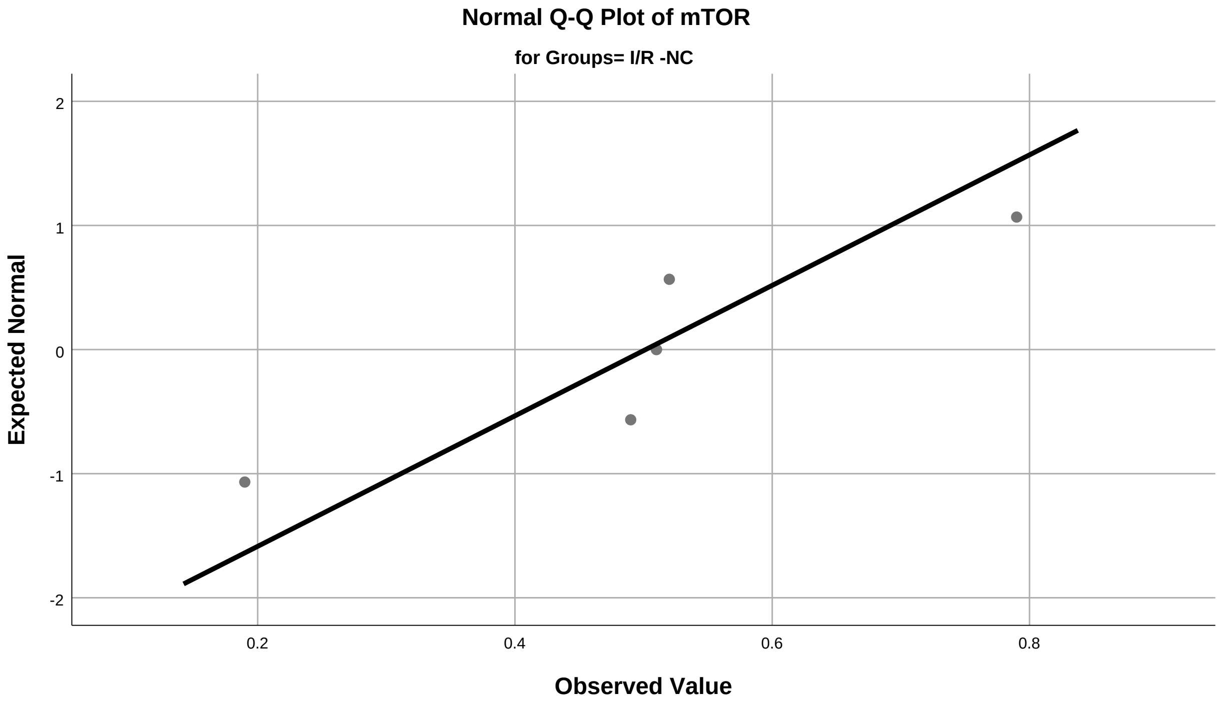
Normal Q-Q Plot of mTOR
for Groups= I/R -NC
Expected Normal
2
1
0
-1
-2
0.2
0.4
0.6
0.8
Observed Value

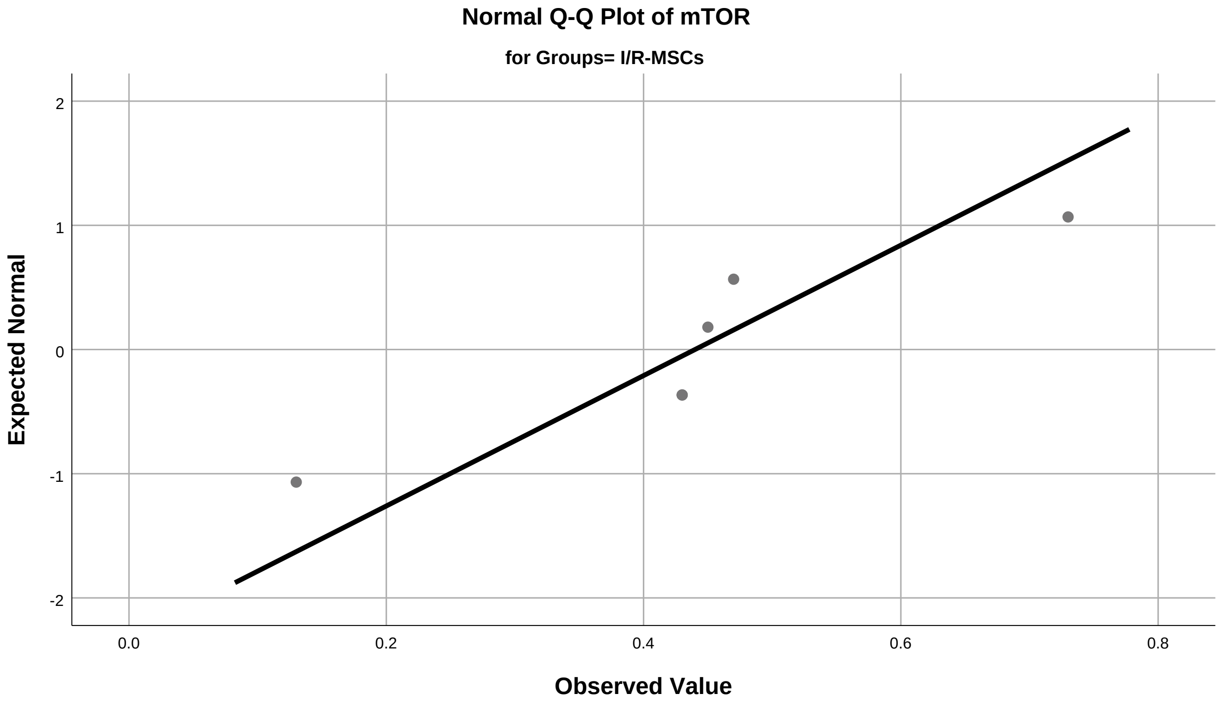
Normal Q-Q Plot of mTOR
for Groups= I/R-MSCs
Expected Normal
2
1
0
-1
-2
0.0
0.2
0.4
0.6
0.8
Observed Value

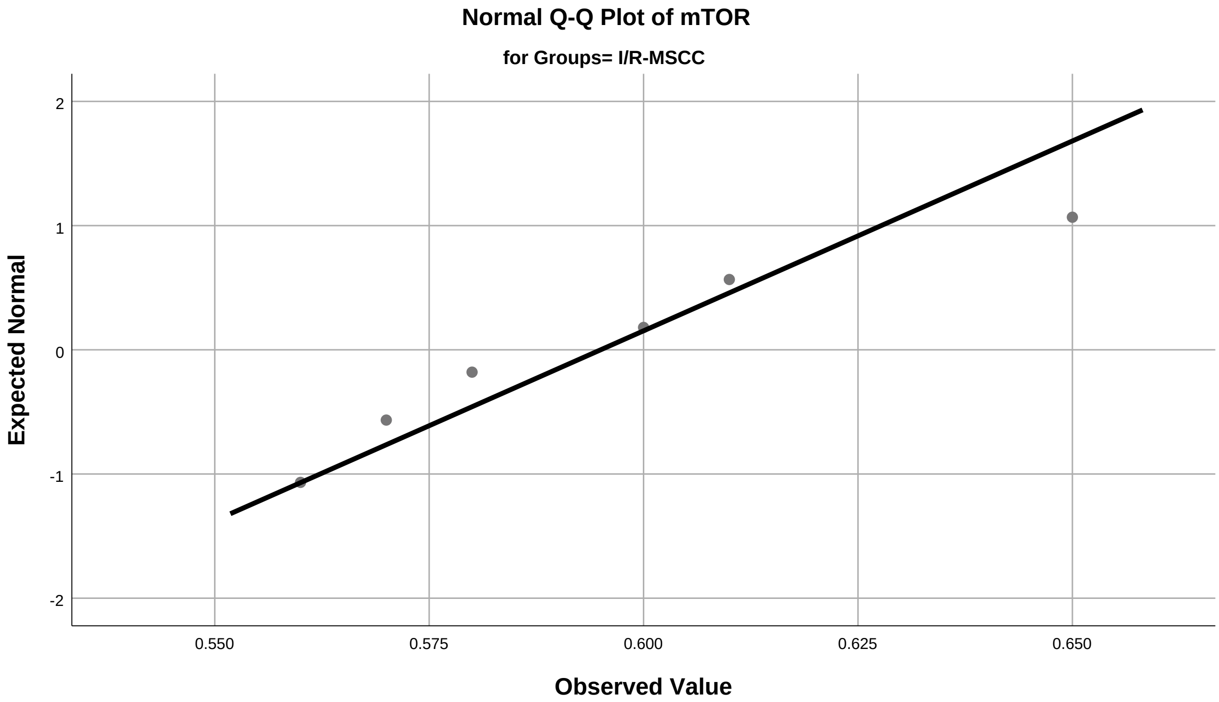

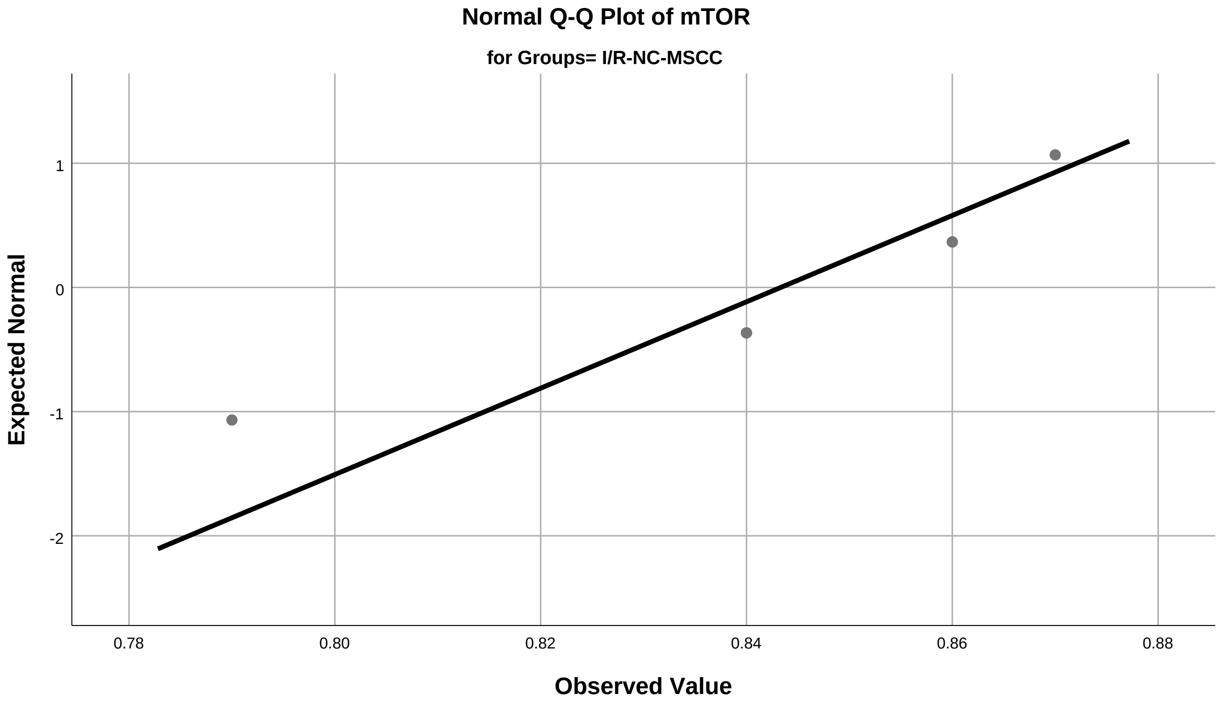

Detrended Normal Q-Q Plots

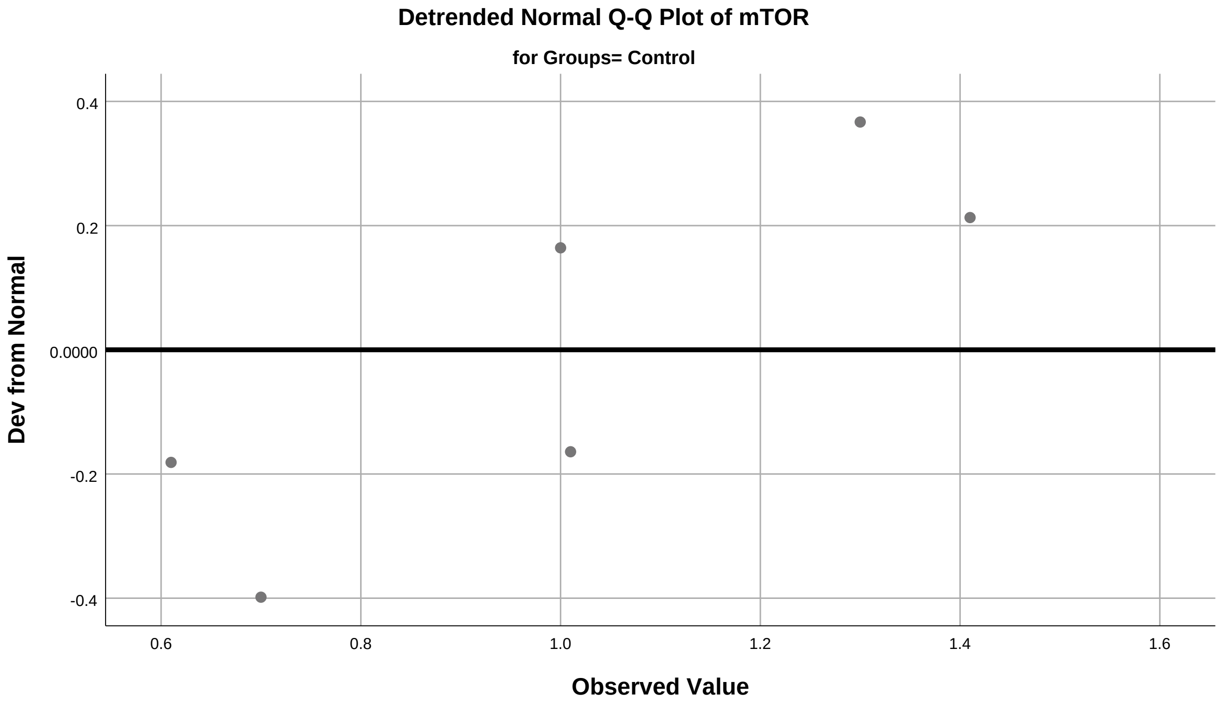
Detrended Normal Q-Q Plot of mTOR
for Groups= Control
Dev from Normal
0.4
0.2
0.0000
-0.2
-0.4
0.6
0.8
1.0
1.2
1.4
1.6
Observed Value

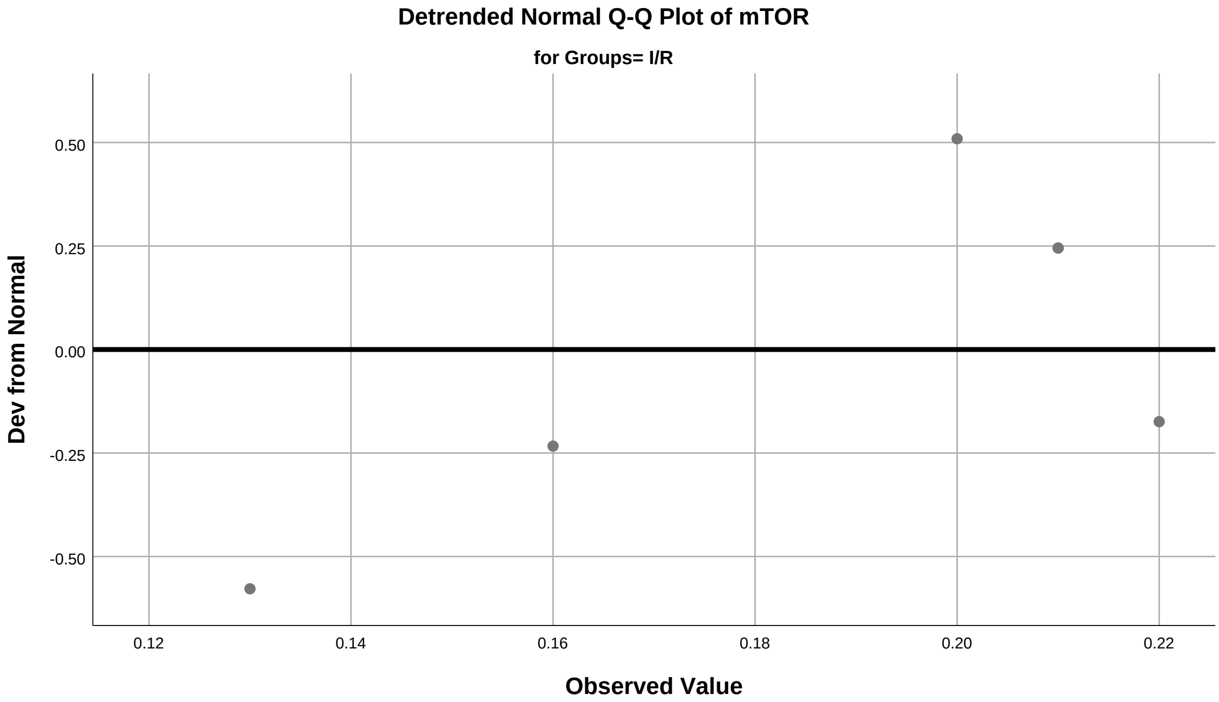
Detrended Normal Q-Q Plot of mTOR
for Groups= I/R
Dev from Normal
0.50
0.25
0.00
-0.25
-0.50
0.12
0.14
0.16
0.18
0.20
0.22
Observed Value

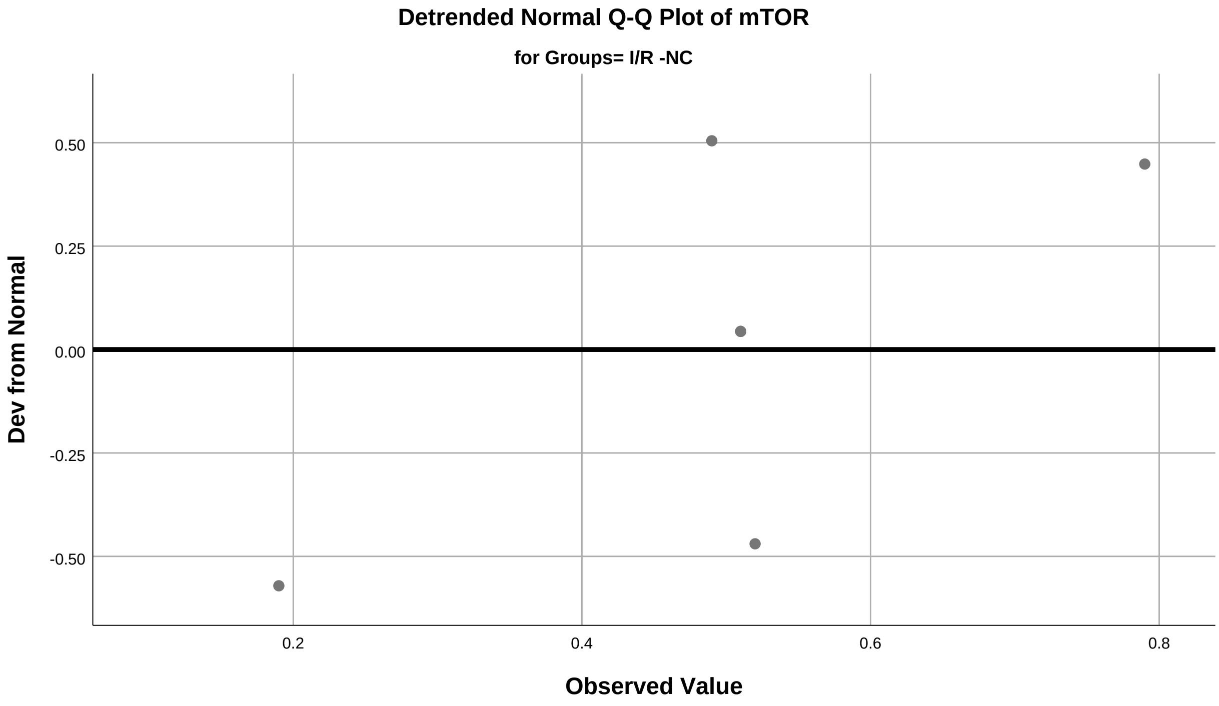
Detrended Normal Q-Q Plot of mTOR
for Groups= I/R -NC
Dev from Normal
0.50
0.25
0.00
-0.25
-0.50
0.2
0.4
0.6
0.8
Observed Value

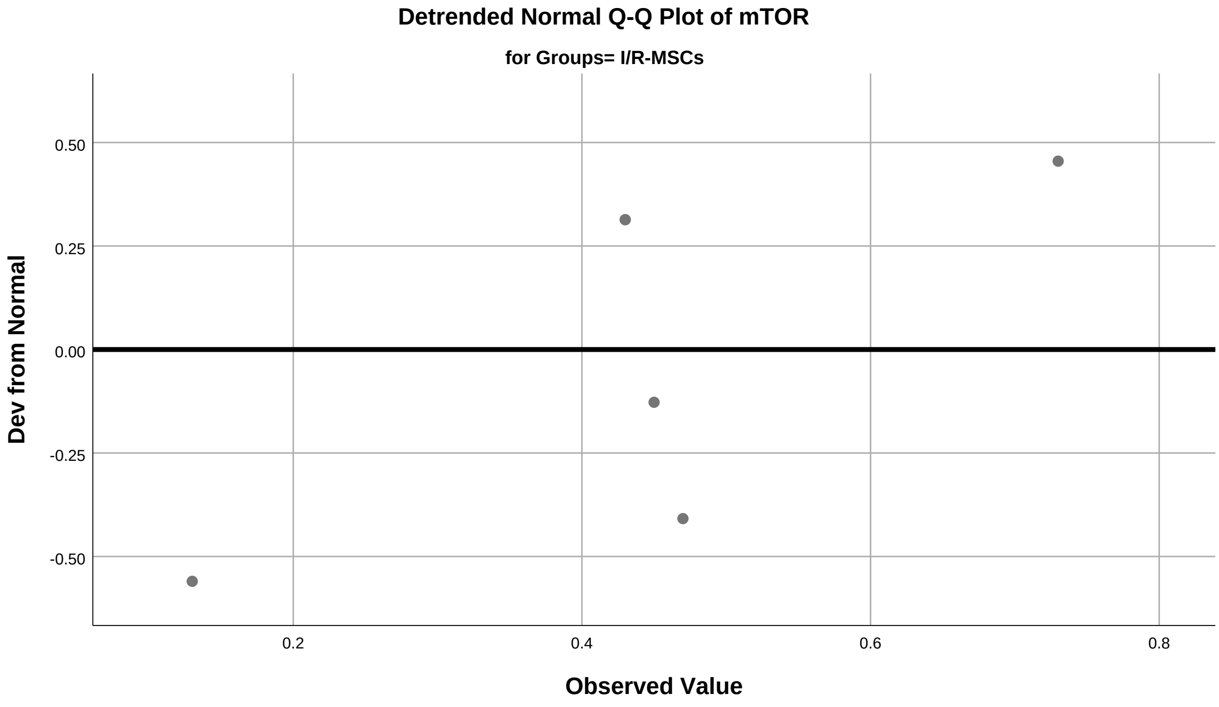
Detrended Normal Q-Q Plot of mTOR
for Groups= I/R-MSCs
Dev from Normal
0.50
0.25
0.00
-0.25
-0.50
0.2
0.4
0.6
0.8
Observed Value

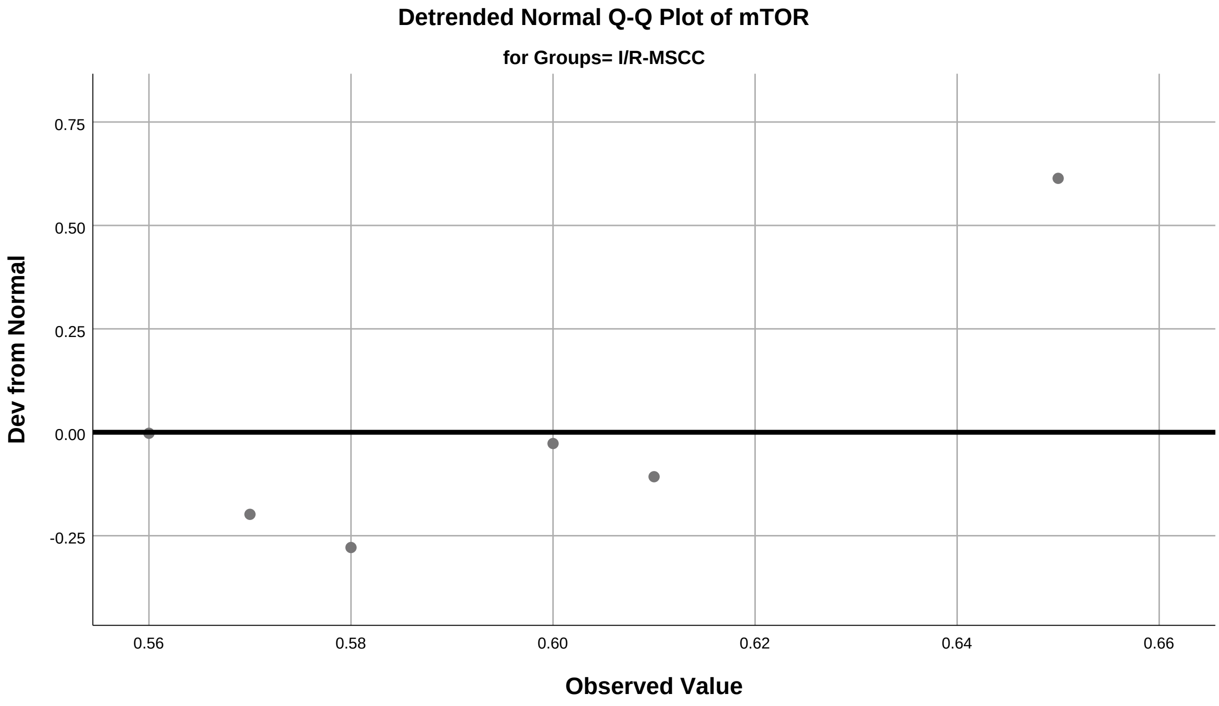
Detrended Normal Q-Q Plot of mTOR
for Groups= I/R-MSCC
Dev from Normal
0.75
0.50
0.25
0.00
-0.25
0.56
0.58
0.60
0.62
0.64
0.66
Observed Value

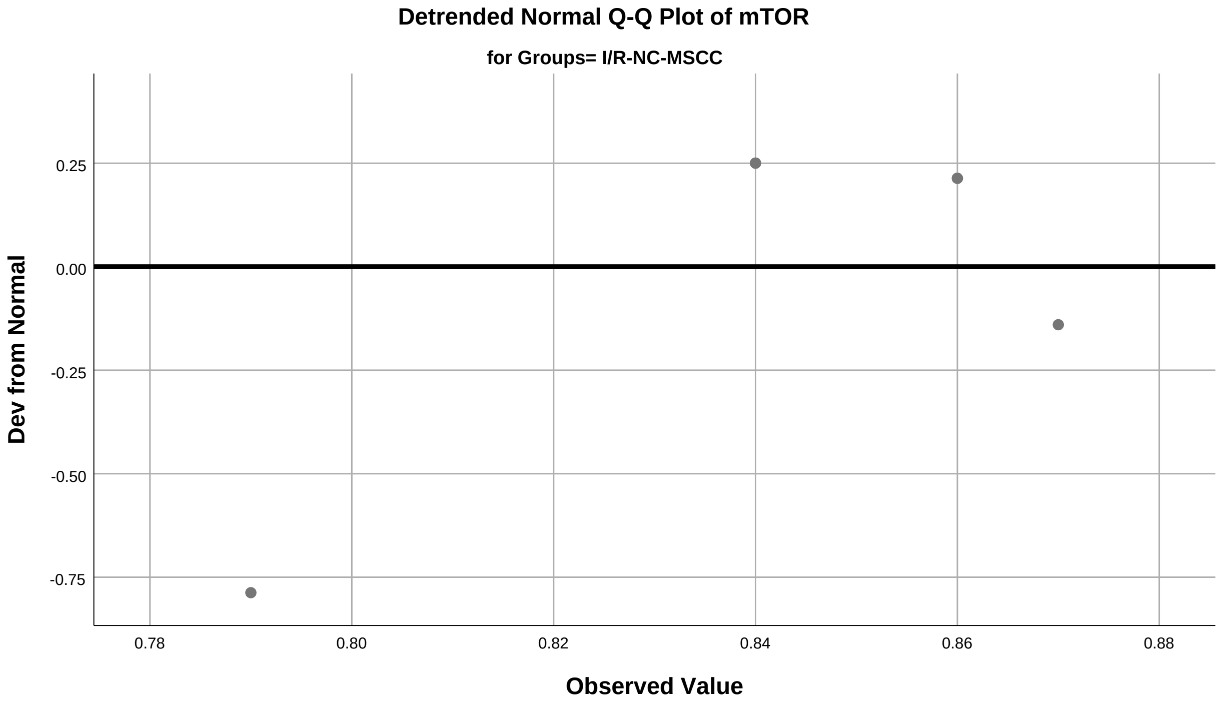
Detrended Normal Q-Q Plot of mTOR
for Groups= I/R-NC-MSCC
Dev from Normal
0.25
0.00
-0.25
-0.50
-0.75
0.78
0.80
0.82
0.84
0.86
0.88
Observed Value

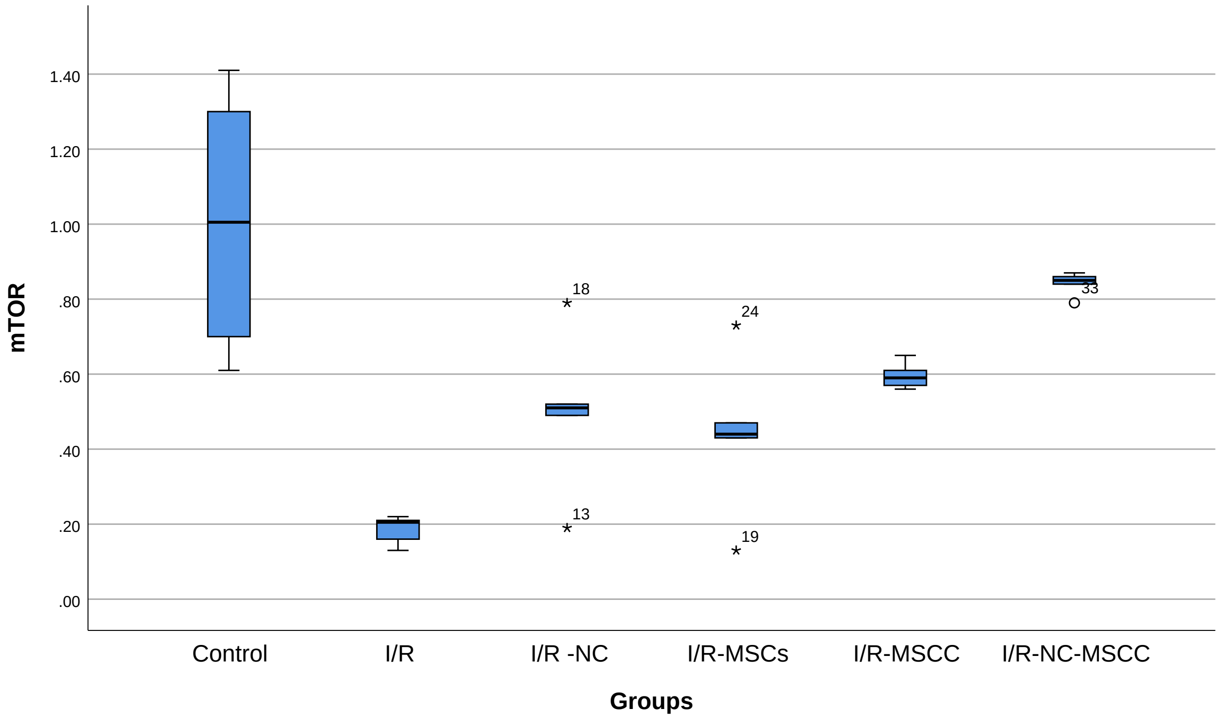

caspaseexocrine

Normal Q-Q Plots

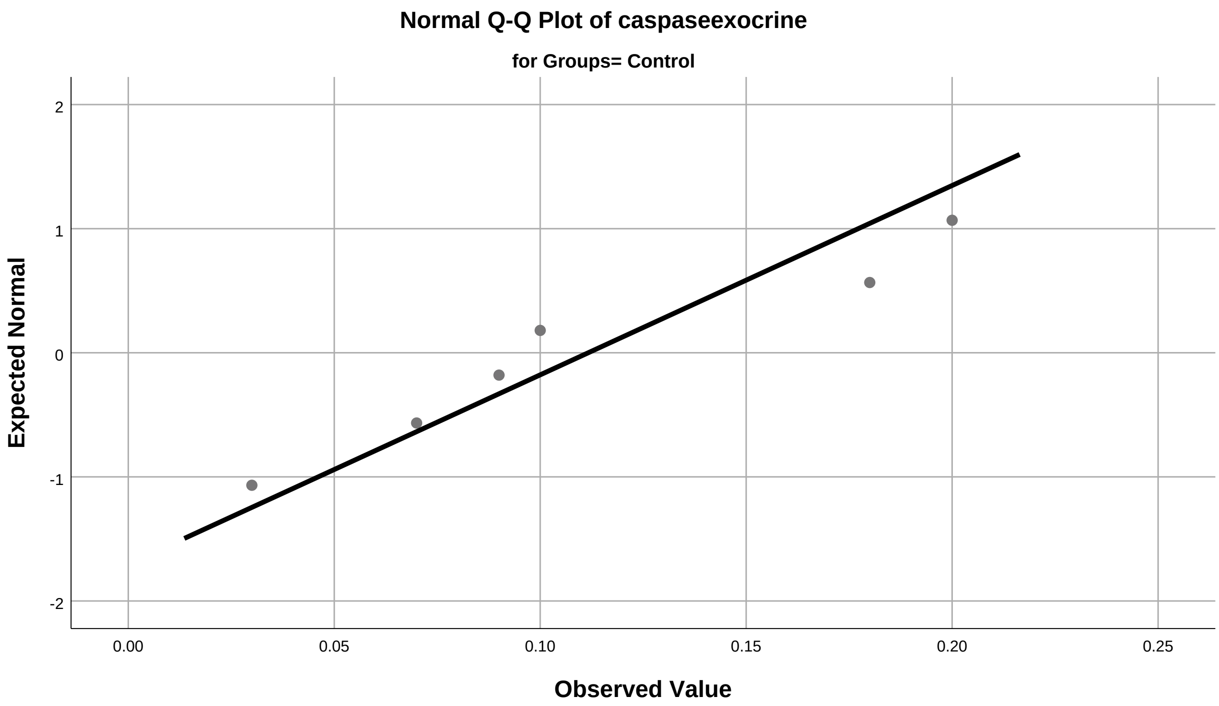

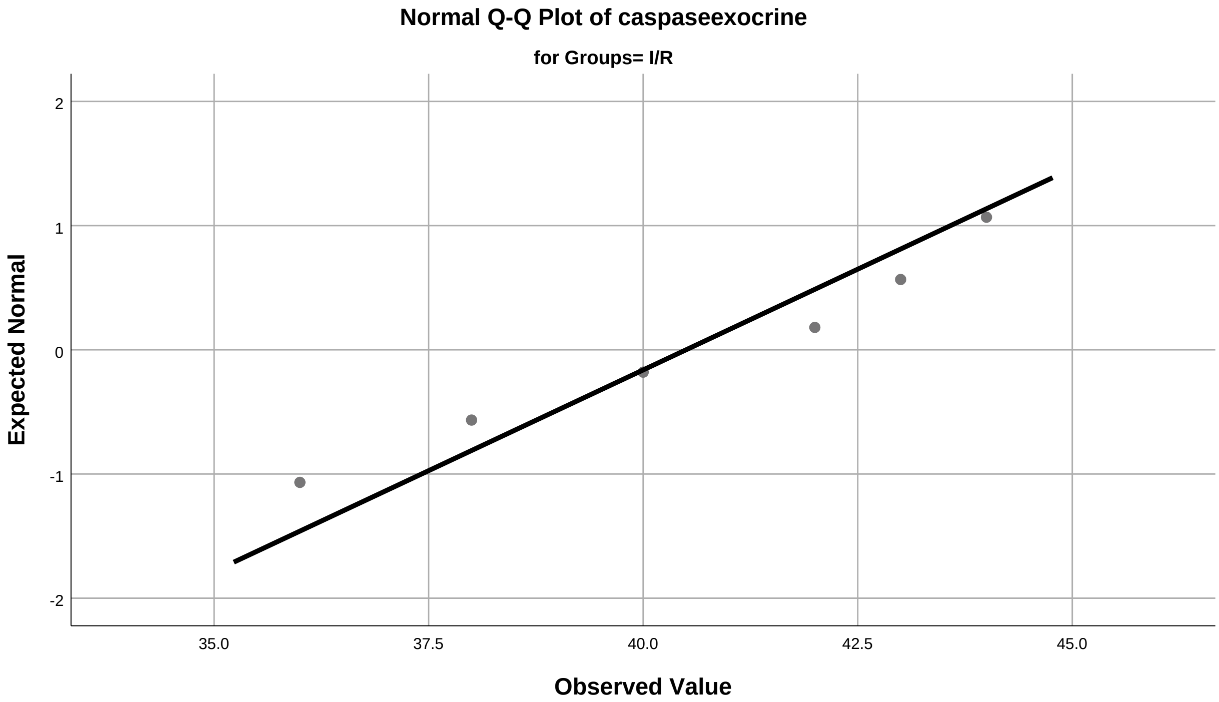
Normal Q-Q Plot of caspaseexocrine
for Groups= I/R
Expected Normal
2
1
0
-1
-2
35.0
37.5
40.0
42.5
45.0
Observed Value

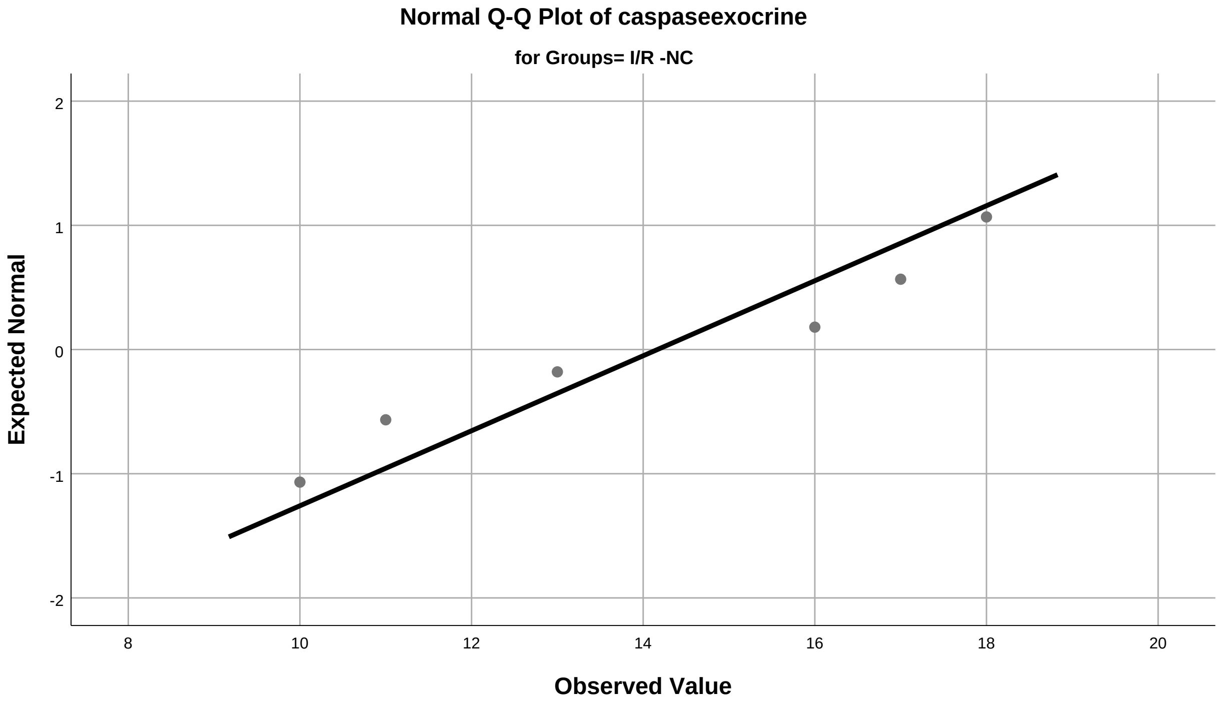
Normal Q-Q Plot of caspaseexocrine
for Groups= I/R -NC
Expected Normal
2
1
0
-1
-2
8
10
12
14
16
18
20
Observed Value

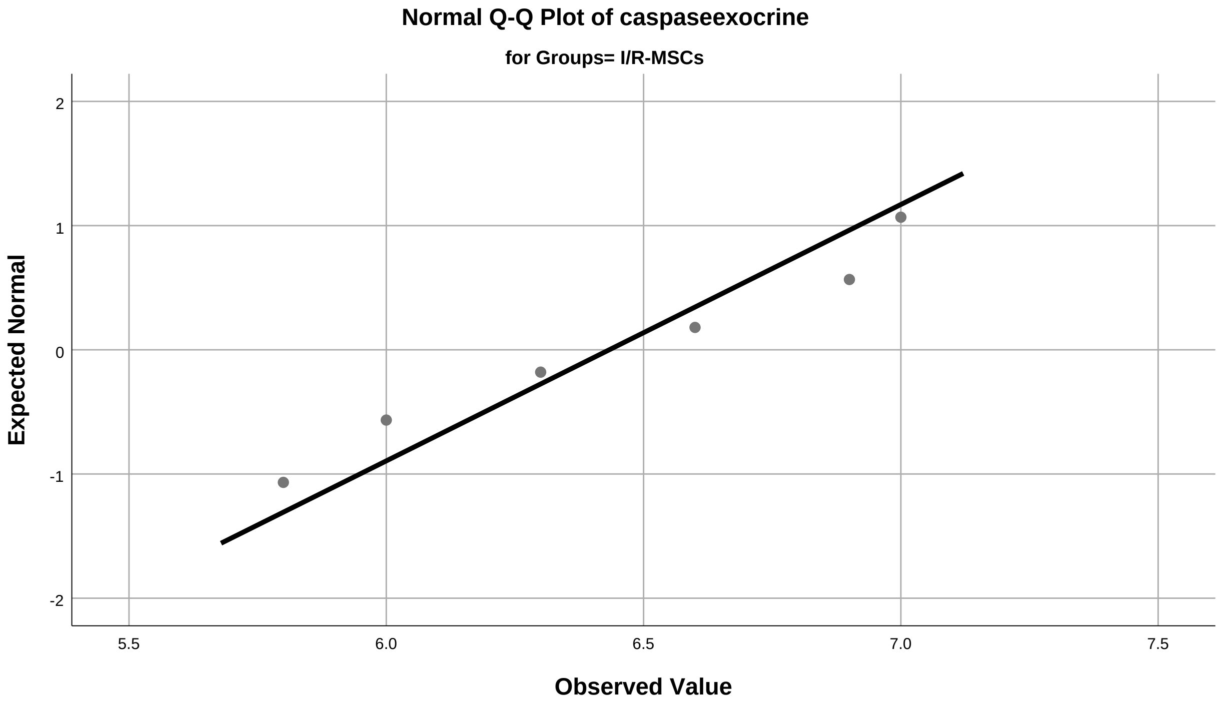
Normal Q-Q Plot of caspaseexocrine
for Groups= I/R-MSCs
Expected Normal
2
1
0
-1
-2
5.5
6.0
6.5
7.0
7.5
Observed Value

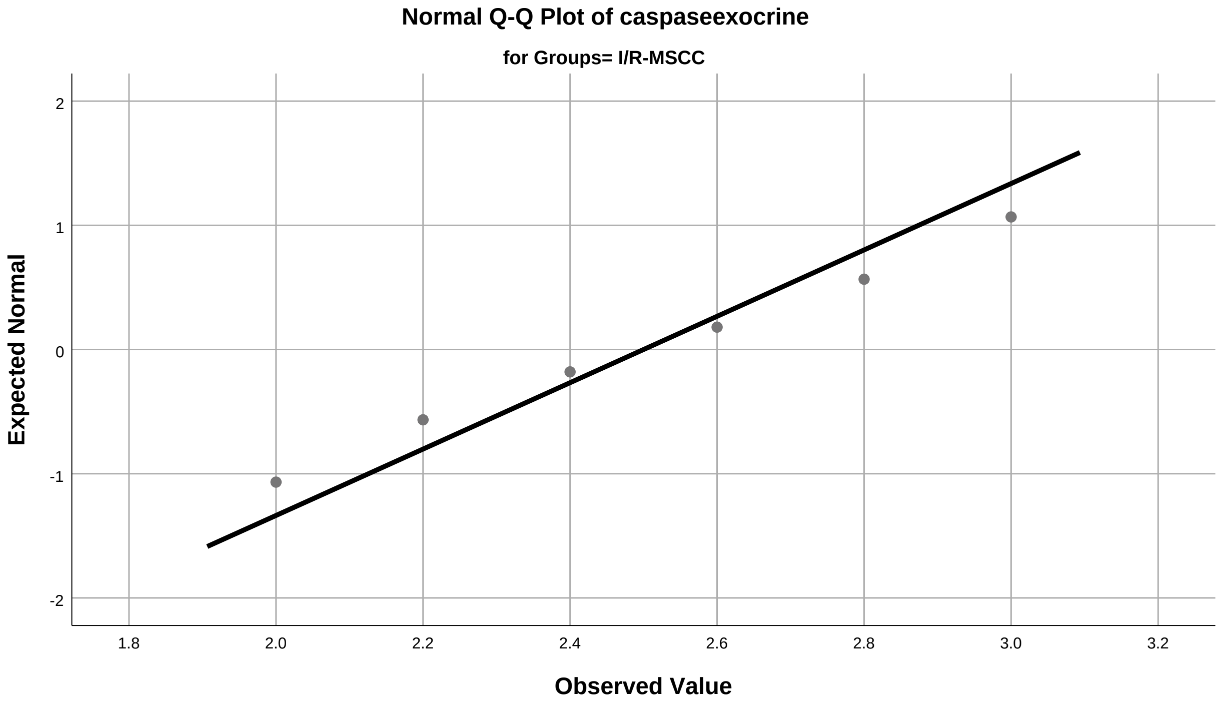
Normal Q-Q Plot of caspaseexocrine
for Groups= I/R-MSCC
Expected Normal
2
1
0
-1
-2
1.8
2.0
2.2
2.4
2.6
2.8
3.0
3.2
Observed Value

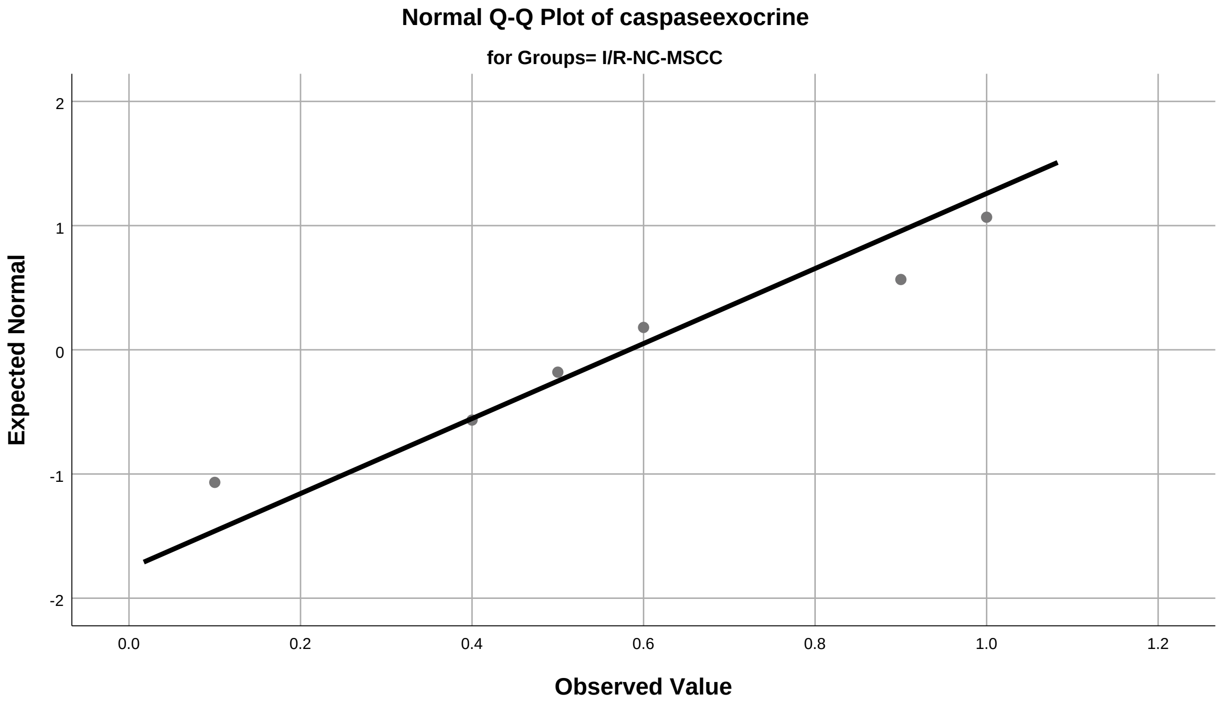

Detrended Normal Q-Q Plots

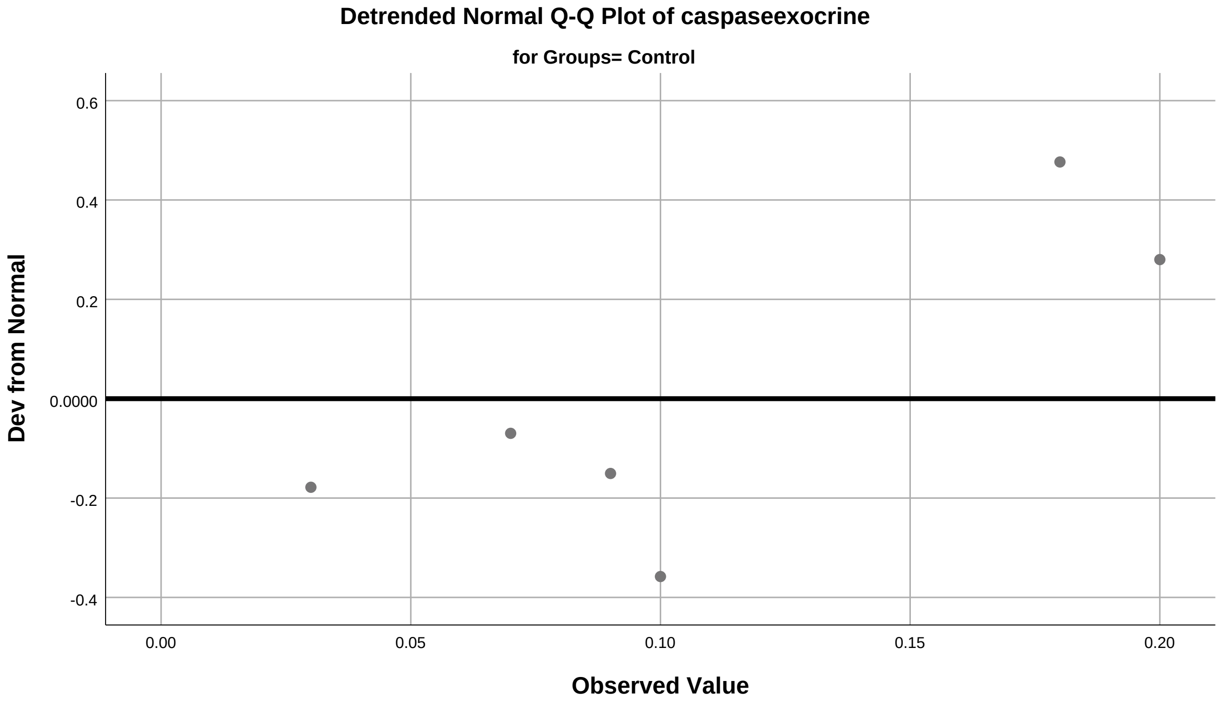

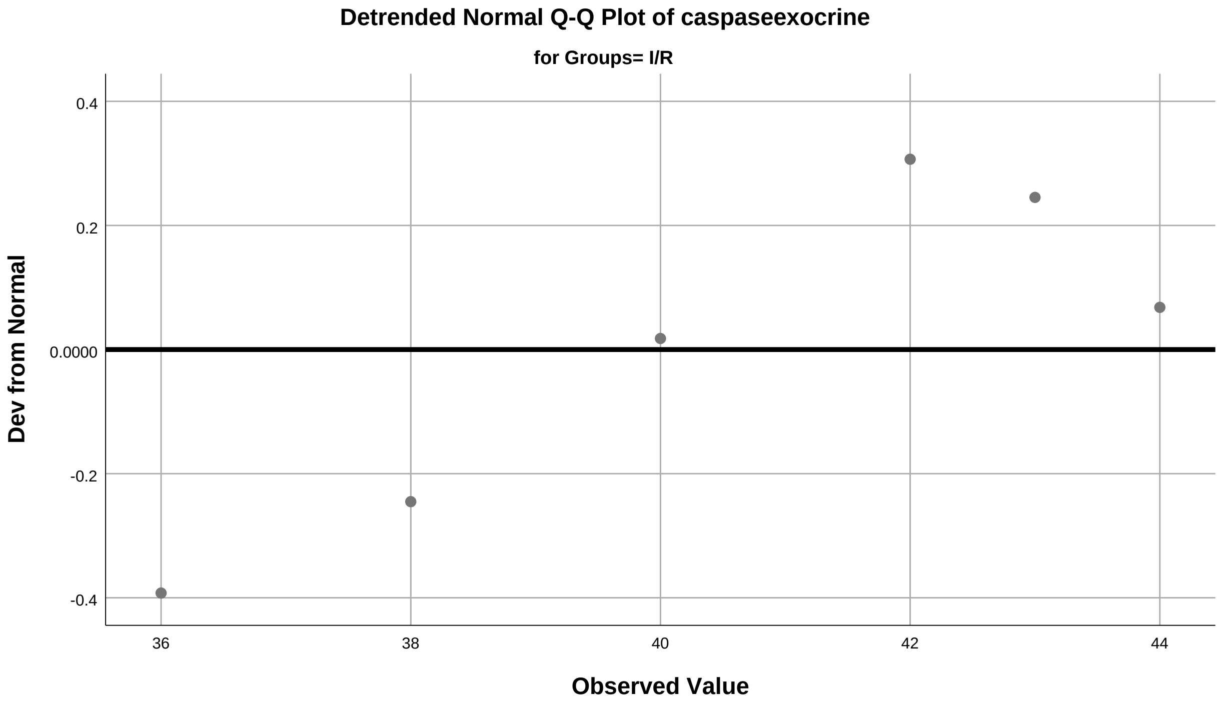
Detrended Normal Q-Q Plot of caspaseexocrine
for Groups= I/R
Dev from Normal
0.4
0.2
0.0000
-0.2
-0.4
36
38
40
42
44
Observed Value

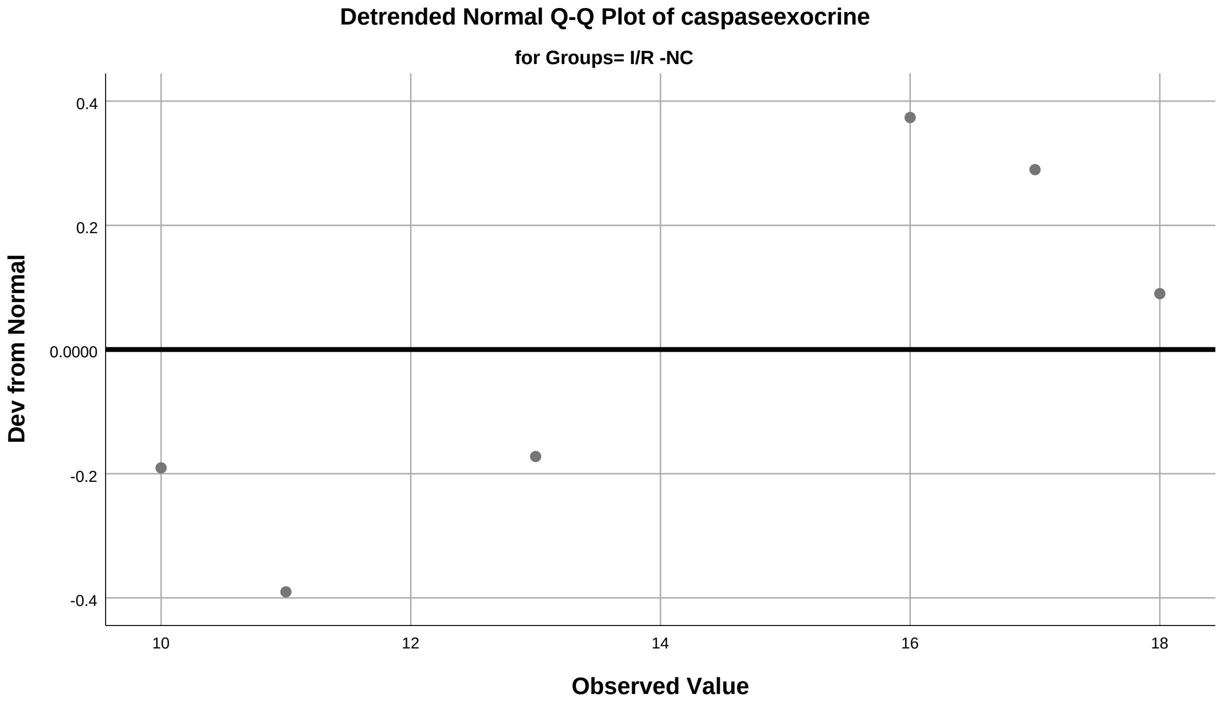
Detrended Normal Q-Q Plot of caspaseexocrine
for Groups= I/R -NC
Dev from Normal
0.4
0.2
0.0000
-0.2
-0.4
10
12
14
16
18
Observed Value

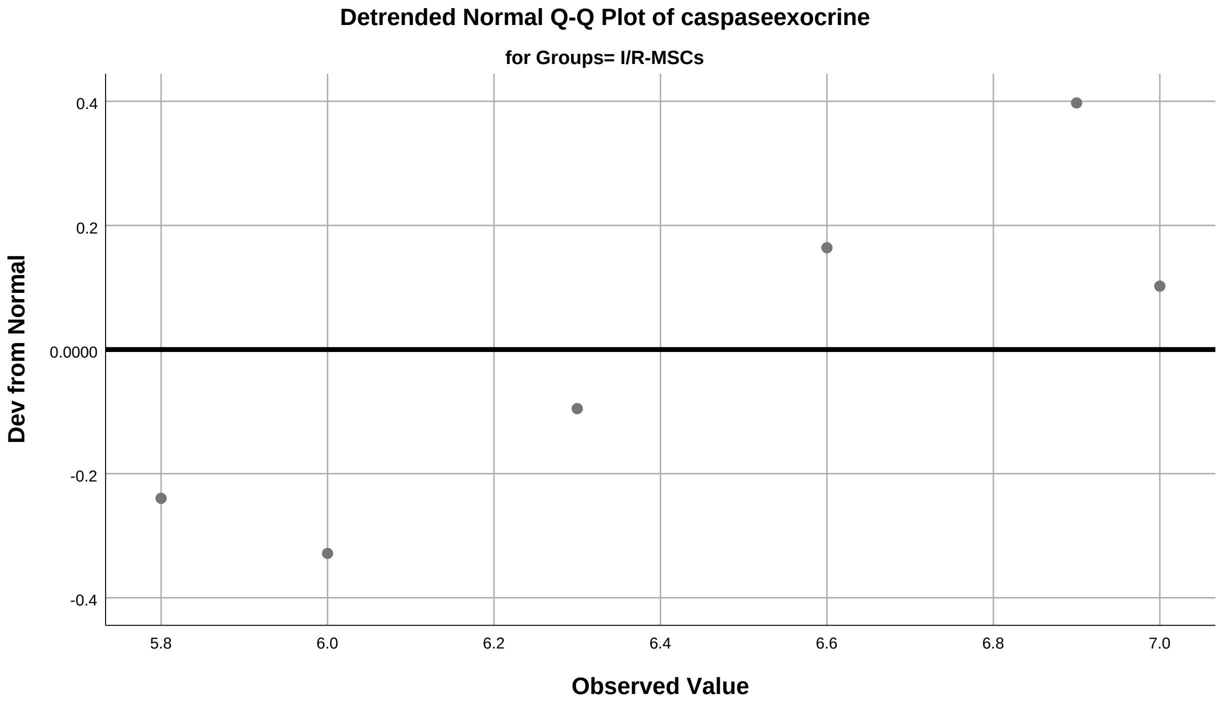
Detrended Normal Q-Q Plot of caspaseexocrine
for Groups= I/R-MSCs
0.4
0.2
0.0000
-0.2
-0.4
Dev from Normal
5.8
6.0
6.2
6.4
6.6
6.8
7.0
Observed Value

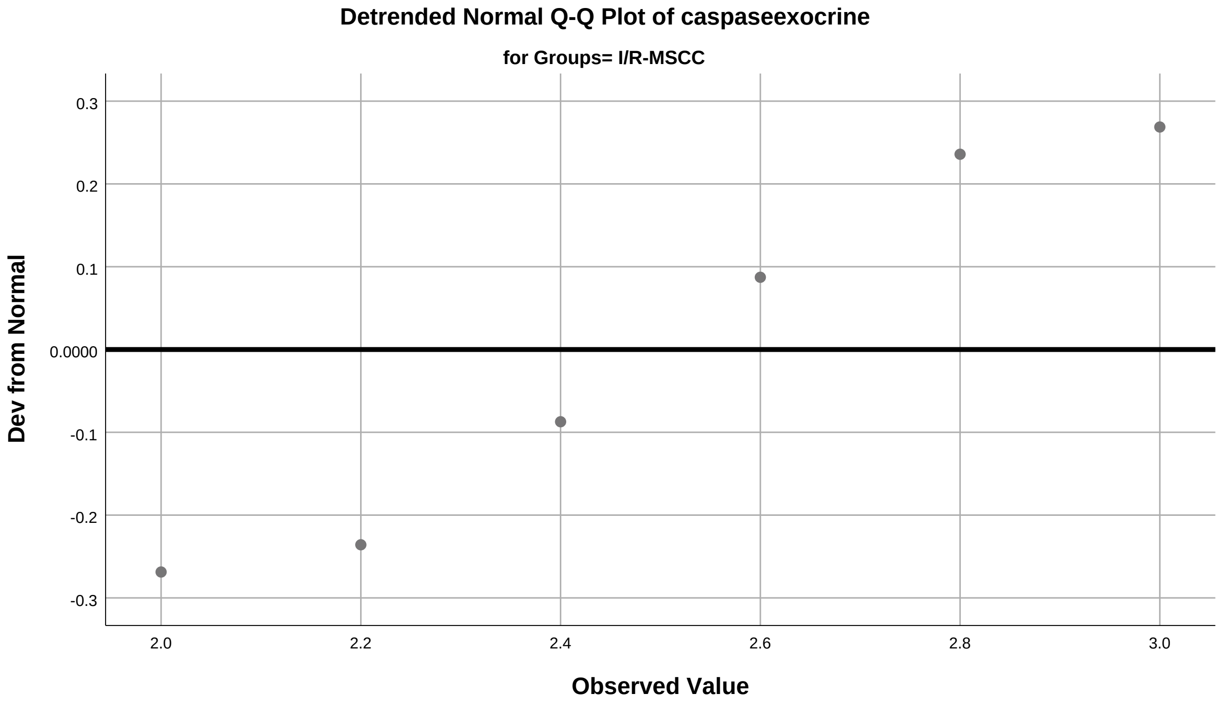
Detrended Normal Q-Q Plot of caspaseexocrine
for Groups= I/R-MSCC
0.3
0.2
0.1
0.0000
-0.1
-0.2
-0.3
Dev from Normal
2.0
2.2
2.4
2.6
2.8
3.0
Observed Value

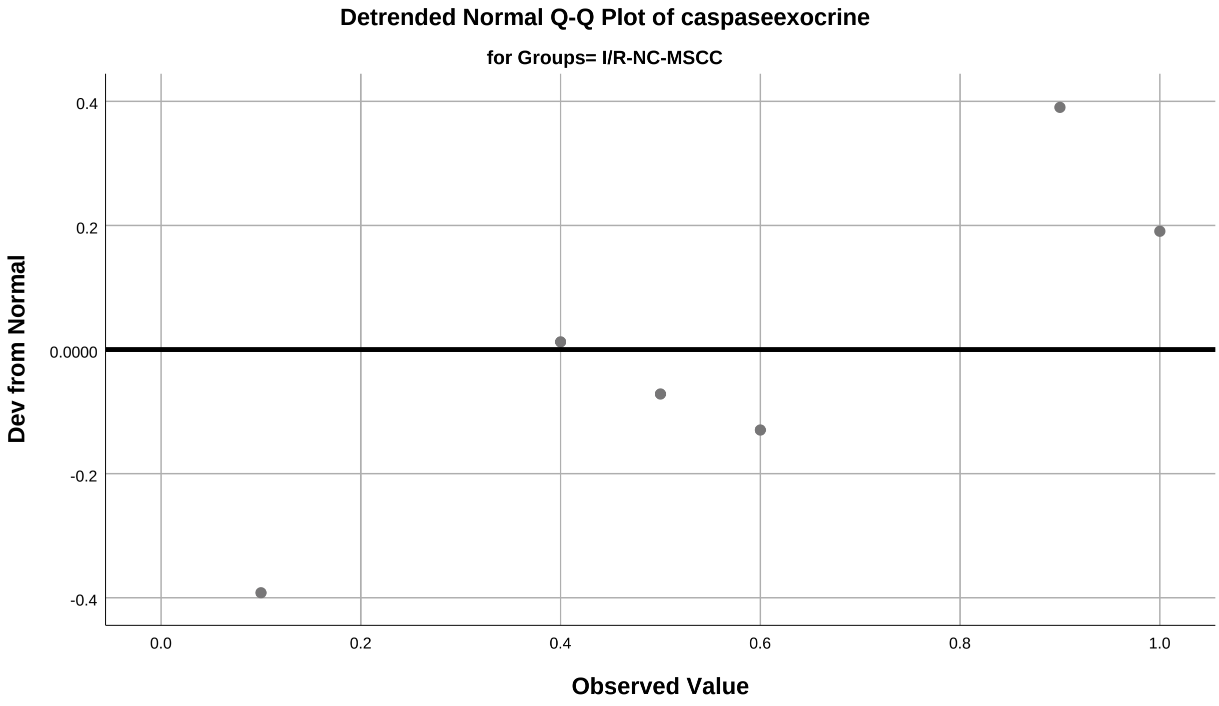

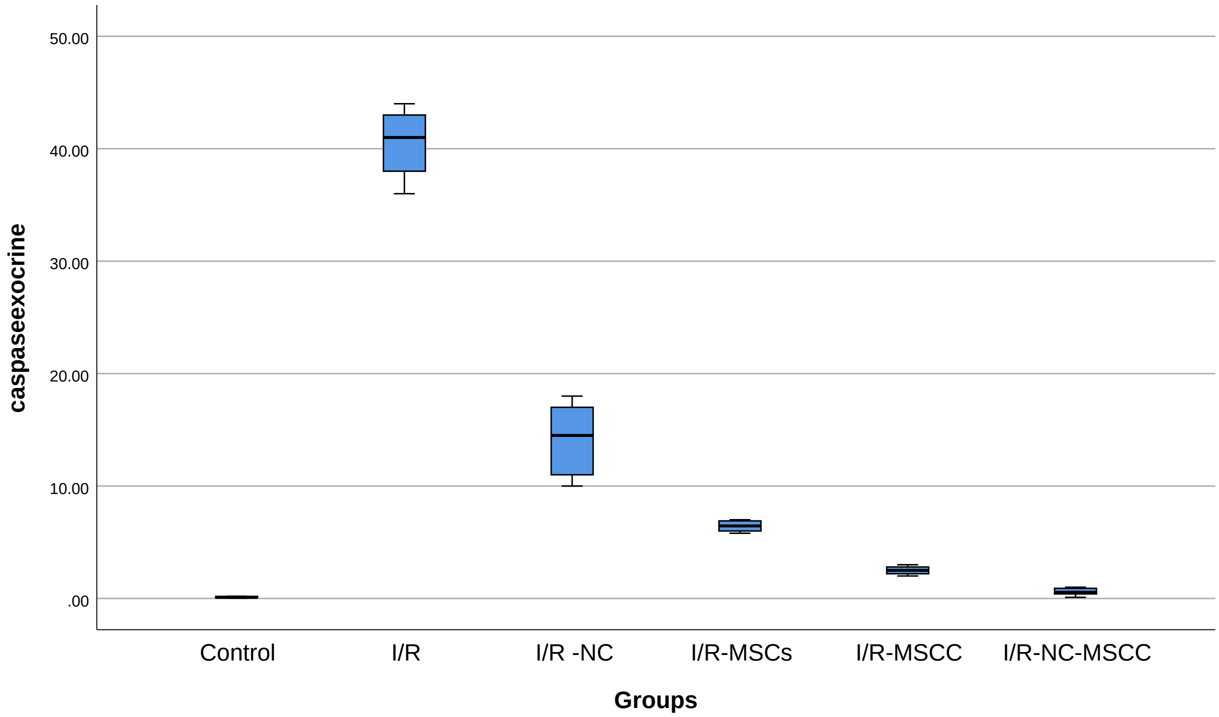

caspaseendocrine

Normal Q-Q Plots

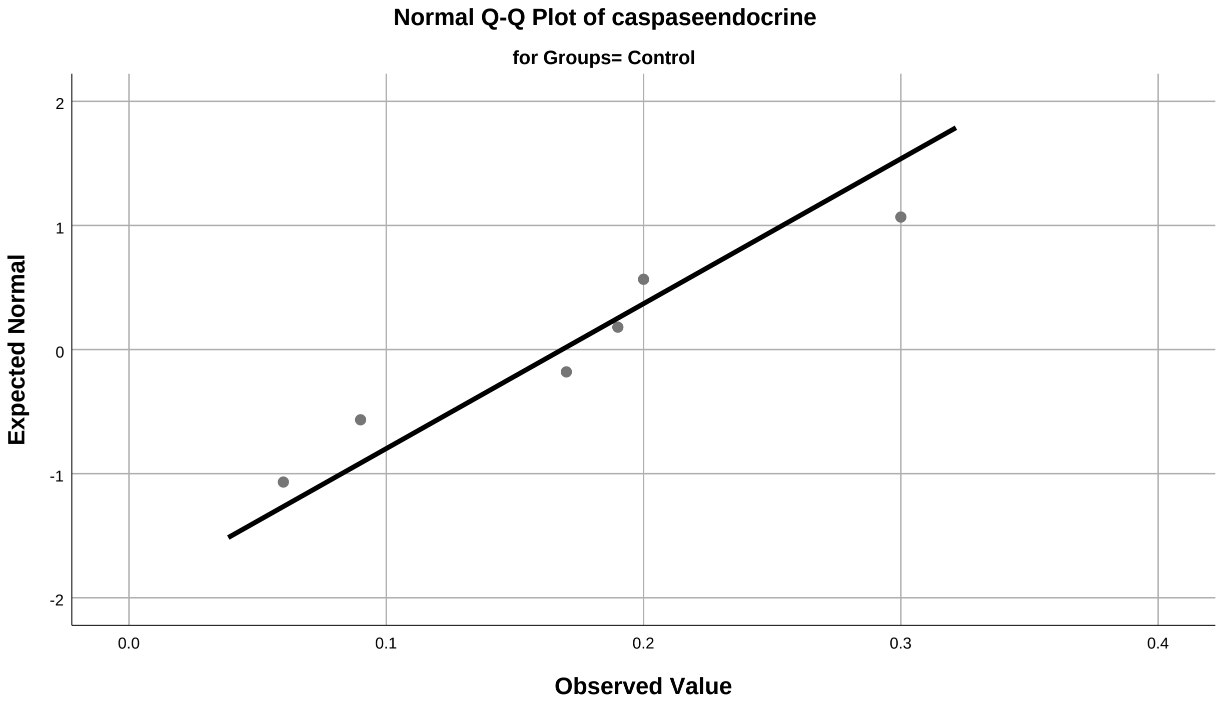
Normal Q-Q Plot of caspaseendocrine
for Groups= Control
Expected Normal
2
1
0
-1
-2
0.0
0.1
0.2
0.3
0.4
Observed Value

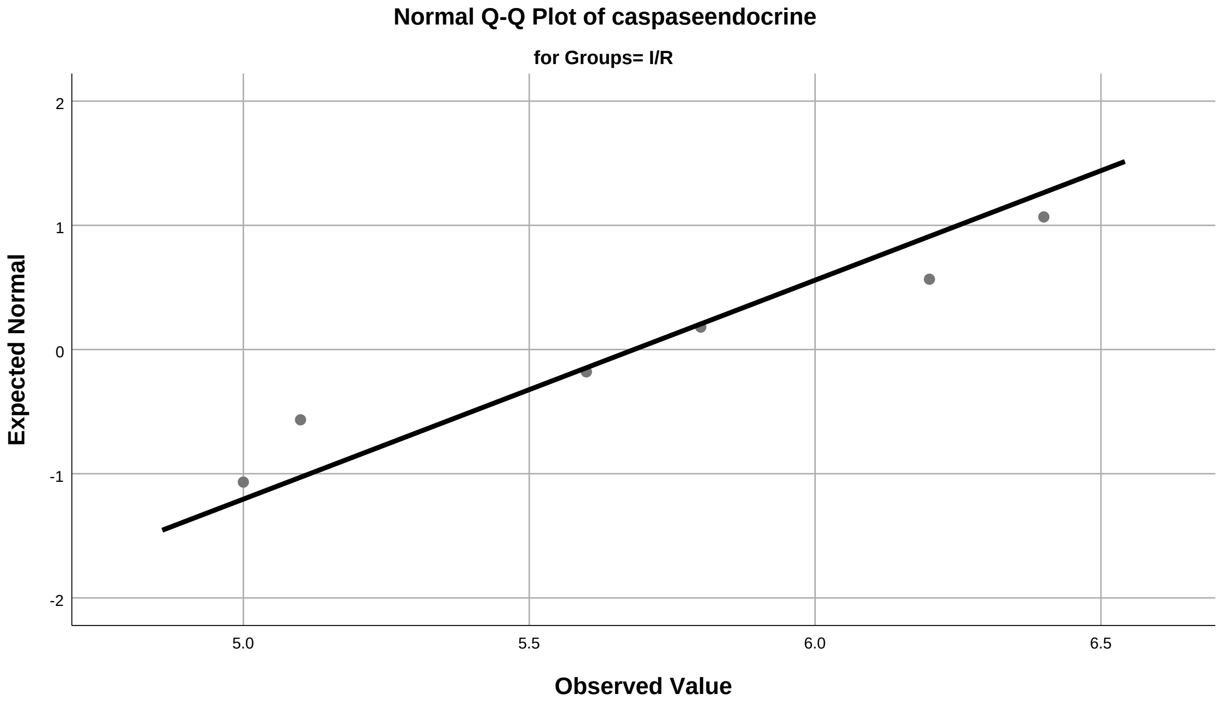
Normal Q-Q Plot of caspaseendocrine
for Groups= I/R
Expected Normal
2
1
0
-1
-2
5.0
5.5
6.0
6.5
Observed Value

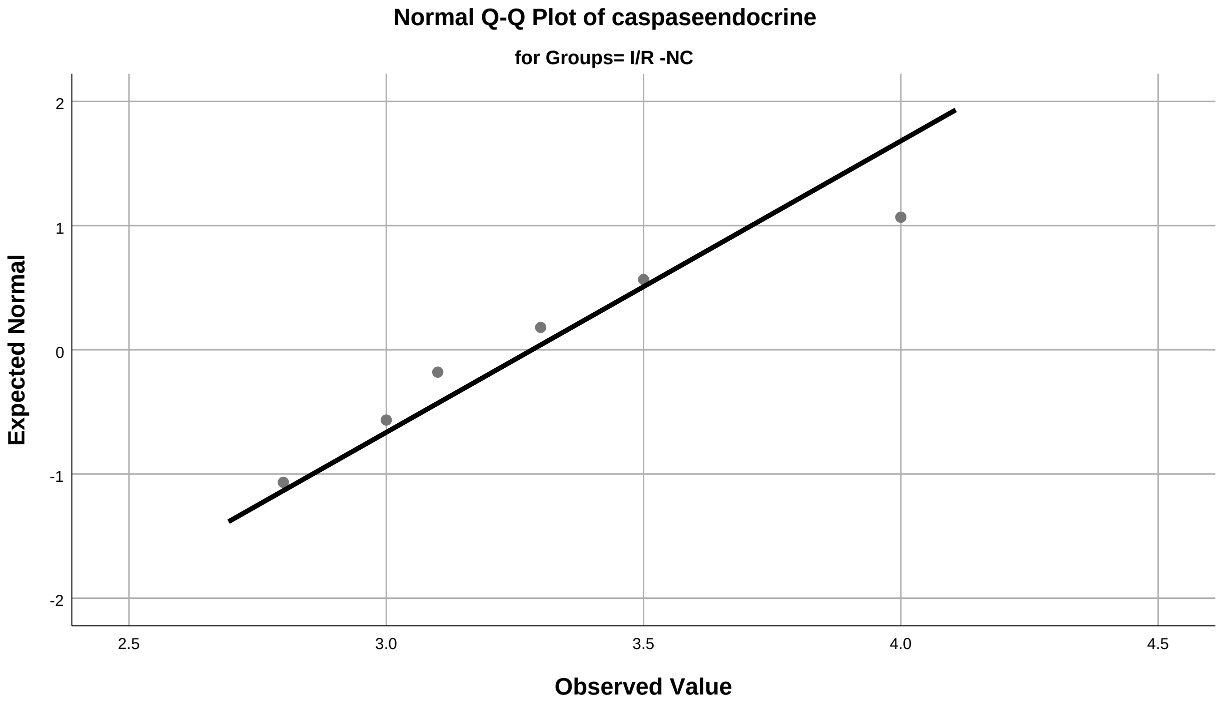
Normal Q-Q Plot of caspaseendocrine
for Groups= I/R -NC
Expected Normal
2
1
0
-1
-2
2.5
3.0
3.5
4.0
4.5
Observed Value

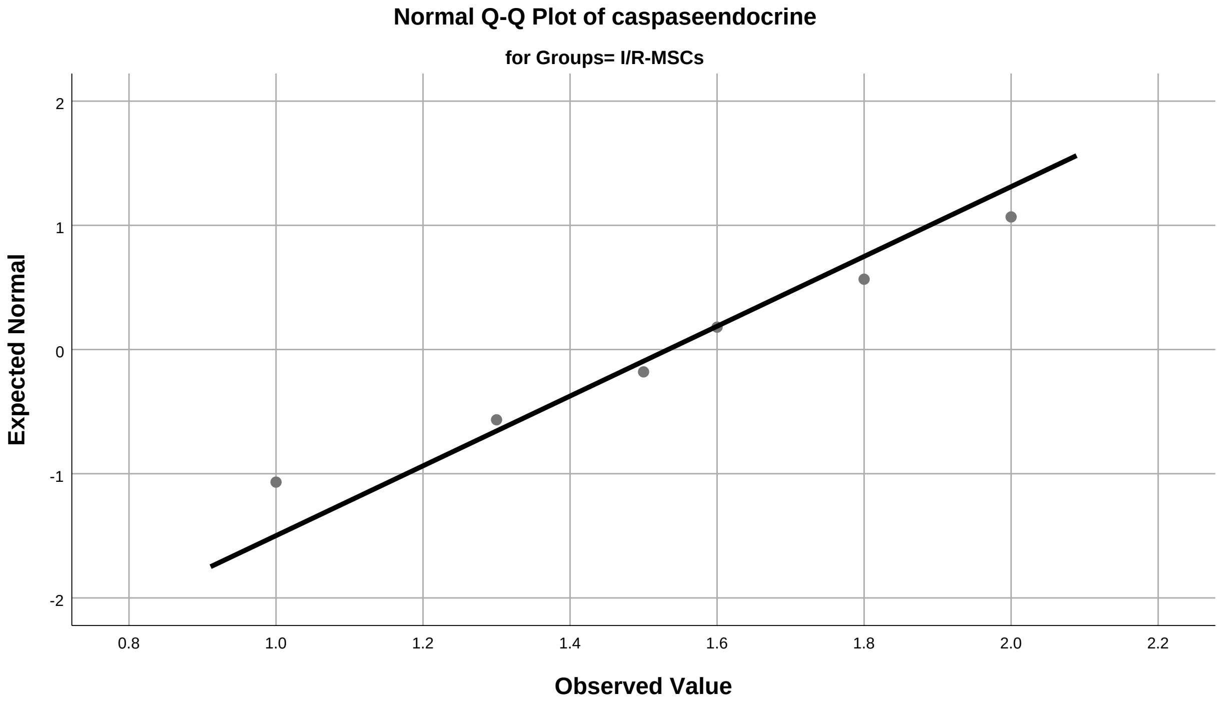
Normal Q-Q Plot of caspaseendocrine
for Groups= I/R-MSCs
Expected Normal
2
1
0
-1
-2
0.8
1.0
1.2
1.4
1.6
1.8
2.0
2.2
Observed Value

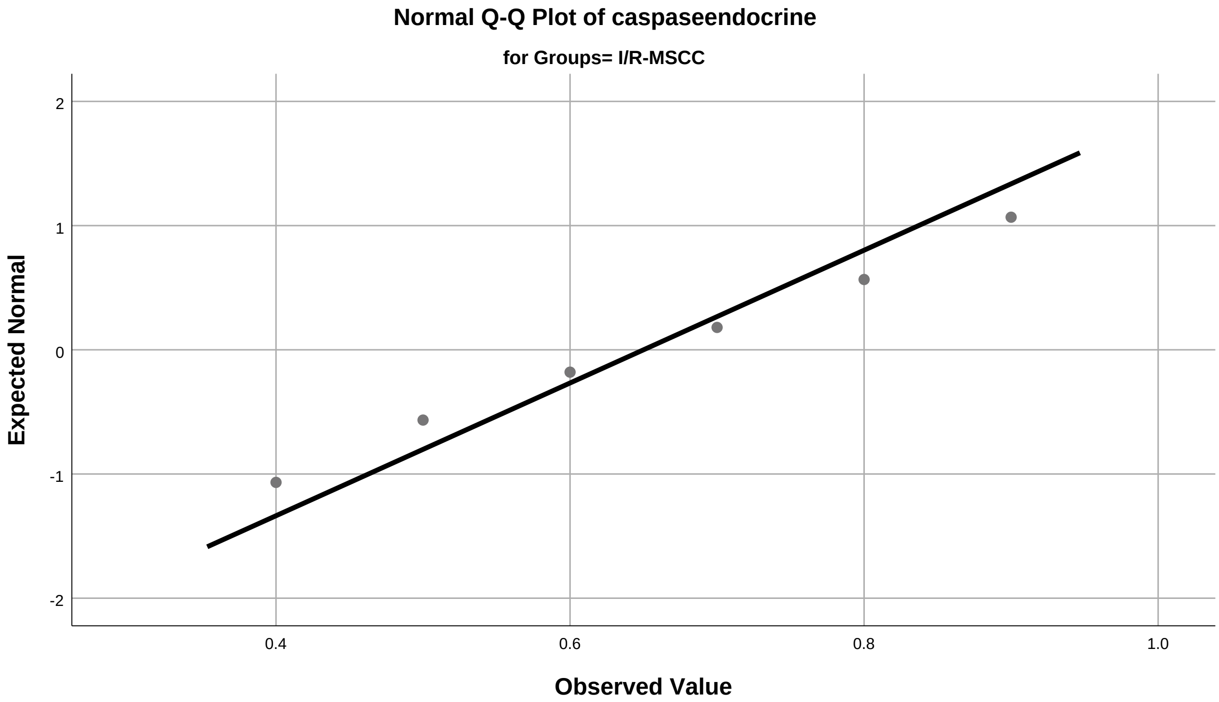

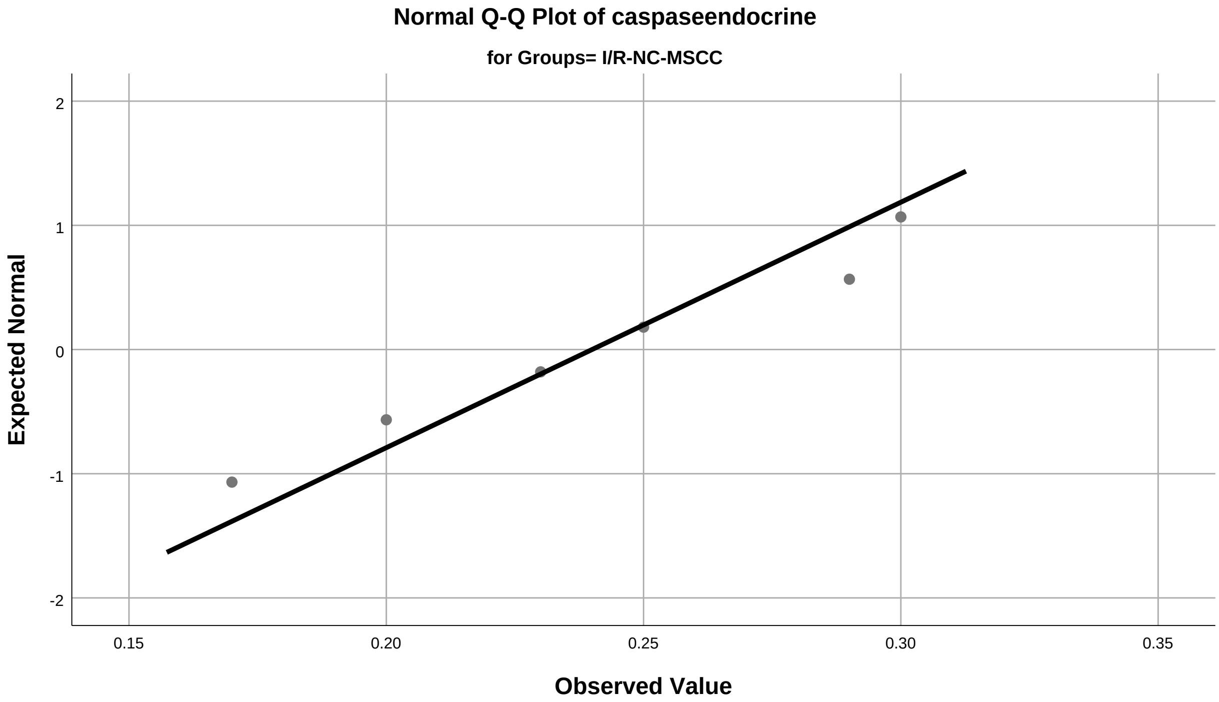

Detrended Normal Q-Q Plots

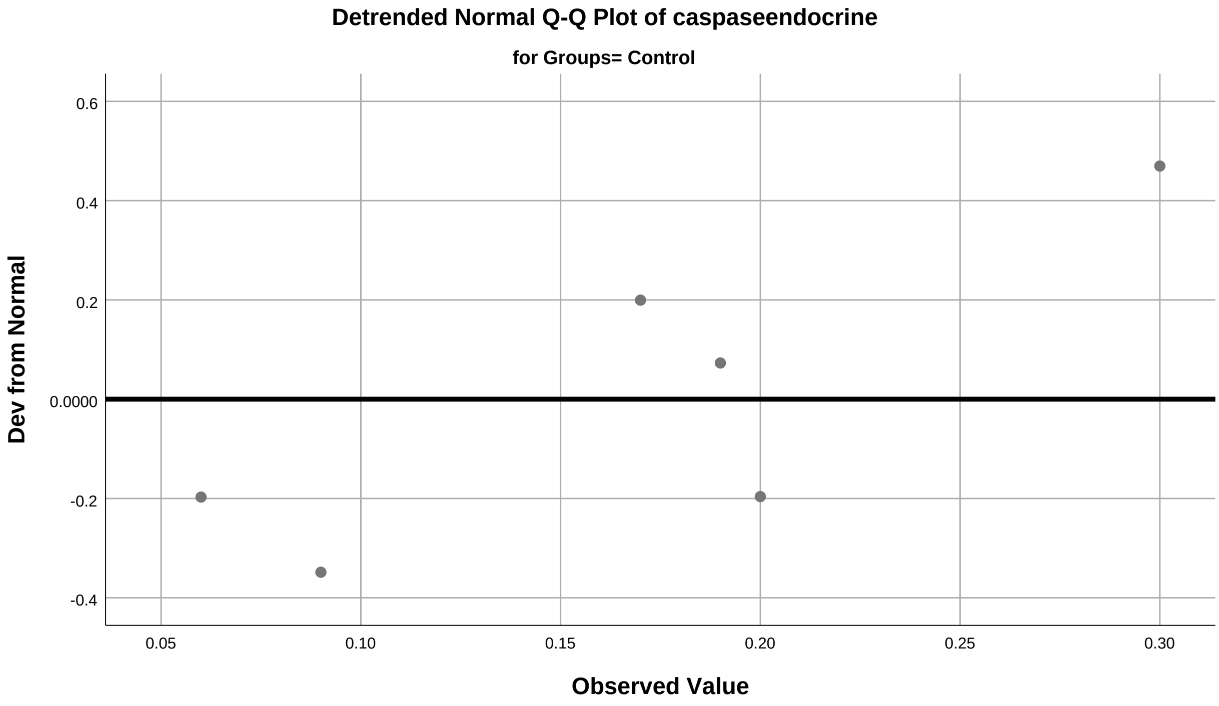
Detrended Normal Q-Q Plot of caspaseendocrine
for Groups= Control
Dev from Normal
0.6
0.4
0.2
0.0000
-0.2
-0.4
0.05
0.10
0.15
0.20
0.25
0.30
Observed Value

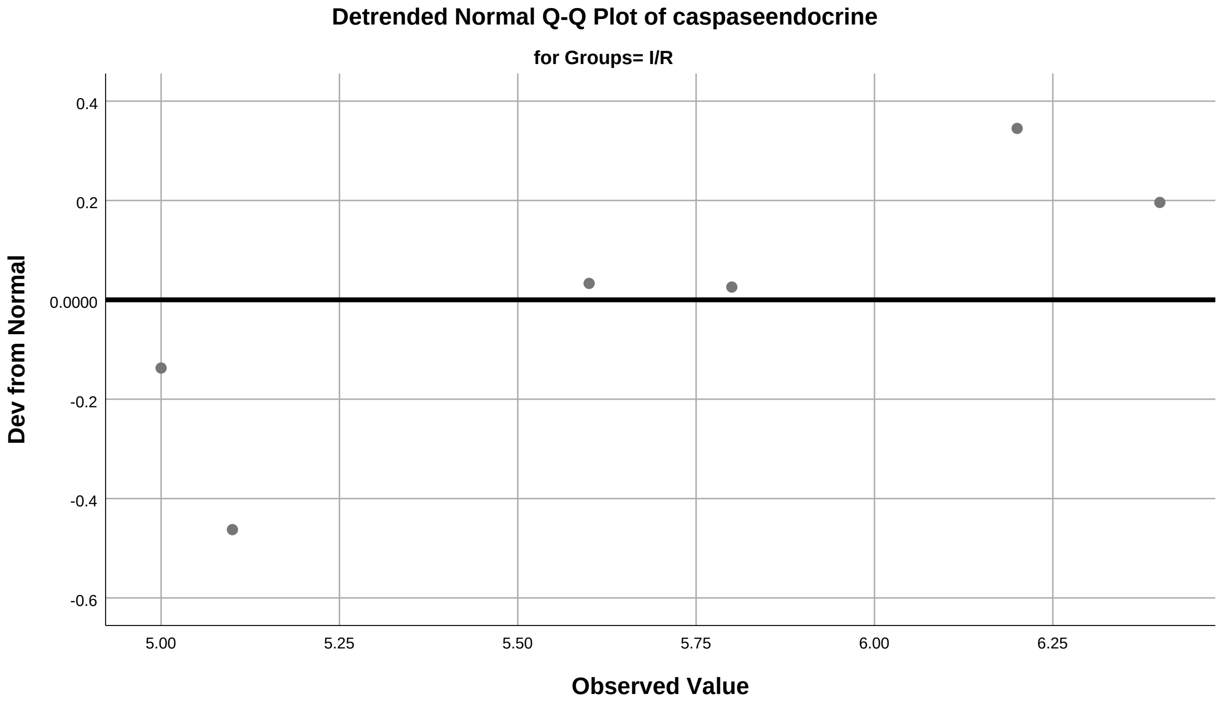
Detrended Normal Q-Q Plot of caspaseendocrine
for Groups= I/R
Dev from Normal
0.4
0.2
0.0000
-0.2
-0.4
-0.6
5.00
5.25
5.50
5.75
6.00
6.25
Observed Value

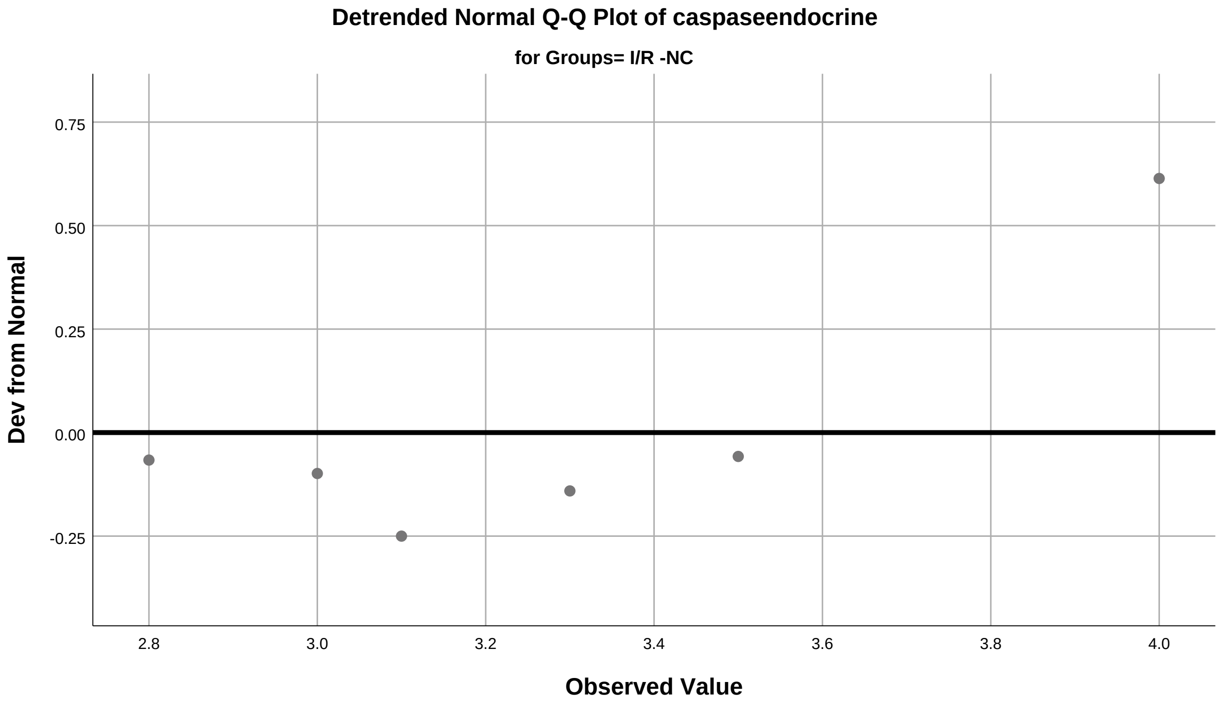
Detrended Normal Q-Q Plot of caspaseendocrine
for Groups= I/R -NC
Dev from Normal
0.75
0.50
0.25
0.00
-0.25
2.8
3.0
3.2
3.4
3.6
3.8
4.0
Observed Value

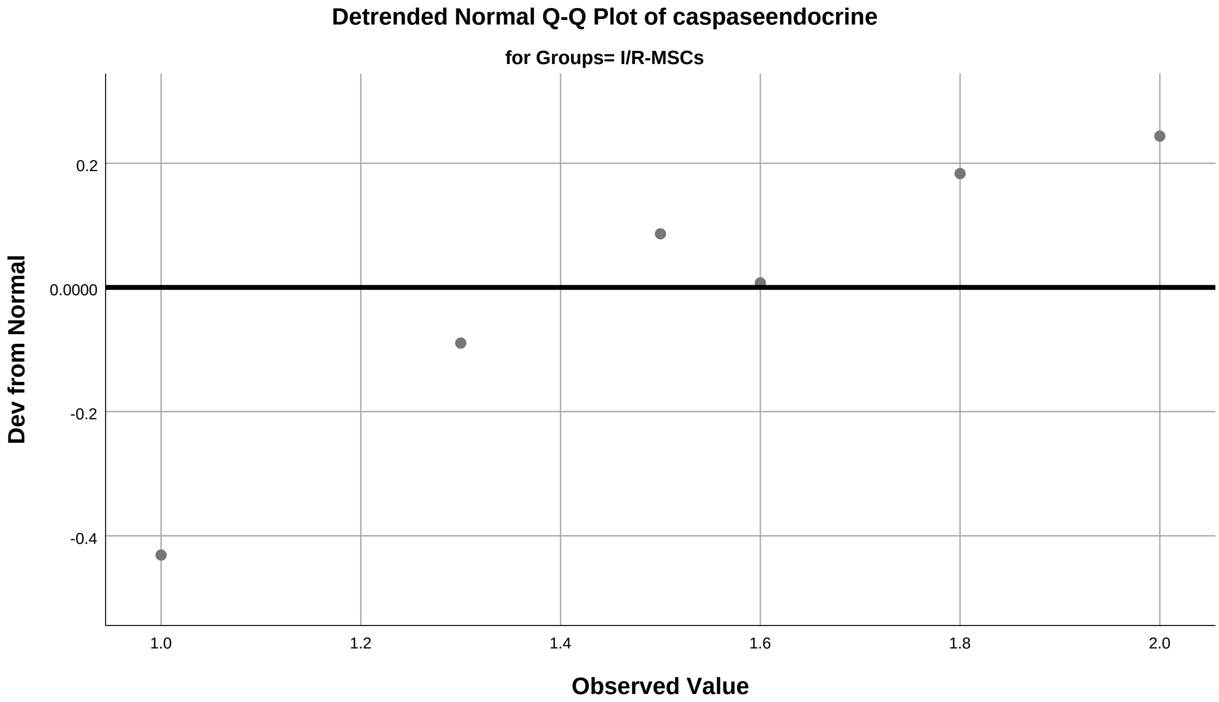
Detrended Normal Q-Q Plot of caspaseendocrine
for Groups= I/R-MSCs
Dev from Normal
0.2
0.0000
-0.2
-0.4
1.0
1.2
1.4
1.6
1.8
2.0
Observed Value

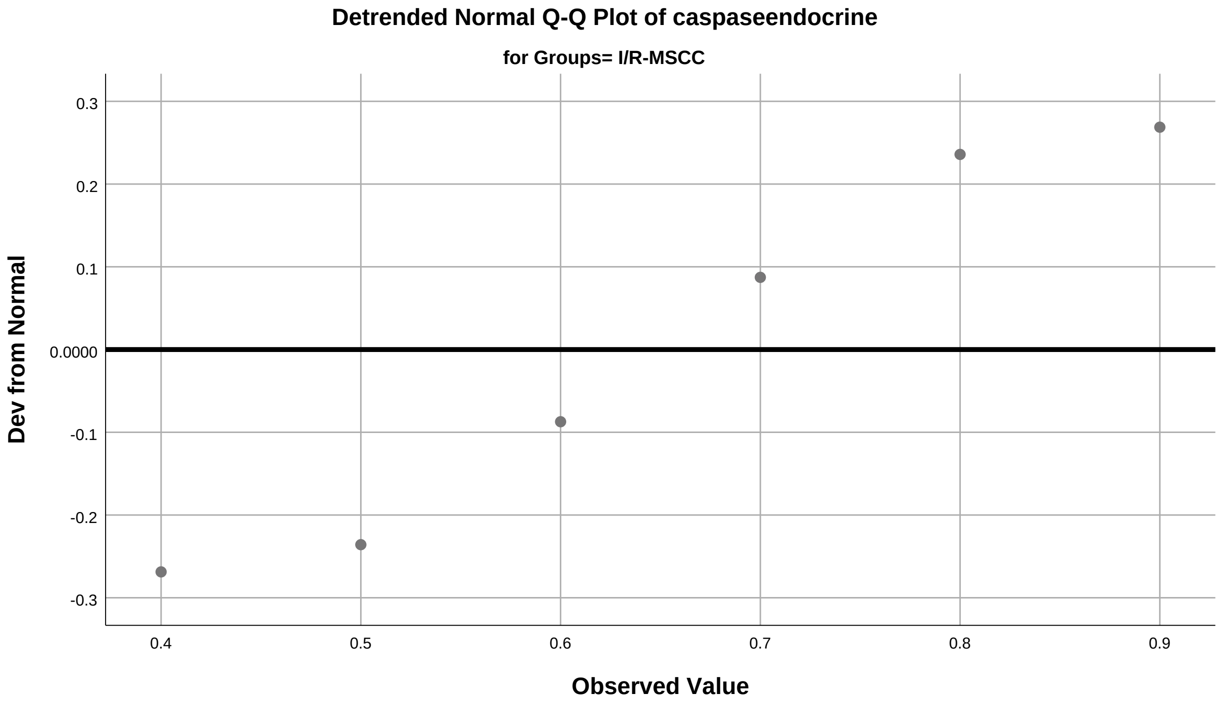

Detrended Normal Q-Q Plot of caspaseendocrine
for Groups= I/R-MSCC
Dev from Normal
0.3
0.2
0.1
0.0000
-0.1
-0.2
-0.3
0.4
0.5
0.6
0.7
0.8
0.9
Observed Value

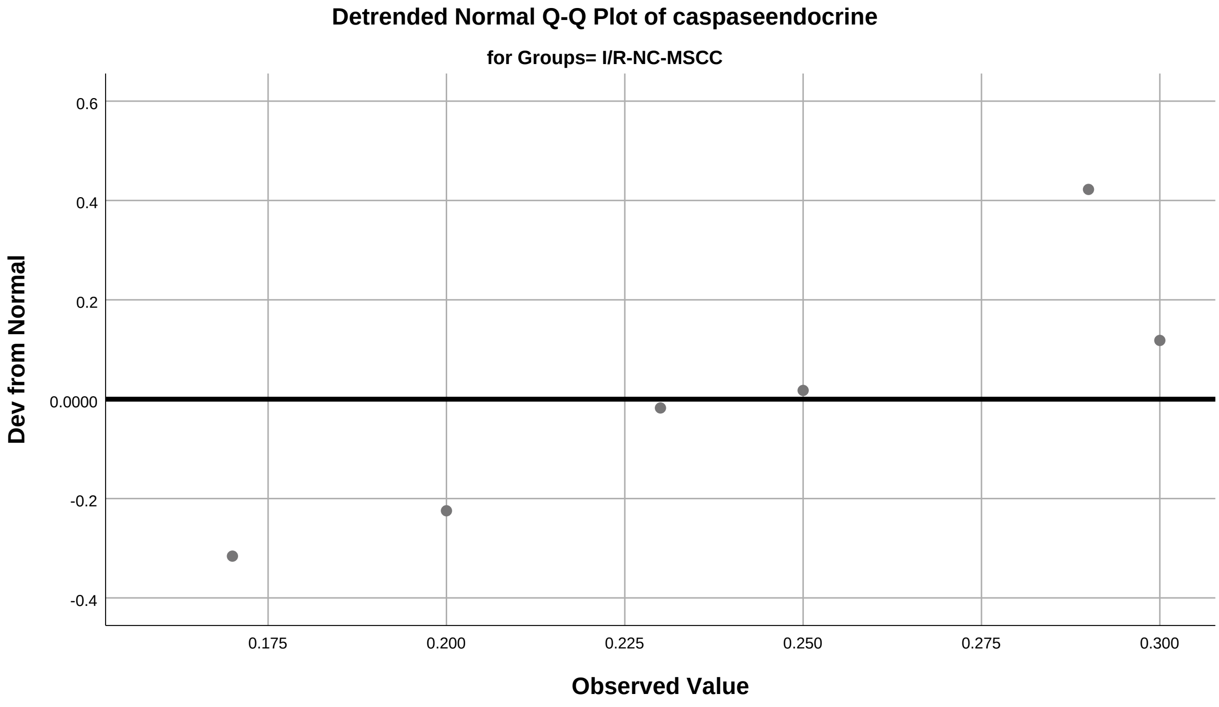

Detrended Normal Q-Q Plot of caspaseendocrine
for Groups= I/R-NC-MSCC
Dev from Normal
0.6
0.4
0.2
0.0000
-0.2
-0.4
0.175
0.200
0.225
0.250
0.275
0.300
Observed Value

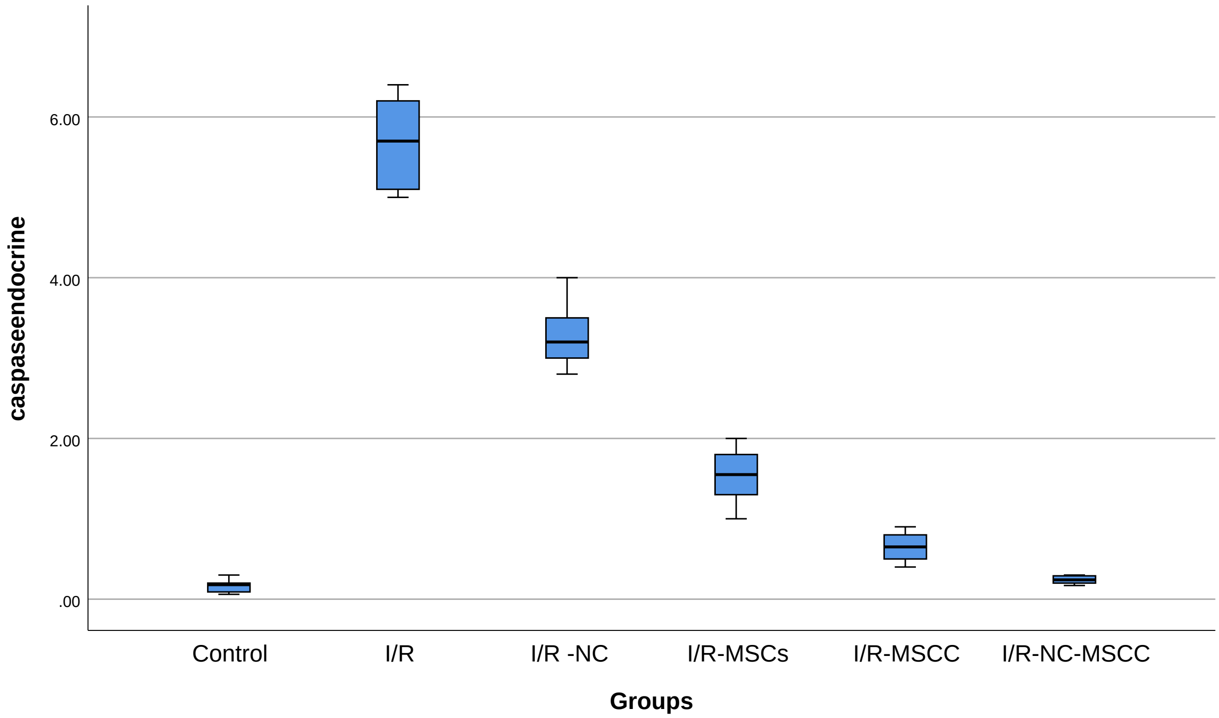

antiinsulinantibody

Normal Q-Q Plots

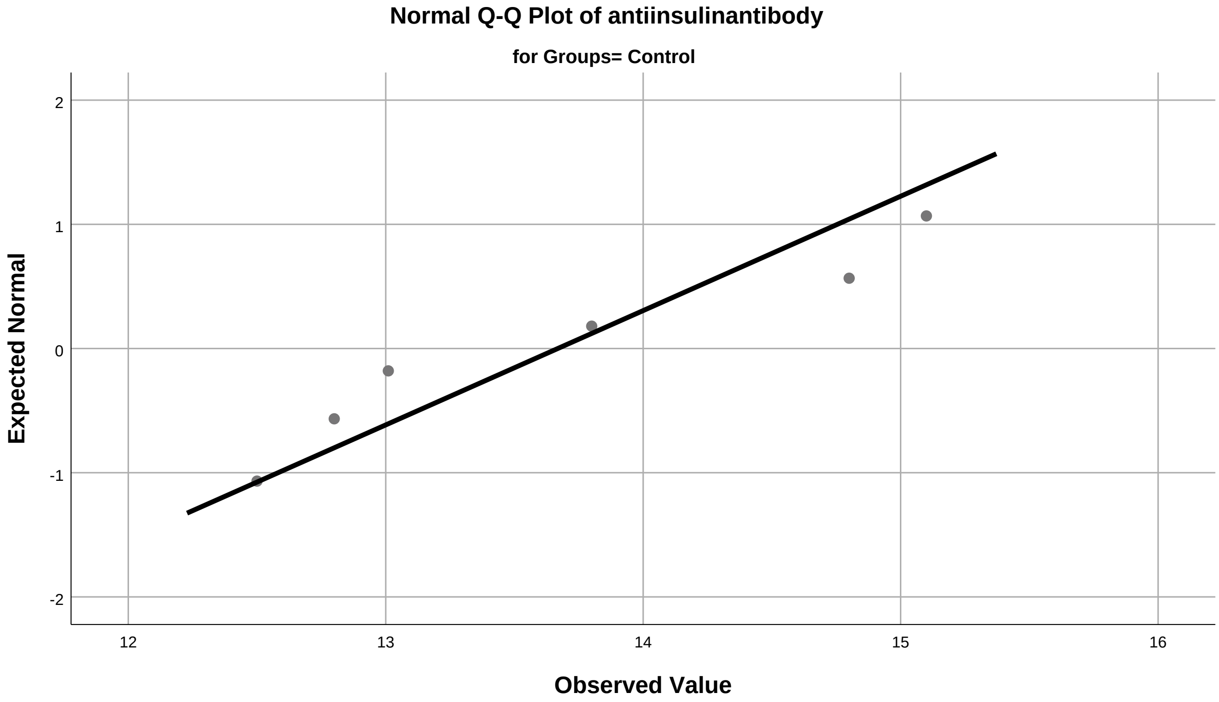

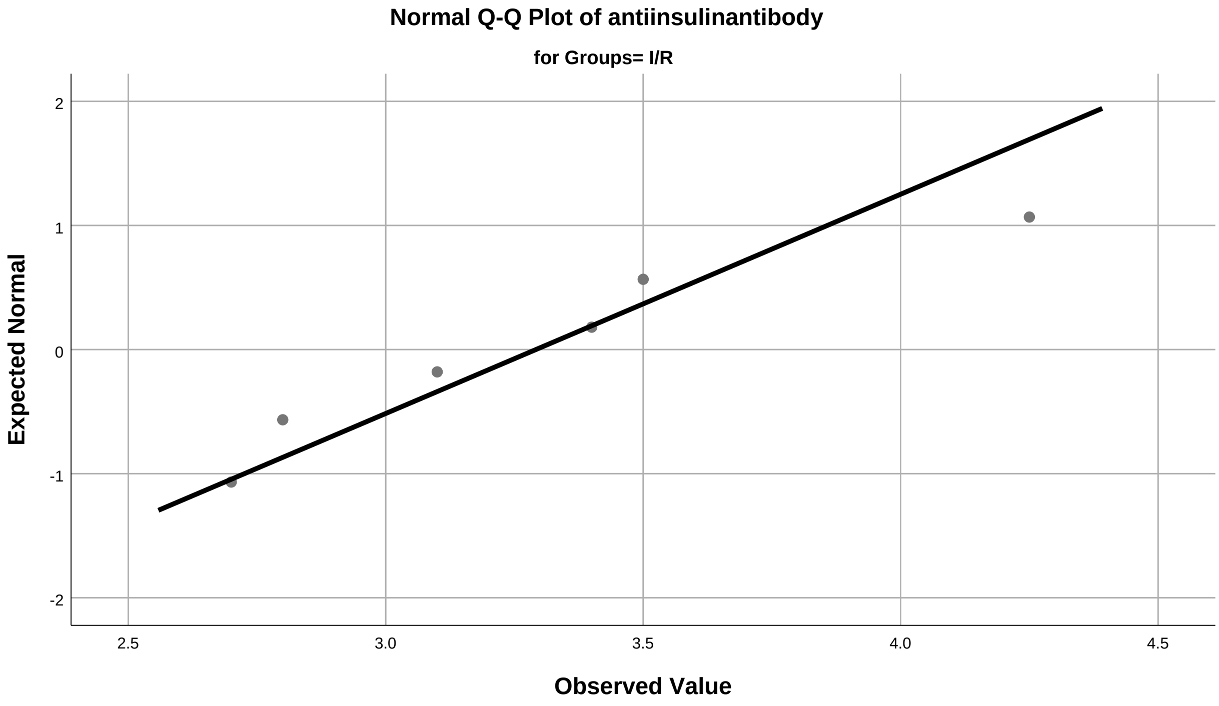
Normal Q-Q Plot of antiinsulinantibody
for Groups= I/R
Expected Normal
2
1
0
-1
-2
2.5
3.0
3.5
4.0
4.5
Observed Value

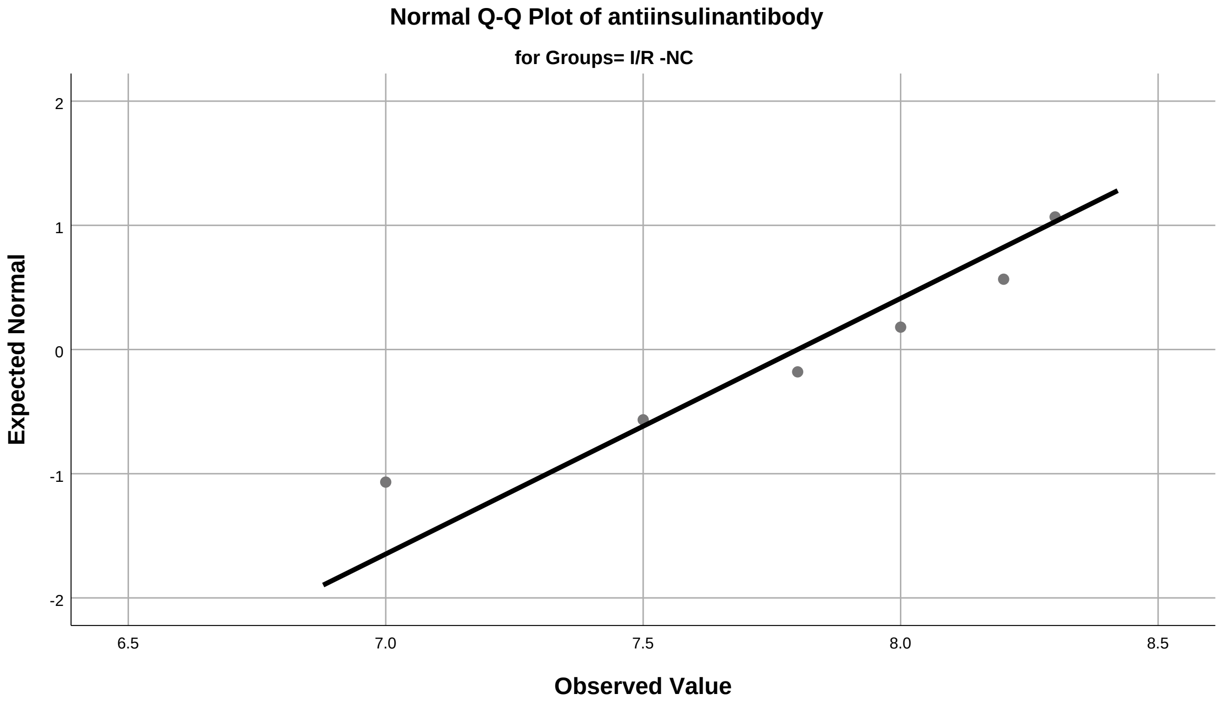
Normal Q-Q Plot of antiinsulinantibody
for Groups= I/R -NC
Expected Normal
2
1
0
-1
-2
6.5
7.0
7.5
8.0
8.5
Observed Value

Normal Q-Q Plot of antiinsulinantibody

for Groups= I/R-MSCs

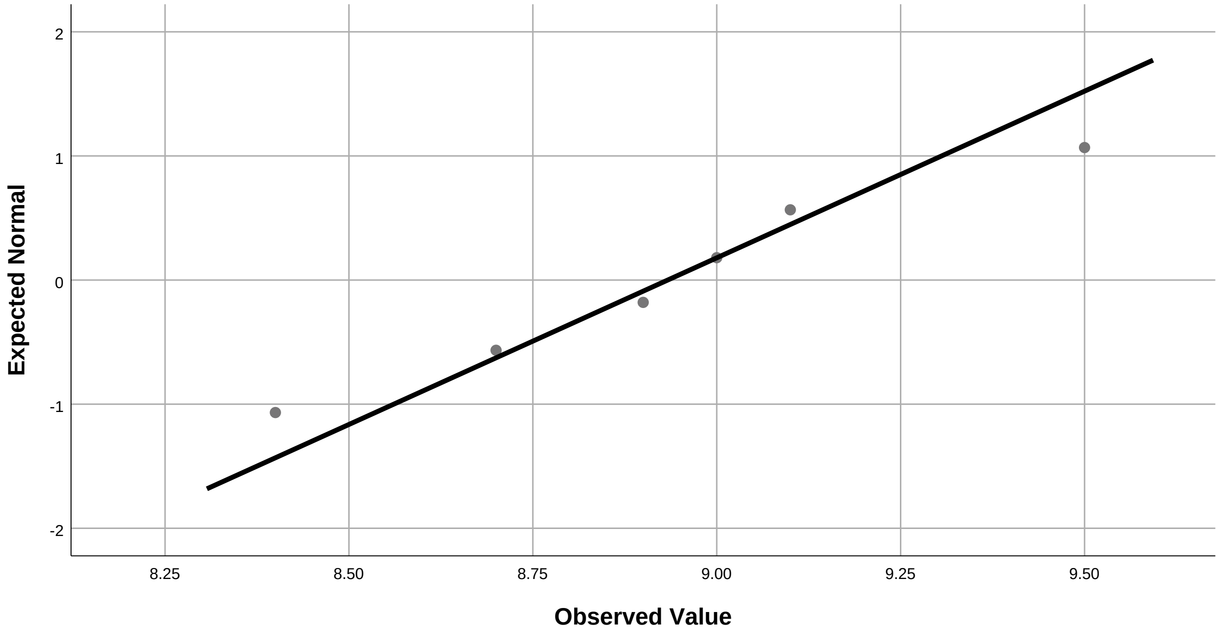

Normal Q-Q Plot of antiinsulinantibody

for Groups= I/R-MSCC

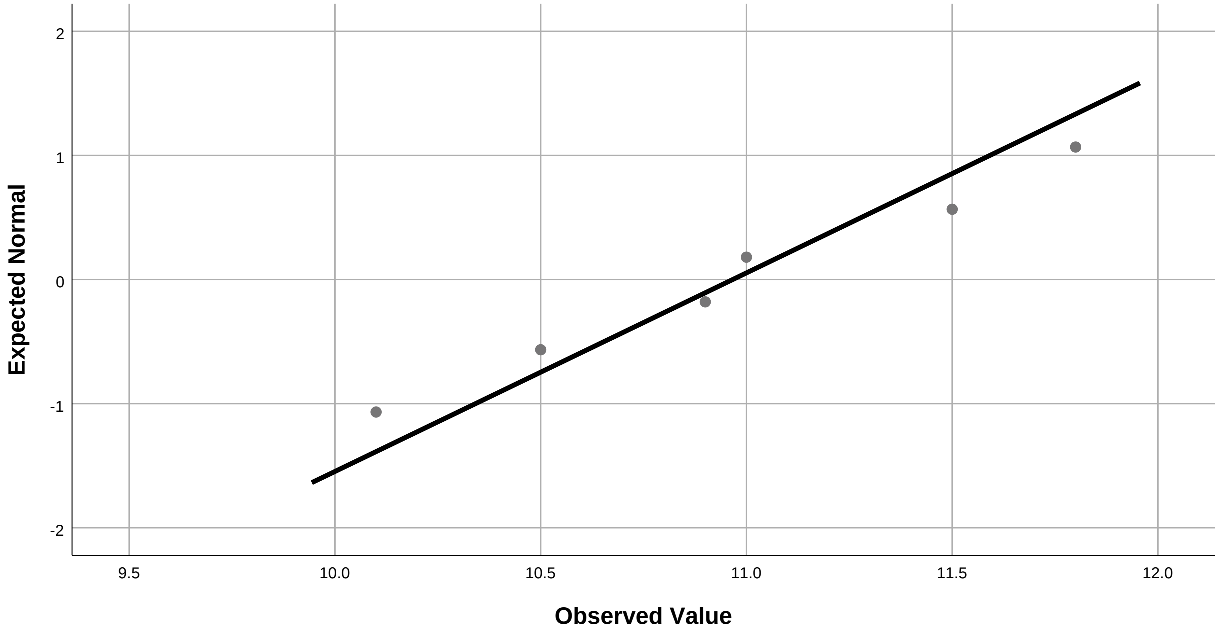

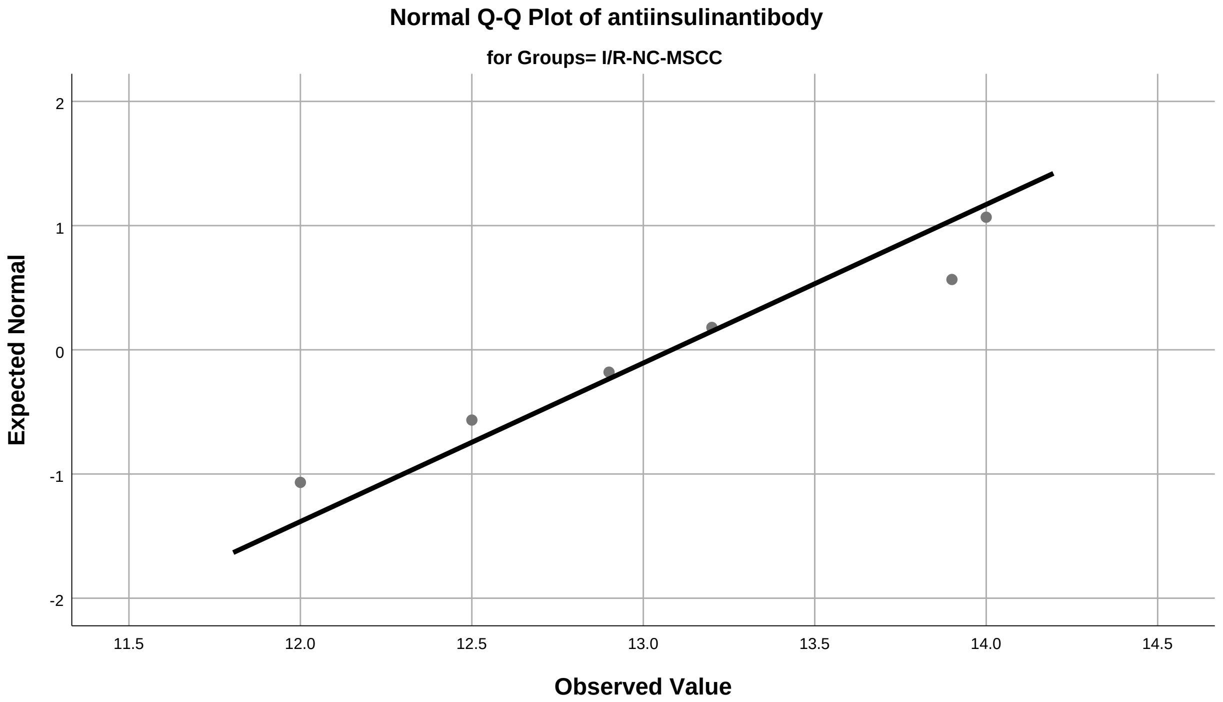

Detrended Normal Q-Q Plots

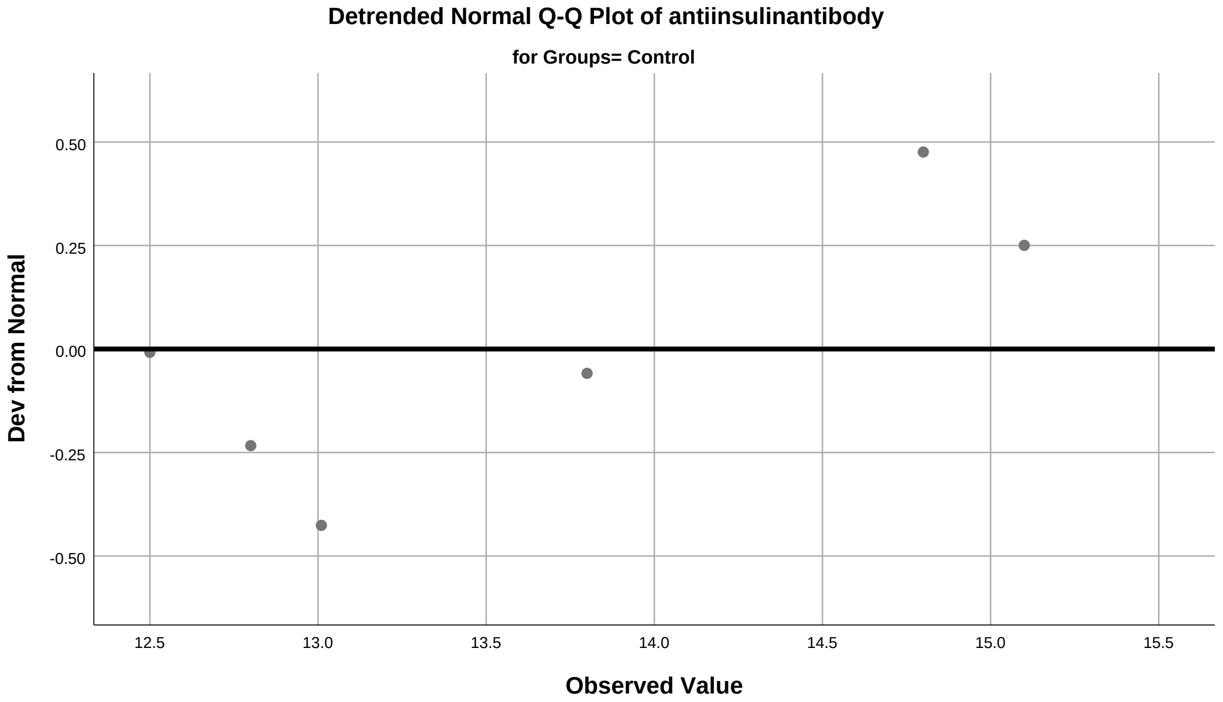

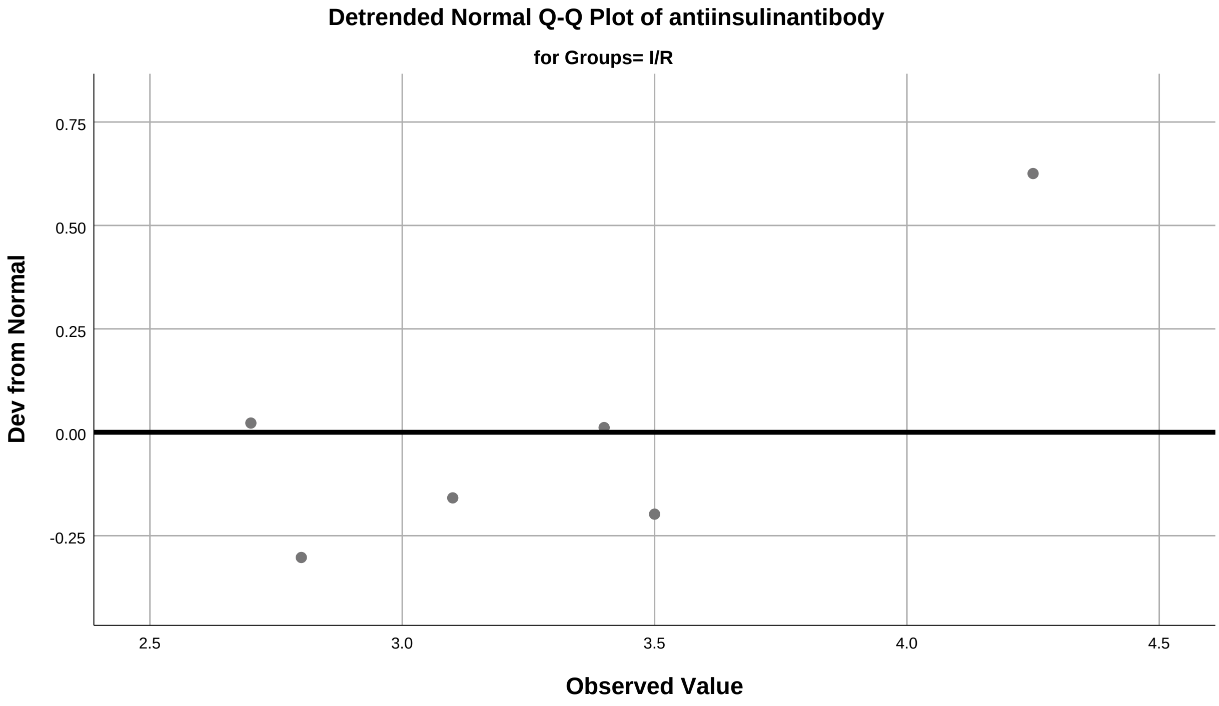
Detrended Normal Q-Q Plot of antiinsulinantibody
for Groups= I/R
Dev from Normal
0.75
0.50
0.25
0.00
-0.25
2.5
3.0
3.5
4.0
4.5
Observed Value

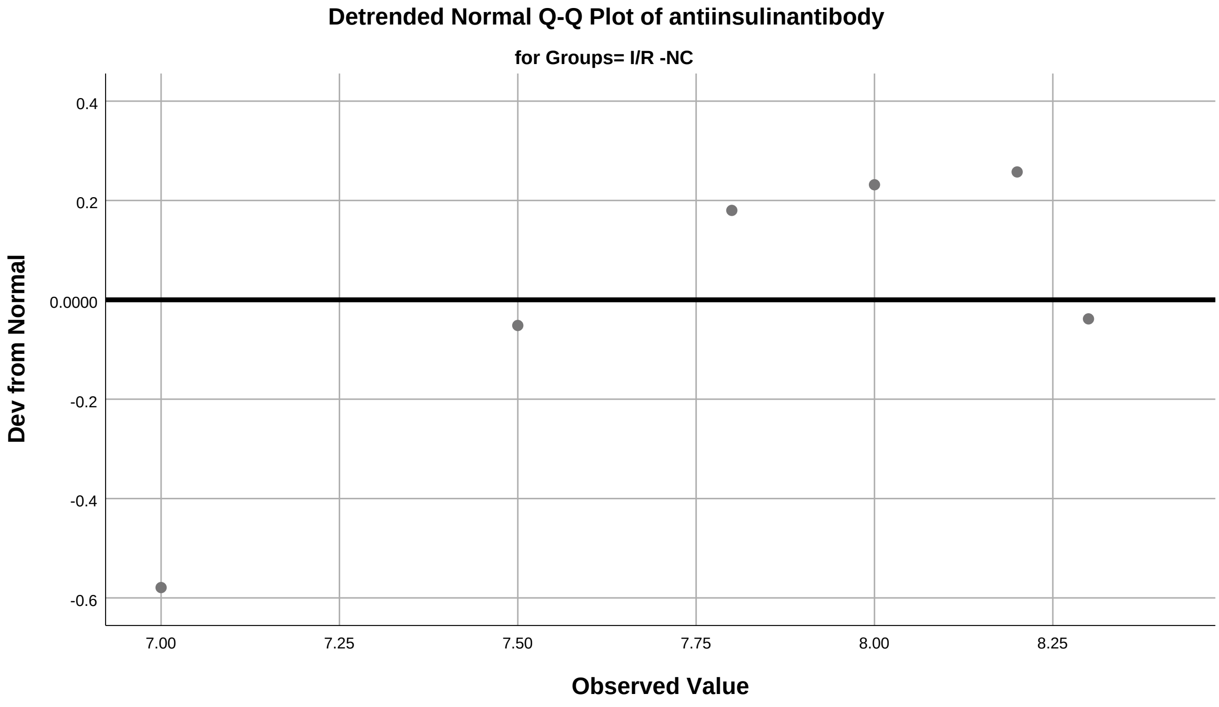
Detrended Normal Q-Q Plot of antiinsulinantibody
for Groups= I/R -NC
Dev from Normal
0.4
0.2
0.0000
-0.2
-0.4
-0.6
7.00
7.25
7.50
7.75
8.00
8.25
Observed Value

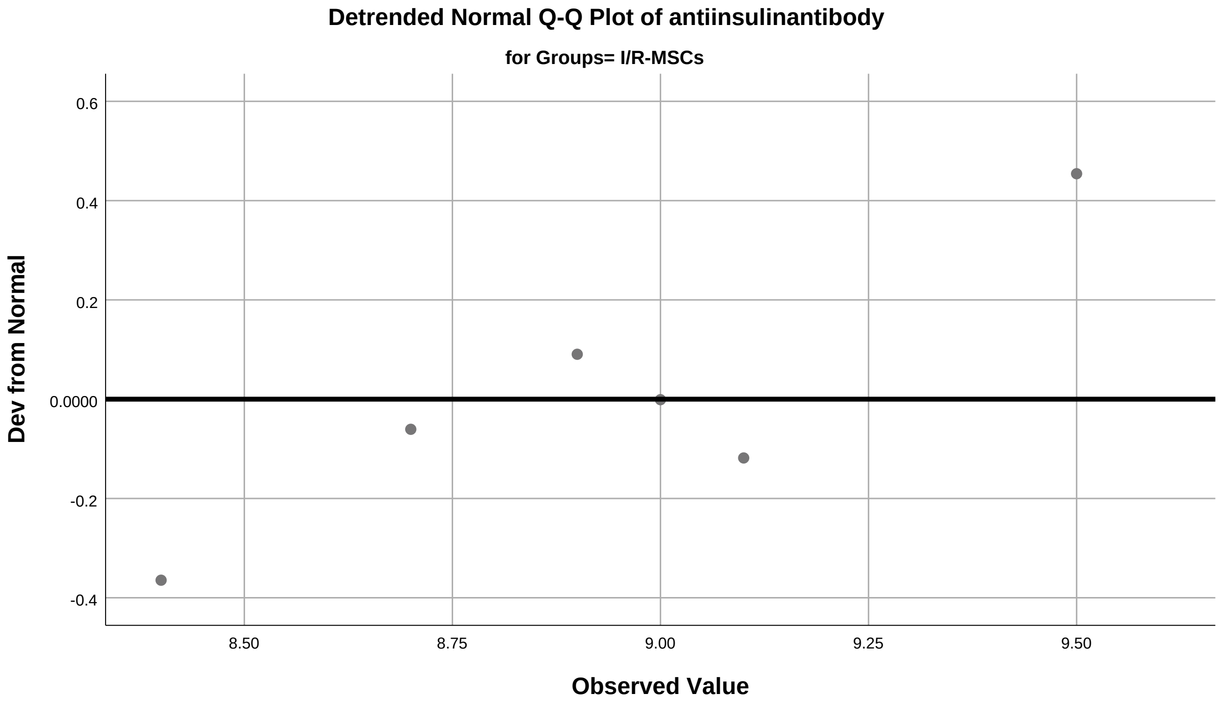

Detrended Normal Q-Q Plot of antiinsulinantibody
for Groups= I/R-MSCs
Dev from Normal
0.6
0.4
0.2
0.0000
-0.2
-0.4
8.50
8.75
9.00
9.25
9.50
Observed Value

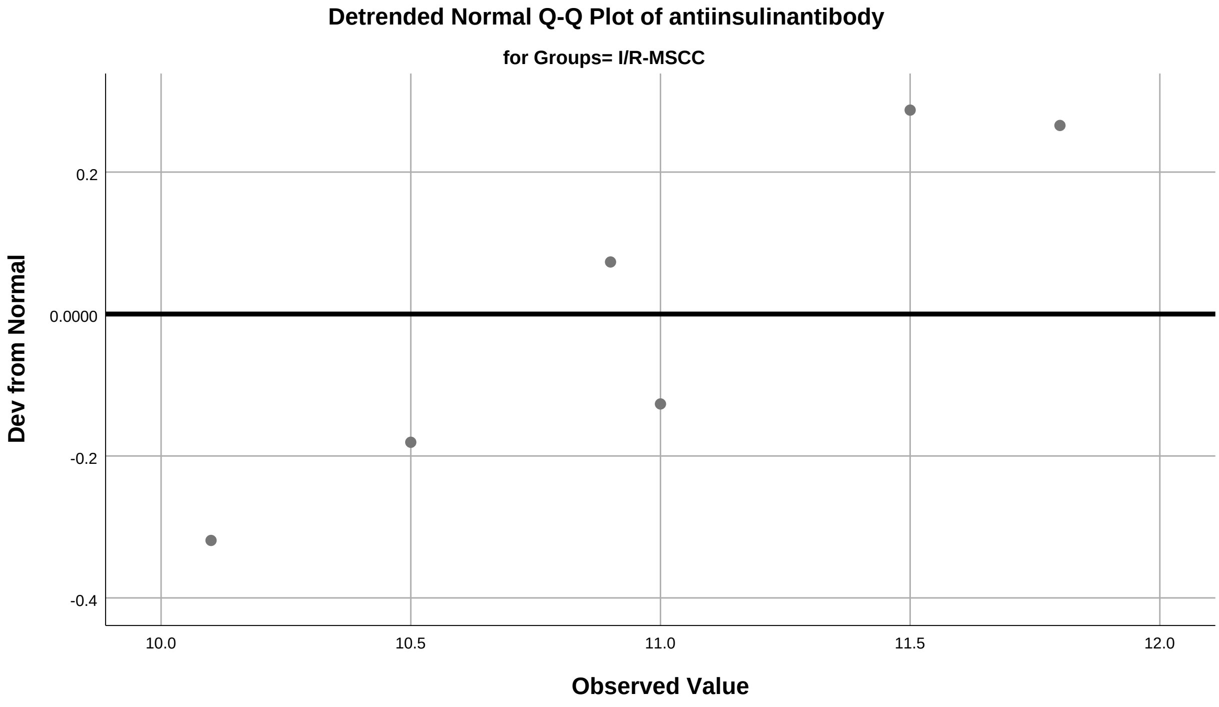

Detrended Normal Q-Q Plot of antiinsulinantibody
for Groups= I/R-MSCC
Dev from Normal
0.2
0.0000
-0.2
-0.4
10.0
10.5
11.0
11.5
12.0
Observed Value

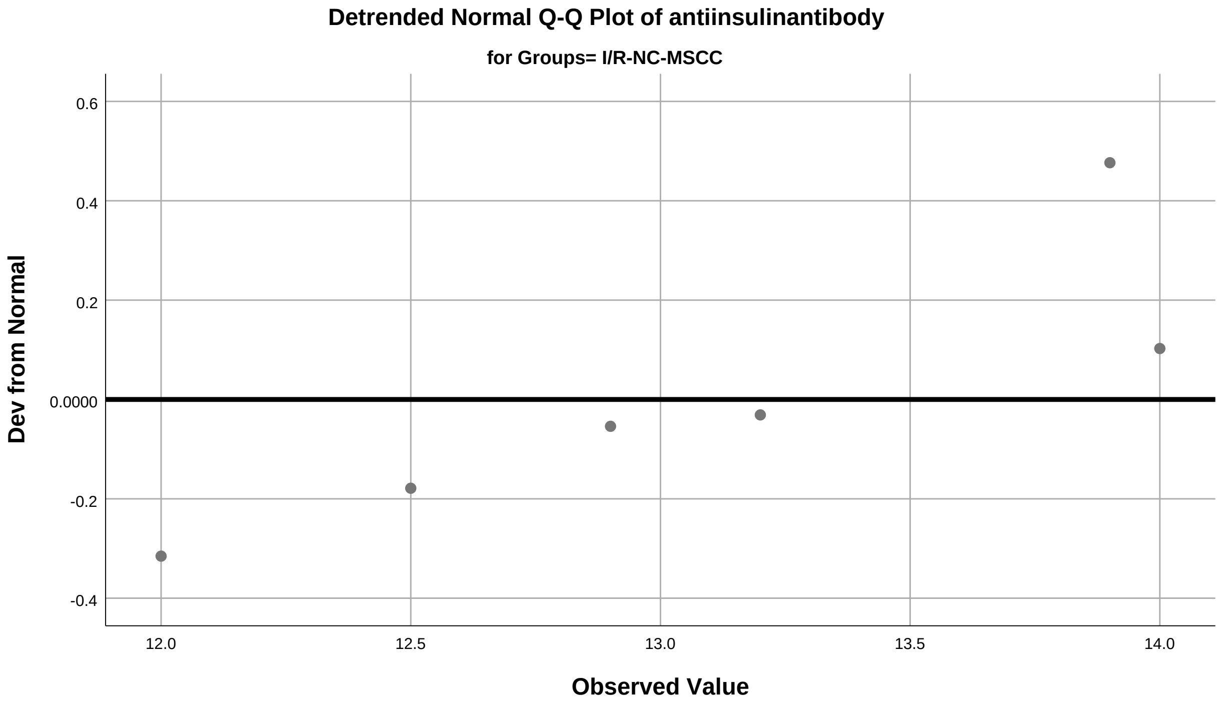

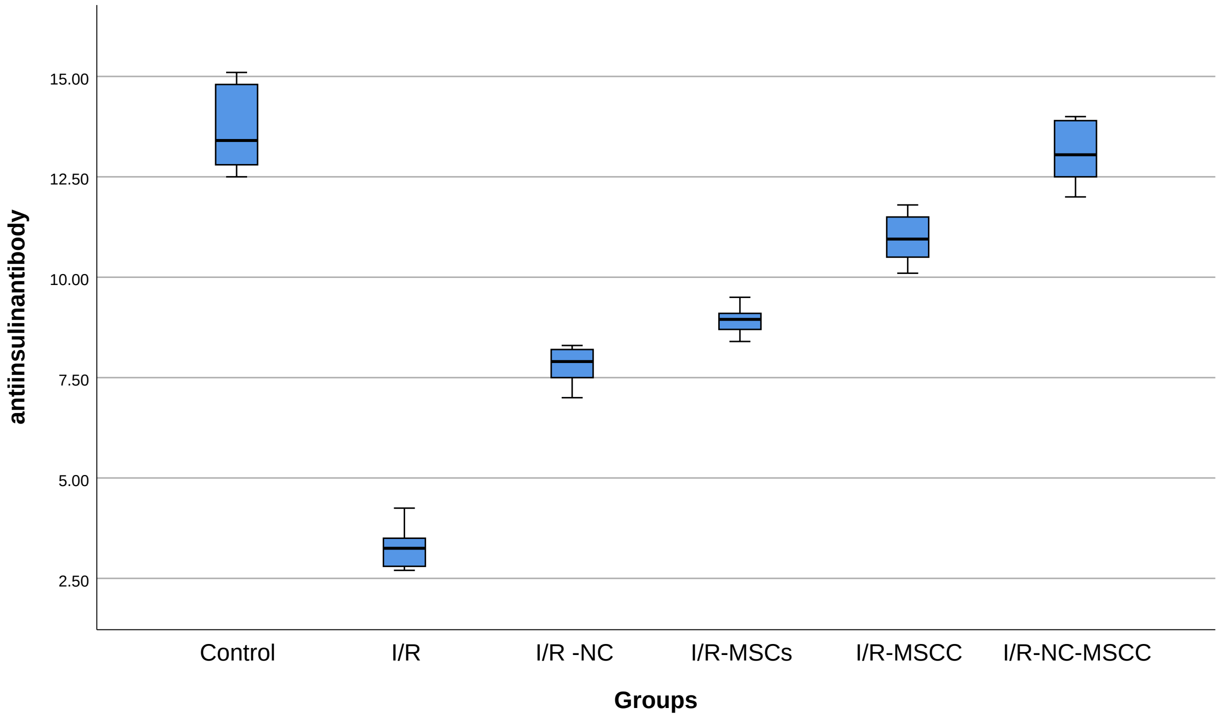

pancreasescore

Normal Q-Q Plots

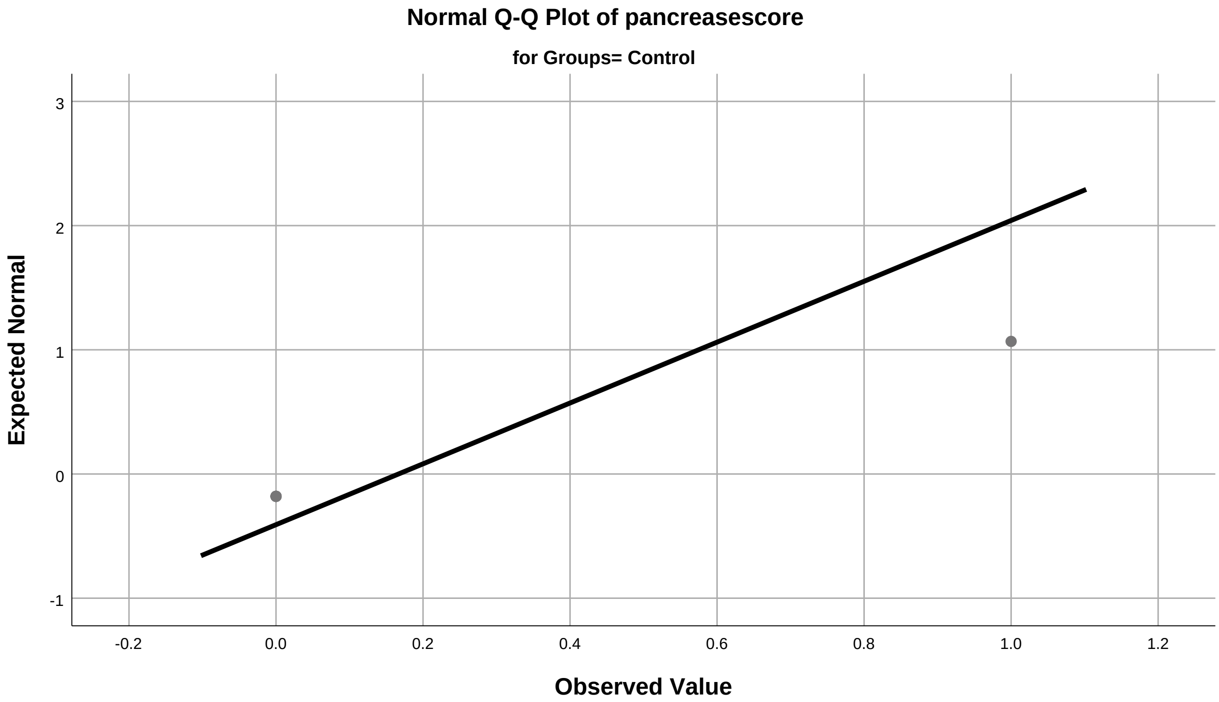
Normal Q-Q Plot of pancreasescore
for Groups= Control
Expected Normal
3
2
1
0
-1
-0.2
0.0
0.2
0.4
0.6
0.8
1.0
1.2
Observed Value

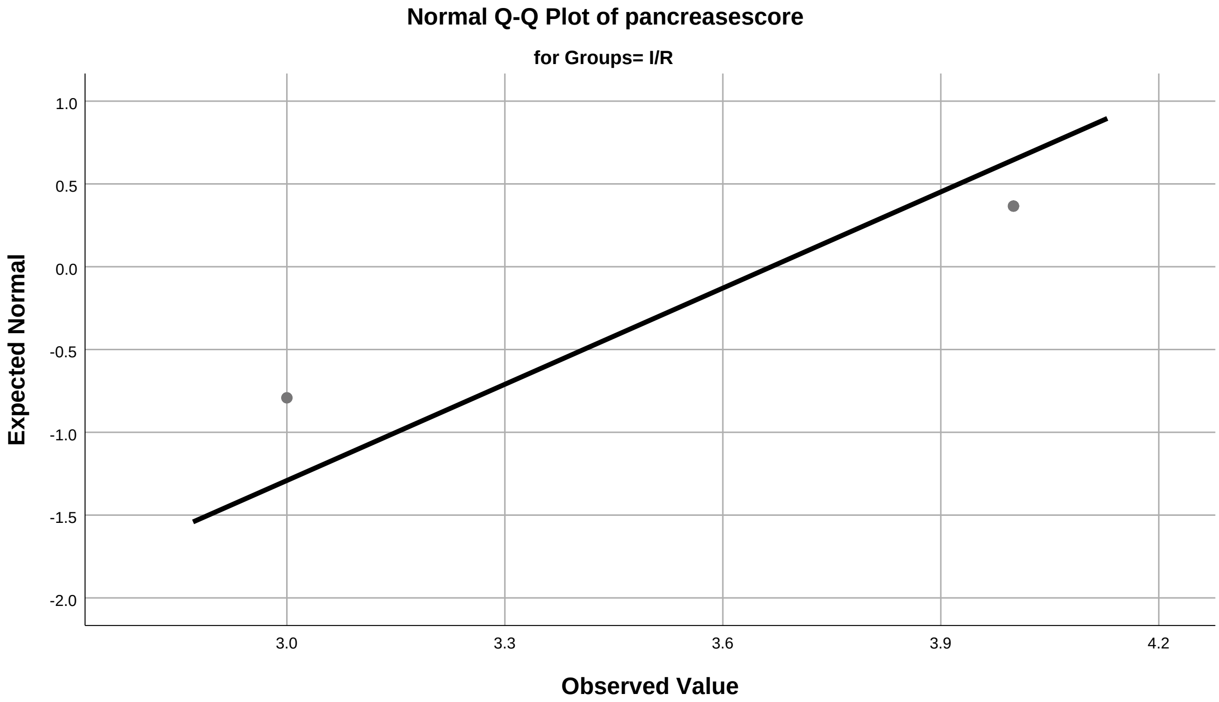
Normal Q-Q Plot of pancreasescore
for Groups= I/R
Expected Normal
1.0
0.5
0.0
-0.5
-1.0
-1.5
-2.0
3.0
3.3
3.6
3.9
4.2
Observed Value

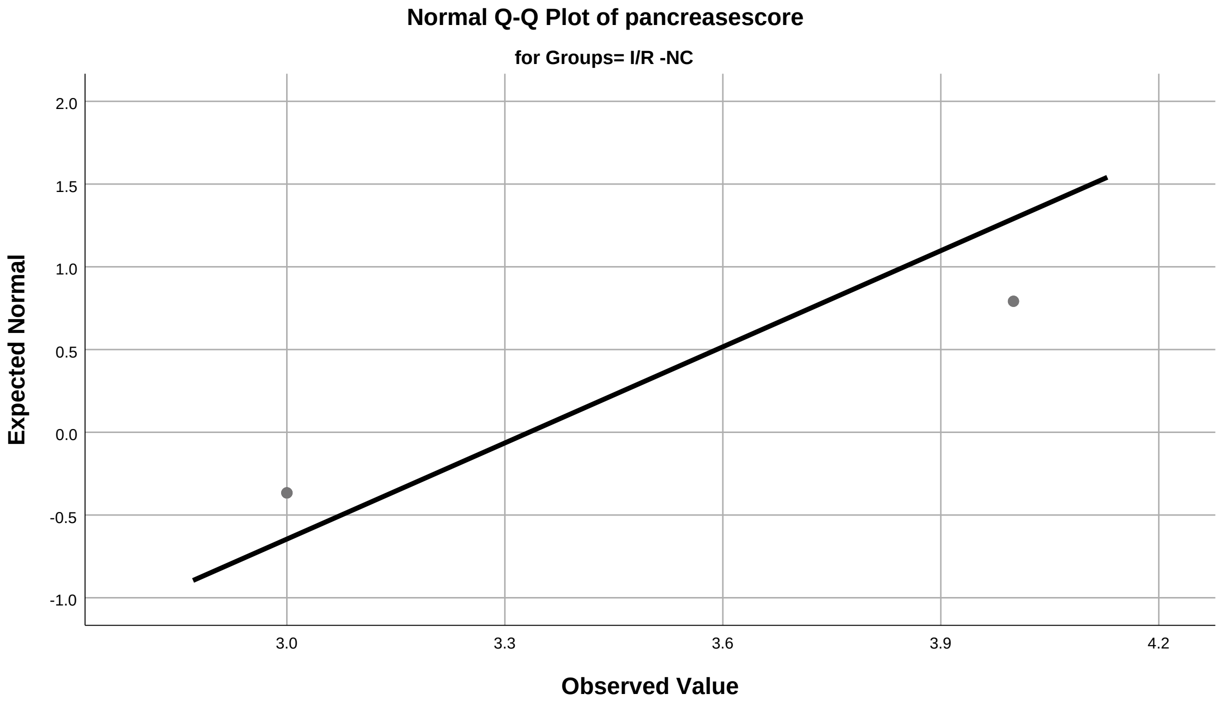
Normal Q-Q Plot of pancreasescore
for Groups= I/R -NC
Expected Normal
Observed Value
2.0
1.5
1.0
0.5
0.0
-0.5
-1.0
3.0
3.3
3.6
3.9
4.2

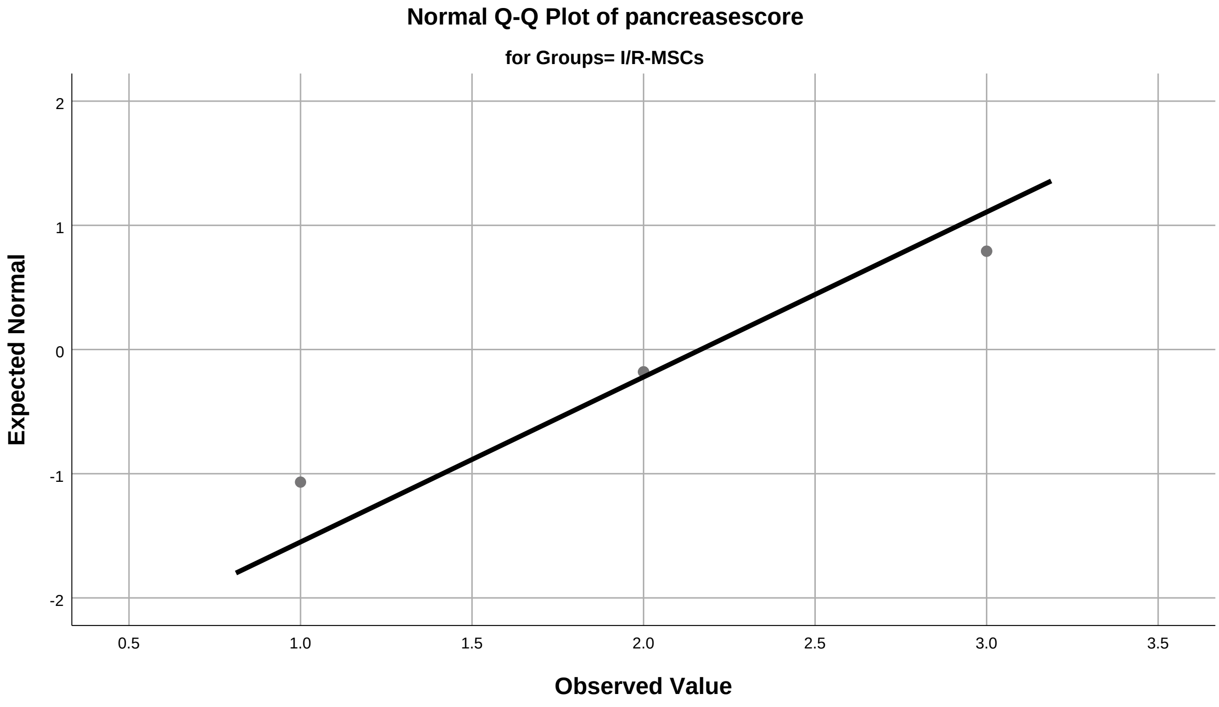
Normal Q-Q Plot of pancreasescore
for Groups= I/R-MSCs
Expected Normal
Observed Value
2
1
0
-1
-2
0.5
1.0
1.5
2.0
2.5
3.0
3.5

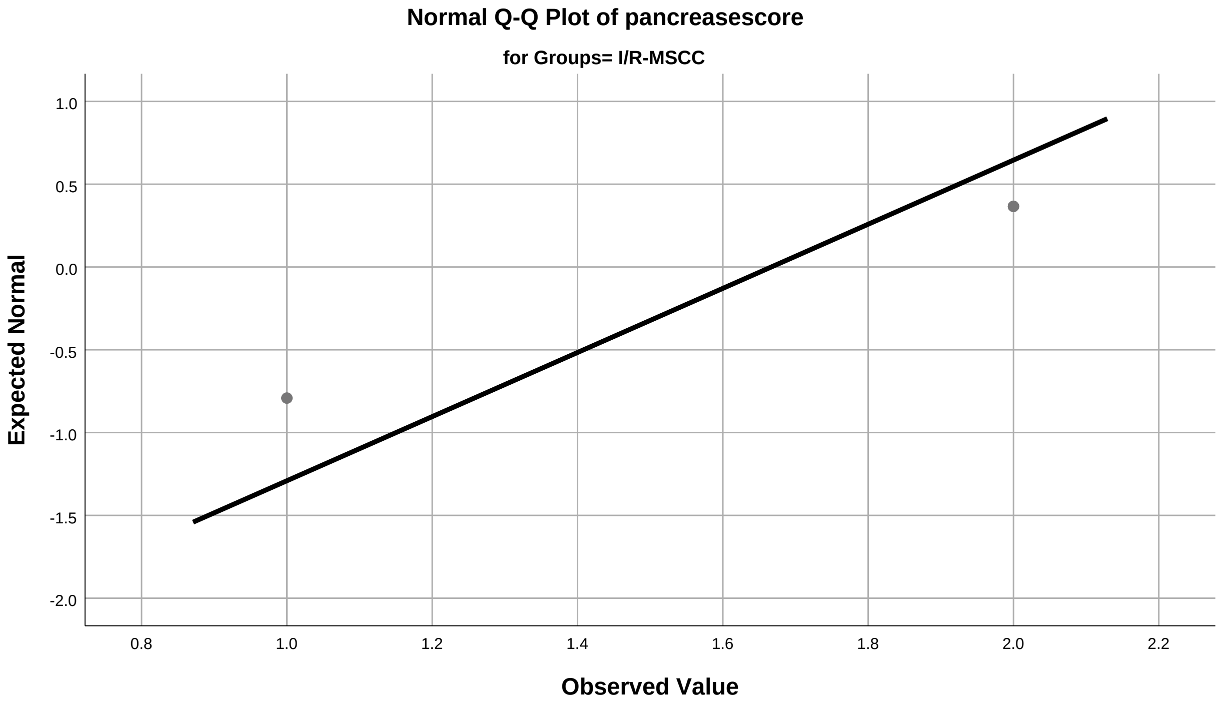

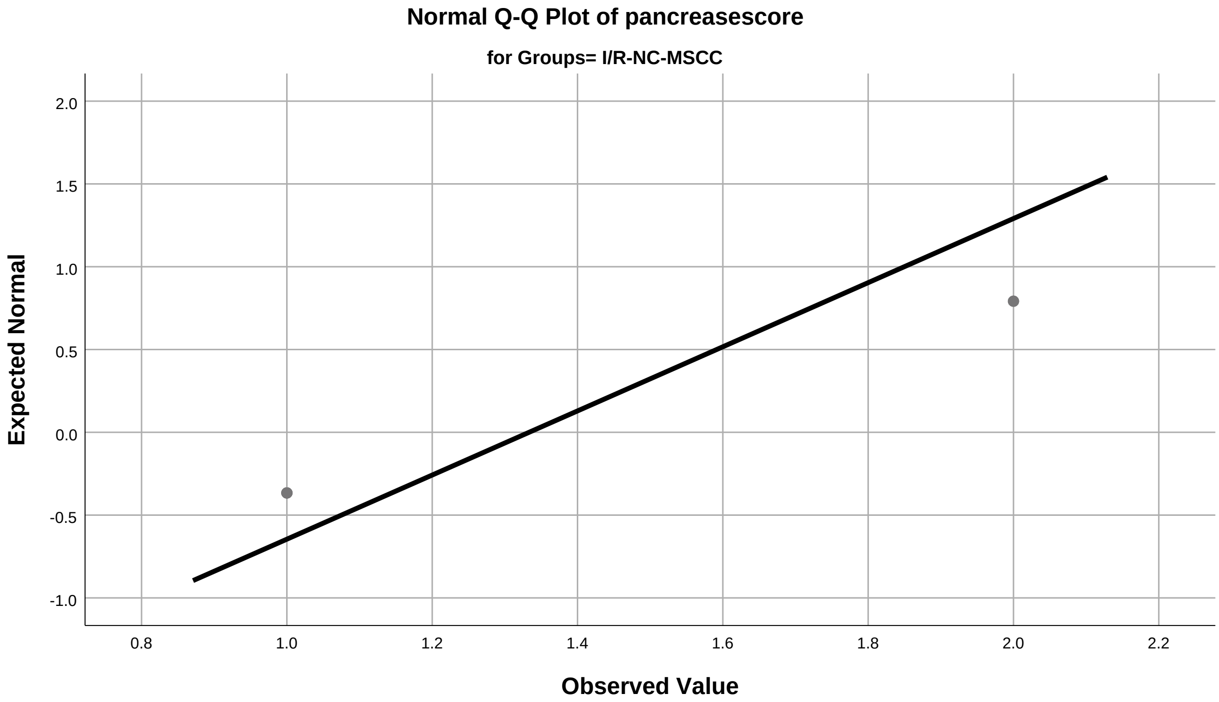

Detrended Normal Q-Q Plots

Detrended Normal Q-Q Plot of pancreasescore

for Groups= Control

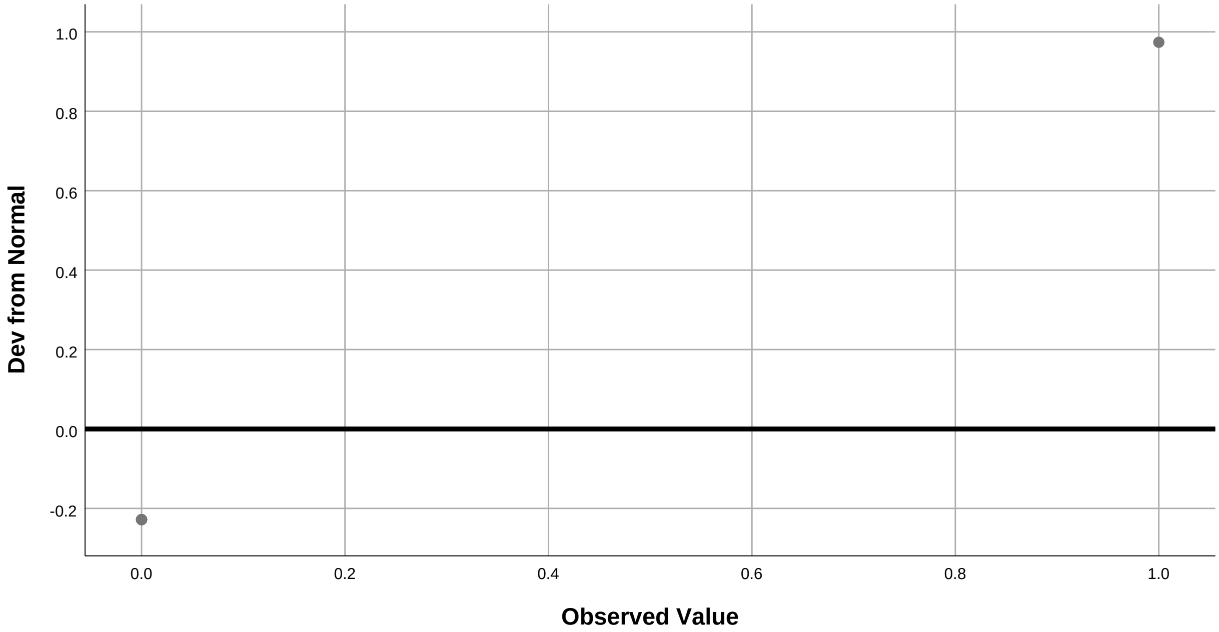

Detrended Normal Q-Q Plot of pancreasescore

for Groups= I/R

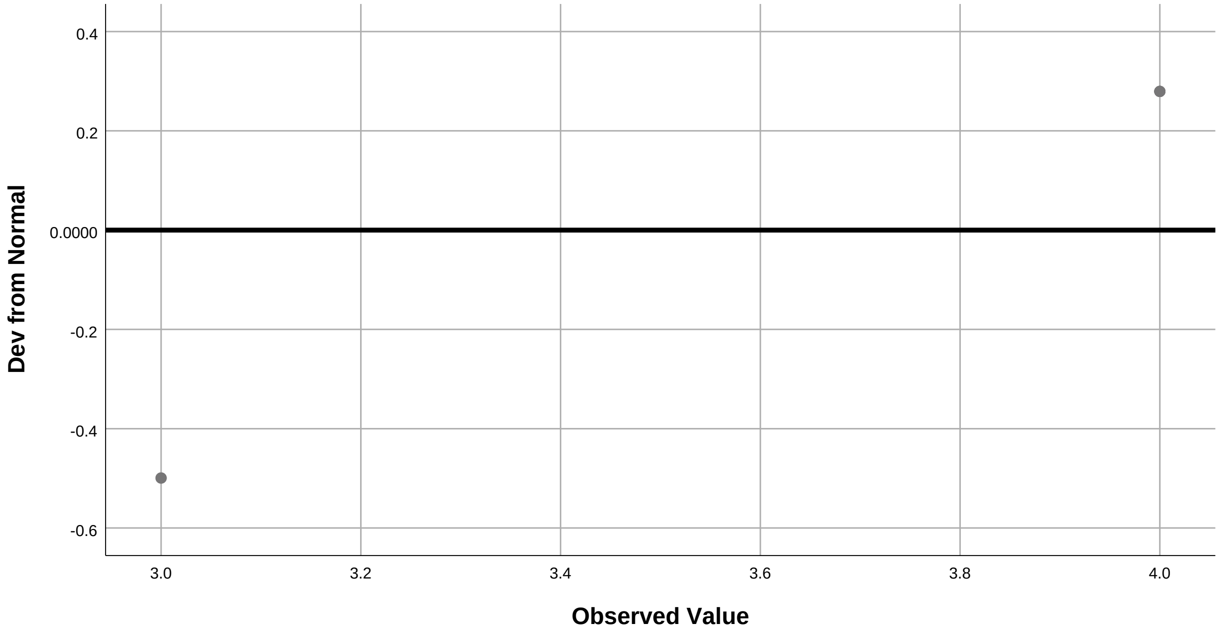

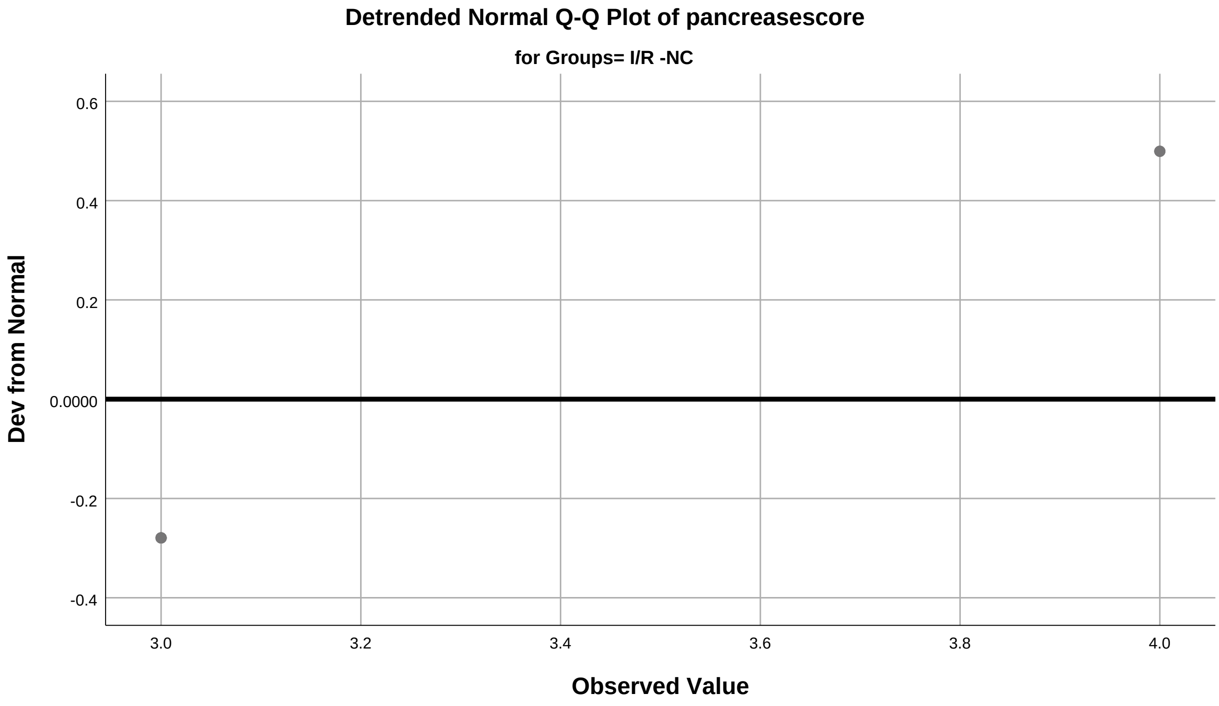
Detrended Normal Q-Q Plot of pancreasescore
for Groups= I/R -NC
Dev from Normal
0.6
0.4
0.2
0.0000
-0.2
-0.4
3.0
3.2
3.4
3.6
3.8
4.0
Observed Value

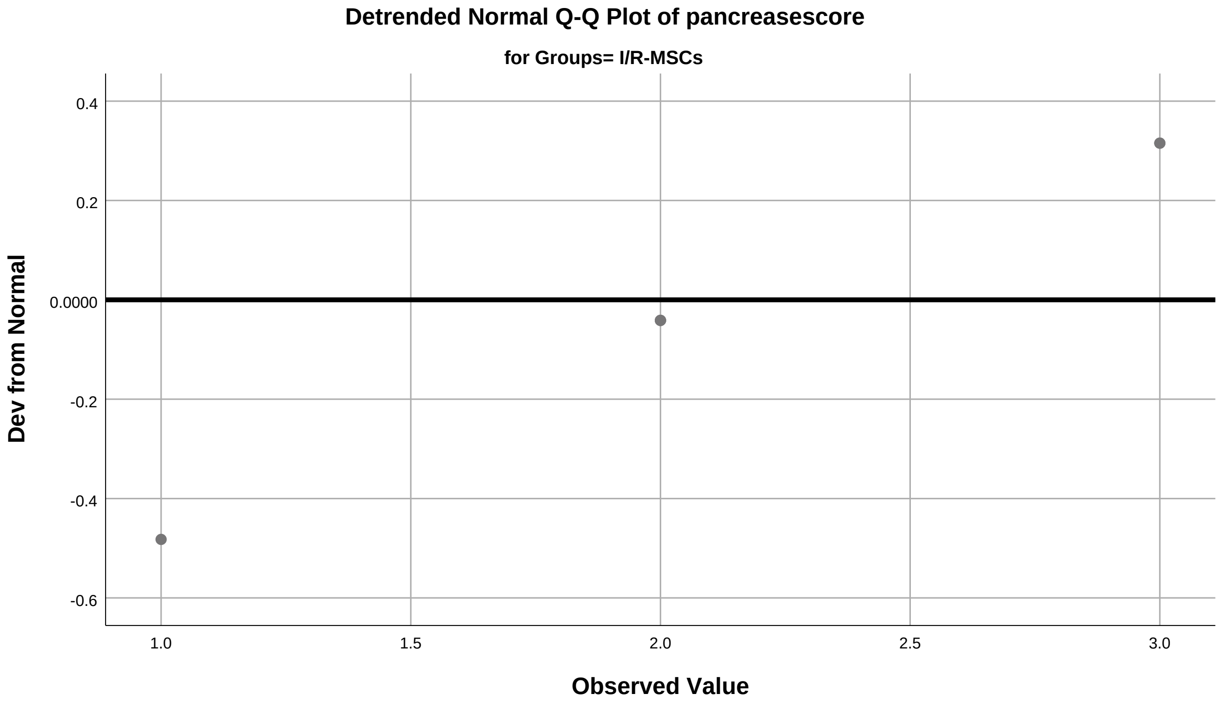
Detrended Normal Q-Q Plot of pancreasescore
for Groups= I/R-MSCs
Dev from Normal
0.4
0.2
0.0000
-0.2
-0.4
-0.6
1.0
1.5
2.0
2.5
3.0
Observed Value

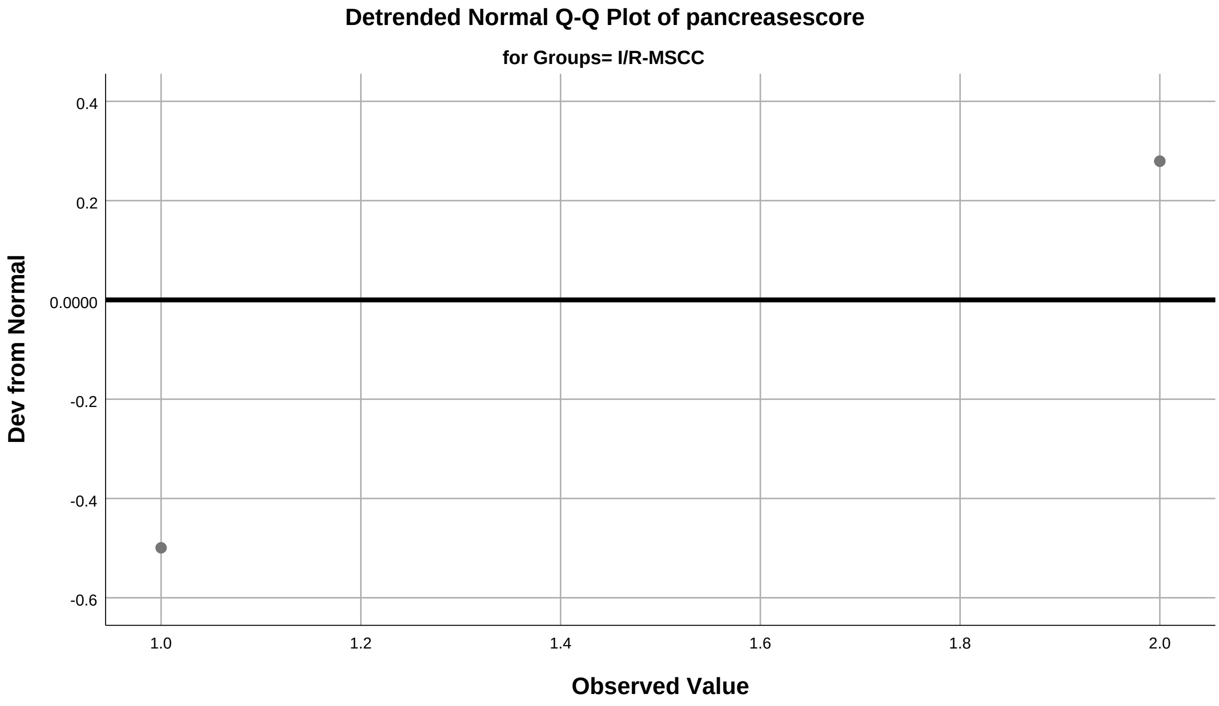
Detrended Normal Q-Q Plot of pancreasescore
for Groups= I/R-MSCC
Dev from Normal
0.4
0.2
0.0000
-0.2
-0.4
-0.6
1.0
1.2
1.4
1.6
1.8
2.0
Observed Value

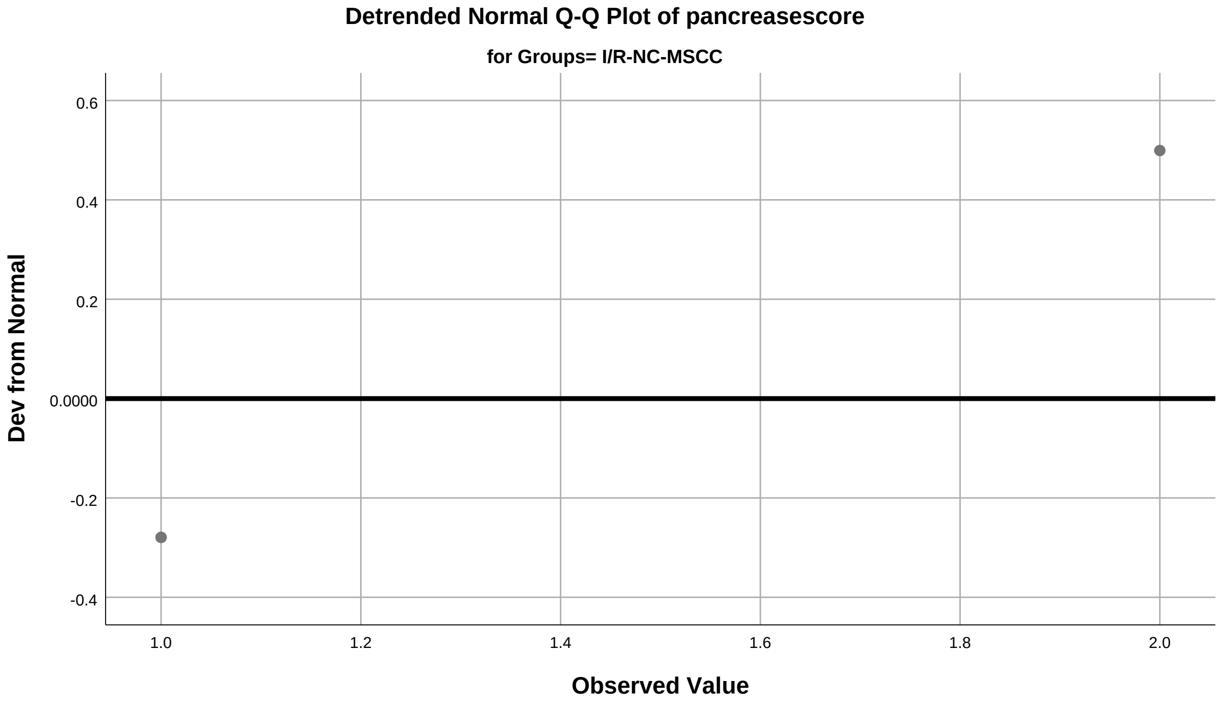
Detrended Normal Q-Q Plot of pancreasescore
for Groups= I/R-NC-MSCC
Dev from Normal
0.6
0.4
0.2
0.0000
-0.2
-0.4
1.0
1.2
1.4
1.6
1.8
2.0
Observed Value

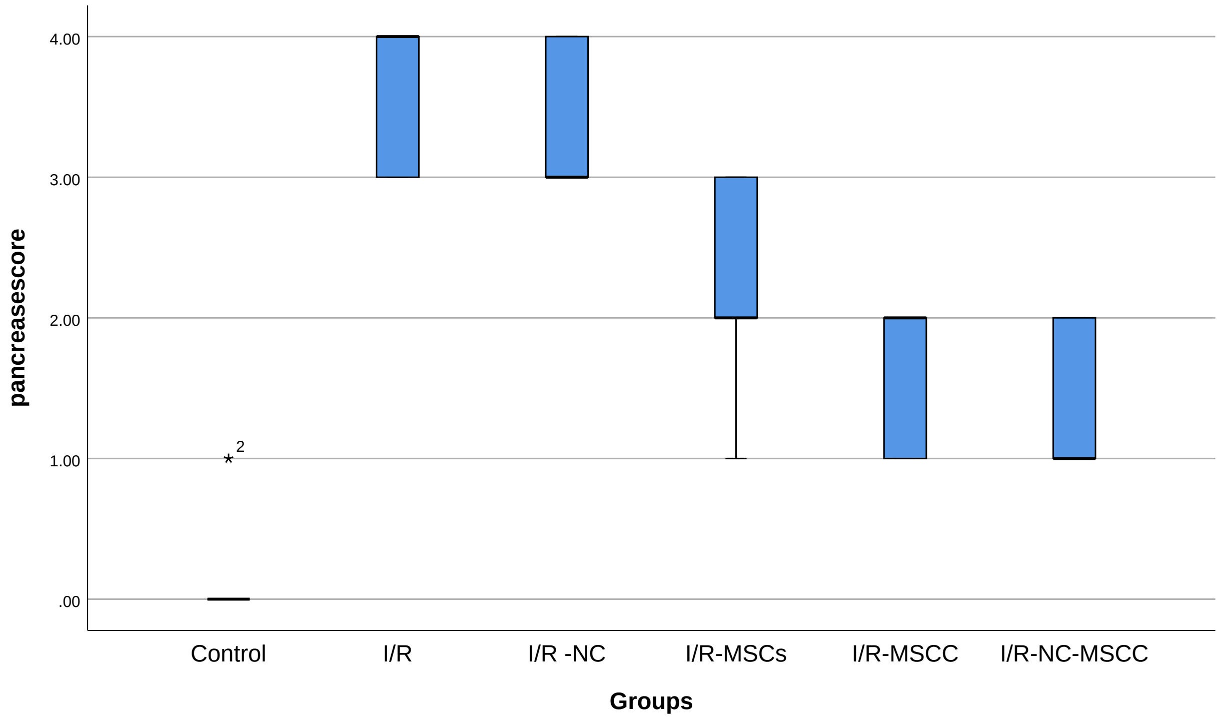


```
 DATASET ACTIVATE DataSet1.

SAVE OUTFILE='C:\Users\Dr_Abeer\Desktop\statistics\dr asmaa shams paper\normal
ity.sav'
  /COMPRESSED.
```
