# Supplementary material for: Combined Systemic Intake of K-ATP Opener (Nicorandil) and Mesenchymal Stem Cells Preconditioned With Nicorandil Alleviates Pancreatic Insufficiency in a Model of Bilateral Renal Ischemia/Reperfusion Injury
Source: Front Physiol. 2022 Jun 23;13:934597. doi: 10.3389/fphys.2022.934597 (PMC9260271; doi:10.3389/fphys.2022.934597)
Supplement: Supplementary file 2 [file DataSheet1.PDF]

```
EXAMINE VARIABLES=proliferation proliferation2 bax1 bax12 bcl bcll pi3k1 pi3k1
2 akt1 akt12 mtor1
    mtor12
  /PLOT BOXPLOT NPPLOT
  /COMPARE GROUPS
  /STATISTICS DESCRIPTIVES
  /CINTERVAL 95
  /MISSING LISTWISE
  /NOTOTAL.
```

Explore

Notes

Output Created		12-MAR-2022 16:09:28
Comments		
Input	Data	C:\Users\Dr_Abeer\Desktop\statistics\dr asmaa shams paper\normality.sav
	Active Dataset	DataSet1
	Filter	<none>
	Weight	<none>
	Split File	<none>
	N of Rows in Working Data File	36
Missing Value Handling	Definition of Missing	User-defined missing values for dependent variables are treated as missing.
	Cases Used	Statistics are based on cases with no missing values for any dependent variable or factor used.

Notes

Syntax		EXAMINE VARIABLES=proliferation proliferation2 bax1 bax12 bcl bcll pi3k1 pi3k12 akt1 akt12 mtor1<br>mtor12<br>/PLOT BOXPLOT NPPLOT<br>/COMPARE GROUPS<br>/STATISTICS DESCRIPTIVES<br>/CINTERVAL 95<br>/MISSING LISTWISE<br>/NOTOTAL.
Resources	Processor Time	00:00:04.13
	Elapsed Time	00:00:04.14

Case Processing Summary

	Cases					
	Valid		Missing		Total	
	N	Percent	N	Percent	N	Percent
proliferation	6	16.7%	30	83.3%	36	100.0%
proliferation2	6	16.7%	30	83.3%	36	100.0%
bax1	6	16.7%	30	83.3%	36	100.0%
bax12	6	16.7%	30	83.3%	36	100.0%
bcl	6	16.7%	30	83.3%	36	100.0%
bcll	6	16.7%	30	83.3%	36	100.0%
pi3k1	6	16.7%	30	83.3%	36	100.0%
pi3k12	6	16.7%	30	83.3%	36	100.0%
akt1	6	16.7%	30	83.3%	36	100.0%
akt12	6	16.7%	30	83.3%	36	100.0%
mtor1	6	16.7%	30	83.3%	36	100.0%
mtor12	6	16.7%	30	83.3%	36	100.0%

Descriptives

			Statistic	Std. Error
proliferation	Mean		97.2000	1.13461
	95% Confidence Interval for Mean	Lower Bound	94.2834	
		Upper Bound	100.1166	
	5% Trimmed Mean		97.0778	
	Median		96.6000	
	Variance		7.724	
	Std. Deviation		2.77921	
	Minimum		94.50	
	Maximum		102.10	
	Range		7.60	
	Interquartile Range		4.45	
	Skewness		1.230	.845
	Kurtosis		1.448	1.741
proliferation2	Mean		140.1667	4.37010
	95% Confidence Interval for Mean	Lower Bound	128.9330	
		Upper Bound	151.4004	
	5% Trimmed Mean		139.9407	
	Median		136.3500	
	Variance		114.587	
	Std. Deviation		10.70452	
	Minimum		129.40	
	Maximum		155.00	
	Range		25.60	
	Interquartile Range		20.43	
	Skewness		.623	.845
	Kurtosis		-1.793	1.741
bax1	Mean		1.0250	.00764
	95% Confidence Interval for Mean	Lower Bound	1.0054	
		Upper Bound	1.0446	
	5% Trimmed Mean		1.0250	
	Median		1.0250	
	Variance		.000	
	Std. Deviation		.01871	
	Minimum		1.00	
	Maximum		1.05	

Descriptives

			Statistic	Std. Error
	Range		.05	
	Interquartile Range		.03	
	Skewness		.000	.845
	Kurtosis		-1.200	1.741
bax12	Mean		.7317	.05653
	95% Confidence Interval for Mean	Lower Bound	.5863	
		Upper Bound	.8770	
	5% Trimmed Mean		.7335	
	Median		.7350	
	Variance		.019	
	Std. Deviation		.13848	
	Minimum		.54	
	Maximum		.89	
	Range		.35	
	Interquartile Range		.28	
	Skewness		-.202	.845
	Kurtosis		-1.486	1.741
bcl	Mean		1.0100	.00516
	95% Confidence Interval for Mean	Lower Bound	.9967	
		Upper Bound	1.0233	
	5% Trimmed Mean		1.0094	
	Median		1.0050	
	Variance		.000	
	Std. Deviation		.01265	
	Minimum		1.00	
	Maximum		1.03	
	Range		.03	
	Interquartile Range		.02	
	Skewness		.889	.845
	Kurtosis		-.781	1.741
bcll	Mean		5.1833	.59745
	95% Confidence Interval for Mean	Lower Bound	3.6475	
		Upper Bound	6.7191	
	5% Trimmed Mean		5.2037	
	Median		5.2000	

Descriptives

			Statistic	Std. Error
	Variance		2.142	
	Std. Deviation		1.46344	
	Minimum		2.90	
	Maximum		7.10	
	Range		4.20	
	Interquartile Range		2.33	
	Skewness		-.394	.845
	Kurtosis		.162	1.741
pi3k1	Mean		1.0167	.00667
	95% Confidence Interval for Mean	Lower Bound	.9995	
		Upper Bound	1.0338	
	5% Trimmed Mean		1.0163	
	Median		1.0150	
	Variance		.000	
	Std. Deviation		.01633	
	Minimum		1.00	
	Maximum		1.04	
	Range		.04	
	Interquartile Range		.03	
	Skewness		.383	.845
	Kurtosis		-1.481	1.741
pi3k12	Mean		1.3433	.01430
	95% Confidence Interval for Mean	Lower Bound	1.3066	
		Upper Bound	1.3801	
	5% Trimmed Mean		1.3426	
	Median		1.3400	
	Variance		.001	
	Std. Deviation		.03502	
	Minimum		1.30	
	Maximum		1.40	
	Range		.10	
	Interquartile Range		.06	
	Skewness		.632	.845
	Kurtosis		.377	1.741
akt1	Mean		1.0183	.00601

Descriptives

			Statistic	Std. Error
	95% Confidence Interval for Mean	Lower Bound	1.0029	
		Upper Bound	1.0338	
	5% Trimmed Mean		1.0181	
	Median		1.0150	
	Variance		.000	
	Std. Deviation		.01472	
	Minimum		1.00	
	Maximum		1.04	
	Range		.04	
	Interquartile Range		.02	
	Skewness		.418	.845
	Kurtosis		-.859	1.741
akt12	Mean		1.3733	.01116
	95% Confidence Interval for Mean	Lower Bound	1.3447	
		Upper Bound	1.4020	
	5% Trimmed Mean		1.3737	
	Median		1.3750	
	Variance		.001	
	Std. Deviation		.02733	
	Minimum		1.33	
	Maximum		1.41	
	Range		.08	
	Interquartile Range		.04	
	Skewness		-.435	.845
	Kurtosis		.586	1.741
mtor1	Mean		1.0150	.00428
	95% Confidence Interval for Mean	Lower Bound	1.0040	
		Upper Bound	1.0260	
	5% Trimmed Mean		1.0150	
	Median		1.0150	
	Variance		.000	
	Std. Deviation		.01049	
	Minimum		1.00	
	Maximum		1.03	
	Range		.03	

Descriptives

			Statistic	Std. Error
	Interquartile Range		.01	
	Skewness		.000	.845
	Kurtosis		-.248	1.741
mtor12	Mean		1.3583	.01515
	95% Confidence Interval for Mean	Lower Bound	1.3194	
		Upper Bound	1.3973	
	5% Trimmed Mean		1.3570	
	Median		1.3500	
	Variance		.001	
	Std. Deviation		.03710	
	Minimum		1.32	
	Maximum		1.42	
	Range		.10	
	Interquartile Range		.06	
	Skewness		.936	.845
	Kurtosis		.250	1.741

Tests of Normality

	Kolmogorov-Smirnov[a]			Shapiro-Wilk		
	Statistic	df	Sig.	Statistic	df	Sig.
proliferation	.195	6	.200*	.905	6	.404
proliferation2	.257	6	.200*	.872	6	.235
bax1	.122	6	.200*	.982	6	.961
bax12	.174	6	.200*	.947	6	.719
bcl	.285	6	.138	.831	6	.110
bcll	.154	6	.200*	.985	6	.975
pi3k1	.180	6	.200*	.920	6	.505
pi3k12	.150	6	.200*	.974	6	.918
akt1	.214	6	.200*	.958	6	.804
akt12	.146	6	.200*	.988	6	.985
mtor1	.183	6	.200*	.960	6	.820
mtor12	.189	6	.200*	.932	6	.596

*. This is a lower bound of the true significance.

a. Lilliefors Significance Correction

proliferation

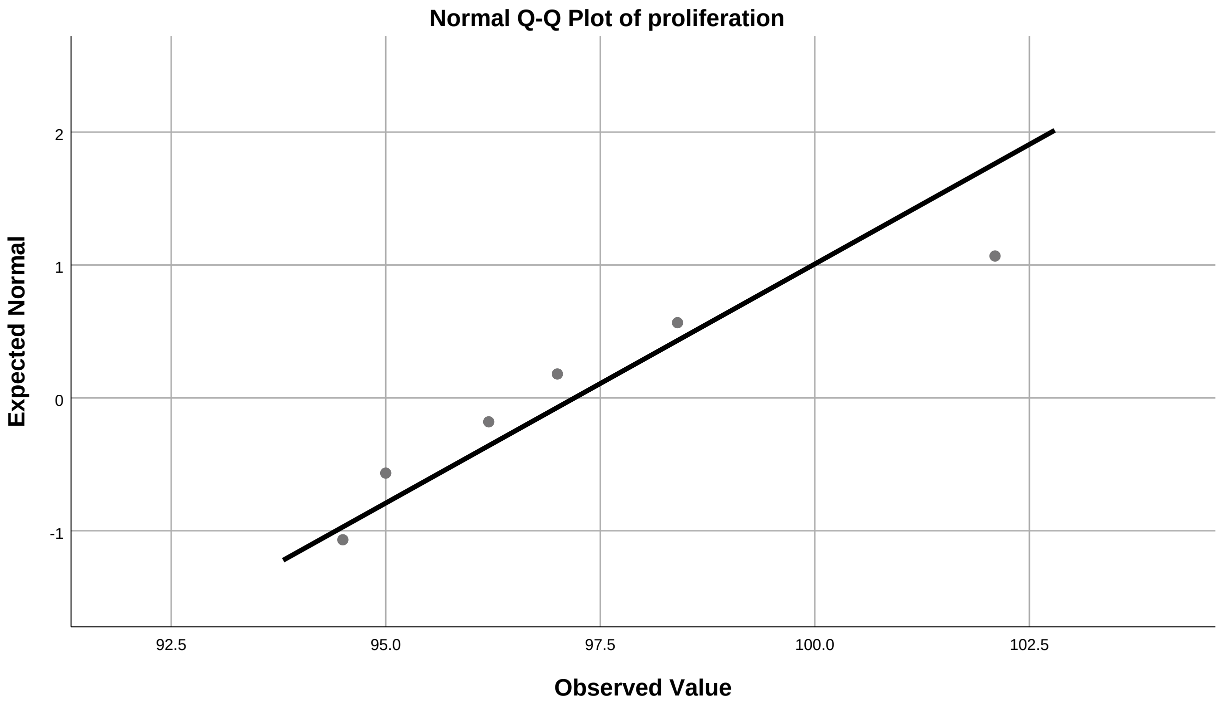

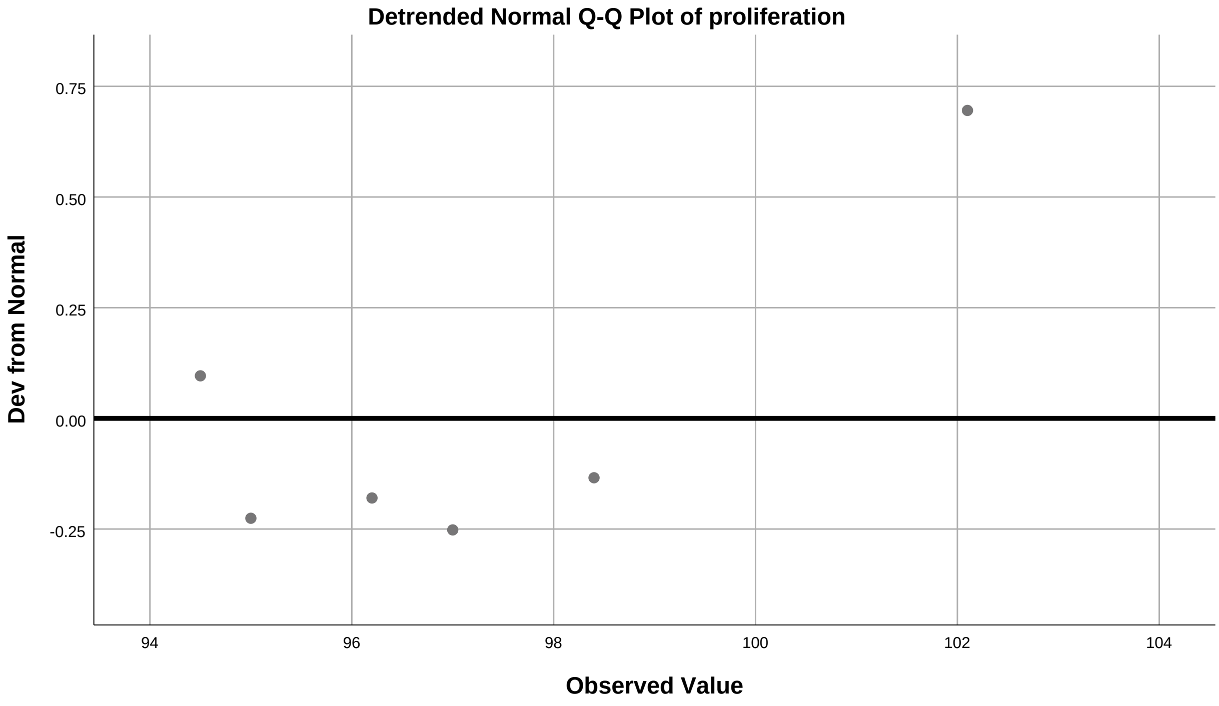

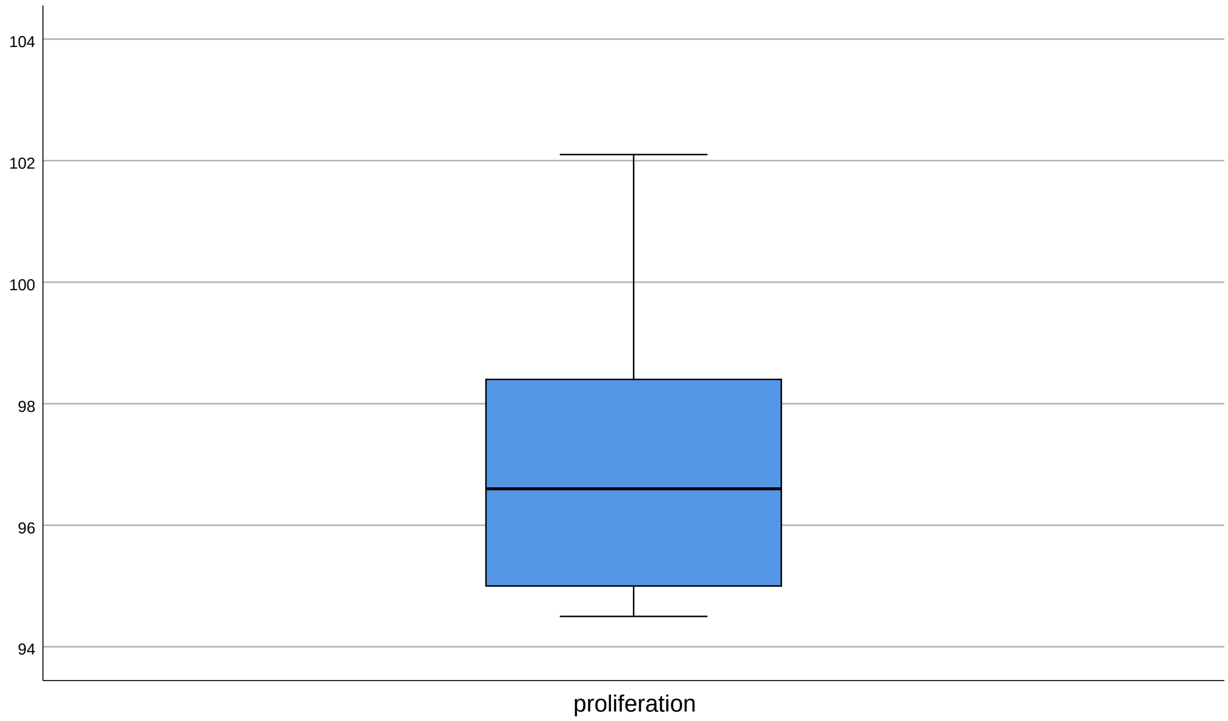

proliferation2

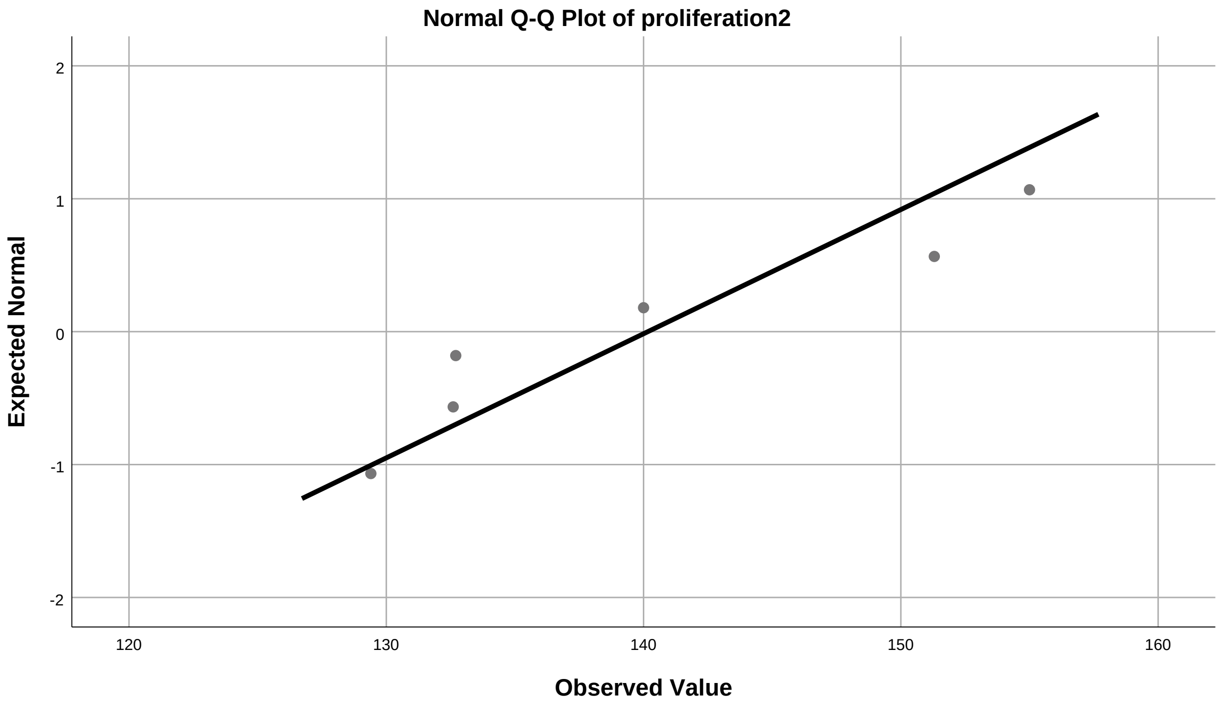

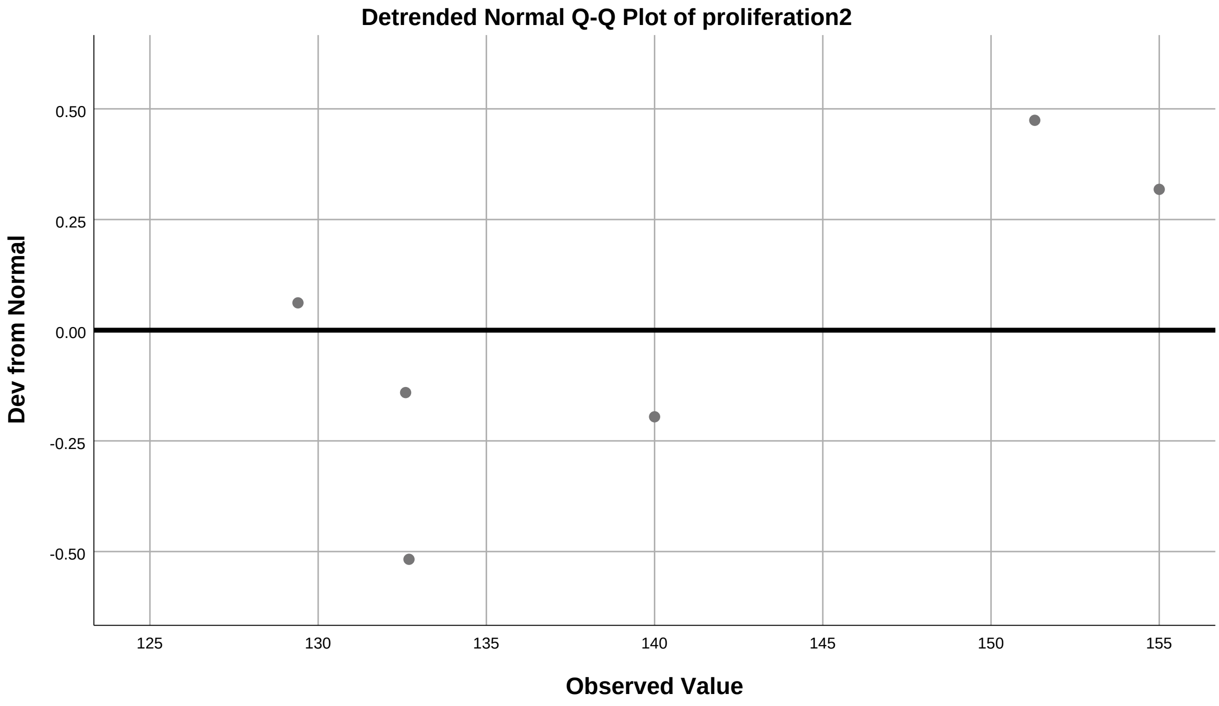
Detrended Normal Q-Q Plot of proliferation2
Dev from Normal
0.50
0.25
0.00
-0.25
-0.50
125
130
135
140
145
150
155
Observed Value

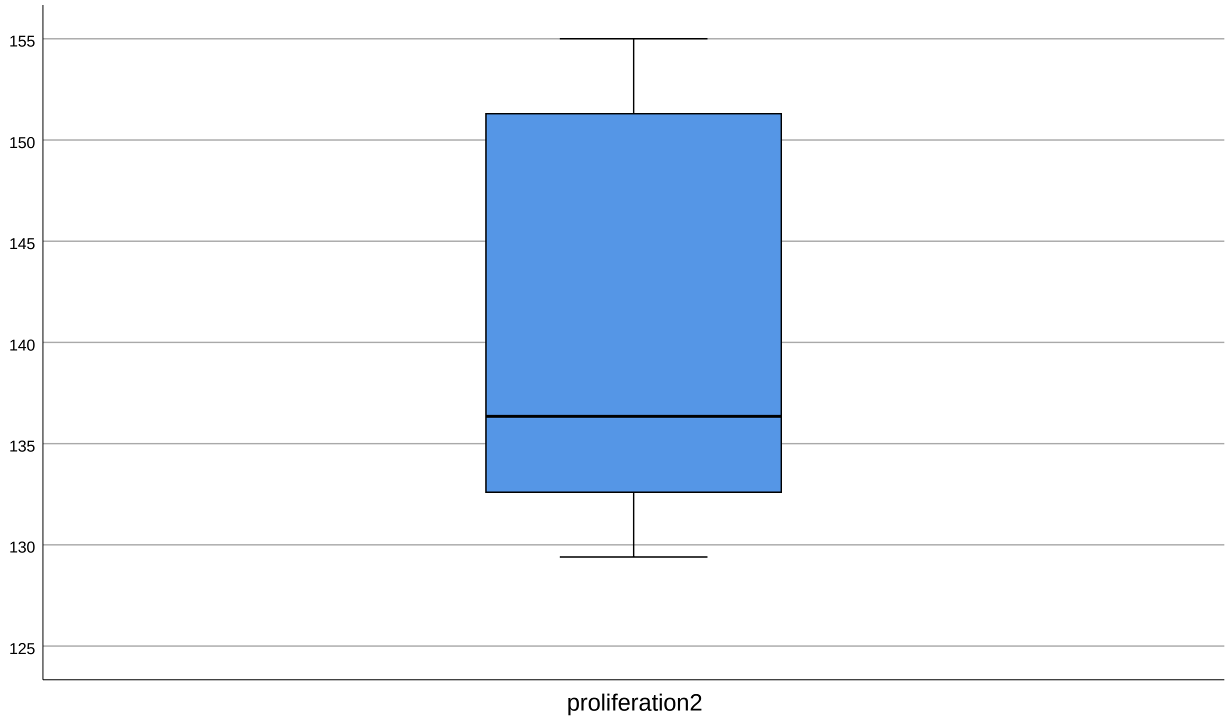
155
150
145
140
135
130
125
proliferation2

bax1

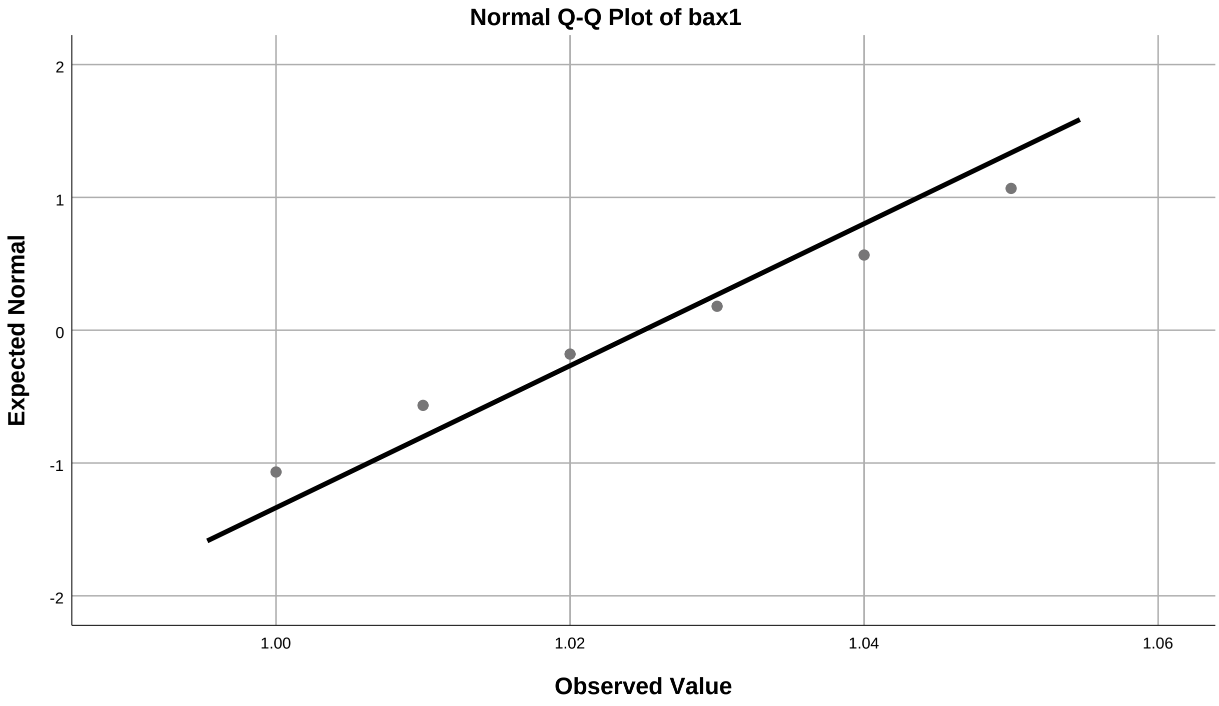
Normal Q-Q Plot of bax1
Expected Normal
2
1
0
-1
-2
1.00
1.02
1.04
1.06
Observed Value

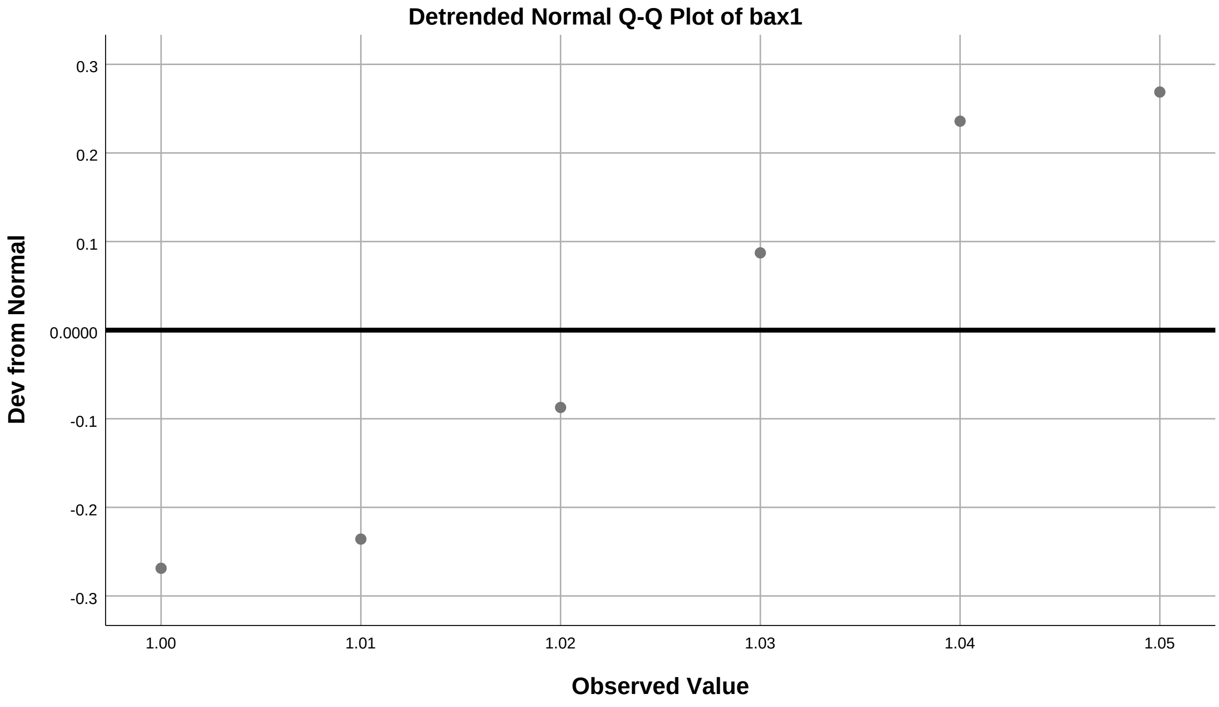
Detrended Normal Q-Q Plot of bax1
Dev from Normal
0.3
0.2
0.1
0.0000
-0.1
-0.2
-0.3
1.00
1.01
1.02
1.03
1.04
1.05
Observed Value

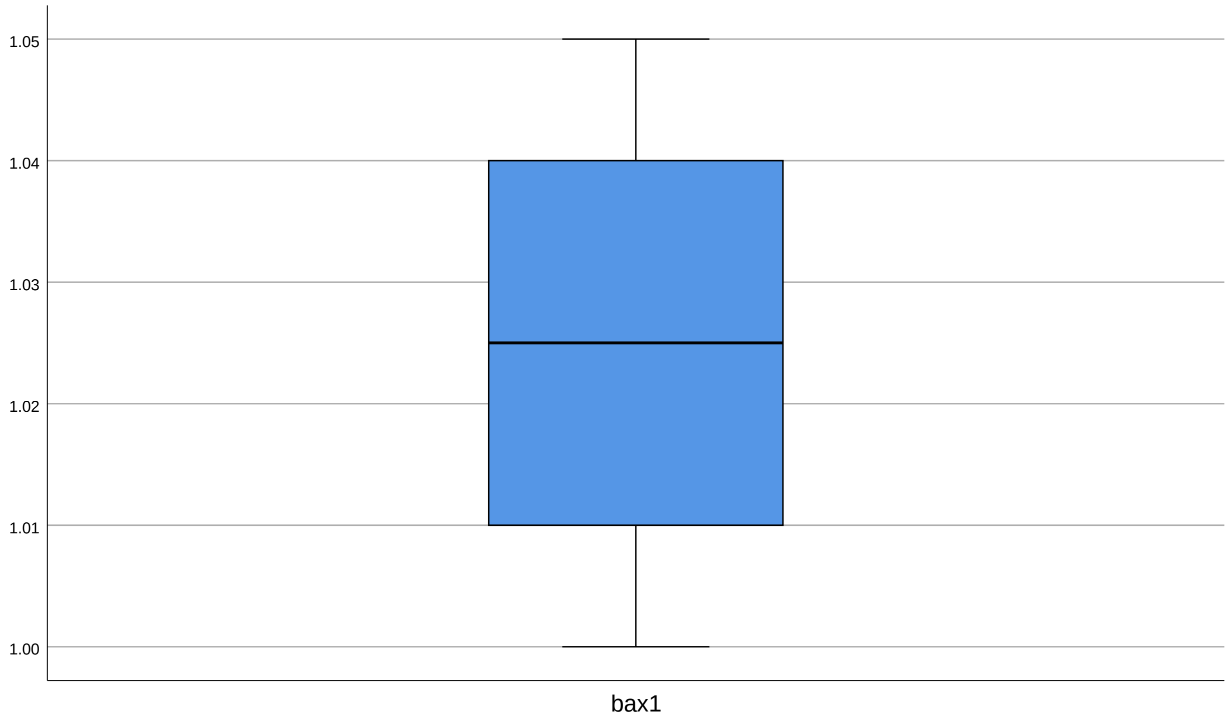
1.05
1.04
1.03
1.02
1.01
1.00
bax1

bax12

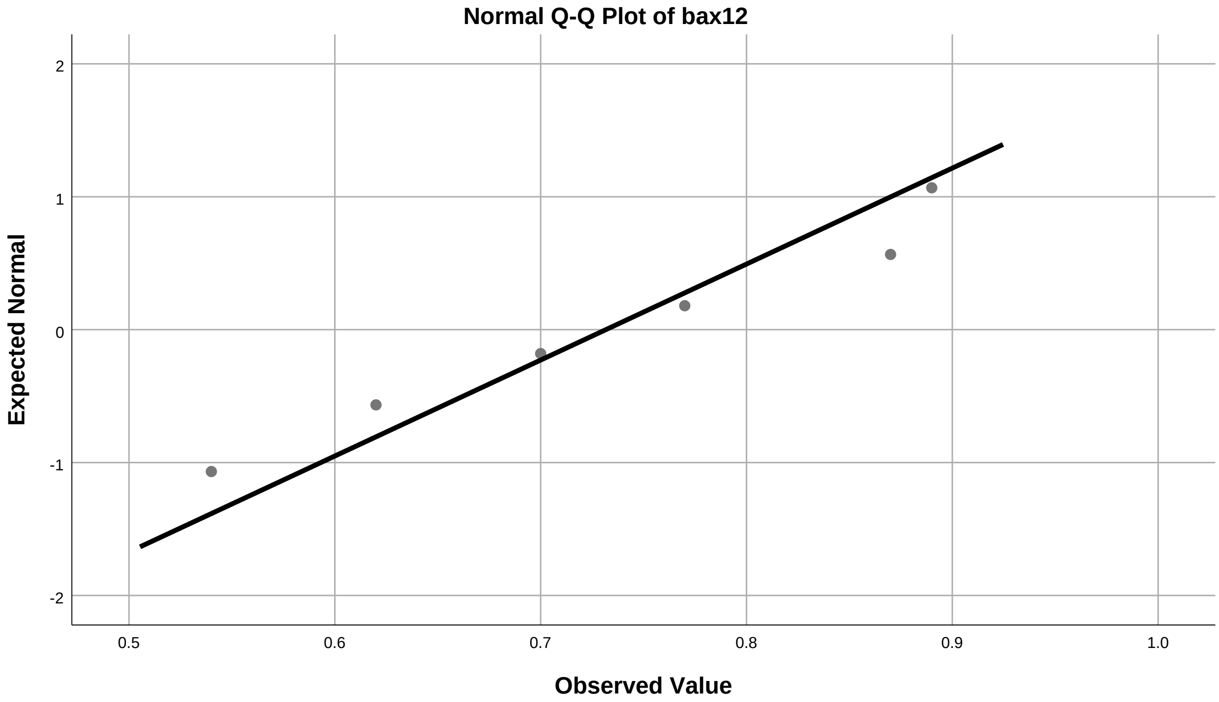
Normal Q-Q Plot of bax12
Expected Normal
2
1
0
-1
-2
0.5
0.6
0.7
0.8
0.9
1.0
Observed Value

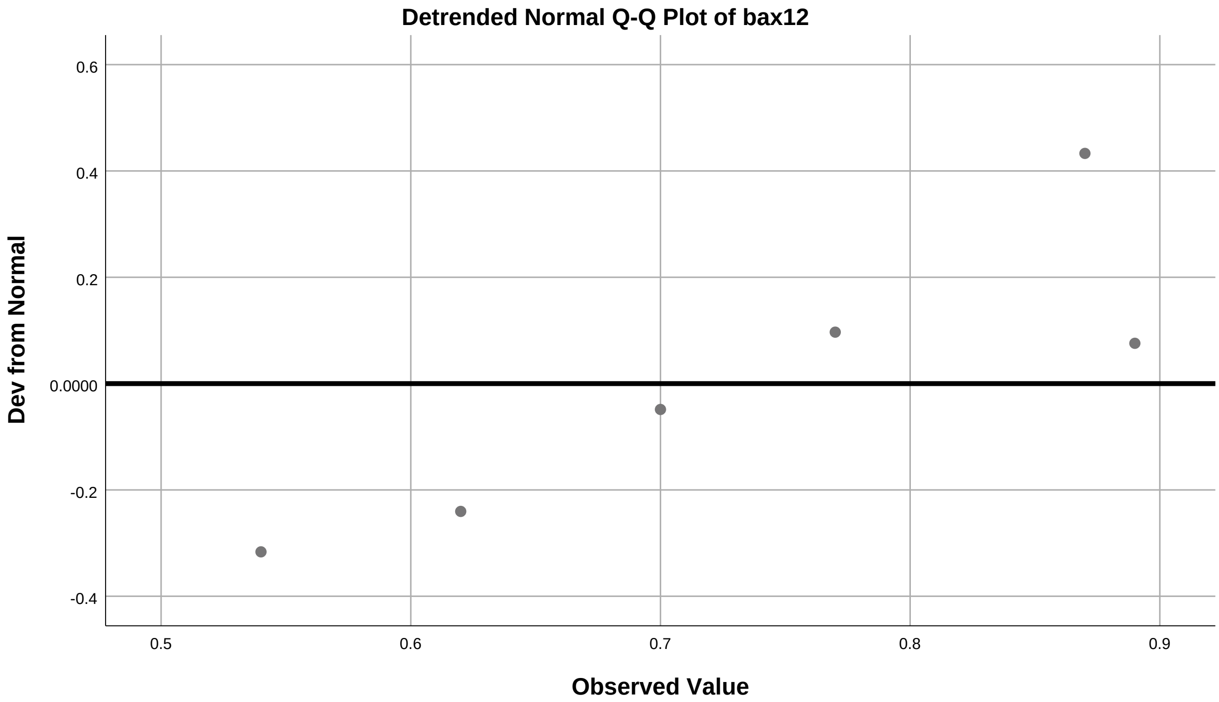
Detrended Normal Q-Q Plot of bax12
Dev from Normal
0.6
0.4
0.2
0.0000
-0.2
-0.4
0.5
0.6
0.7
0.8
0.9
Observed Value

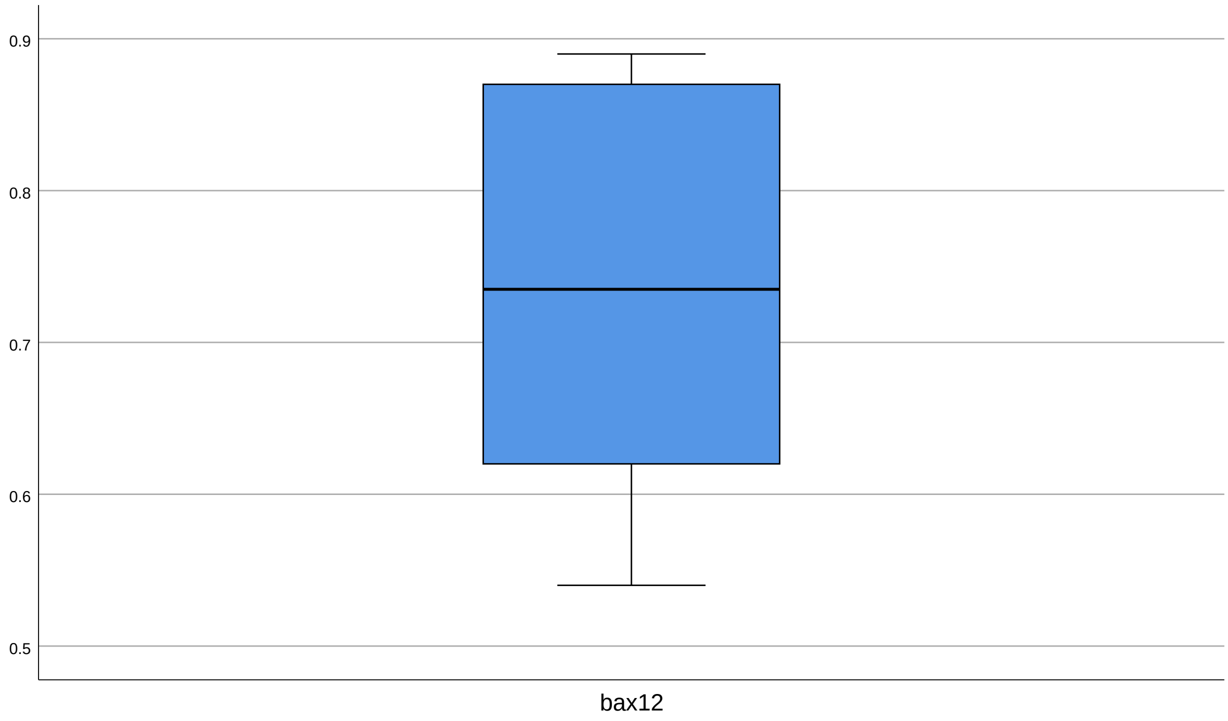
0.9
0.8
0.7
0.6
0.5
bax12

bcl

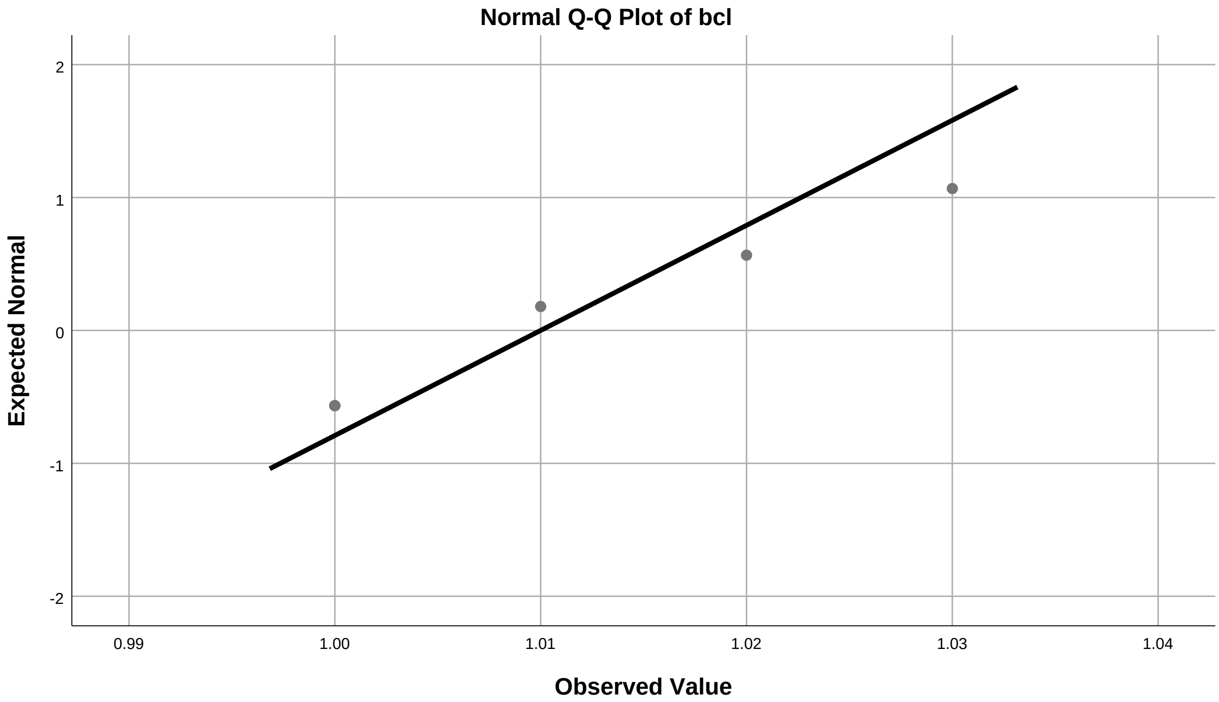
Normal Q-Q Plot of bcl
Expected Normal
Observed Value
2
1
0
-1
-2
0.99
1.00
1.01
1.02
1.03
1.04

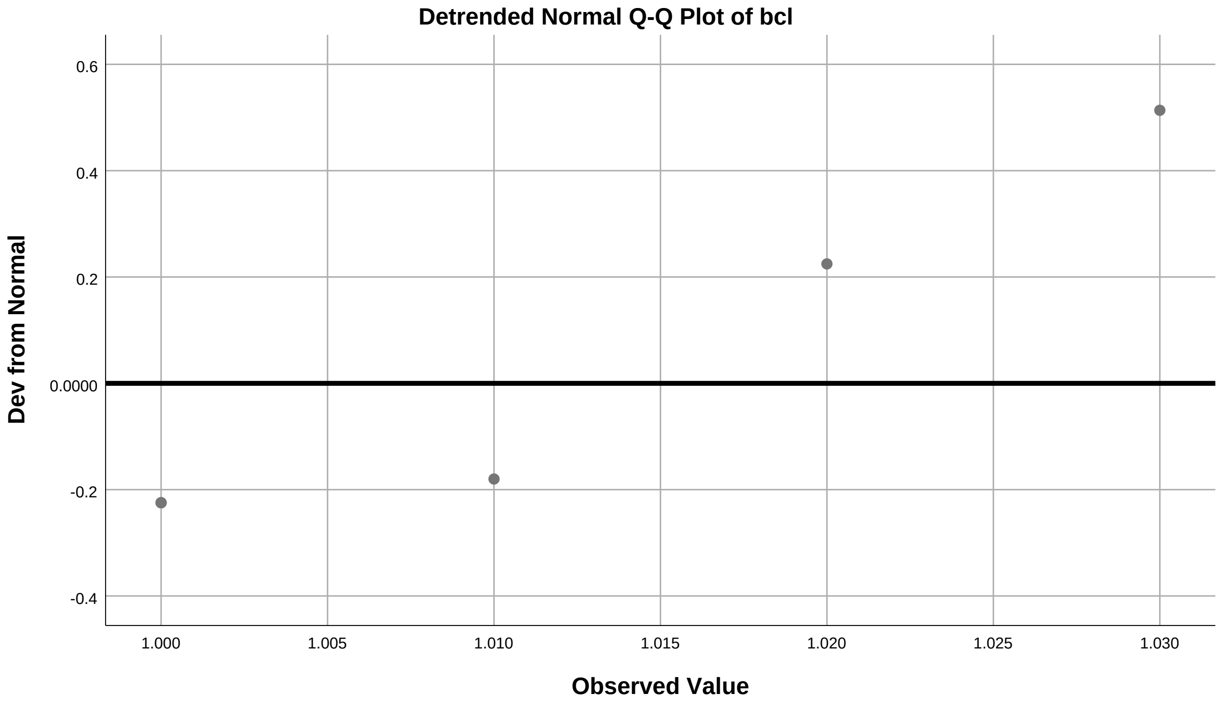
Detrended Normal Q-Q Plot of bcl
Dev from Normal
Observed Value
0.6
0.4
0.2
0.0000
-0.2
-0.4
1.000
1.005
1.010
1.015
1.020
1.025
1.030

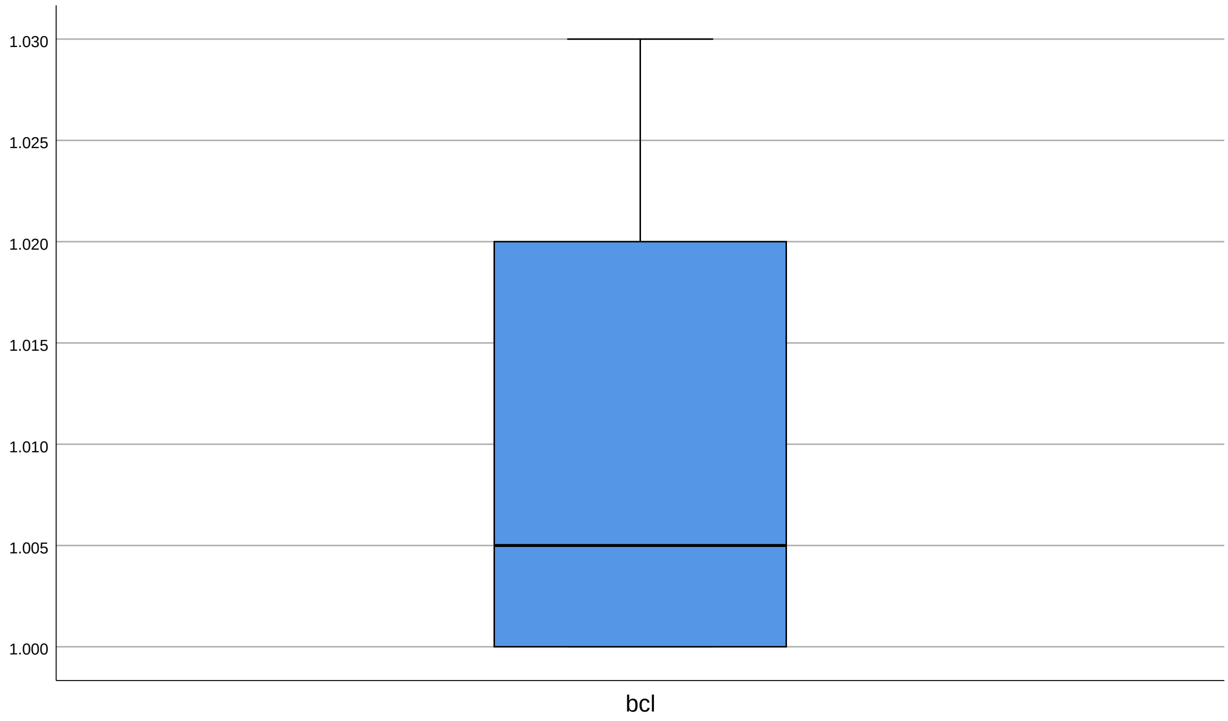
1.030
1.025
1.020
1.015
1.010
1.005
1.000
bcl

bcll

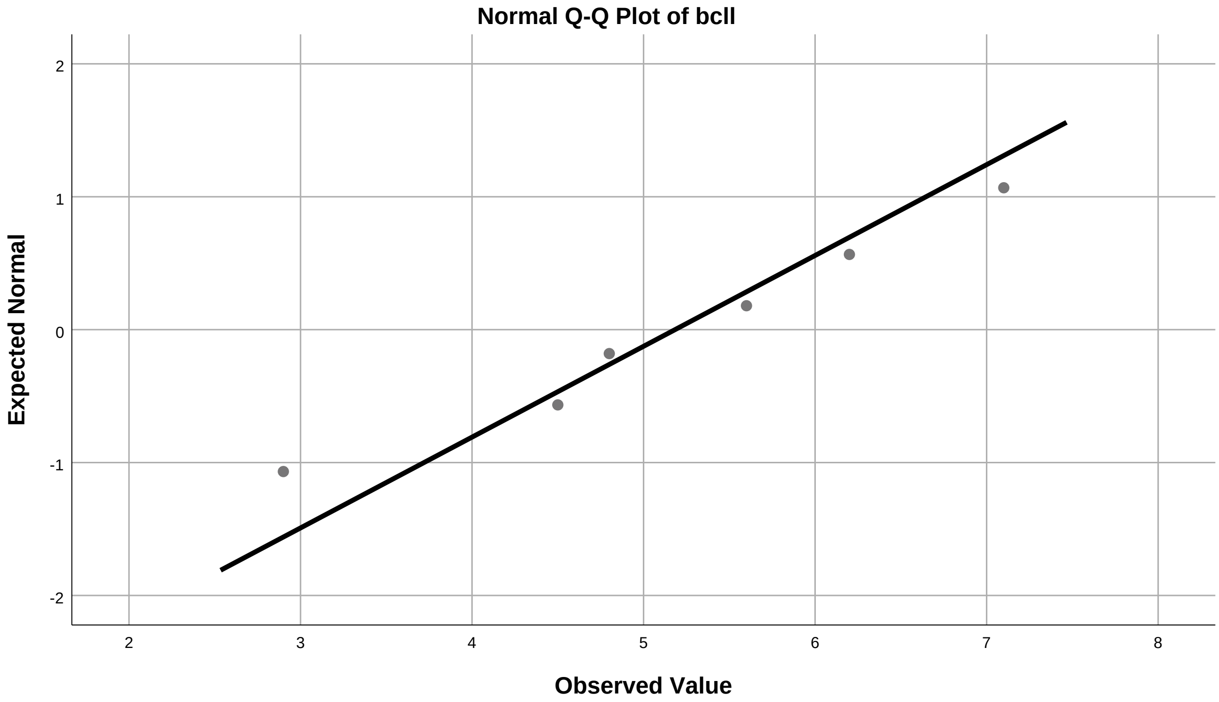
Normal Q-Q Plot of bcll
Expected Normal
2
1
0
-1
-2
2
3
4
5
6
7
8
Observed Value

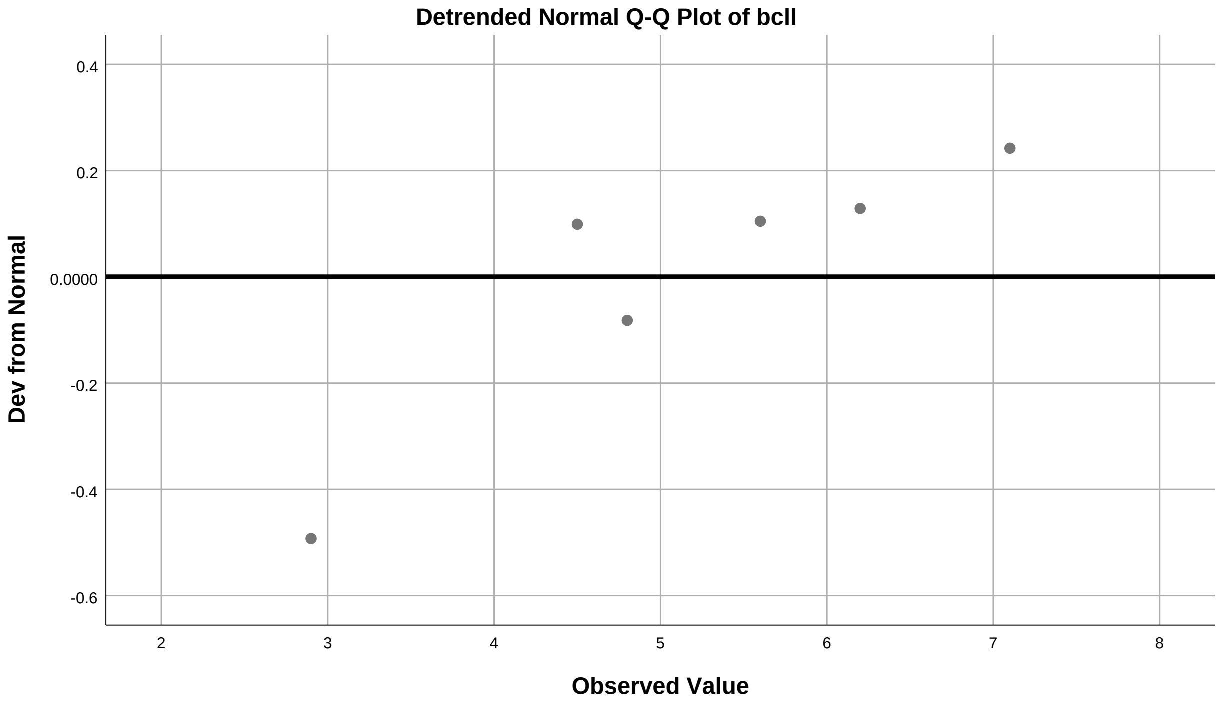
Detrended Normal Q-Q Plot of bcll
Dev from Normal
0.4
0.2
0.0000
-0.2
-0.4
-0.6
2
3
4
5
6
7
8
Observed Value

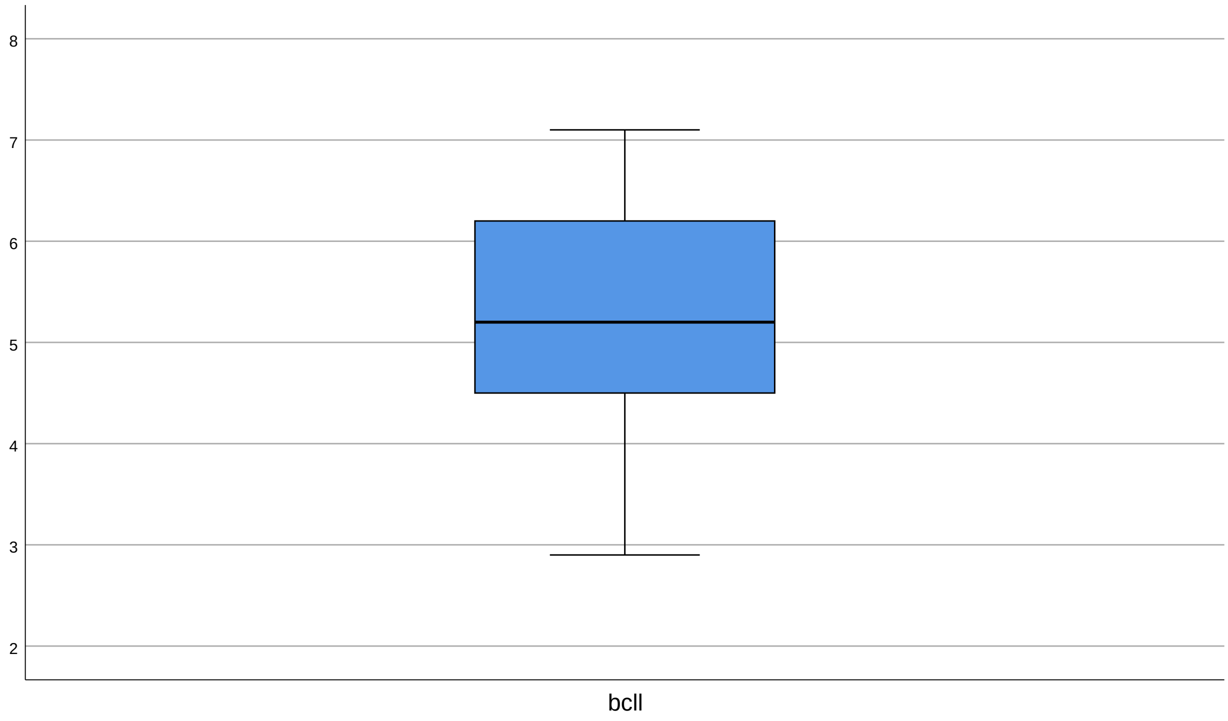
8
7
6
5
4
3
2
bcll

pi3k1

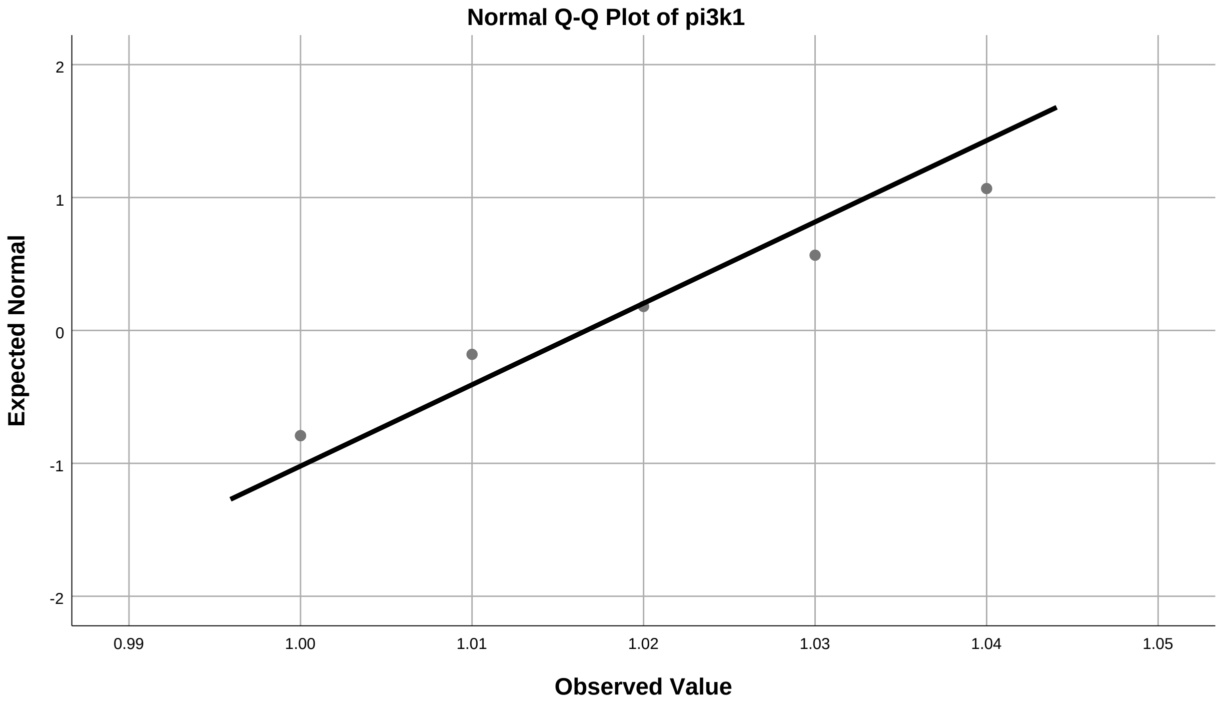
Normal Q-Q Plot of pi3k1
Expected Normal
2
1
0
-1
-2
0.99
1.00
1.01
1.02
1.03
1.04
1.05
Observed Value

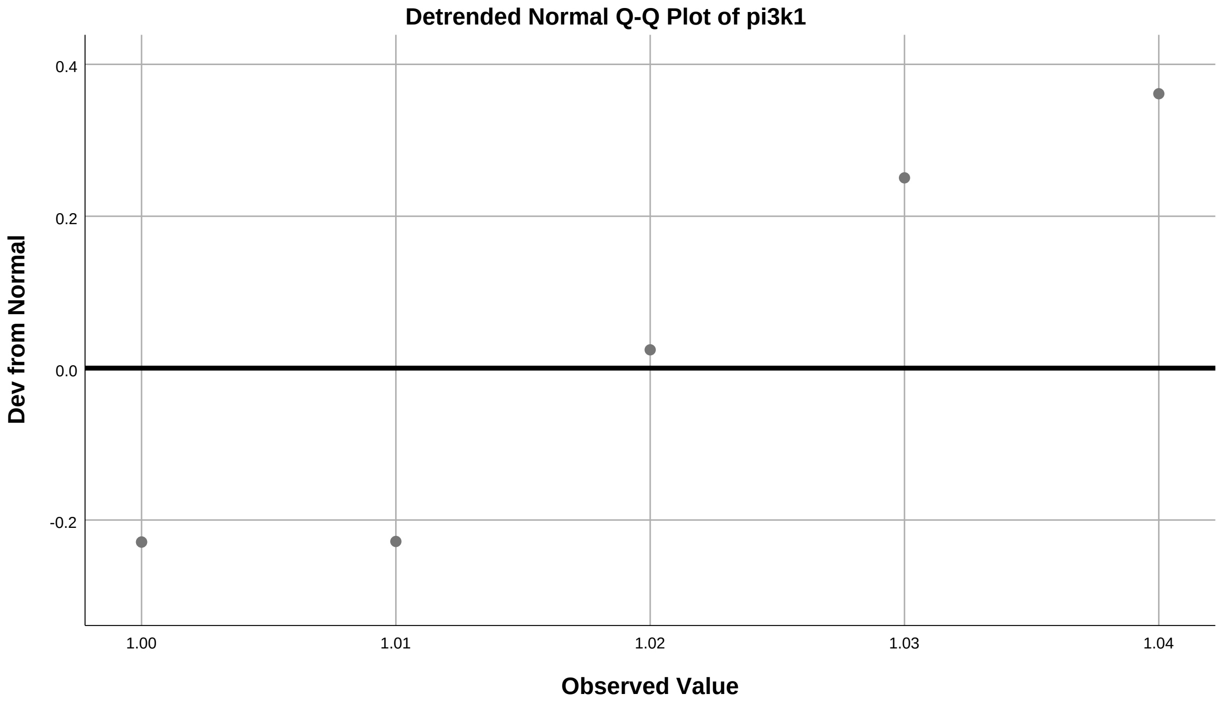
Detrended Normal Q-Q Plot of pi3k1
Dev from Normal
0.4
0.2
0.0
-0.2
1.00
1.01
1.02
1.03
1.04
Observed Value

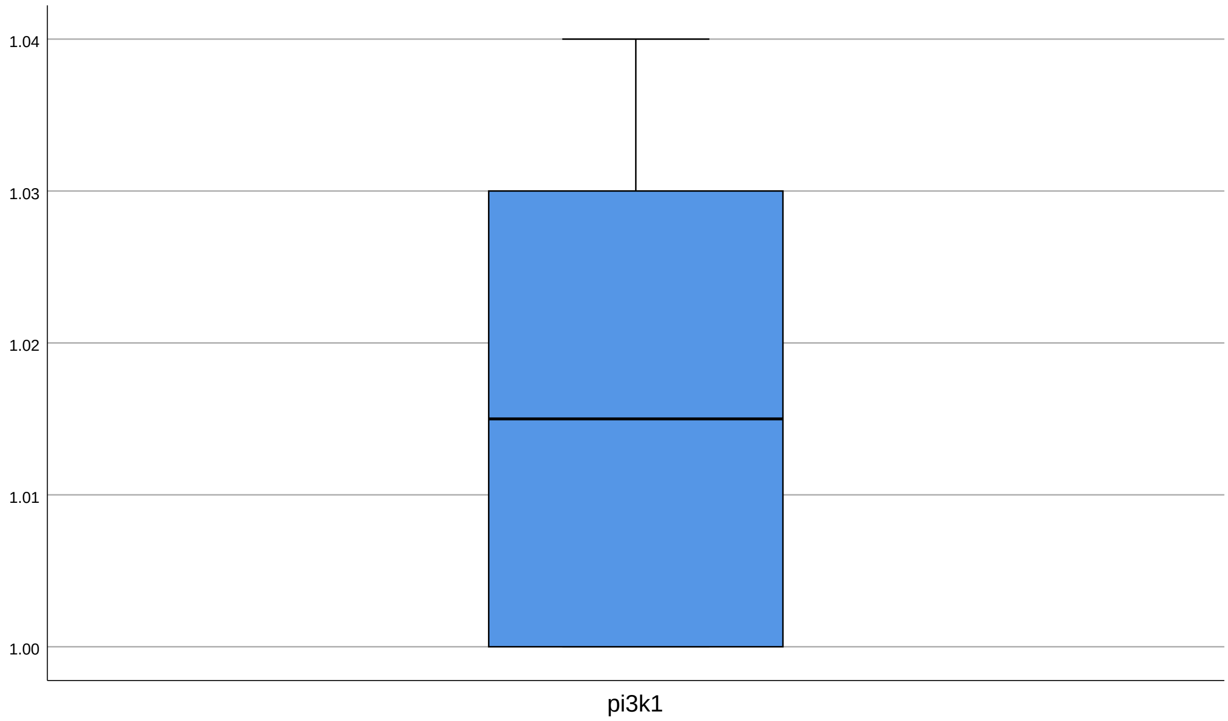

1.04
1.03
1.02
1.01
1.00
pi3k1

pi3k12

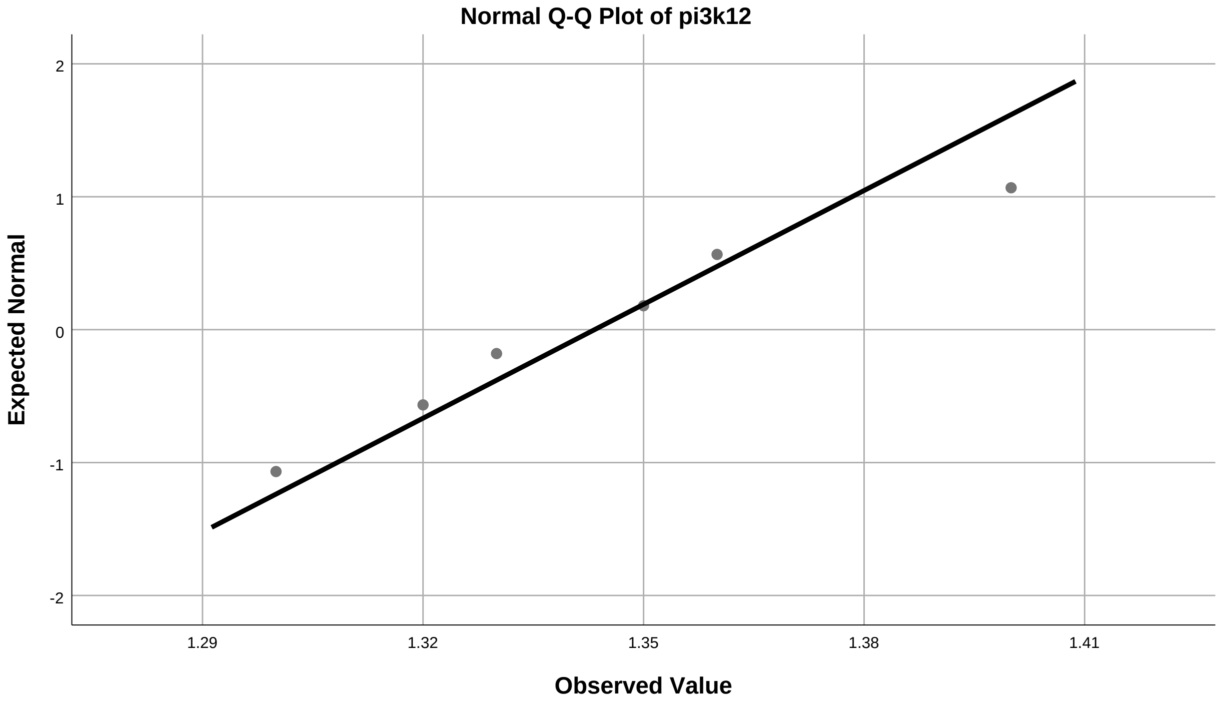

Normal Q-Q Plot of pi3k12
Expected Normal
2
1
0
-1
-2
1.29
1.32
1.35
1.38
1.41
Observed Value

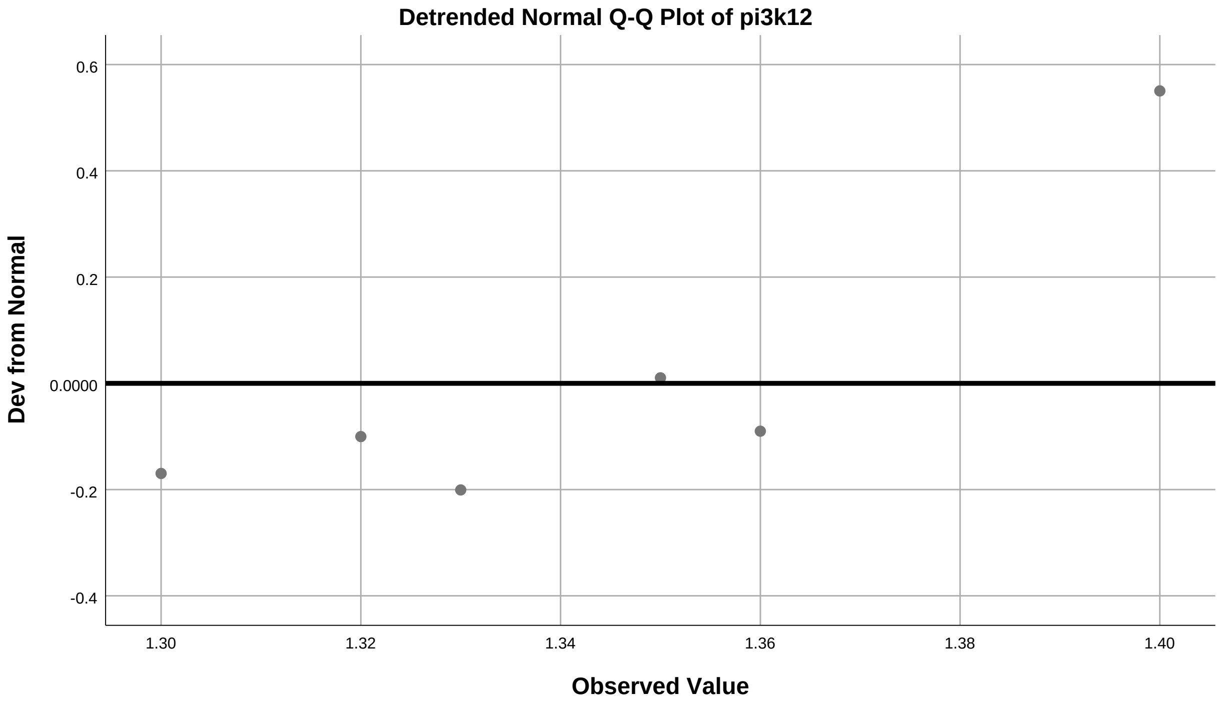
Detrended Normal Q-Q Plot of pi3k12
Dev from Normal
0.6
0.4
0.2
0.0000
-0.2
-0.4
1.30
1.32
1.34
1.36
1.38
1.40
Observed Value

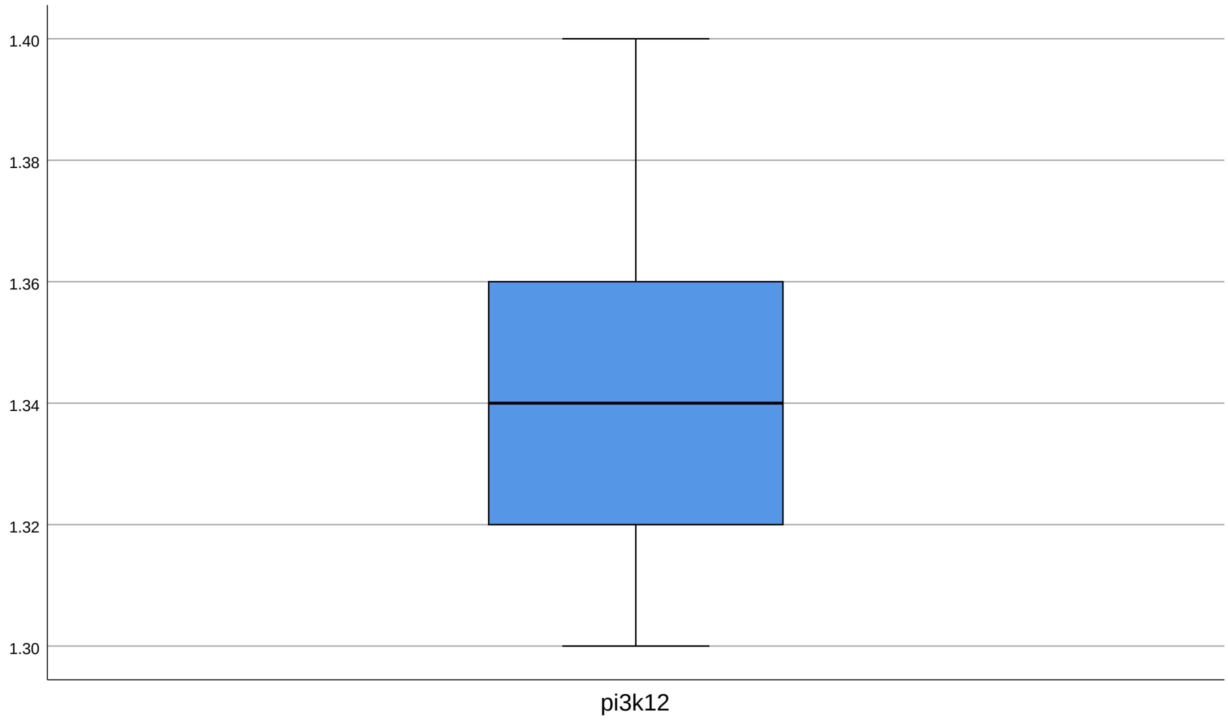
1.40
1.38
1.36
1.34
1.32
1.30
pi3k12

akt1

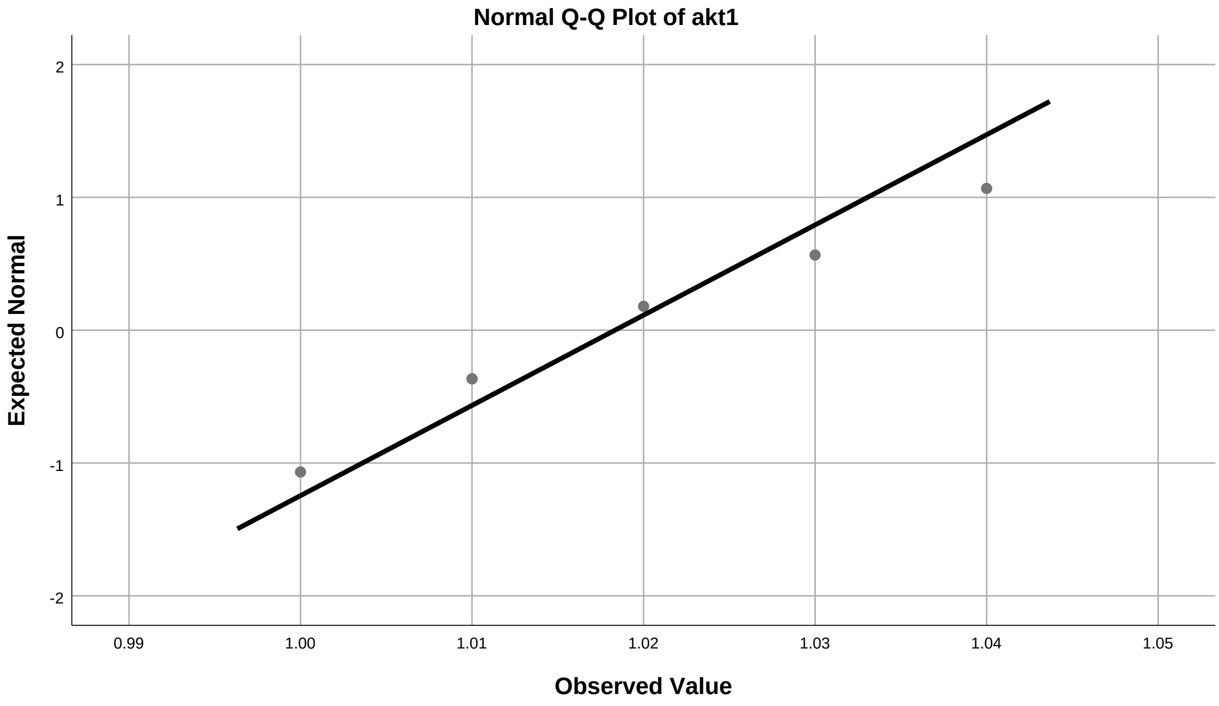

Normal Q-Q Plot of akt1
Expected Normal
2
1
0
-1
-2
0.99
1.00
1.01
1.02
1.03
1.04
1.05
Observed Value

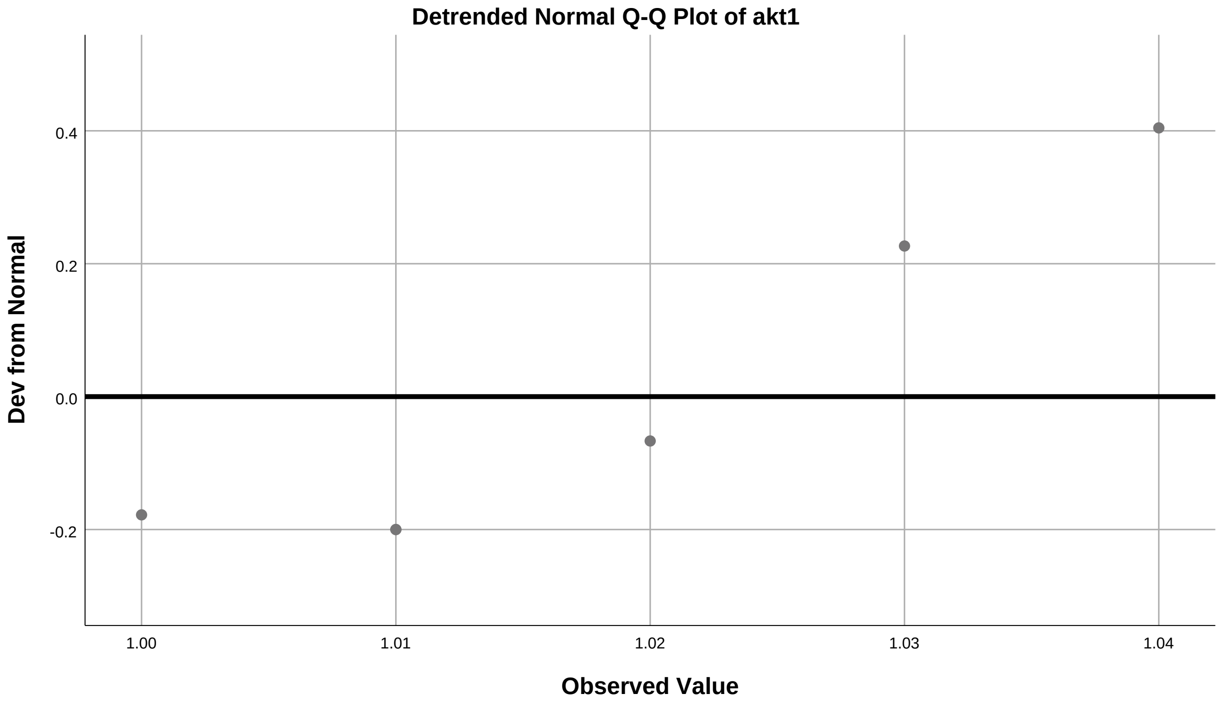

Detrended Normal Q-Q Plot of akt1
Dev from Normal
0.4
0.2
0.0
-0.2
1.00
1.01
1.02
1.03
1.04
Observed Value

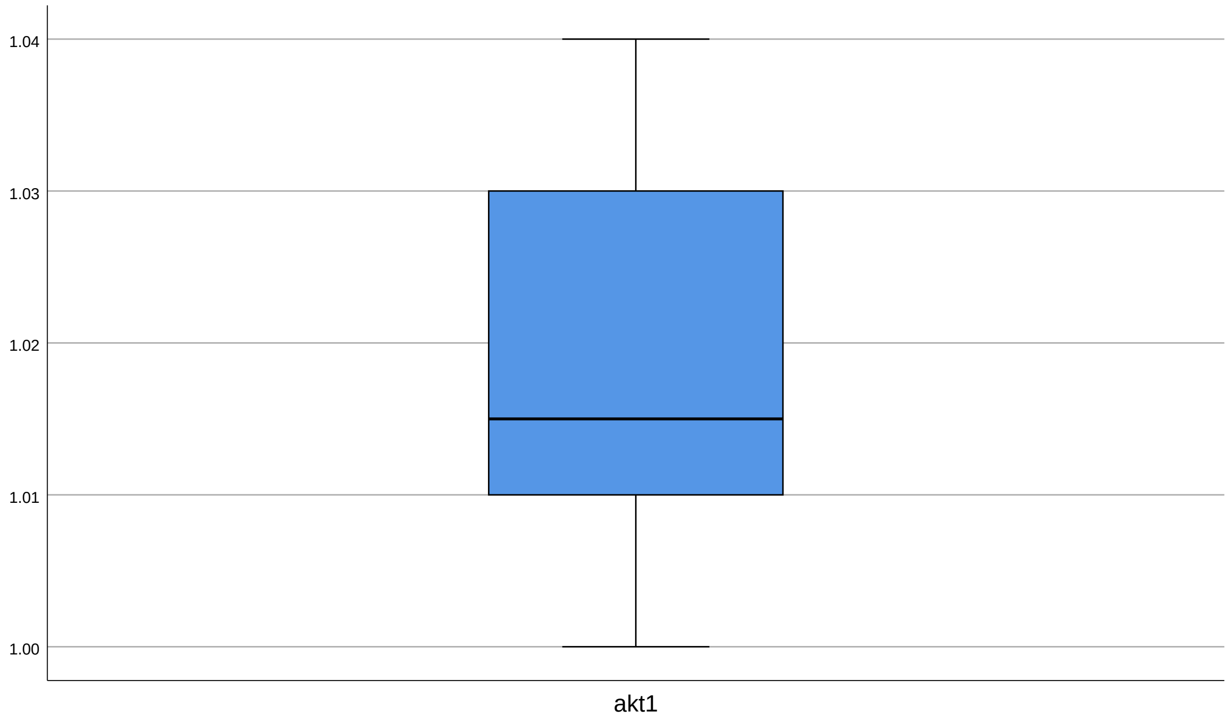
1.04
1.03
1.02
1.01
1.00
akt1

akt12

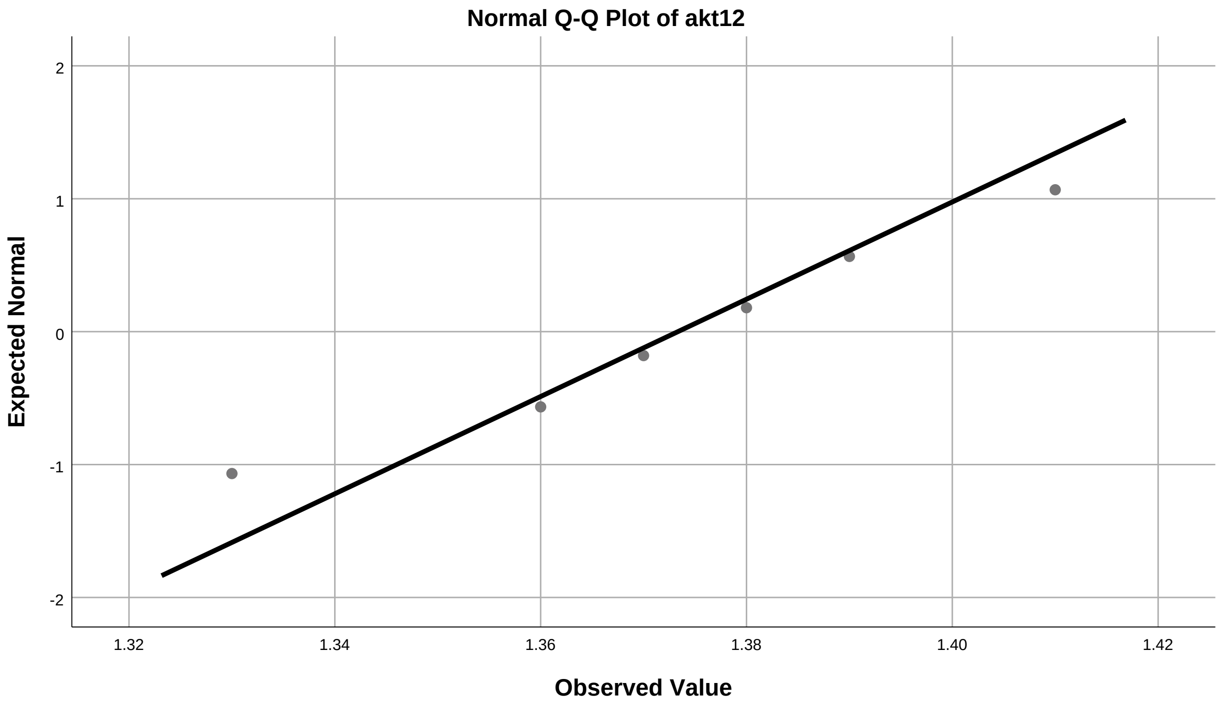
Normal Q-Q Plot of akt12
Expected Normal
2
1
0
-1
-2
1.32
1.34
1.36
1.38
1.40
1.42
Observed Value

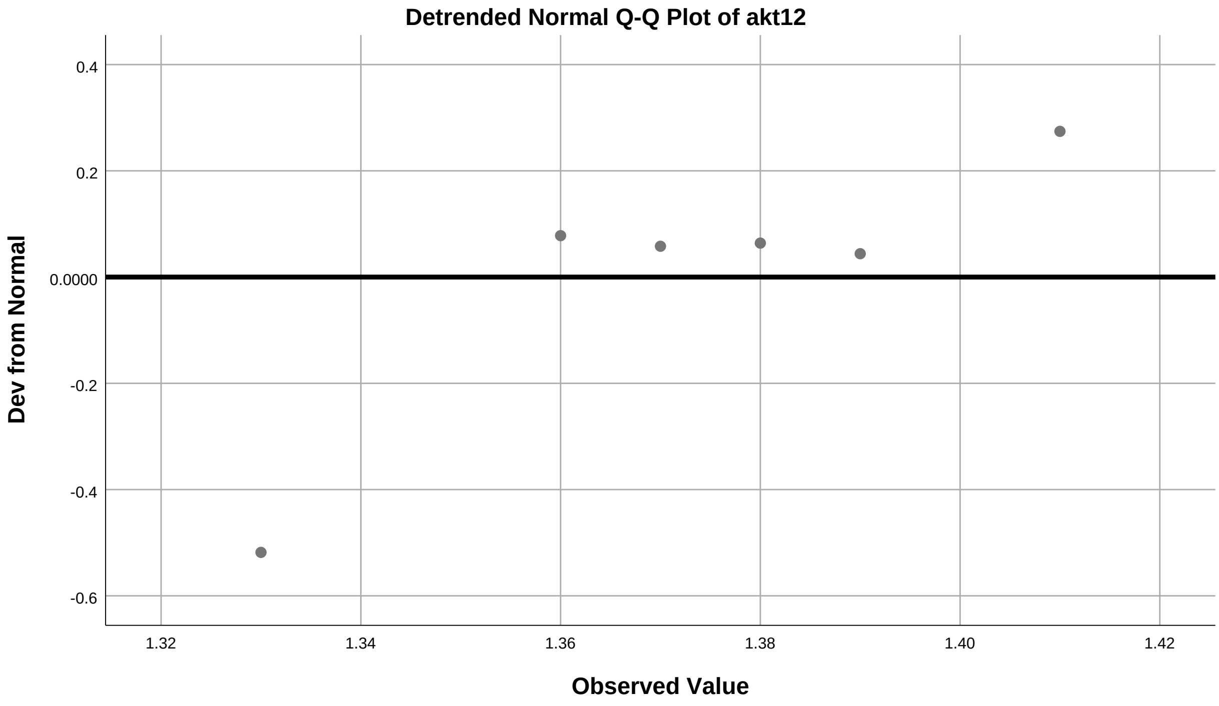

Detrended Normal Q-Q Plot of akt12
Dev from Normal
0.4
0.2
0.0000
-0.2
-0.4
-0.6
1.32
1.34
1.36
1.38
1.40
1.42
Observed Value

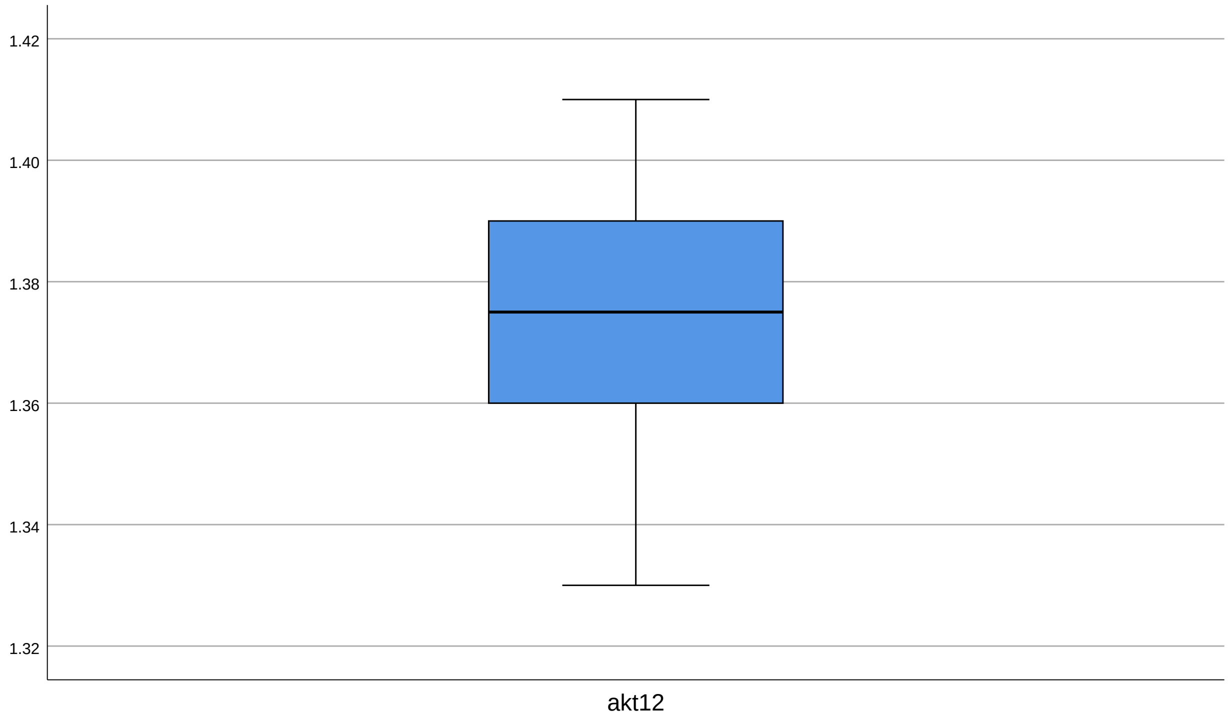

1.42
1.40
1.38
1.36
1.34
1.32
akt12

mtor1

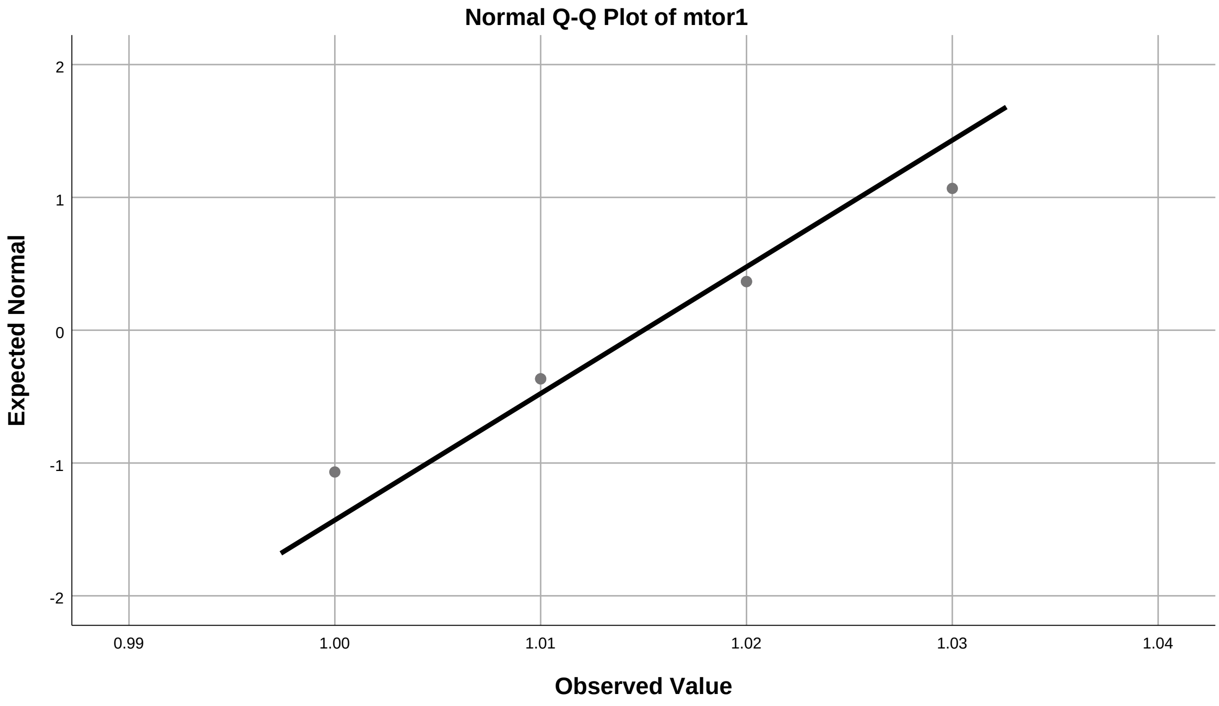
Normal Q-Q Plot of mtor1
Expected Normal
Observed Value
2
1
0
-1
-2
0.99
1.00
1.01
1.02
1.03
1.04

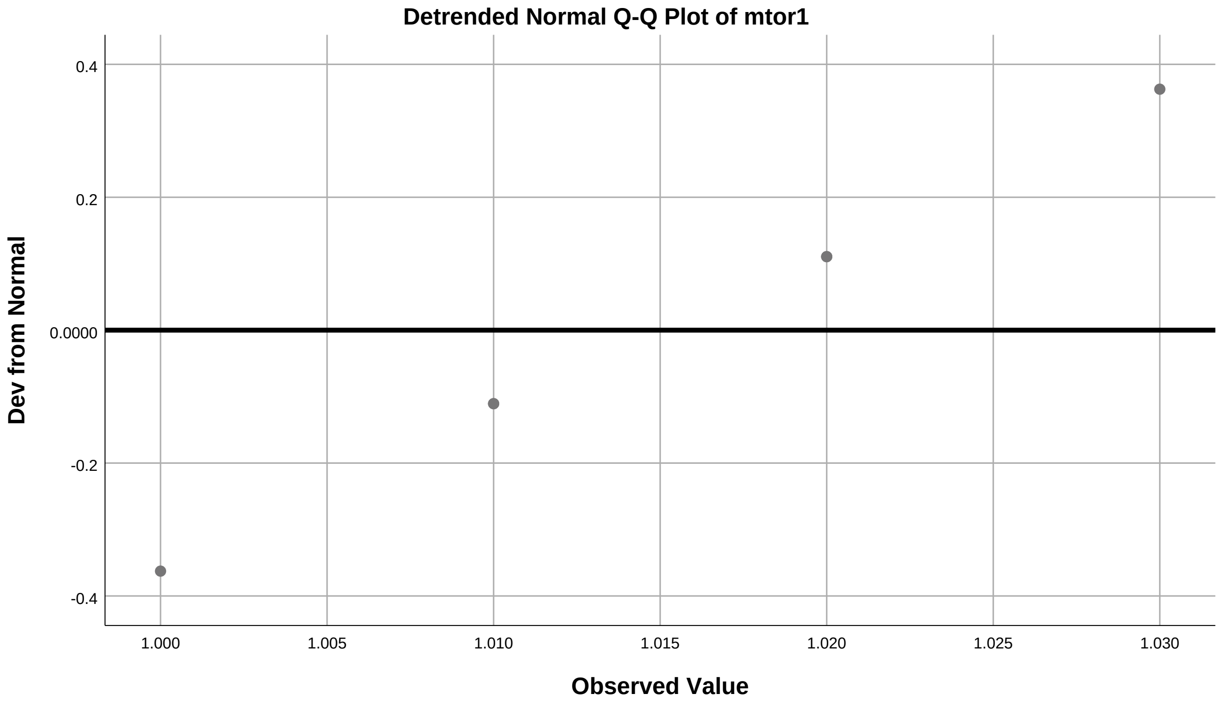
Detrended Normal Q-Q Plot of mtor1
Dev from Normal
Observed Value
0.4
0.2
0.0000
-0.2
-0.4
1.000
1.005
1.010
1.015
1.020
1.025
1.030

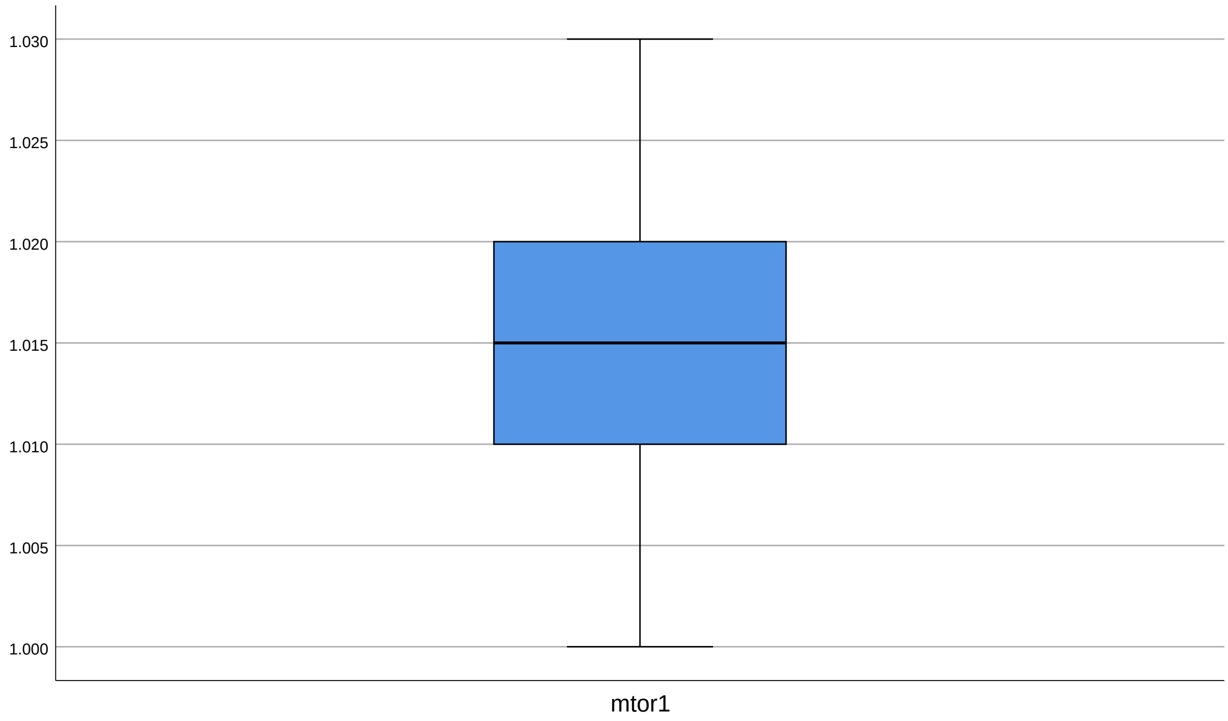
1.030
1.025
1.020
1.015
1.010
1.005
1.000
mtor1

mtor12

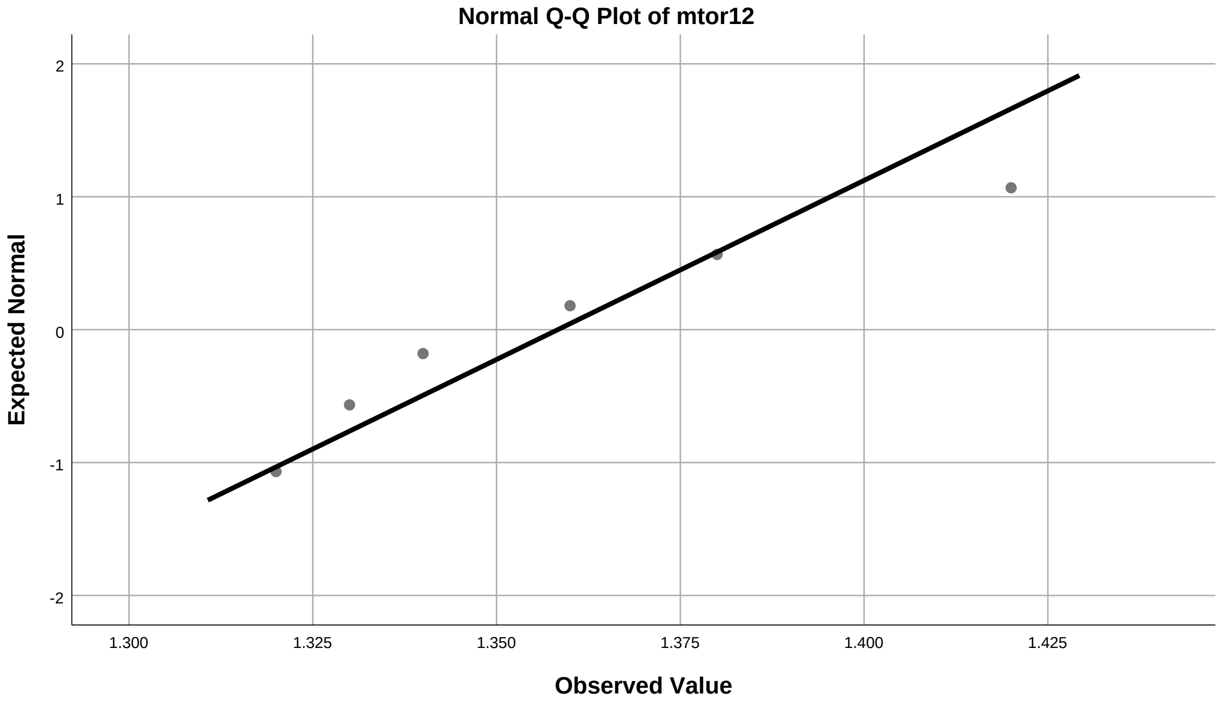
Normal Q-Q Plot of mtor12
Expected Normal
2
1
0
-1
-2
1.300
1.325
1.350
1.375
1.400
1.425
Observed Value

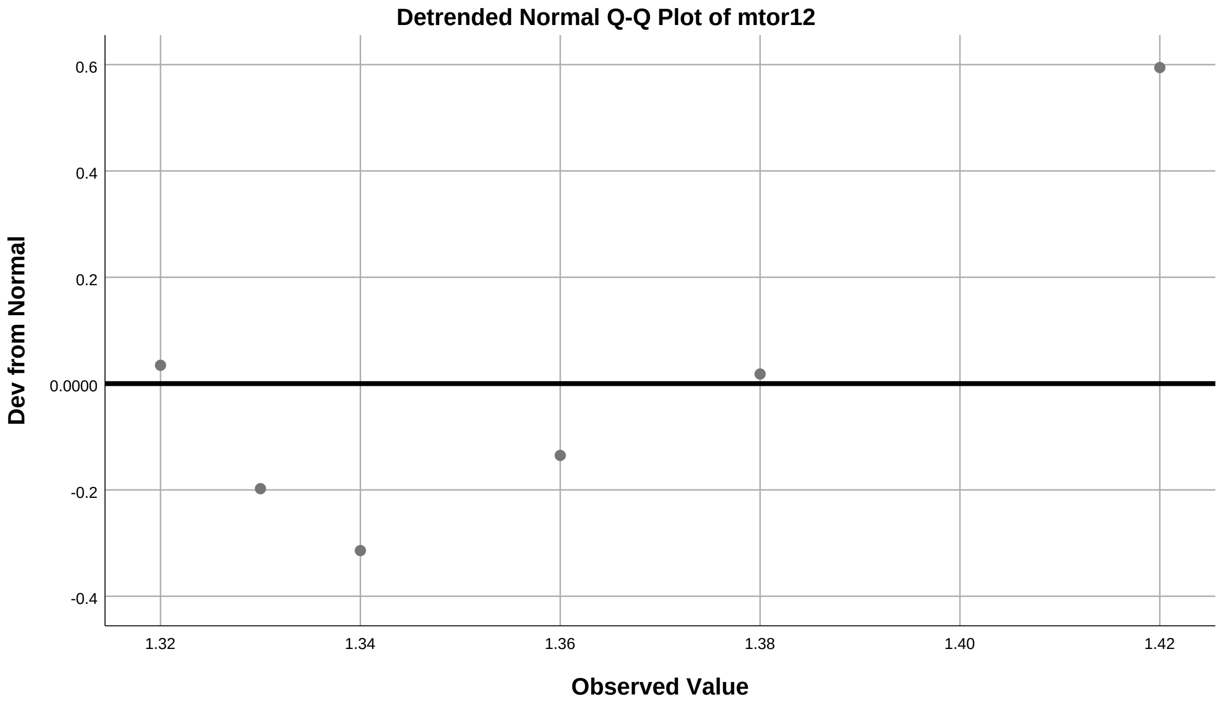
Detrended Normal Q-Q Plot of mtor12
Dev from Normal
0.6
0.4
0.2
0.0000
-0.2
-0.4
1.32
1.34
1.36
1.38
1.40
1.42
Observed Value

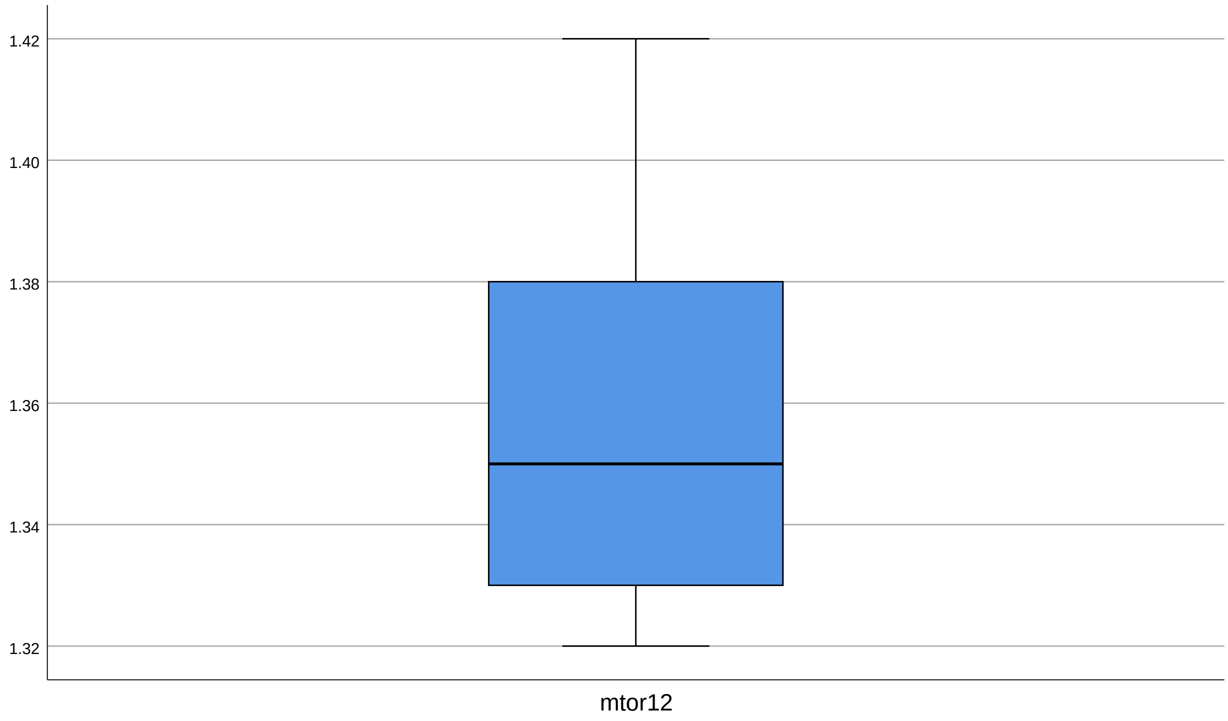
1.42
1.40
1.38
1.36
1.34
1.32
mtor12
